# Identification and Functional Annotation of Genes Related to Horses’ Performance: From GWAS to Post-GWAS

**DOI:** 10.3390/ani10071173

**Published:** 2020-07-10

**Authors:** Thayssa O. Littiere, Gustavo H. F. Castro, Maria del Pilar R. Rodriguez, Cristina M. Bonafé, Ana F. B. Magalhães, Rafael R. Faleiros, João I. G. Vieira, Cassiane G. Santos, Lucas L. Verardo

**Affiliations:** 1 Department of Animal Science, Universidade Federal dos Vales do Jequitinhonha e Mucuri, Diamantina 39100-000, Brazil; thayssalittiere1@gmail.com (T.O.L.); gustavo.castro@ufvjm.edu.br (G.H.F.C.); rodrigpilar@gmail.com (M.d.P.R.R.); crisbonafe@gmail.com (C.M.B.); ana.fabricia@ufvjm.edu.br (A.F.B.M.); joaoinaciozoo@hotmail.com (J.I.G.V.); cassianezootecnia@gmail.com (C.G.S.); 2 EQUINOVA Research Group, Universidade Federal de Minas Gerais, Belo Horizonte 31270-901, Brazil; faleirosufmg@gmail.com

**Keywords:** sport horse, genomics, gene network, systematic review

## Abstract

**Simple Summary:**

It is assumed that the athletic performance of horses is influenced by a large number of genes; however, to date, not many genomic studies have been performed to identify candidate genes. In this study we performed a systematic review of genome-wide association studies followed by functional analyses aiming to identify the most candidate genes for horse performance. We were successful in identifying 669 candidate genes, from which we built biological process networks. Regulatory elements (transcription factors, TFs) of these genes were identified and used to build a gene–TF network. Genes and TFs presented in this study are suggested to play a role in the studied traits through biological processes related with exercise performance, for example, positive regulation of glucose metabolism, regulation of vascular endothelial growth factor production, skeletal system development, cellular response to fatty acids and cellular response to lipids. In general, this study may provide insights into the genetic architecture underlying horse performance in different breeds around the world.

**Abstract:**

Integration of genomic data with gene network analysis can be a relevant strategy for unraveling genetic mechanisms. It can be used to explore shared biological processes between genes, as well as highlighting transcription factors (TFs) related to phenotypes of interest. Unlike other species, gene–TF network analyses have not yet been well applied to horse traits. We aimed to (1) identify candidate genes associated with horse performance via systematic review, and (2) build biological processes and gene–TF networks from the identified genes aiming to highlight the most candidate genes for horse performance. Our systematic review considered peer-reviewed articles using 20 combinations of keywords. Nine articles were selected and placed into groups for functional analysis via gene networks. A total of 669 candidate genes were identified. From that, gene networks of biological processes from each group were constructed, highlighting processes associated with horse performance (e.g., regulation of systemic arterial blood pressure by vasopressin and regulation of actin polymerization and depolymerization). Transcription factors associated with candidate genes were also identified. Based on their biological processes and evidence from the literature, we identified the main TFs related to horse performance traits, which allowed us to construct a gene–TF network highlighting TFs and the most candidate genes for horse performance.

## 1. Introduction

The horse was one of the first animals to be domesticated. There are records showing that the domestication process started around 6000 BC, when these animals stopped being considered as a food source and started being used for transportation, as a tool in agriculture and subsequently as an instrument of war [1]. In this way, horses were important for the formation of ancient civilizations. Currently, horses are used in various activities but are mainly used in outdoor leisure and sport activities. The global equine population was estimated at 59 million animals [2], with the annual economic impact of the global equine industry being estimated at U$300 billion dollars and generating more than 1.6 million full-time jobs [3].

These new roles and relationships between humans and horses have brought new opportunities for a healthy way of life for both humans and horses, and have resulted in a relevant agribusiness segment in the world economy. In this way, a better biological understanding is necessary to identify and select equine genes that are associated with athletic performance. Research on animal breeding, combined with prior knowledge of exercise physiology, can be the key to success in selecting horses with all the necessary phenotypic and physiological characteristics to successfully perform desirable leisure and sport activities, while minimizing the chance of developing skeletal muscle injuries due to inadequate body conformation [4].

The athletic performance of horses is a trait influenced by a large number of genes. However, this trait has been little studied at the genomic level, with few strong candidate genes identified for athletic performance in horses. Genome-wide association studies (GWAS) might allow the identification of genomic regions and contribute to a better understanding of performance traits of the main breeds of horses used today in the world. In addition, the integration of genomic data with gene network analysis can be a relevant strategy for unraveling molecular mechanisms, in that these networks can be used to expose shared metabolic pathways, annotations of biological processes, as well as highlighting transcription factors (TF) related to specific phenotypes [5,6,7]. From the understanding of these molecular mechanisms and biological processes, crossbreeding can be planned and directed to each breed and individual, so that animals with proven high performance can be selected.

Unlike in other species, gene–TF networks have so far not been applied to traits of economic importance in horses. In this context, the aim of this study was to (1) identify candidate genes associated with physiological adaptations in the physical conditioning of horses via systematic review, and (2) build biological process networks and gene–TF networks from the identified candidate genes aiming to highlight the most candidate genes for horse performance.

## 2. Material and Methods

### 2.1. Identification of Candidate Genes—Systematic Review

A systematic review was performed to identify candidate genes related to performance in horses. The articles were searched using the Web of Science (www.webofknowledge.com) search engine in September of 2019. The search queries consisted of combinations of keywords with the following criteria: (a) term related to the evaluated trait (“performance”, “exercise”, “lactate”, “energetic metabolism”, “muscle metabolism”); (b) association test (“GWAS”, “genome-wide association”); and (c) species or breed (“horse”, “Mangalarga Marchador”). It is important to note that the use of Mangalarga Marchador term was intended to search for a Brazilian national breed without compromising the final results, in that we used the “horse” keyword, and then focused on studies of horses. All combinations among the keywords of each criteria were performed using the quote symbol between the letters (“a”, “b”, “c”). In this way, all combinations between the keywords of each criteria were performed a total of 20 times.

Two independent judges (Researcher 1—M.d.P.R. and Researcher 2—T.O.L.) searched for peer-reviewed articles using the queries described above. Discrepancies in judgment were resolved by consensus among the judges. The first step for selecting articles was to check if the article contained, either in the abstract, in the title or in the keywords, the words used in the combinations. Those articles that fulfilled these criteria were selected. Secondly, duplicated articles were removed. An article was evaluated if the article (1) was peer-reviewed and published in English; (2) performed a GWAS for performance traits; (3) used a horse as a model; (4) provided consistent gene position and methodology used; and (5) had a full-text available. Those articles that met all the criteria described went on to the full-text review stage. From these, the phenotypes that were not related with performance traits in horses were removed from the functional analysis in order to reduce the complexity and heterogeneity of the evaluated traits. In addition, articles that did not present enough information about the associated markers or windows (marker names, “rs” marker accession number, or at least the complete genomic coordinates) for candidate gene identification were also excluded from this step.

### 2.2. Functional Annotation—Gene Networks

All articles that fulfilled the criteria described above were selected for functional analysis. Initially, all the associated markers and/or windows reported in each article were annotated as well as their position (bp) on the respective chromosomes. Thenceforth, genes mapped in the flanking regions (described in each article) around the associated markers or windows reported were annotated based on the assembly used in the respective articles (EquCab 2.0) through the GenBank database from the National Center of Biotechnology Information (NCBI). In addition, the observed genes were separated by groups, with each group corresponding to an article. Thus, it was possible to relate the candidate genes previously identified to different biological processes, using analyses performed group by group, and regulatory mechanisms such as transcription factors, which we analyzed group by group and in combination to highlight any shared regulatory elements between candidate genes.

The gene network highlighting the biological processes related to the candidate genes from each group was constructed using the ClueGO [8] application in the open source software platform Cytoscape [9]. This analysis was based on two-sided hypergeometric test and the Bonferroni correction. In addition, a search of promoter sequences of candidate genes was performed, aiming to identify possible regulatory elements associated with each gene.

Thus, promoter sequences (in FASTA format) were collected based on a flanking sequence of 3000 bp upstream and 300 bp downstream around genes’ transcription start sites [10] in the updated EquCab 3.0 assembly on the NCBI web site. In this step, non-coding RNA genes and pseudogenes were excluded. The EquCab 3.0 assembly was used in order to optimize the quality of the promoter sequence of the candidate gene. These data were submitted to the TFM-Explorer software, to identify the TF related with the candidate genes from each group. This software uses weighting matrices from the JASPAR database [11] to detect potential transcription factor binding sites (TFBS) and extracts significant clusters (TFBS regions of the selected gene sequences associated with a factor) by calculating a score function. This score threshold is chosen to generate a *p*-value ≤ 10^−3^ for each position for each sequence [12].

The given list of TFs obtained from each group was analyzed in Cytoscape software [7] using the Biological Networks Gene Ontology tool (BiNGO) [13] in order to determine significantly overrepresented functional gene ontology (GO) terms from hypergeometric tests and multiple test corrections (*p* < 0.05). Based on biological process overrepresented in BiNGO related with horse performance, as well as evidence from the literature review, we were able to identify the main TFs related to horse performance in each group (according their biological role and evidence from the literature), which allowed us to construct gene–TF networks. In order to analyze the gene–transcription factor relationship, the NetworkAnalyzer tool was used in Cytoscape software [8]. In this way, the most connected genes and TFs in the TF–gene network were determined according to the number of TFBS and, consequently, the number of connections/lines in each node (gene and TF). Genes with more TFBS for the most representative key TFs were highlighted in the TF–gene network. Finally, TF-networks provided a better functional understanding of genes and TFs associated with high performance in horses.

## 3. Results

### 3.1. Systematic Review

**Figure 1:**
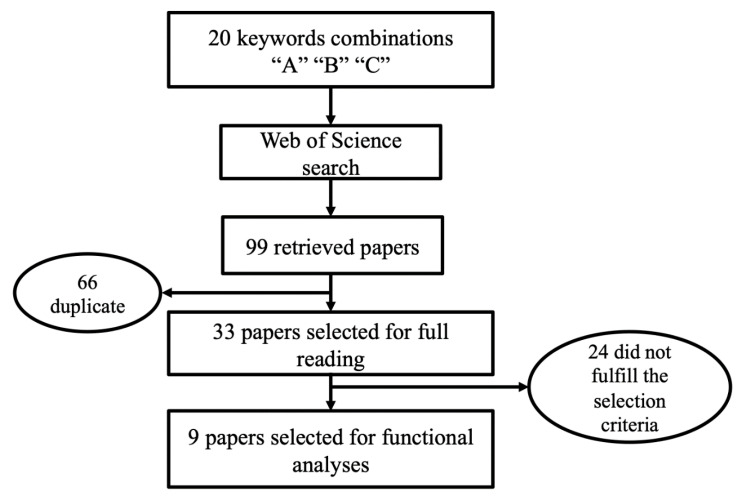
Flow chart displaying the pipeline used to select articles based on specific criteria to the systematic review.

The systematic review process is summarized in Figure 1. The search processes, performed with 20 keyword combinations (Table S1), returned a total of 99 articles (46 found by judge M.d.P.R. and 53 found by judge T.O.L.). Of these 99 articles, 66 were duplicates between keyword combinations and were removed. The remaining 33 articles proceeded to the full-text review step and 24 of them were removed due to not fulfilling the selection criteria (e.g., inconsistent gene position and methodology used). Thus, nine articles were selected and defined in groups (numbered from 1 to 9) for functional analysis, as shown in Table 1.

The traits analyzed in each article are related to the performance of athlete horses of different breeds across the world and they were used to perform a GWAS. The observed sample size used in each article varied from 112 up to 4499 genotyped animals, in which Illumina and Affymetrix SNP-chip data were used. All articles presented the genotyping and quality control as Hardy–Weinberg equilibrium and minor allele frequency rate (Table S2).

### 3.2. Functional Analysis

From significant SNPs/windows described in each article and their respective position (bp) on the chromosomes, we retrieved candidate genes, mapped in the flanking regions, windows or around associated markers also reported in each study (Table S3). Thus, gene networks were built highlighting the biological processes (Figures S1–S9), in which different biological processes (e.g., regulation of systemic arterial blood pressure by vasopressin, regulation of actin polymerization and depolymerization, as well as glucocorticoid secretion) were observed to be associated with candidate genes (e.g., *IFNAR1*, *AVPR1A*, *PTPRM*, *ANK1*, *GRIK2*, *GRM8*, *SELM*, *SLC39A12*, *SORCS3*, *PROK2*, *CHRND*, *IAPP*, *INPP5D*, *STK40*, *RC3H2*, *WASF1*, *PLAEKHH2*, *HAO1*, *PRCP*, *JAM3*, *ANKRD2*, *AOC1*, *SLC8B1*, *AGXT2*, *SDS*, *PASK* and *PSAT1*) identified in athletic horses from each group.

According to the TFM-Explorer program, 83 transcription factors were identified in total (Supplementary Materials—TFM_explorer_out). Of these, based on the biological processes (Supplementary Materials—Biological Process of TF) and literature review, 16 transcription factors most associated with horse performance were selected (Table 2).

**Figure 2:**
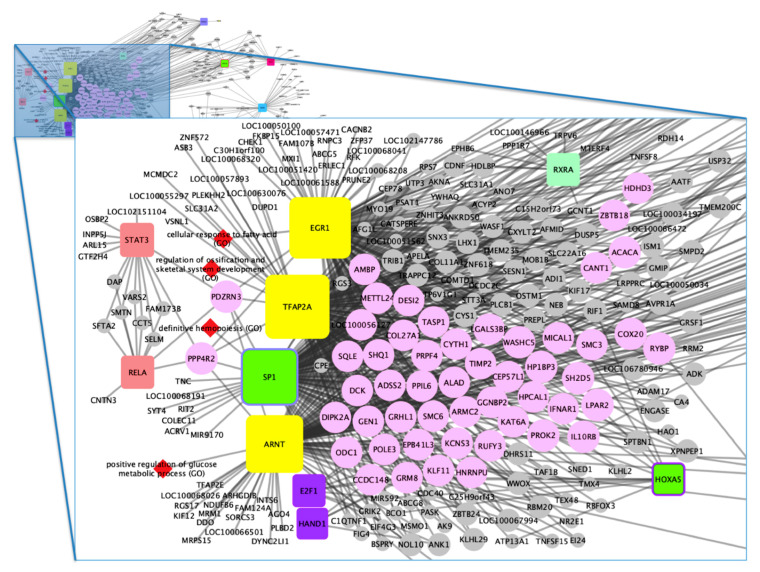
Horse performance gene–transcription factor (TF) network, focus 1. Rounded square nodes represent the TFs, wherein four main TFs are highlighted: EGR1, TFAP2A, SP1, and ARNT. Yellow nodes are associated with three or more groups, while those with two colors (interior and border) represent 2 groups, and nodes with one color represent only one group*. Red diamond nodes are the TF-related biological processes. Gray nodes are the identified candidate genes, whereas the main candidate genes related with horse performance are represented by light pink color. *Green nodes: group 1, purple nodes: group 2; salmon nodes: group 3; blue nodes: group 5; orange nodes: group 7; light green nodes: group 9.

**Figure 3:**
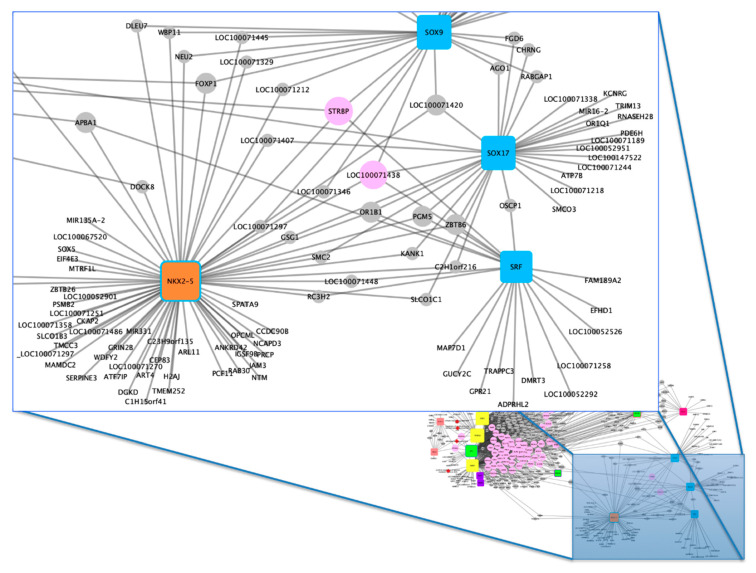
Horse performance gene–transcription factor (TF) network, focus 2. Rounded square nodes represent the TFs, wherein four main TFs are highlighted: NKX2-5, SRF, SOX17 and SOX9. Blue nodes are associated with genes from group 5, whereas the one with two colors (orange and blue border) is associated with groups 7 and 5, respectively. Gray nodes are the identified candidate genes, whereas the main candidate genes related with horse performance are represented by light pink color.

The main TFs associated with horse performance were used to generate a gene–TF network for each group (Supplementary Materials—Gene–TF networks). Based on the separate analyses, a merged network was constructed (Figure S10, Figure 2 and Figure 3) which enabled us to identify the most candidate genes for horse performance. Initially, 669 genes were identified in total, according to the selected articles in the systematic review. Of these, 53 genes were highlighted in the gene–TF network as more associated with performance traits in horses. Table 3 shows these genes and their respective TFs.

We can observe that the TFs early growth response 1 (EGR1), transcription factor AP-2 alpha (TFAP2A), aryl hydrocarbon receptor nuclear translocator (ARNT), and Sp1 transcription factor (SP1) had a greater number of binding sites associated with the genes. However, although EGR1, TFAP2A and ARNT are associated with three or more groups, SP1 TFs are associated only with two groups (groups 6 and 1). Among the genes that have a greater number of binding sites for TFs, we can highlight *IFNAR1*; *GRM8*; lysine acetyltransferase 6A (*KAT6A*); protein phosphatase 4 regulatory subunit 2 (*PPP4R2*); *PROK2*; *PDZ* domain containing ring finger 3 (*PDZRN3*); microtubule associated monooxygenase, calponin and LIM domain containing 1 (*MICAL1*); erythrocyte membrane protein band 4.1 like 3 (*EPB41L3*); *SHQ1*; H/ACA ribonucleoprotein assembly factor (*SHQ1*); armadillo repeat containing 2 (*ARMC2*) and olfactory receptor 1N2-like (*LOC100071438*) with possible roles in horse performance, as further discussed.

## 4. Discussion

Through the analysis of selected articles from the systematic review, we could observe that many countries, including Brazil, have carried out research in order to identify candidate genes related to the performance of athlete horses. This research can be related with the search for animals that present superior performance in several competitions, due to the popularization of equestrian sports worldwide, which makes the horse a fundamental part in the world economy.

Consequently, the genetics of several breeds of horses are being studied, since each breed has particularities to perform different sports activities. From the articles selected, it is possible to note that most research is focused on breeds that have greater representativeness in equestrian sports, such as the Quarter Horse (in sprint races), the Thoroughbred (horse racing), Lipizzan (dressage), and Arabian horse (endurance riding and dressage). These articles show that different traits have been related with horse performance. In this way, it is important to understand the traits of each breed and its relationship with horse performance through knowledge of the morphology and physiology of these animals. The need for more genomic studies in horses is also evident, with more breeds and standardized traits related with animal performance.

### 4.1. Gene–Biological Process Network

Based on candidate genes identified from selected articles, we built gene–biological processes networks aiming to observe enriched processes related to horse performance. Thus, we could identify the *AVPR1A* gene as being involved with regulation of systemic arterial blood pressure by vasopressin through the biological process network from group 1. The protein encoded by *AVPR1A* acts as a receptor for arginine vasopressin (AVP). Vasopressin is a powerful vasoconstrictor and it is important to control blood pressure during exercise in horses [39]. Researches indicated that an increase in plasma concentration of AVP is caused by exercise and it is correlated with the intensity and duration of the exercise in humans [40]. There is a significant increase in the AVP plasma concentration during high intensity exercise and volitional exhaustion, which exceeds the predicted levels concurrent with increases in volume or osmotic regulation [41]. Thus, we can suggest that it occurs not only in human athletes but also in horses that are adapted for high intensity exercises, such as the Quarter Horse, which is the breed evaluated in group 1. In this group we also identified the *IFNAR1* gene, related to positive regulation of cytokine secretion. According to Peake et al. [42], cytokines are important mediators of glucose and lipid metabolism and are involved with skeletal muscle hypertrophy and atrophy in humans. Cytokines act in a hormone-like manner during exercise, mediating metabolism in working skeletal muscle, angiogenesis, neurobiology and the liver and adipose tissue [43]. In horses, this gene was cited to be down-regulated in high-level animals immediately after endurance [44], suggesting a higher expression before exercise. Based on this, and considering that this gene was highlighted as candidate for high intensity exercise in horses, it is suggested that *IFNAR1* plays a role in horse performance.

In group 2, based on the gene–biological process network, we could highlight the *GRM8* and *GRIK2* genes, which are linked with the glutamate receptor signaling pathway. The *GRM8* gene belongs to group III of metabotropic glutamate receptors that are the G-protein coupled receptors superfamily [45], whereas the *GRIK2* gene belongs to the kainate family of ionotropic glutamate receptors [46]. Both receptors (metabotropic and ionotropic) are activated by L-glutamate, which is the main excitatory neurotransmitter in the central nervous system [47]. Moreover, glutamate receptors mediate the majority of the excitatory neurotransmission in the mammalian brain. Glutamate is involved in neural development, synaptic plasticity, memory, learning and other biological processes [48]. In horses, learning and behavioral processes can influence not only the performance but also its usefulness [49]. In addition, athletic activities performed by horses, such as show jumping, dressage, racing and carriage work require specialized training and good learning ability from the horse [50]. This group studied a sample of a racing line of Quarter Horses, which is characterized by great sprinting speed over short distances on straight tracks [15], which may be explained by action of these genes.

The *SELM* gene was highlighted in group 3. It is located in the endoplasmic reticulum and is associated with corticosterone secretion, which is a glucocorticoid class hormone. This class of hormones is associated with stress response and mobilization of energy reserves during physical activity by stimulating gluconeogenesis, increasing protein catabolism and promoting lipolysis of blood lipids [51]. Glucocorticoids plays an important role in aerobic metabolism, which can be associated with prolonged muscle activity. In this way, the secretion of these hormones may play a role in racehorses, such as the Thoroughbred.

In group 4, we could identify the *SLC39A12* gene, which is related to zinc ion transmembrane import. This gene encodes the ZIP12a zinc transporter, which performs zinc uptake and maintains cell zinc homeostasis in many species [52]. Zinc is a cofactor in nucleic acid, protein, carbohydrate and lipid metabolism. In addition to these functions, zinc presents antioxidant properties, such as its participation in the structure of the superoxide dismutase enzyme, in addition to being a potent stabilizer of cell membranes, structural proteins and cell signaling [53]. Intense physical exercise induces the excessive formation of reactive oxygen species associated with accelerated energy metabolism, which can contribute to tissue and cellular damage and impair the performance of athletes [54]. In this way, the antioxidant properties of zinc may prevent or reduce the effects caused by oxidative stress of athlete horses. Therefore, sports that obtain energy through aerobic metabolism are easier to promote the release of these substances compared to those that obtain energy through anaerobic metabolism. As a result, athletes of aerobic modalities suffer the consequences more in the presence of reactive oxygen species [55], as in the case of endurance horses like the Arabian horses studied in group 4. Thus, the *SLC39A12* gene might also be highlighted as a candidate gene in optimizing horses’ performance.

In addition, we could highlight the *PROK2* gene identified in groups 5 and 6. This gene encodes the prokinectin 2 protein composing the prokineticin signaling via activation of the G-protein-coupled receptor PK-R1, and induces vessel-like formation in the cultured cardiac endothelial cells independent of vascular endothelial growth factor up-regulation [56]. This gene is also associated with the regulation of smooth muscle contraction, such as that of blood vessels, which are basically made up of vascular smooth muscle cells and the endothelium [57]. Smooth muscle contractions allow alterations in the cross-sectional area of the arteries and thus provide a mechanism for regulating blood flow [58]. Visceral vasoconstriction occurs during exercise in order to decrease the blood flow in non-exercised organs and consequently, through vasodilation, an increase in blood flow to the muscles that are being exercised. Based on its role in optimizing the blood flow of exercised muscles and by the fact that it was identified in GWAS for horse performance, it is suggested as a candidate gene in our study.

In addition to *PROK2*, we also highlighted the *HAO1* gene in group 6, which is related to lipid and fatty acid oxidation. Lipid oxidation occurs in aerobic conditions and can produce about three times as much ATP as the oxidative phosphorylation of carbohydrates. During moderate exercise maintained for a long period, fatty acids are mobilized from adipose tissue (peripheral and intramuscular) through lipolysis and are used by skeletal muscle. In contrast, during high-intensity exercise, the release of fatty acids from adipose tissue is markedly decreased, followed by an increase in glucose availability and oxidation [59]. In group 6, the horse breed studied is the racing line of the Quarter Horse, a breed used for shorter distance races, which are classified as high-intensity exercise. Thus, the oxidation of carbohydrates predominates instead of lipid oxidation in this situation, corroborating the importance of *HAO1* in horse performance.

From the gene–biological process network of group 7, we could observe that the *PRCP* gene is associated with the kinin cascade. According to it gene ontology (GO:0002254), kallidin and bradykinin are final products that induce smooth muscle contraction, vasoconstriction and increased vascular permeability. Furthermore, researchers suggested that *PRCP* deficiency influences blood pressure and cardiac function [60,61]. In this way, this gene’s function may have a direct effect on the physiological phenotype (e.g., vasoconstriction and increased vascular permeability) relevant to exercise in horses.

In group 8, *ACO1* could be linked with the response to iron (II) ions. This gene is a bifunctional, cytosolic protein that exerts its function as aconitase and/or modulating intracellular iron homeostasis depending on iron availability [62]. Iron participates in several vital functions of the organism, such as the transport of oxygen and electrons and the synthesis of DNA. Most of the iron in the body is bound to proteins, such as heme compounds, which are complexes of iron and protoporphyrin present in hemoglobin and myoglobin [63]. Hemoglobin is responsible for transporting oxygen in the blood, whereas myoglobin is responsible for oxygen storage in the muscles. Intense and regular exercise promotes an increase in the synthesis of myoglobin and iron-containing enzymes, which—associated with the increase in the rate of erythropoiesis and losses in the digestive tract, urine and sweat—increases the body’s demand for iron. In this context, researchers evaluated the concentrations of iron, copper, zinc and manganese trace minerals in Purebred Lusitano athlete horses before and after exercise, and they concluded that short-term physical exercise is sufficient to generate sweating and splenic contraction capable of altering the serum concentrations of iron, copper, zinc and manganese [64]. Furthermore, physical activity can directly influence the requirement of microelements by horses subjected to a trotting routine and a gentle gallop [64]. Thus, the up- or down-regulation of *ACO1* may be related with horse performance differences.

In addition, we could observe the *PASK* gene in group 9, which is associated with polysaccharide biosynthetic process, regulation of glucagon secretion and regulation of glycogen metabolic process. Glucagon is a primary regulator of hepatic glucose production during fasting, hypoglycemia and exercise [65]. Its main function is to increase the concentration of glucose in the blood, through hepatic glycogenolysis and gluconeogenesis, and its secretion is mainly controlled by the plasma glucose level of the blood flowing through the pancreas [66]. These authors also suggested that there is a greater release of glucagon in exercise of longer duration, and in moderate exercise of short duration a decrease in its plasma levels is observed. In addition, aerobic exercises lead to a fall in serum insulin and a rise in glucagon concentrations, which protect against a severe decline in blood glucose levels [67].

### 4.2. Gene–TF Network 

Among the sixteen key TFs that were used to establish a gene–TF network, four were highlighted as the main TFs associated with horse performance: EGR1, TFAP2A, ARNT and SP1. Of these, TFAP2A had a greater number of binding sites associated with the studied genes. In addition, TFAP2A was associated with groups 1, 2, 6 and 9, and consequently with two breeds: Quarter Horse (groups 1, 2 and 6) and Mangalarga Marchador (group 9). TFAP2A is important for chondrogenic and skeletal development [26]. Furthermore, studies indicated that TFAP2A-knockout mice presented severe skeletal defects in growth and the development of the face and limbs [68]. In the same way, TFAP2A was described as a regulator for face and limb bud development in chickens [69].

In athlete horses, regardless of breed, malformation and development of the skeleton can compromise the performance and useful life of the animal in competitions, since conformation, especially of the hind limbs, determines the functional integrity and success of the gaits of these animals. In addition, poor conformation of limbs can cause lameness and produces abnormal strain in particular parts of the limbs of horses [70,71]. Likewise, researchers evaluated the prevalence of tarsal diseases in healthy Mangalarga Marchador horses at the national horse show and they concluded that these diseases can be related with small tarsal angles [72].

The ARNT was another TF highlighted in the gene–TF network and it is associated with genes of five groups (1, 4, 5, 6 and 8) and thus five horse breeds (Quarter Horse, Arabian horse, Norwegian-Swedish coldblooder trotter, Lipizzan and Franches Montagnes). In studies carried out on mice, researchers demonstrated that ARNT is important in the control of metabolism in β cells and the liver [25]. In this way, the expression of these TFs in the liver may lead to dysregulation of glucose homeostasis (increased gluconeogenesis) and lipid metabolism without increased ketogenesis.

It is known that lipids can be used as an energetic substrate for athlete horses. In low to moderate intensity exercise, there is a progressive increase in lipid oxidation with an increase in exercise duration, such as in endurance competitions. Arabian horses are the most used in this sport, and this is probably due to the composition of their muscle fibers and their particular ability to use lipids during submaximal exercise [73]. In addition, hepatic gluconeogenesis is essential to maintain the glycemia of horses in physical activity, especially of fasting horses, because the energy stored in muscles in the form of glycogen, used in muscle contraction, is relatively small [74]. Moreover, studies indicated that the rate of gluconeogenesis is higher with prolongation of physical effort [75]. 

EGR1 is related with groups 1, 6 and 9, and also with two breeds: Quarter Horse and Mangalarga Marchador. This TF regulates cholesterol biosynthetic gene expression in mice having extensive localization to the proximal promoters of cholesterol biosynthetic genes in response to insulin, suggesting that this TF regulates a number of genes in this pathway [28]. In the same way, an induction of EGR1 binding to cholesterol biosynthetic promoters follows high carbohydrate feeding [28]. It is also reported that a polymorphism in the human EGR1 promoter is associated with reduced serum cholesterol, as well as a higher ratio of high density lipoproteins (HDLs) to low density lipoproteins (LDLs) [76].

Horses subjected to intense exercises use lipids as the principal source of energy, which involves the breakdown of the fat reserves of the body. Thus, researchers evaluated the effect of an endurance ride on the serum biochemical profiles of Mangalarga Marchador horses, and observed that the serum cholesterol levels in these animals decreased gradually during the exercise [77]. It can be explained by the fact that the horses had been ridden during the synthesis of cortisol in the adrenal cortex [78]. Earlier studies indicated that serum cortisol levels increase considerably in response to high intensity exercise causing stress to the horse [79], and during stress 80% of the circulating cortisol derives from plasma cholesterol [80].

Finally, SP1 was also highlighted in the gene–TF network, and this TF was identified in two groups (1 and 6), both of which were associated with Quarter Horses. This TF plays a role at the onset of contractile activity of skeletal muscle cells, since it is an important mediator of mitochondrial biogenesis [81]. In addition to producing mitochondrial biogenesis, chronic contractile activity also evokes the remodeling of muscles and results in improved muscle function [82]. This research also affirmed that endurance training may be responsible for an increase in mitochondrial content, which leads to improvements in fatigue resistance, and consequently in improved performance.

Increased mitochondrial content may also occurs in horse breeds that have a greater number of type I muscle fibers (red endurance muscle), such as the Arabian horse, because this type of fiber contains a very high number of mitochondria, so they have greater aerobic capacity. However, type I fibers are slow to contract and relax. In contrast, breeds like the Quarter Horse have a major number of type II muscle fibers (white sprint muscle) that are more powerful to contract but much less resistant to fatigue, because this type of muscle fiber has fewer mitochondria. In this way, a greater proportion of type II muscle fiber than in other horse breeds was found in the gluteus medium of Quarter Horses [83]. However, all horse breeds present the two types of muscle fibers and their proportion in horses’ muscles may vary according to the type of exercise performed, either due to selection in breeding or as a result of training, and the type of muscle (postural and locomotor) [84].

Moreover, based on these main TFs, we could identify the most candidate genes for horse performance. From the 53 enriched genes in the gene–TF network, we were able to highlight the genes *KAT6A*, *PPP4R2*, *PDZRN3*, *MICAL1*, *SHQ1*, *IFNAR1*, *GRM8*, *LOC100071438* and *PROK2*.

*KAT6A* was previously related with horse performance because this gene may act as a transcriptional coactivator for RUNX2 (runt-related 75 transcription factor 2) [15]. RUNX2 is important for skeletal development and is related to processes of both intramembranous and endochondral ossification, including chondrocyte maturation, vascular invasion into the cartilage and bone formation by osteoblasts [85]. In addition, this TF regulates chondrocyte proliferation and differentiation [86], and an irregularity of chondrocyte development and maturation can alter endochondral ossification, leading to osteochondrosis [87]. In this context, the most important causes of poor performance and reduced usefulness of athlete horses are the diseases of the locomotor system [88]. These diseases may be related with stress on immature bones and joints, and are observed mainly in racehorses, since they start training for competitions very young [89]. Consequently, racehorses, such as the Thoroughbred and the Quarter Horse of the racing line, must have good conformation, in order to have speed over short distances and to stay sound under the stress of training and running at top speed. In this way, studies have demonstrated that the extent and maturity of the skeletal musculature is the principal characteristic that contributes to the ability of Thoroughbred to perform well in sprint races [90].

The *PPP4R2* and *PDZRN3* genes were also enriched in the gene–TF network and they are related to differentiation and maintenance of neuromotor functions [18]. *PPP4R2* is associated with the survival of motor neuron (SMN) protein, which affects modulation of skeletal muscle [91]. These researches showed that the protein product of *PPP4R2* is related to a motor disorder characterized by the progressive loss of motor neurons and spinal muscular atrophy through its regulatory interaction with survival of SMN. On the other hand, *PDZRN3* participates in embryo morphogenesis, aiding an important pathway related to the planar orientation and organization of highly branched vascular plexuses [92]. In addition, *PDZRN3* is essential in the differentiation of myoblasts into myotubes by acting either downstream or independently of myogenin [93]. Both *PPP4R2* and *PDZRN3* are associated with groups 3 and 6, which were identified in Thoroughbred and Quarter Horse breeds, respectively. 

Other genes also highlighted in group 6 are *MICAL1* and *SHQ1*. *MICAL1* is associated with actin depolymerization [94,95], which is related to many important cellular processes, including muscle contraction, cell motility, cell division and cytokinesis, movement of vesicles and organelles, cell signaling and establishment, as well as maintenance of junctions and cell shape [96]. Moreover, in humans, *MICAL* is required for normal actin organization in non-neural cells and regulates actin stress fibers [97]. It is also suggested that the generation of reactive oxygen species by *MICAL* proteins is crucial for their actin-regulatory function. Otherwise, *SHQ1* was described as a candidate for running performance in Thoroughbred horses [16]. This gene encodes an element necessary for the assembly of telomerase ribonucleoproteins [98]. In this way, telomerase activity in muscle stem cells is retained in old and age-specific telomere shortening [99].

*IFNAR1* (group 1), *GRM8* (group 2) and *PROK2* (groups 5 and 6) were highlighted in both gene–biological processes and gene–TF network, which may suggest that they are demonstrably related with horse performance, mainly with the breeds from the groups analyzed: Thoroughbred and Quarter Horse. Furthermore, other genes are related to these two breeds, such as *PPP4R2*, *PDZRN3* and *SHQ1*. It may be explained due to Thoroughbred being one of the horse breeds used in the formation of the racing line of Quarter Horses, and thus it is observed that the results of genetic studies involving these two breeds may be similar [19].

Furthermore, we observed that *LOC100071438* is also enriched in the gene–TF network. This gene is not well annotated yet, but encodes for olfactory receptor 1N2-like protein. Olfactory receptors are mainly expressed in olfactory sensory neurons and detect volatile odorants in smell [100]. However, some olfactory receptors are expressed in other tissues like heart [101], blood [102] and lung tissues [103]. Furthermore, there is evidence of olfactory receptors displaying distinct mRNA expression patterns during myogenesis and muscle regeneration [104]. Even though the olfactory receptor 1N2-like protein’s role has not been well annotated yet, it was first identified in a GWAS of horse performance [18] and was well enriched in our gene–TF network analysis, and thus has potential to be a candidate gene for horse performance.

Complex traits, such as horse performance, are subject to the interaction of a large number of genes regulated by a variety of TFs, many of them still to be identified. Different components of horse physiology may be important for determining the final horse performance according to their aim (e.g., in sprint races the Quarter Horse, Thoroughbreds in racing horses; and Arabian horses used for endurance riding and dressage), and different genes are therefore important and contribute to the observed genetic differences among the studied groups and breeds. Moreover, other factors such as temperament and motor neuron control may also be better considered to affect the final horse performance.

## 5. Conclusions

Candidate genes associated with physiological adaptations in the physical conditioning of horses were identified. Thus, the construction of gene networks enabled us to identify the main TFs (e.g., TFAP2A, ARNT, EGR1 and SP1) and the most candidate genes (e.g., *PPP4R2*, *PDZRN3*, *IFNAR1* and *LOC100071438*) associated with horse performance. Those genes and TFs are suggested to play a role in the studied traits through biological process related with exercise performance (e.g., positive regulation of glucose metabolic process, regulation of vascular endothelial growth factor production, skeletal system development, definitive hemopoiesis, cellular response to fatty acid and cellular response to lipid). In summary, we highlighted 53 genes that may provide insights into the genetic architecture underlying horse performance of different breeds around the world.

## Figures and Tables

**Table 1:** Country, breed, sample constitution and trait information from selected articles.

Article (Publication Year)	Group	Trait	Material	Breed	Country
Meira et al. (2014a) [14]	1	Morphometric traits (weight, rump length and body length)	Blood	Quarter horse	Brazil
Meira et al. (2014b) [15]	2	Speed index (SI)	Blood	Quarter horse	Brazil
Shin et al. (2015) [16]	3	Estimated breeding value (EBV) of race time	Blood	Thoroughbred	Korea
Ricard et al. (2017) [17]	4	Total race distance, average race speed and finishing status (qualified, eliminated or retired)	Blood	Arabian and crossed Arabian	France
Velie et al. (2018) [18]	5	Harness racing success (career earnings, best km time (s) and number of gallops)	Hair and/or blood	Norwegian-Swedish coldblood trotter	Sweden
Pereira et al. (2018) [19]	6	Maximum speed index	Blood	Quarter Horse	Brazil
McGivney et al. (2019) [20]	7	Racecourse starts, durability traits	Hair and/or blood	Thoroughbred	16 countries
Gmel et al. (2019) [21]	8	Joint angles (poll, elbow, carpal, fetlock (front and hind), hip, stifle and hock)	Blood and/or sperm	Franches-Montagnes and Lipizzan	Switzerland, Austria, Croatia, Slovakia, and Hungary
Bussiman et al. (2020) [22]	9	Gait type	Blood	Mangalarga Marchador	Brazil

**Table 2:** Main Transcription factors (TFs). Most representative TFs, associated with genes identified for each group, based on their biological processes and the literature review.

TF	Group	Biological Process	Literature Review *
HOXA5	1 and 2	Respiratory system process and cartilage morphogenesis	Lung regulatory signaling pathways and tracheal cartilage patterning [23]
HIF1A	1 and 8	Response to oxidative stress and response to hypoxia	Skeletal muscle’s response to endurance training [24]
ARNT	1, 4, 5, 6 and 8	Positive regulation of glucose metabolic process and regulation of vascular endothelial growth factor production	Role in gluconeogenesis and lipogenic gene expression [25]
TFAP2A	1, 2, 6, and 9	Skeletal system development and regulation of bone mineralization	Regulation of cartilage and skeletal development [26]
SP1	1 and 6	Definitive hemopoiesis	Muscle cell differentiation [27]
EGR1	1, 6 and 9	Cellular response to fatty acids and cellular response to lipids	Regulation of cholesterol biosynthetic gene expression [28]
HAND1	2	Cardiac ventricle development and cardiac chamber development	Cardiomyocyte proliferation and cardiac development [29]
E2F1	2	Response to lipids and to fatty acids	Muscle oxidative metabolism [30]
STAT3	3	Response to cytokine stimulus and inflammatory response	Muscle hypertrophy following resistance exercise [31]
RELA	3	Regulation of cartilage development and negative regulation of protein catabolic process	Transcriptional regulation during skeletal growth and osteoarthritis development [32]
SOX9	5	Cartilage development and regulation of skeletal muscle fiber development	Regulation of chondrogenesis [33]
SOX17	5	Cardiac endothelium cell differentiation and heart formation	Specification of endocardium cells and heart development [34]
SRF	5	Regulation of muscle contraction and regulation of muscle system process	Skeletal muscle hypertrophy [35]
FOXC1	6	Cardiac muscle cell proliferation and heart growth	Morphogenesis of the cardiac outflow tract [36]
SOX2	8	Lung morphogenesis and detection of mechanical stimulus	Branching morphogenesis and epithelial cell differentiation [37]
RXRA	9	Cardiac muscle tissue growth and heart growth	Myocardial growth and coronary artery formation [38]

* The studies cited are only a sample of the vast literature available.

**Table 3:** Genes highlighted in gene–TF network and their respective TFs and group.

Article (Publication Year)	Group	TF	Gene
Meira et al. (2014a) [14]	1	ARNT, TFAP2A, SP1, EGR1, HIF1A	*HP1BP3, SH2D5, IL10RB*
		ARNT, TFAP2A, SP1, EGR1, HIF1A, HOXA5	*LPAR2*
		TFAP2A, SP1, EGR1, HOXA5	*IFNAR1*
Meira et al. (2014b) [15]	2	TFAP2A, E2F1, HOXA5, HAND1	*GRM8, CCDC148, KAT6A*
Shin et al. (2015) [16]	3	STAT3, RELA	*PDZRN3, PPP4R2*
Ricard et al. (2017) [17]	4	ARNT	*-*
Velie et al. (2018) [18]	5	ARNT, NKX2-5, SOX9, SRF	*STRBP*
		ARNT, NKX2-5	*RYBP*
		ARNT, SOX17	*PROK2*
		NKX2-5, SOX9, SOX17, SRF	*LOC100071438*
Pereira et al. (2018) [19]	6	ARNT, TFAP2A, SP1, FOXC1	*HDHD3*
		ARNT, TFAP2A, EGR1, FOXC1	*ZBTB18*
		ARNT, TFAP2A, SP1, EGR1, FOXC1	*ACACA*
		ARNT, TFAP2A, SP1, EGR1	*EPB41L3, ARMC2, CEP57L1, PPIL6, CYTH1, GEN1, SMC6, SHQ1, RYBP, PROK2, DIPK2, ODC1, HPCAL1, KLF11, GRHL1, PPP4R2, PRPF4, ALAD, POLE3*
		ARNT	*GGNBP2, PDZRN3*
Pereira et al. (2018) [19]	6	ARNT, TFAP2A, SP1, EGR1	*COL27A1, DCK, SQLE, DESI2, ADSS2, HNRNPU, METTL24, TIMP2, TASP1, LOC100056127, KCNS3, RUFY3, LGAL53BP, AMBP*
		ARNT, TFAP2A, SP1, EGR1, FOXC1	*COX20, WASHC5*
		TFAP2A, SP1, EGR1, FOXC1	*MICAL1, SMC3*
		FOXC1	*CANT1*
McGivney et al. (2019) [20]	7	NKX2-5	*-*
Gmel et al. (2019) [21]	8	ARNT, HIF1A, SOX2	*-*
Bussiman et al. (2020) [22]	9	TFAP2A, EGR1, RXRA	*-*

## Supplementary Materials

The following are available online at https://www.mdpi.com/2076-2615/10/7/1173/s1. The legends and heads of supplementary figures and tables are specified in each file. **Figure S1.** Functional network between genes and biological processes, associated with horse performance of Group 1. Different colors are associated with different genes and their respective biological processes. The node size represents the term enrichment significance from ClueGo. The most significant term per subnetwork is shown in bold. **Figure S2.** Functional network between genes and biological processes, associated with horse performance of Group 2. Different colors are associated with different genes and their respective biological processes. The node size represents the term enrichment significance from ClueGo. The most significant term per subnetwork is shown in bold. **Figure S3.** Functional network between genes and biological processes, associated with horse performance of Group 3. Different colors are associated with different genes and their respective biological processes. The node size represents the term enrichment significance from ClueGo. The most significant term per subnetwork is shown in bold. **Figure S4.** Functional network between genes and biological processes, associated with horse performance of Group 4. Different colors are associated with different genes and their respective biological processes. The node size represents the term enrichment significance from ClueGo. The most significant term per subnetwork is shown in bold. **Figure S5.** Functional network between genes and biological processes, associated with horse performance of Group 5. The figure shows zoom in the main biological processes terms and genes. Different colors are associated with different genes and their respective biological processes. The node size represents the term enrichment significance from ClueGo. The most significant term per subnetwork is shown in bold. **Figure S6.** Functional network between genes and biological processes, associated with horse performance of Group 6. The figure shows zoom in the main biological processes terms and genes. Different colors are associated with different genes and their respective biological processes. The node size represents the term enrichment significance from ClueGo. The most significant term per subnetwork is shown in bold. **Figure S7.** Functional network between genes and biological processes, associated with horse performance of Group 7. Different colors are associated with different genes and their respective biological processes. The node size represents the term enrichment significance from ClueGo. The most significant term per subnetwork is shown in bold. **Figure S8.** Functional network between genes and biological processes, associated with horse performance of Group 8. The figure shows zoom in the main biological processes terms and genes. Different colors are associated with different genes and their respective biological processes. The node size represents the term enrichment significance from ClueGo. The most significant term per subnetwork is shown in bold. **Figure S9.** Functional network between genes and biological processes, associated with horse performance of Group 9. Different colors are associated with different genes and their respective biological processes. The node size represents the term enrichment significance from ClueGo. The most significant term per subnetwork is shown in bold. **Figure S10.** Horse performance Gene-Transcription Factor (TF) network. Rounded squares nodes represent the TFs. Yellow nodes are associated with three or more groups, while those with two colors (interior and border) represent 2 groups, and nodes with one color represent only one group*. Red diamond nodes are the TF related biological process. Gray nodes are the identified candidate genes, while the main candidate genes related with horse performance are represented by light pink color. *Green nodes: group 1, purple nodes: group 2; salmon nodes: group 3; blue nodes: group 5; orange nodes: group 7; light green nodes: group 9. **Biological Process of TF**. Supplementary tables of each group presenting the gene ontology identification (GO ID), gene ontology description, p-values, corrected p-values, frequency of the clusters and total frequency of transcription factors (TF) identified on each group. **Gene-TF networks**. Gene-Transcription Factor (TF) networks of genes identified on each Group. **TFM_explorer_out**. TFM-Explorer program outputs presenting the list of transcription factors associated with each group set of genes. **Table S1**. Number of articles identified by each judge (1 and 2)* according each combination of terms and their respective references. **Table S2.** Genotyping and quality control summary for the selected articles. The quality control performed in each article through the analysis of Hard-Weinberg Equilibrium (HWE), Call Frequency (CR), and Minor Allele Frequency (MAF). All the information were extracted from each article. **Table S3.** Genome-wide association studies summary for the selected articles and gene annotation. The number of SNPs/window were extracted from each article, as well as the respective Chromosome (Chr), position in base pair (bp), and candidate gene annotation.
