# Supplementary material for: Identification and Functional Annotation of Genes Related to Horses’ Performance: From GWAS to Post-GWAS
Source: Animals (Basel). 2020 Jul 10;10(7):1173. doi: 10.3390/ani10071173 (PMC7401650; doi:10.3390/ani10071173)
Supplement: Supplementary file 1 [file animals-10-01173-s001.zip › supplementarymaterials/Supplementary tables/Table S2.pdf]

Table S2. Genotyping and quality control summary for the selected articles. The quality control performed in each article through the analysis of Hard-Weinberg Equilibrium (HWE), Call Frequency (CR), and Minor Allele Frequency (MAF). All the information were extracted from each article.

Article (publication year)	Sample size	Traits	SNP-chip (number of markers used)	Approach	GWAS sig. criteria (value)	Quality control (removed markers)		
						HWE	CR	MAF
Meira *et al*. (2014a)	188	Morphometric traits (weight, rump length, and body length)	Illumina EquineSNP50 Genotyping BeadChips (54,602)	Mixed model	P-value	P < 0.001	<0.95	<0.05
Meira *et al*. (2014b)	112	Speed index	Illumina EquineSNP50 Genotyping BeadChips (54,602)	Mixed model	P-value	P < 1 x 10-3	<0.9	<0.05
Shin *et al*. (2015)	1156 (240 stage 1 and 916 stage 2)	Estimated breeding value (EBV) of race time	Illumina EquineSNP50 Genotyping BeadChips (54,602)	Joint analyses	P-value	P < 0.001	-	<0.05
Ricard *et al*. (2017)	597	Total race distance, average race speed and finishing status (qualified, eliminated or retired)	Illumina Equine SNP-74K chip (56,200)	3 models (Mixed model SNP based and haplotype based)	P-value	P > 10−8	≥80%	≥ 0.01
Velie *et al*. (2018)	661	Career earnings, best km time (s) and number of gallops	670 K Axiom Equine Genotyping Array	Mixed model-structured association approach	P-value	P < 1e-10	-	<0.05

Table S2. *Cont.*

Pereira *et al*. (2018)	360	Maximum speed index	Illumina EquineSNP50 Genotyping BeadChips (54,602) 120 animals (2011) / Illumina Equine SNP70 Genotyping BeadChips 65k 240 animals (2015)	Mixed model; single-step genomic best linear unbiased prediction (ssGBLUP) method	Total genetic variance (gVar)	P < 1 x 10-5	<0.9	<0.05
McGivney *et al*. (2019)	4499	Racecourse starts	Illumina EquineSNP50 Genotyping BeadChips (54,602), Illumina Equine SNP70 BeadChip 65k or Affymetrix AxiomTM Equine 670K SNP	Random forests with mixed effects	P-value	-	<0.95	<0.05
Gmel *et al*. (2019)	524	Joint angles (poll, elbow, carpal, fetlock (front and hind), hip, stifle, and hock	Affymetrix equine 670K SNP	Mixed model	P-value	P < 0.0001	<0.90	<0.05
Bussiman *et al*. (2020)	240	Gait type	Illumina Equine SNP70 Genotyping BeadChips 65k (48 animals - 2015) and Axiom® Equine Genotyping Array (192 animals)	Bayesian approach, Cluster and PCA, and Fivefold cross-validation	Additive genetic variance	P > 0.15	<0.95	≤ 0.01
