# Supplementary material for: Identification and Functional Annotation of Genes Related to Horses’ Performance: From GWAS to Post-GWAS
Source: Animals (Basel). 2020 Jul 10;10(7):1173. doi: 10.3390/ani10071173 (PMC7401650; doi:10.3390/ani10071173)
Supplement: Supplementary file 1 [file animals-10-01173-s001.zip › supplementarymaterials/Supplementary tables/Table S3.pdf]

Table S3. Genome-wide association studies summary for the selected articles and gene annotation. The number of SNPs/window were extracted from each article, as well as the respective Chromosome (Chr), position in base pair (bp), and candidate gene annotation.

Article (publication year)	Trait	Breed	#SNPs/window	Significant SNPs/Region	Chr	Position*** in bp/start-end	Candidate gene annotation	Candidate genes
Meira et al. (2014a)	morphometric traits (weight, rump length, and body length)	Quarter Horse	13	rs68562545	2	98900000	100 kb upstream and downstream to each SNP	EIF4G3, HP1BP3, SH2D5, KIF17
				rs68614112	2	103700000		ANKRD50
				rs68626382	2	33500000		
				rs68488737	3	26700000		WWOX
				rs68488737	3	26700000		WWOX
				rs68559987	3	29100000		GAN, LOC102150591*, BCO1
				rs68625621	3	15000000		
				rs68714893	6	79400000		AVPR1A, LOC106783311*
				rs68762127	7	34100000		CHEK1, ACRV1, LOC100630076, STT3A, EI24, LOC100630107
				rs68795337	8	32800000		PTPRM
				rs68802762	9	18500000		MCMDC2, LOC100052030, LOC102147850*
				rs69315059	21	4100000		LOC100054881, ATP13A1, LOC106782338*, GMIP, LPAR2 (PBX4)
				rs69402054	26	29900000		IFNAR2, IL10RB, LOC102150829*, IFNAR1
Meira et al. (2014b)	Speed index	Quarter Horse (racing)	8	rs68593122	2	97634986	100 kb upstream and downstream to each SNP	
				rs68593141	2	97642160		-
				rs69498497	4	82402787		GRM8, MIR592

				rs68879046	10	52188059		GRIK2
				rs69127140	18	33671391		NEB, RIF1
				rs69134578	18	36917870		-
				rs69194990	18	39128343		CCDC148
				rs69517755	27	3522223		ANK1, KAT6A
Shin et al. (2015)	Estimated breeding value (EBV) of race time	Thoroughbred	28	BIEC2_906777	5	40747218	within or 10 kb upstream and downstream to each SNP	LOC102151104
				BIEC2_1026019	8	5906904		INPP5J, SELM, SMTN
				BIEC2_1026200	8	6126833		OSBP2
				BIEC2_1029757	8	11590300		-
				BIEC2_330101	16	14185461		-
				BIEC2_330360	16	15550375		-
				BIEC2_330495	16	16289366		CNTN3
				BIEC2_330509	16	16334663		CNTN3
				BIEC2_330558	16	16948655		PDZRN3
				BIEC2_330572	16	16993987		PDZRN3
				BIEC2_330575	16	16999220		PDZRN3
				BIEC2_330578	16	17001997		PDZRN3
				BIEC2_330677	16	17421511		PPP4R2
				BIEC2_330691	16	17546589		GXYLT2
				BIEC2_330725	16	17763943		-
				BIEC2_330739	16	17790340		-
				BIEC2_527753	20	29897398		VARS2, SFTA2, GTF2H4
				BIEC2_527879	20	30126579		-
				BIEC2_529755	20	32127869		-
				BIEC2_529760	20	32131071		-

				BIEC2_554645	21	18154976		ARL15
				BIEC2_554739	21	18348602		-
				BIEC2_568963	21	47502588		-
				BIEC2_569862	21	48967346		DAP
				BIEC2_570062	21	49384234		CCT5, FAM173B
				BIEC2_570485	21	49894191		-
				BIEC2_732151	28	17405077		-
				BIEC2_814518	30	3869336		-
Ricard et al. (2017)	Total race distance, average race speed and finishing status	Arabian and crossed Arabian	6	BIEC2_11782	1	25715334	within	SORCS3
				BIEC2_1022884	6	79312770		LOC106783311*
				BIEC2_363958	7	6283266		-
				BIEC2_977605	16	79466436		-
				BIEC2_755603	29	17580597		SLC39A12
				BIEC2_755604	29	17580992		SLC39A12
Velie et al. (2018)	Career earnings, Best km time (s) and Number of gallops	Norwegian-Swedish coldblooder trotter	32	AX-103445942	1	151919692	500 kb upstream and downstream to each SNP	C1H15orf41, DPH6
				AX-104373992	1	162993722		STXBP6, LOC100052901, LOC100052951, LOC100052742*, LOC100052638, LOC100052689, LOC100052582, LOC100052526, LOC100049805, LOC100052229, LOC106782233*, LOC102150430*, LOC100052292, LOC106782231*, LOC106782222*, LOC100033969, LOC100050554*, LOC102150071*, LOC100147522, LOC100052107*
				AX-104568609	1	159285045		**

Velie et al. (2018)	Career earnings, Best km time (s) and Number of gallops	Norwegian-Swedish coldblooder trotter	32	AX-103803214	2	21466714	500 kb upstream and downstream to each SNP	OSCP1, LOC102150623*, CSF3R, STK40, MRPS15, LSM10, THRAP3, SH3D21, EVA1B, AGO3, COL8A2, TRAPPC3, MAP7D1, LOC102150891*, ADPRHL2, TEKT2, LOC100055196(AGO4), LOC106782791*, LOC100055148(AGO1), CLSPN, C2H1orf216, PSMB2, TFAP2E, NCDN, KIAA0319L, LOC102147570*, ZMYM4
				AX-104450418	2	21311680		OSCP1, LOC102150623*, CSF3R, STK40, MRPS15, LSM10, THRAP3, SH3D21, EVA1B, AGO3, COL8A2, TRAPPC3, MAP7D1, LOC102150891*, ADPRHL2, TEKT2, LOC100055196(AGO4), LOC106782791*, LOC100055148(AGO1), CLSPN, C2H1orf216, PSMB2, TFAP2E, NCDN, KIAA0319L, LOC102147570*
				AX-103090138	6	42063985		GUCY2C, WBP11, ERP27, ARHGDIB, PDE6H, C6H12orf60, SMCO3, ART4, MGP, LOC100063835 (H4-16), LOC102150778 (H2AJ) , ATF7IP, PLBD1, LOC102150749*
				AX-103248294	6	49512490		SOX5
				AX-104307051	6	41329519		GRIN2B, EMP1, LOC102150193*, GSG1, LOC102150077*
				AX-104494389	6	20020914		LOC100065342, USP40, DGKD, ATG16L1, INPP5D, NEU2, NGEF, GIGYF2, KCNJ13, LOC106783256*, EFHD1, LOC106783248*, LOC106783249*, ECEL1, CHRND, PRSS56, EIF4E2, CHRNG
				AX-104611735	6	47132529		PDE3A, SLCO1A2, IAPP, LOC100068155 (SLCO1B3), SLCO1C1
				AX-104711589	6	41462481		GRIN2B, ATF7IP
				AX-104117851	7	65266179		LOC100630698*, MIR708, LOC100062728 (TENM4)

Velie et al. (2018)	Career earnings, Best km time (s) and Number of gallops	Norwegian-Swedish coldblooder trotter	32	AX-104865129	16	18366321	500 kb upstream and downstream to each SNP	RYBP, LOC102148599*, LOC102148566*, MIR9170, LOC106781771*, EIF4E3, LOC102148998*, PROK2, FOXP1
				AX-104219924	17	19525955		-
				AX-104538418	17	21083126		LOC102150380*, LOC102150326*, LOC106781872*, TRIM13, LOC102150424*, SPRYD7, KCNRG, MIR3613, MIR16-2, KPNA3, ARL11, LOC102150635*, EBPL, LOC102150605*, LOC106781885*, MIR15A, RCBTB1
				AX-104591507	17	20813164		RNASEH2B, LOC102150008*, DLEU7, LOC102150326*, LOC106781872*, TRIM13, LOC102150424*, SPRYD7, KCNRG, MIR3613, MIR16-2, MIR15A
				AX-104645782	17	19318167		LOC100054677*, LOC100630841, LOC106781883*, VPS36, THSD1, CKAP2, NEK5, NEK3, ALG11, ATP7B, CCDC70, DHRS12, WDFY2, LOC106781884*, INT56
				AX-102964033	23	22423197		DMRT1, LOC102147582*, KANK1, LOC106782466*, DOCK8, LOC100050598(CBWD1), PGM5, TMEM252, LOC100057291
				AX-102982528	23	23324996		DMRT1, LOC100147177(DMRT3), DMRT2
				AX-103166989	23	22496787		DMRT1, LOC102147582*, KANK1, LOC106782466*, DOCK8, LOC100050598(CBWD1), PGM5, TMEM252, LOC100057291, LOC100147177

Velie et al. (2018)	Career earnings, Best km time (s) and Number of gallops	Norwegian-Swedish coldblooder trotter	32	AX-103261370	23	22522071	500 kb upstream and downstream to each SNP	DMRT1, LOC100147177 (DMRT3), LOC102147582*, KANK1, LOC106782466*, DOCK8, LOC100050598(CBWD1), LOC100057291(FOXD4), PGM5, TMEM252
				AX-103287280	23	21064571		FXN, LOC106782465*, TJP2, FAM189A2, APBA1, PTAR1, C23H9orf135, MAMDC2, SMC2, LOC106782493*
				AX-103530176	23	22464604		DMRT1, LOC102147582*, KANK1, LOC106782466*, DOCK8, LOC100050598 (CBWD1), PGM5, TMEM252, LOC100057291
				AX-103762427	23	22461979		DMRT1, LOC102147582*, KANK1, LOC106782466*, DOCK8, LOC100050598(CBWD1), PGM5, TMEM252, LOC100057291
				AX-104144838	23	23333501		DMRT1, LOC100147177(DMRT3), DMRT2

Velie et al. (2018)	Career earnings, Best km time (s) and Number of gallops	Norwegian-Swedish coldblooder trotter	32	AX-104268231	23	21689609	500 kb upstream and downstream to each SNP	PGM5, TMEM252, FAM122A, PIP5K1B, LOC106782465*, FXN, TJP2, FAM189A2, APBA1
				AX-104634248	23	21857316		PGM5, LOC100050598(CBWD1), LOC100057291, TEM252, FAM122A, PIP5K1B, LOC106782465*, FXN, TJP2, FAM189A2
				AX-103305676	25	26866219		LOC100071486, LOC100071469, LOC100071460, LOC100071448, LOC100071445, LOC100071420, LOC100071438, LOC106782596*, LOC100071407, LOC100071402, LOC100071358, LOC100071365, LOC100071352(OR1N1), LOC100071338, LOC100071346, LOC100071332, LOC100071329, LOC100071311, LOC100071297, LOC100071264, LOC100071270, LOC100071275(OR1B1), LOC100071278(OR1Q1), LOC100071227, LOC100071251, LOC100067520, LOC100071258, LOC100071218, LOC100071244, LOC100071212, LOC100071189(OR5C1), LOC100067499(OR1K1), LOC100071170, PDCL, RC3H2, ZBTB26, ZBTB6, RABGAP1, GPR21, STRBP
				AX-103147507	28	22153465		NEDD1, LOC102149424*, LOC106782711*, MIR135A-2
				AX-104828170	28	20006740		NDUFA12, PLXNC1, CEP83, TMCC3, NR2C1, FGD6, MIR331
				AX-103734745	29	24530437		
				AX-104642194	31	14300483		MTRF1L, FBXO5, SYNE1, MYCT1, VIP, RGS17, LOC106782928*, LOC106782927*

Pereira et al. (2018)	Maximum speed index	Quarter Horse (racing)	33 SNPS windows of 1 Mb	SS1_REG01	1	20721039 – 21689628	within[a]	DUSP5, ADD3, XPNPEP1, MXI1, SMC3, RBM20
				SS1_REG02	1	61826124 – 62818065		ADK, KAT6B, DUSP13, SAMD8,COMTD1, VDAC2, DUPD1, LOC102149419*, LOC106782060*
				SS1_REG03	2	69370047 – 70369774		MSMO1, KLHL2, TMEM192, CPE, APELA, LOC100061588, LOC100629609, LOC100068191, LOC100068208, LOC100068320
				SS1_REG04	3	65380573 – 66369357		LOC100066359, LOC100066444, LOC100066472, LOC100066501, LOC106783008*, LOC100067994, LOC100068026, LOC100068012, LOC100068026, LOC100068041, LOC100146977
				SS2_REG01	3	63351099 – 64348556		GC, DCK, MOB1B, GRSF1, RUFY3, UTP3, ENAM, JCHAIN, SLC4A4
				SS2_REG02	5	62937896 – 63920127		COL11A1, RNPC3, LOC100049851, LOC102147595*
				SS1_REG05	8	30526194 – 31504074		LOC106780945*, LOC102147433*, L3MBTL4, TMEM200C, LOC106780946, LOC100059101(EPB41L3)
				SS1_REG06	8	62566792 – 63559486		RIT2, SYT4
				SS2_REG03	9	66879753 – 67826611		ZNF572, SQLE, KIAA0196(WASHC5), NSMCE2, TRIB1, LOC106780992*, LOC106780991*

Pereira et al. (2018)	Maximum speed index	Quarter Horse (racing)	33 SNPS windows of 1 Mb	SS1_REG07	10	57990860 – 58961242	within[a]	NR2E1, SNX3, LACE1(AFG1L), FOXO3, ARMC2, SESN1, CEP57L1, OSTM1, LOC102151001*
				SS1_REG08	10	58997563 – 59976633		PPIL6, SMPD2, MICAL1, ZBTB24, FIG4, WASF1, CDC40, METTL24, DDO, LOC102147700*, LOC102147733*, LOC102147786, AK9, LOC100630272(CD164), LOC106782267*, SLC22A16
				SS2_REG04	10	58735380 – 59710571		ARMC2, SESN1, CEP57L1, PPIL6, SMPD2, MICAL1, ZBTB24, FIG4, WASF1, AK9, LOC100630272(CD164), LOC106782267*, LOC102147733*, LOC102147700*, LOC102147786
				SS1_REG09	11	3345863 – 4341072		RBFOX3, ENGASE, C1QTNF1, CANT1, LGALS3BP, TIMP2, USP36, CYTH1, DNAH17, PGS1, SOCS3, TMEM235, AFMID
				SS1_REG10	11	35523097 – 36481549		CA4, ZNHIT3, MYO19, PIGW, GGNBP2, DHRS11, MRM1, LHX1, AATF, ACACA, LOC100071389*, USP32
				SS1_REG11	15	45572062 – 46563225		SPTBN1, ACYP2, LOC106781739*, ASB3, CHAC2, PSME4, ERLEC1, GPR75, LOC106781738*, C15H2orf73, LOC102148694*
				SS1_REG12	15	46601694 – 47575761		LOC102149170*
				SS1_REG13	15	76296526 – 77289510		KCNS3, MSGN1, GEN1, SMC6, VSNL1, RDH14, LOC100056590

Pereira et al. (2018)	Maximum speed index	Quarter Horse (racing)	33 SNPS windows of 1 Mb	SS1_REG14	15	82855154 – 83838250	within[a]	NOL10, ODC1, HPCAL1, RRM2, KLF11, GRHL1, TAF1B, YWHAQ, ADAM17, LOC106781712*, LOC102149884(CYS1), LOC102149855*
				SS1_REG15	15	87766362 – 88753736		ALLC, COLEC11, RPS7, ADI1, TRAPPC12, DCDC2C, LOC102147742*
				SS2_REG05	15	53973327 – 54889258		SLC3A1, PREPL, PPM1B, LRPPRC, ABCG8, ABCG5, DYNC2LI1, PLEKHH2, CAMKMT, LOC106781689*, LOC102147346*, LOC106781702*
				SS2_REG06	15	72126768 – 73121731		KLHL29, LOC106781710*, LOC106781709*
				SS2_REG07	15	75795552 – 76782973		RDH14, LOC100056590
				SS1_REG16	16	16542440 – 17474810		PDZRN3, PPP4R2, GXYLT2, LOC106781787*, LOC106781788*
				SS1_REG17	16	17542693 – 18503480		SHQ1, RYBP, GXYLT2, LOC102148566*, LOC102148599*, MIR9170, LOC106781771*, PROK2
				SS1_REG18	16	77697272 – 78681644		LOC102148929*, LOC106781810*, LOC106781811*, C16H3orf58(DIPK2A)
				SS2_REG08	20	59429342 – 60422986		LOC102151117*
				SS1_REG19	22	10868934 – 11861413		TASP1, ISM1, SPTLC3, LOC106782388*
				SS2_REG09	22	10600456 – 11589137		TASP1, ISM1, SPTLC3, LOC106782388*, LOC102149908*

Pereira et al. (2018)	Maximum speed index	Quarter Horse (racing)	33 SNPS windows of 1 Mb	SS2_REG10	22	14927558 – 15876595	within[a]	PLCB1, TMX4, HAO1
				SS1_REG20	25	17757118 – 18713444		ZFP37, SLC31A2, FKBP15, SLC31A1, CDC26, PRPF4, RNF183, WDR31, BSPRY, HDHD3, ALAD, RGS3, POLE3, LOC100056127, LOC106782558*, LOC100034197
				SS1_REG21	25	18768518 – 19722839		ZNF618, AMBP, KIF12, COL27A1, AKNA, ATP6V1G1, TNFSF15, TNFSF8, TNC, LOC106782561*, LOC102149355*, LOC106782560(TEX48), LOC100051420, LOC100050034, LOC100051562, LOC106782559*, LOC100050100
				SS2_REG11	25	18073334 – 19043039		SLC31A2, FKBP15, SLC31A1, CDC26, PRPF4, RNF183, WDR31, BSPRY, HDHD3, AMBP, LOC106782559*, C25H9orf43, POLE3 ALAD, RGS3, ZNF618, AMBP, KIF12, COL27A1
				SS2_REG12	30	138095 – 1097585		HNRNPU, DESI2, ADSS2, ZBTB18, C30H1orf100, C30H1orf101(CATSPERE), LOC102148849*, LOC100630213(COX20)
McGivney et al. (2019)	racecourse starts	Thoroughbred	14	BIEC2-1004	7	62413693	500 kb up and downstream of the flanking SNPs	CCDC90B, ANKRD42, PCF11, RAB30, PRCP, DDIAS
				BIEC2-1051	7	41738138		SPATA9, IGSF9B, LOC102150790*
				BIEC2-9966	7	41367656		OPCML
				BIEC2-9966	7	40454393		NTM, OPCML
				BIEC2-9966	7	41489510		OPCML
				BIEC2-9966	7	41537197		OPCML, SPATA9
				BIEC2-9966	7	40710230		NTM, OPCML
				BIEC2-9966	7	40713658		NTM, OPCML
				BIEC2-9966	7	41492057		OPCML

McGivney et al. (2019)	racecourse starts	Thoroughbred	14	BIEC2-9966	7	41363562	500 kb up and downstream of the flanking SNPs	OPCML
				BIEC2-9966	7	41363409		OPCML
				BIEC2-9966	7	40217482		NTM, OPCML
				BIEC2-9967	7	41818480		SPATA9, IGSF9B, LOC102150790*, JAM3, NCAPD3
				BIEC2-9967	7	41799089		SPATA9, IGSF9B, LOC102150790*, JAM3, NCAPD3
Gmel et al. (2019)	joint angles (poll, elbow, carpal, fetlock (front and hind), hip, stifle, and hock)	Franches-Montagnes and Lipizzan	14	AX-103779310	1	124405158	200 kb up and downstream of the significant or suggestive QTL	CORO2B, ITGA11, LOC106782204*, FEM1B
				AX-105008533	3	106128177		QDPR, CLRN2, LAP3, MED28, FAM184B
				AX-104691515	4	37412203		BET1, TFPI2, GNG11, GNGT1
				AX-103944161	7	42659817		LOC100072895, LOC100072904, LOC100072910(GLB1L2), LOC106783347*, LOC102151035*, B3GAT1
				AX-102974274	8	19266146		OAS2, RASAL1, CFAP73, DDX54, DTX1, IQCD, RITA1, TPCN1, LOC106783487*, SLC8B1, PLBD2, SDS, SDSL, LHX5
				AX-103391767	20	49183107		PKHD1, LOC106782309*
				AX-103234126	21	30166547		RAI14, TTC23L, BRIX1, RAD1, AGXT2, DNAJC21, LOC106782348*, PRLR
				AX-104124989	21	30263265		-
				AX-104559840	23	49183107		TMEM215, NDUFB6, TOPORS, LOC102149554*, ACO1, DDX58

Gmel et al. (2019)	joint angles (poll, elbow, carpal, fetlock (front and hind), hip, stifle, and hock)	Franches-Montagnes and Lipizzan	14	AX-103624054	27	22021462	200 kb up and downstream of the significant or suggestive QTL	-
				AX-103396551	28	12101898		-
				AX-103978374	28	12102163		LRRIQ1, ALX1
				AX-104440677	28	12106363		-
				AX-103649839	29	18799958		RSU1, C1QL3, PTER
Bussiman et al. (2020)	gait type	Mangalarga Marchador	22	-	4	95530303	250 kb genomic window (125 kb up and downstream to each SNP)	LOC102148563**, LOC106783092**, LOC106783093*, LOC106783105**, LOC106783094*, LOC106783095*, LOC106783096**, LOC100055383**, LOC102150403*
				-	4	95541786		LOC106783092**, LOC106783093*, LOC106783105**, LOC106783094*, LOC106783095*, LOC106783096**, LOC100055383**, LOC102150403*, LOC102150354**
				-				
				-				
				-				
				-				
				-	4	95644265		LOC106783094*, LOC106783095*, LOC106783096**, LOC100055383**, LOC102150403*, LOC102150354**, LOC100629784*, LOC106783106*, LOC102150258**, LOC102150225*, LOC100629906*
				-	4	95704189		LOC106783095*, LOC106783096**, LOC100055383**, LOC102150403*, LOC102150354**, LOC100629784*, LOC106783106*, LOC102150258**, LOC102150225*, LOC100629906*, LOC102150165**

Bussiman et al. (2020)	gait type	Mangalarga Marchador	22	-	4	95843696	250 kb genomic window (125 kb up and downstream to each SNP	LOC100055383**, LOC102150225*, LOC100629906*, LOC102150165**, LOC100055297, LOC100629325**, EPHB6, TRPV6
				-	6	26511708		SNED1, MTERF4, PASK, PPP1R7, ANO7, LOC106783239*, HDLBP
				-	6	73076296		LOC100051018, LOC100057893, LOC100057392, LOC100057471, LOC100057512, LOC100057630, LOC100146966
				-	16	29380920		
				-	23	13420227		PSAT1, CEP78, LOC100063011, LOC102148975*
				-	23	14986689		PRUNE2, GCNT1, RFK
				-	23	16892410		-
				-	23	16892719		-
				-	23	17961772		TRPM6, LOC100146487*
				-	23	17982265		-
				-	23	17985802		-
				-	23	23143405		DMRT2
				-	23	23443921		-
				-	23	23494785		-
				-	23	23597542		
					26	18598541		
					29	17178229		NSUN6, CACNB2
					29	20239175		SUV39H2, HSPA14, LOC106782741*, CDNF, FAM107B

*ncRNA gene type; **pseudo-gene; ***Positions given from EquCab 2.0 according to the cited articles, [a]since the article identified significant windows, candidate genes were identified within each window.
