# Supplementary material for: Identification and Functional Annotation of Genes Related to Horses’ Performance: From GWAS to Post-GWAS
Source: Animals (Basel). 2020 Jul 10;10(7):1173. doi: 10.3390/ani10071173 (PMC7401650; doi:10.3390/ani10071173)
Supplement: Supplementary file 1 [file animals-10-01173-s001.zip › supplementarymaterials/Supplementary tables/Table S1.pdf]

Table S1. Number of articles identified by each judge (1 and 2)* according each combination of terms and their respective references.

**Combinations of keywords**	**Number of articles**	**Article (publication year)**
performance/GWAS/horse	18	Shröder et al. (2011b) [1]
		Tozaki et al. (2010) [2]
		Eriksson et al. (2016) [3] [2]
		Trakovická et al. (2012) [4]
		Lewczuk et al. (2018) [5]
		Ricard et al. (2017) [6]
		Manson et al. (2018) [7]
		Pereira et al. (2019) [8]
		Staiger et al. (2016) [9]
		Frischknecht et al. (2016) [10]
		Pereira et al. (2018) [11]
		Tozaki et al. (2012b) [12]
		Shin et al. (2015) [13]
		Pereira et al. (2015) [14]
		Dupuis et al. (2011) [15]
		Meira et al. (2014b) [16]
		Boyko et al. (2014) [17] [1]
		Gmel et al. (2019) (18) [1]
performance/genome-wide association/horse	21	Tozaki et al. (2012a) [19]
		Velie et al. (2018) [20]
		Meira et al. (2014a) [21]
		Shröder et al. (2011b)
		Tozaki et al. (2010)
		Trakovická et al. (2012)
		Schroder et al. (2011a) [22]
		Dupuis et al. (2012) [23]
		Lewczuk et al. (2018)
		Ricard et al. (2017)
		Lykkjen et al. (2013) [24]
		Staiger et al. (2016)
		Frischknecht et al. (2016)
		Brard and Ricard (2014) [25]
		Pereira et al. (2018)
		Tozaki et al. (2012b)
		Shin et al. (2015)
		Pereira et al. (2015)
		Meira et al. (2014b)
		Hendrickson (2013) [26] [1]

		McGivney et al. (2019) [27] [1]
exercise/GWAS/horse	7	Blott et al. (2014) [28]
		Ricard et al. (2017)
		Lykkjen et al. (2013) [2]
		Dupuis et al. (2012)
		Lewis et al. (2017) [29]
		Gim and Kim (2016) [30]
		Bussiman et al. (2020) [31] [1]
exercise/genome-wide association/horse	7	McGivney et al. (2019)
		Ricard et al. (2017)
		Fritz et al. (2012) [32]
		Lewis et al. (2017)
		Valberg et al. (2011) [33]
		Blott et al. (2014) [1]
		Bussiman et al. (2020) [1]
exercise/GWAS/Mangalarga marchador	1	Bussiman et al. (2020) [1]
exercise/genome-wide association/Mangalarga marchador	1	Bussiman et al. (2020) [1]

* Judges 1 and 2 are the researchers who performed the systematic review. (1) Maria del Pilar R. Rodriguez; (2) Thayssa O. Littiere. References with no superscript means that it was in common between judges.

Articles selected in the first step of the systematic review:

1. Schröder, W.; Klostermann, A.; Stock, K. F.; Distl, O. A genome-wide association study for quantitative trait loci of show-jumping in Hanoverian warmblood horses. *Anim. Genet*. **2011b**, 43, 392-400. [doi:10.1111/j.1365-2052.2011.02265.x]
2. Tozaki, T.; Miyake, T.; Kakoi, H.; Gawahara, H.; Sugita, S.; Hasegawa, T.; Ishida, N.; Hirota, K.; Nakano, Y. A genome-wide association study for racing performances in Thoroughbreds clarifies a candidate region near the MSTN gene. *Anim. Genet*. **2010**, 41, 28-35. [doi:10.1111/j.1365-2052.2010.02095.x]
3. Eriksson, S.; Viklund, Å.; Mikko, S. A genome-wide association study of young horse test traits in Swedish Warmblood. Proceedings of the 67th Annual Meeting of the EAAP, Belfast, Ireland, 2016, p.576.
4. Trakovická, A.; Gábor, M.; Miluchová, M.; Minarovič, T.; Šťastná, D. Analysis of the Nebulin-Related Anchoring protein gene (NRAP) SNP polymorphism (C/T) in Slovak Warmblood horse by PCR-RFLP method. *Anim. Sci. Biotech*. **2012**, 45, 265-268.
5. Lewczuk, D.; Bereznowski, A.; Hecold, M.; Frąszczak, M.; Ruść, A.; Korwin-Kossawska, A.; Szyda, J. Differences between horse selection based on two forms of osteochondrosis in fetlock. *J. Appl. Genet*. **2018**, 59, 225-230. [doi: 10.1007/s13353-018-0437-6]
6. Ricard, A.; Robert, C.; Blouin, C.; Baste, F.; Torquet, G.; Morgenthaler, C.; Rivière, J.; Mach, N.; Mata, X.; Schibler, L.; Barrey, E. Endurance exercise ability in the horse: A trait with complex polygenic determinism. *Front. Genet*. **2017**, 8, 1-15. [doi:10.3389/fgene.2017.00089]
7. Mason, V. C.; Schaefer, R. J.; McCue, M. E.; Leeb, T.; Gerber, V. eQTL discovery and their association with severe equine asthma in European Warmblood horses. *BMC Genomics* **2018**, 19, 1-15. [doi: 10.1186/s12864-018-4938-9]
8. Pereira, G. L.; Malheiros, J. M.; Ospina, A. M. T.; Chardulo, L. A. L.; Curi, R. A. Exome sequencing in genomic regions related to racing performance of Quarter Horses. *J. Appl. Genet*. **2019**, 60, 79-86. [doi: 10.1007/s13353-019-00483-1]
9. Staiger, E. A.; Albright, J. D.; Brooks, S. A. Genome-wide association mapping of heritable temperament variation in the Tennessee Walking Horse. *Genes Brain Behav*. **2016**, 15, 514-526. [doi: 10.1111/gbb.12290]
10. Frischknecht, M.; Signer-Hasler, H.; Leeb, T.; Rieder, S.; Neuditschko, M. Genome-wide association studies based on sequence-derived genotypes reveal new QTL associated with conformation and performance traits in the Franches-Montagnes horse breed. *Anim. Genet***. 2016**, 47, 227-229. [doi: 10.1111/age.12406]

11. Pereira, G.L.; Chardulo, L.A.; Silva, J.A.IIV.; Faria, R.; Curi, R.A. Genomic regions associated with performance in racing line of Quarter Horses. *Livest. Sci.* **2018**, 211, 42-51. [https://doi.org/10.1016/j.livsci.2018.02.015]
12. Tozaki, T.; Miyake, T.; Kakoi, H.; Gawahara, H.; Hirota, K.; Nakano, Y.; Kurosawa, M. Heritability estimates for racing performance in Japanese Thoroughbred recehorses using linear and non-linear model analyses. *J. Anim. Breed. Genet.* **2012b**, 129, 402-408. [doi:10.1111/j.1439-0388.2011.00982.x]
13. Shin, D-H.; Lee, J.W.; Park, J-E.; Choi, I-Y.; Oh, H-S.; Kim, H.J.; Kim, H. Multiple genes related to muscle identified through a joint analysis of two-stage genome-wide association study for racing performance of 1,156 Thoroughbreds. *Asian Australas. J. Anim. Sci.* **2015**, 28, 771-781. [doi:10.5713/ajas. 14.008]
14. Pereira, G. L.; Regatieri, I. C.; Ferraz, G. C.; Queiroz Neto, A.; Curi, R. A. Perspectivas do uso de marcadores moleculares no melhoramento genético de equinos de corrida da raça Quarto de Milha. *Vet. e Zootec.* **2015**, 22, 347-369.
15. Dupuis, M-C.; Zhang, Z.; Druet, T.; Denoix, J-M.; Charlier, C.; Lekeux, P.; Georges, M. Results of haplotype-based GWAS for recurrent laryngeal neuropathy in the horse. *Mamm. Genome* **2011**, 22, 613-620. [doi:10.1007/s00335-011-9337-3]
16. Meira, C.T.; Fortes, M.M.; Farah, M.R.S.; Porto-Neto, L.R.; Kelly, M.; Moore, S.S.; Pereira, G.L.; Chardulo, L.A.L.; Curi, R.A. Speed index in the racing Quarter Horse: A genome-wide association study. *J Equine Vet Sci.* **2014b**, 34, 1263-1268. [http://dx.doi.org/10.1016/j.jevs.2014.09.003]
17. Boyko, A. R.; Brooks, S. A.; Behan-Braman, A.; Castelhano, M.; Corey, E.; Oliveira, K. C.; Swinburne, J. E.; Todhunter, R. J.; Zhang, Z.; Ainsworth, D. M.; Robinson, N. E. Genomic analysis establishes correlation between growth and laryngeal neuropathy in Thoroughbreds. *BMC Genomics* **2014**, 15, 1-9. [doi: 10.1186/1471-2164-15-259]
18. Gmel, A.I.; Druml, T.; Niederhäusern, R.; Leeb, T.; Neuditschko, M. Genome-wide association studies based on equine joint angle measurements reveal new QTL affecting the conformation of horses. *Genes* **2019**, 10, 1-17. [doi:10.3390/genes10050370]
19. Tozaki, T.; Hill, E. W.; Hirota, K.; Kakoi, H.; Gawahara, H.; Miyake, T.; Sugita, S.; Hasegawa, T.; Ishida, N.; Nakano, Y.; Kurosawa, M. A cohort study of racing performance in Japanese Thoroughbred recehorses using genome information on ECA18. *Anim. Genet.* **2012a,** 43, 42-52. [doi:10.1111/j.1365-2052.2011.02201.x]
20. Velie, B.D.; Fegraeus, K.J.; Solé, M.; Rosengren, M.K.; Røed, K.H.; Ihler, C-F.; Strand, E.; Lindgren, G. A genome-wide association study for harness racing success in the Norwegian-Swedish coldblooded trotter reveals genes for learning and energy metabolism. *BMC Genetics* **2018**, 19, 1-13. [https://doi.org/10.1186/s12863-018-0670-3]
21. Meira, C.T.; Farah, M.M.; Fortes, M.R.S.; Moore, S.S.; Pereira, G.L.; Silva, J.A.IIV.; Mota, M.D.S.; Curi, R.A. A genome-wide association study for morphometric traits in Quarter Horse. *J Equine Vet Sci.* **2014a**, 34, 1028-1031. [https://doi.org/10.1016/j.jevs.2014.05.11]
22. Schröder, W.; Klostermann, A.; Distl, O. Candidate genes for physical performance in the horse. *Vet. J.* **2011**a, 190, 39-48. [doi:10.1016/j.tvjl.2010.09.029]
23. Dupuis, M. C.; Zhang, Z.; Durkin, K.; Charlier, C.; Lekeux, P.; Georges, M. Detection of copy number variants in the horse genome and examination of their association with recurrent laryngeal neuropathy. *Anim. Genet.* **2012**, 44, 206-208. [doi: 10.1111/j.1365-2052.2012.02373.x]
24. Lykkjen, S.; Dolvik, N. I.; McCue, M. E.; Rendahl, A. K.; Mickelson, J. R.. Røed, K. H. Equine developmental orthopedic diseases – a genome-wide association study of first phalanx plantar osteochondral fragments in Standardbred trotters. *Anim. Genet.* **2013**, 44, 766-769. [doi: 10.1111/age.12064]
25. Brard, S.; Ricard, A. Genome-wide association study for jumping performances in French sport horses. *Anim. Genet.* **2014**, 46, 78-81. [doi: 10.1111/age.12245]
26. Hendrickson, S. L. A genome wide study of genetic adaptation to high altitude in feral Andean horses of the páramo. *BMC Evol. Biol.* **2013**, 13, 1-12. [doi: 10.1186/1471-2148-13-273]
27. McGivney, B.A.; Hernandez, B.; Katz, L.M.; MacHugh, D.E.; McGovern, S.P.; Parnell, A.C.; Wiencko, H.L.; Hill, E.W. A genomic prediction model for racecourse starts in the Thoroughbred horse. *Anim. Genet.* **2019**, 50, 347-357. [doi: 10.1111/age.12798]

28. Blott, S. C.; Swinburne, J. E.; Sibbons, C.; Fox-Clipsham, L. Y.; Helwegen, M.; Hillyer, L.; Parkin, T. D. H.; Newton, J. R.; Vaudin, M. A genome-wide association study demonstrates significant genetic variation for fracture risk in Thoroughbred racehorses. *BMC Genomics* **2014**, 15, 1-8. [doi: 10.1186/1471-2164-15-147]
29. Lewis, S. L.; Holl, H. M.; Streeter, C.; Posvergh, C.; Schanbacher, B. J.; Place, N. J.; Mallicote, M. F.; Long, M. T.; Brooks, S. A. Genomewide association study reveals a risk locus for equine metabolic syndrome in the Arabian horse. *J. Anim. Sci.* **2017**, 95, 1071-1079. [doi:10.2527/jas2016.1221]
30. Gim, J-A.; Kim, H-S. Recent strategy for superior horses. *Life Sci.* **2016**, 26, 855-867. [doi: 10.5352/JLS.2016.26.7.855]
31. Bussiman, F.O.; Santos, B.A.; Silva, B.C.A.; Mamani, G.C.M.; Grigoletto, L.; Pereira, G.L.; Ferraz, G.C.; Ferraz, J.B.S.; Mattos, E.C.; Eler, J.P.; Ventura, R.V.; Curi, R.A.; Balieiro, J.C.C. Genome-wide association study: Understanding the genetic basis of the gait type in Brazilian Mangalarga Marchador horses, a preliminary study. *Livest. Sci.* **2020**, 231, 1-11. [https://doi.org/10.1016/j.livsci.2019.103867]
32. Fritz, K. L.; McCue, M. E.; Valberg, S. J.; Rendahl, A. K.; Mickelson, J. R. Genetic mapping of recurrent exertional rhabdomyolysis in a population of North American Thoroughbreds. *Anim. Genet.* **2012**, 43, 730-738. [doi: 10.1111/j.1365-2052.2012.02351.x]
33. Valberg, S. J.; McCue, M. E.; Mickelson, J. R. The interplay of genetics, exercise, and nutrition in polysaccharide storage myopathy. *J. Equine Vet. Sci.* **2011**, 31, 205-210. [doi:10.1016/j.jevs.2011.03.001]
