# Supplementary material for: Identification and Functional Annotation of Genes Related to Horses’ Performance: From GWAS to Post-GWAS
Source: Animals (Basel). 2020 Jul 10;10(7):1173. doi: 10.3390/ani10071173 (PMC7401650; doi:10.3390/ani10071173)
Supplement: Supplementary file 1 [file animals-10-01173-s001.zip › supplementarymaterials/TFM_explorer_out/tfmexplorer_group8.pdf]

```
;TFM Explorer
;Date               : Wed May 13 06:38:01 2020
;Scanned location   : -3000:300
;Scanned sequences  : None (46 sequences)
;Scanned matrices   : (130 matrices)
;Parameters         : minsize= 30, maxsize= 1500, ratio=3.0, top=25
;
; headers
;    1    rank              window rank
;    2    matrix            matrix name
;    3    tf                transcription factor name
;    4    info              information of content of the matrix
;    5    gc                gc content of the matrix (G+C)%
;    6    location          location of the window (relatively to TSS)
;    7    pvalue            pvalue of the window

1   MA0080.2        SPI1    9.64024729829   0.435374151649  -1423:-1154
3.56627272327e-08
                list of hits
                LRRIQ1  -1387   -1380   -       6.42    ACTTTCT
                CORO2B  -1275   -1268   +       6.81    AGGAAGC
                CORO2B  -1264   -1257   -       6.28    ACTTCCG
                ITGA11  -1311   -1304   -       6.78    ATTTCCT
                FEM1B   -1211   -1204   -       8.65    ACTTCCT
                RSU1    -1282   -1275   +       6.31    AGGAAGA
                C1QL3   -1312   -1305   +       6.81    AGGAAGC
                C1QL3   -1273   -1266   -       6.81    GCTTCCT
                PTER    -1309   -1302   -       6.31    TCTTCCT
                PTER    -1179   -1172   -       6.78    ATTTCCT
                BET1    -1322   -1315   +       6.42    AGAAAGT
                TFPI2   -1415   -1408   +       6.31    AGGAAGA
                GNG11   -1275   -1268   -       6.45    ACTTCTT
                GNGT1   -1392   -1385   +       8.65    AGGAAGT
                GNGT1   -1326   -1319   +       6.31    AGGAAGA
                CLRN2   -1192   -1185   -       6.28    ACTTCCG
                LAP3    -1372   -1365   +       8.65    AGGAAGT
                MED28   -1292   -1285   -       6.28    AGTTCCT
                MED28   -1242   -1235   +       6.42    AGAAAGT
                FAM184B -1350   -1343   +       6.31    AGGAAGA
                FAM184B -1316   -1309   -       6.31    TCTTCCT
                GLB1L2  -1304   -1297   -       8.65    ACTTCCT
                B3GAT1  -1295   -1288   -       8.65    ACTTCCT
                TMEM215 -1361   -1354   -       6.96    ACTTCCC
                TMEM215 -1198   -1191   -       6.31    TCTTCCT
                NDUFB6  -1326   -1319   -       6.31    TCTTCCT
                TOPORS  -1273   -1266   -       6.96    ACTTCCC
                ACO1    -1323   -1316   -       8.65    ACTTCCT
                ACO1    -1223   -1216   +       6.42    AGAAAGT
                DDX58   -1394   -1387   +       8.65    AGGAAGT
                DDX58   -1216   -1209   +       6.31    AGGAAGA
                DDX58   -1164   -1157   +       6.31    AGGAAGA
                PKHD1   -1202   -1195   +       6.81    AGGAAGC
                RASAL1  -1399   -1392   +       6.96    GGGAAGT
                RASAL1  -1391   -1384   -       8.65    ACTTCCT
                DDX54   -1158   -1151   +       6.31    AGGAAGA
                PLBD2   -1419   -1412   +       6.31    AGGAAGA
                PLBD2   -1385   -1378   -       6.31    TCTTCCT
                SDSL    -1421   -1414   -       6.31    TCTTCCT
                TTC23L  -1423   -1416   +       6.81    AGGAAGC
                TTC23L  -1389   -1382   -       6.31    TCTTCCT
                TTC23L  -1236   -1229   -       6.45    ACTTCTT
                BRIX1   -1375   -1368   -       6.96    ACTTCCC
                RAD1    -1384   -1377   +       6.78    AGGAAAT
                RAD1    -1252   -1245   -       6.31    TCTTCCT
                AGXT2   -1154   -1147   +       6.28    AGGAACT
                DNAJC21 -1155   -1148   -       6.31    TCTTCCT
                PRLR    -1234   -1227   +       6.81    AGGAAGC
                PRLR    -1185   -1178   -       6.31    TCTTCCT
```

RAI14	-1387	-1380	+	6.45	AAGAAGT
RAI14	-1327	-1320	+	6.31	AGGAAGA
RAI14	-1252	-1245	+	8.65	AGGAAGT

2 MA0031.1 FOXD1 11.9264176032 0.27500000177 -1819:-1739
8.25901485677e-07

list of hits

FEM1B	-1774	-1766	-	6.77	TTGTTTAA
BET1	-1787	-1779	+	6.77	ATAAACAA
MED28	-1818	-1810	+	6.85	GTAAATAT
MED28	-1786	-1778	+	5.88	GTAAATAG
MED28	-1761	-1753	+	6.77	ATAAACAA
GLB1L2	-1768	-1760	+	9.69	GTAAACAT
TMEM215	-1757	-1749	+	5.94	CTAAACAG
TOPORS	-1801	-1793	+	6.91	TTAAACAT
ACO1	-1747	-1739	+	6.66	GTCAACAA
DDX58	-1808	-1800	+	6.72	GTAAAAAA
DTX1	-1739	-1731	+	6.77	ATAAACAA
_IQCD	-1814	-1806	-	6.72	TTTTTTAC
TPCN1	-1744	-1736	+	6.85	GTAACCAT
PLBD2	-1799	-1791	-	5.94	CTGTTTAT
PLBD2	-1774	-1766	-	6.72	TTTTTTAC
SDS	-1775	-1767	+	6.66	GTCAACAA
TTC23L	-1819	-1811	-	6.91	ATGTTTAA
RAI14	-1761	-1753	+	6.85	GTAAGCAT

3 MA0259.1 HIF1A::ARNT 9.73982402355 0.657451923937 -2309:-2025
9.9483690962e-07

list of hits

ITGA11	-2114	-2106	-	7.13	TCACGTCC
QDPR	-2292	-2284	-	7.14	CCACGTAC
MED28	-2267	-2259	+	7.45	CCACGTGC
GLB1L2	-2267	-2259	-	6.64	CCACGTCT
TMEM215	-2230	-2222	+	7.08	GCACGTGG
RASAL1	-2309	-2301	-	7.23	CCACGTCC
CFAP73	-2048	-2040	+	7.13	GGACGTGA
DDX54	-2232	-2224	-	6.95	GCACGCCC
TPCN1	-2269	-2261	+	6.95	GGACGTGT
TPCN1	-2230	-2222	+	7.41	ACACGTGC
SDS	-2025	-2017	+	7.41	ACACGTGC
TTC23L	-2245	-2237	+	7.45	CCACGTGC
TTC23L	-2164	-2156	-	7.61	GCACGTCG
AGXT2	-2128	-2120	+	6.54	AGACGTGA
AGXT2	-2087	-2079	+	6.79	GCACGTGT

4 MA0259.1 HIF1A::ARNT 9.73982402355 0.657451923937 -333:55
3.76114574543e-06

list of hits

CORO2B	-0333	-325	+	6.95	GGGCGTGC
CORO2B	+0041	49	-	6.39	GCACGCCG
FEM1B	+0036	44	-	6.79	GCACGCGC
RSU1	-0219	-211	-	6.97	TCACGTGC
RSU1	-0121	-113	+	7.13	GGACGTGA
C1QL3	+0012	20	+	7.08	GCACGTGG
BET1	+0055	63	+	6.64	AGACGTGG
TFPI2	-0282	-274	+	6.55	ATACGTGG
QDPR	-0219	-211	+	6.55	ATACGTGG
QDPR	-0124	-116	-	6.95	GCACGCCC
QDPR	+0011	19	+	6.95	GGGCGTGC
LAP3	-0321	-313	+	6.79	GCGCGTGC
LAP3	-0290	-282	-	6.86	GCACGCAC
LAP3	-0154	-146	-	6.95	GCACGCCC
LAP3	-0026	-18	+	7.45	CCACGTGC
MED28	-0123	-115	+	7.57	AGACGTGC
FAM184B	-0325	-317	-	6.59	CCACGTAG
FAM184B	-0253	-245	+	8.00	GCACGTGC
FAM184B	-0044	-36	-	6.97	TCACGTGC
DDX58	-0272	-264	-	6.86	ACACGTAC
DDX58	-0070	-62	-	6.30	GCACGCAG

```
                OAS2    -0116   -108     +       6.97    GCACGTGA
                DDX54   -0269   -261     +       7.61    CGACGTGC
                DDX54   +0040   48       -       6.86    GCACGCAC
                DTX1    -0074   -66      -       6.38    TCACGTGT
                _IQCD   -0201   -193     -       7.13    TCACGTCC
                RITA1   -0281   -273     +       6.86    GTGCGTGC
                RITA1   +0028   36       -       7.61    GCACGTCG
                TPCN1   -0057   -49      +       7.13    GGACGTGA
                SLC8B1  -0201   -193     -       6.45    TCACGTAT
                SDSL    -0128   -120     +       8.00    GCACGTGC
                LHX5    -0061   -53      +       7.51    CTACGTGC
                BRIX1   -0294   -286     -       6.40    GCACGTTG
                RAD1    -0321   -313     +       6.79    GCGCGTGC
                RAI14   -0075   -67      +       6.52    CCACGTGG

    5   MA0087.1        Sox5    10.8307137621   0.198757763952  -1426:-1190
 5.79285645001e-06
                list of hits
                LRRIQ1  -1282   -1275    +       5.34    AAGCAAT
                LRRIQ1  -1212   -1205    -       5.34    ATTGCTT
                LRRIQ1  -1190   -1183    -       5.34    ATTATTT
                FEM1B   -1326   -1319    -       5.39    ATTGTAT
                FEM1B   -1259   -1252    +       5.34    AAACACT
                C1QL3   -1385   -1378    -       5.34    AGTGTTA
                C1QL3   -1194   -1187    +       5.39    ATACAAT
                PTER    -1411   -1404    +       5.34    TAGCAAT
                BET1    -1305   -1298    -       5.34    ATTATTT
                BET1    -1241   -1234    -       5.39    ATTGTAA
                GNGT1   -1299   -1292    -       8.38    ATTGTTT
                QDPR    -1426   -1419    +       5.39    TTACAAT
                QDPR    -1419   -1412    -       5.34    AGTGTTA
                CLRN2   -1223   -1216    +       8.38    TAACAAT
                FAM184B -1283   -1276    -       5.34    ATTATTA
                TMEM215 -1324   -1317    +       5.39    TGACAAT
                NDUFB6  -1413   -1406    -       8.38    ATTGTTT
                NDUFB6  -1290   -1283    +       5.39    ATACAAT
                NDUFB6  -1217   -1210    +       5.39    TGACAAT
                NDUFB6  -1190   -1183    +       7.69    CAACAAT
                TOPORS  -1327   -1320    -       5.34    ATTGCTT
                ACO1    -1309   -1302    +       5.34    AAATAAT
                DDX58   -1248   -1241    +       5.34    AAGCAAT
                OAS2    -1424   -1417    +       5.34    AAATAAT
                CFAP73  -1389   -1382    +       5.39    TGACAAT
                CFAP73  -1289   -1282    -       5.34    AGTGTTT
                _IQCD   -1420   -1413    -       5.39    ATTGTCT
                _IQCD   -1342   -1335    -       5.39    ATTGTCA
                RITA1   -1218   -1211    +       5.34    TAGCAAT
                TPCN1   -1398   -1391    -       5.39    ATTGTAT
                TPCN1   -1347   -1340    -       5.34    ATTGCTT
                SDSL    -1199   -1192    -       7.69    ATTGTTG
                LHX5    -1315   -1308    +       5.39    ATACAAT
                TTC23L  -1205   -1198    -       8.38    ATTGTTT
                BRIX1   -1282   -1275    -       5.34    ATTATTA
                BRIX1   -1249   -1242    +       5.34    AAACACT
                AGXT2   -1332   -1325    -       5.34    AGTGTTT
                AGXT2   -1250   -1243    +       7.41    GAACAAT
                DNAJC21 -1411   -1404    -       5.39    ATTGTCT
                DNAJC21 -1394   -1387    +       5.39    ATACAAT

    6   MA0067.1        Pax2    6.20567064398   0.516129044515  -1410:-1244
 9.15373698354e-06
                list of hits
                LRRIQ1  -1410   -1402    -       5.99    AAGTGACT
                ALX1    -1310   -1302    -       5.85    GCGTGATT
                CORO2B  -1316   -1308    -       5.94    ACGTGACC
                FEM1B   -1317   -1309    +       5.89    TGTCACTT
                FEM1B   -1244   -1236    +       6.26    GGTCACGG
                TFPI2   -1398   -1390    -       6.41    CCATGACA
                LAP3    -1301   -1293    -       6.20    CAGTGACA
```

```
                TMEM215 -1366   -1358   -       6.20    ACATGACT
                ACO1    -1274   -1266   -       6.41    GCATGACA
                DDX58   -1391   -1383   -       5.89    AAGTGACA
                RASAL1  -1396   -1388   -       5.99    AAGTGACT
                TPCN1   -1297   -1289   -       5.99    AAGTGACT
                SDSL    -1343   -1335   -       5.99    AAGTGACT
                SDSL    -1318   -1310   -       5.85    GCGTGATT
                SDSL    -1253   -1245   +       5.84    CGTCATGT
                TTC23L  -1334   -1326   -       6.51    CCATGACT
                DNAJC21 -1340   -1332   -       5.99    AAGTGACT
                DNAJC21 -1279   -1271   +       5.89    TGTCACTT

    7   MA0136.1        ELF5    8.69309550562   0.429292928841  -1399:-1293
2.17710570593e-05
                list of hits
                ITGA11  -1312   -1303   +       8.56    TATTTCCTT
                PTER    -1310   -1301   +       6.19    CTCTTCCTT
                GNGT1   -1393   -1384   -       7.93    AAGGAAGTG
                GNGT1   -1340   -1331   -       7.33    AAGGATATT
                GNGT1   -1327   -1318   -       6.34    CAGGAAGAA
                QDPR    -1324   -1315   +       6.82    TTTTTCCTT
                CLRN2   -1303   -1294   -       6.31    AAGGAAAAT
                LAP3    -1373   -1364   -       8.08    CAGGAAGTA
                MED28   -1293   -1284   +       7.35    TAGTTCCTT
                FAM184B -1338   -1329   -       6.64    ACGGATGTT
                GLB1L2  -1305   -1296   +       7.77    TACTTCCTC
                B3GAT1  -1397   -1388   -       6.82    AAGGAAAAA
                B3GAT1  -1296   -1287   +       7.26    AACTTCCTC
                TOPORS  -1398   -1389   +       6.43    TATTTCCCT
                ACO1    -1324   -1315   +       7.93    CACTTCCTT
                DDX58   -1395   -1386   -       6.30    TAGGAAGTG
                RASAL1  -1392   -1383   +       6.21    GACTTCCTG
                PLBD2   -1399   -1390   +       6.64    AACATCCGT
                TTC23L  -1390   -1381   +       6.48    ATCTTCCTT
                RAD1    -1385   -1376   -       6.81    GAGGAAATG
                RAD1    -1342   -1333   +       7.35    TAGTTCCTT

    8   MA0131.1        MIZF    13.1966528521   0.610000000149  -1995:-1765
3.10860638316e-05
                list of hits
                CORO2B  -1884   -1874   -       5.28    GAGGGCGTTC
                ITGA11  -1765   -1755   -       7.97    ACGGACTTTG
                RSU1    -1990   -1980   -       5.23    CCGGAAGTAA
                LOC100072895    -1945   -1935   +       5.00    GTAGGTCCGG
                TOPORS  -1784   -1774   +       4.85    TTACTTCCTC
                RASAL1  -1921   -1911   -       5.84    CAGGACGTTG
                CFAP73  -1834   -1824   +       5.99    TAACGTCCTG
                CFAP73  -1791   -1781   +       5.14    GAACGGCCGC
                SLC8B1  -1796   -1786   +       6.53    CTAGGTCCGT
                SDS     -1964   -1954   +       6.51    CAACTTCCGA
                LHX5    -1995   -1985   +       5.23    TAAATTCCGT
                RAI14   -1869   -1859   -       4.40    AAGGACCTTG
                RAI14   -1796   -1786   -       5.52    GCTGACGTTA

    9   MA0143.1        Sox2    12.9512704469   0.361474939125  -781:-704
3.70170555013e-05
                list of hits
                LRRIQ1  -0761   -746    +       5.90    TAATTCTTATGTTGA
                LRRIQ1  -0711   -696    +       6.10    CTATTTTCTTTGAAA
                TFPI2   -0714   -699    -       5.02    TCTCAAAAACAGTAG
                GNG11   -0764   -749    +       5.47    TTATTCTTCTTCTAG
                GNGT1   -0766   -751    -       7.03    GGTGCAGAAGAAAGA
                GNGT1   -0744   -729    +       7.34    CTTTTATGATGTACA
                GNGT1   -0704   -689    +       5.10    CCTTTAACCTGCTGT
                QDPR    -0750   -735    -       5.61    CTAGCAGACCAAAGA
                LAP3    -0755   -740    +       6.17    GCATTTTTTTACAAA
                LAP3    -0726   -711    -       7.05    TTTAAAAAAGAAAAA
                MED28   -0772   -757    +       7.05    TTTTTCTTTTTTAAA
                MED28   -0712   -697    +       5.17    TTTTTCTTCTGCTTT
```

```
                FAM184B -0781   -766    +       5.65    CCTTTGTTCCGTGAT
                GLB1L2  -0737   -722    +       6.62    CCATTCTTCTGTCAG
                TOPORS  -0714   -699    -       5.67    GATGCAGCAGAATGA
                OAS2    -0742   -727    +       6.29    CCTTTGTCTTCTGTA
                CFAP73  -0780   -765    -       6.10    TGTGAAAAAAAATGA
                DDX54   -0708   -693    -       5.18    TACAGATGACAAAAC
                RAD1    -0706   -691    -       6.18    ACCGCATAACAAGGG
                DNAJC21 -0722   -707    +       6.02    GCATTCTTAACCTAA

    10  MA0122.1        Nkx3-2  8.54224970754   0.462962961859  -1177:-1110
4.32905859778e-05
                list of hits
                ITGA11  -1177   -1168   +       6.88    CTGAGTGGG
                CLRN2   -1161   -1152   -       6.26    GCCACTTCT
                LAP3    -1125   -1116   +       6.22    TGAAGTGAA
                LOC100072895    -1166   -1157   -       6.11    CCCACTCCA
                LOC100072895    -1154   -1145   +       6.15    CCAAGTGCA
                TMEM215 -1144   -1135   +       6.37    CTGAGTAGA
                TMEM215 -1114   -1105   +       6.69    TGGAGTGGA
                TOPORS  -1138   -1129   +       6.84    CCAAGTGGA
                RASAL1  -1171   -1162   -       6.13    GTCACTCAG
                CFAP73  -1137   -1128   +       6.84    CCAAGTGGA
                _IQCD   -1116   -1107   +       6.26    GGAAGTGGC
                RITA1   -1110   -1101   -       6.53    GGCACTCAG
                TPCN1   -1138   -1129   -       6.53    GGCACTCAG
                PLBD2   -1168   -1159   -       6.37    TCTACTCAG

    11  MA0145.1        Tcfcp2l1        11.6502911816   0.609628129495  -1339:-1287
4.97937789201e-05
                list of hits
                C1QL3   -1298   -1284   +       5.86    CCAGTTAAGACCAT
                MED28   -1339   -1325   +       5.53    CCACTTCTGTCCTG
                LOC100072895    -1287   -1273   -       5.33    CTGCCTGGCGCAGG
                B3GAT1  -1334   -1320   -       5.87    CTGCACTGGGCAGG
                DTX1    -1317   -1303   +       6.08    CCAGAAAAATGCAG
                RITA1   -1336   -1322   +       5.48    CAAGGTTGGGCCAG
                RITA1   -1313   -1299   +       5.75    CCGGGCTGCACCAG
                RITA1   -1289   -1275   -       5.75    CTGGCCTGAGCCAC
                SLC8B1  -1287   -1273   +       5.33    CCAGTCCCATGTTG
                SDS     -1316   -1302   -       6.45    CCGGCCTCGCCTGG
                LHX5    -1298   -1284   +       5.80    CAAGTGTAAGCTAG
                RAI14   -1306   -1292   -       5.85    TTGCTTTGAACTGG

    12  MA0004.1        Arnt    10.9916749403   0.641666668778  -419:13 5.51564255093e-05
                list of hits
                RSU1    -0218   -212    +       8.00    CACGTG
                C1QL3   +0013   19      +       8.00    CACGTG
                LAP3    -0403   -397    +       8.00    CACGTG
                LAP3    -0397   -391    +       8.00    CACGTG
                LAP3    -0025   -19     +       8.00    CACGTG
                MED28   -0040   -34     +       8.00    CACGTG
                FAM184B -0380   -374    +       8.00    CACGTG
                FAM184B -0252   -246    +       8.00    CACGTG
                FAM184B -0043   -37     +       8.00    CACGTG
                NDUFB6  -0419   -413    +       8.00    CACGTG
                TOPORS  -0193   -187    +       8.00    CACGTG
                OAS2    -0115   -109    +       8.00    CACGTG
                RASAL1  -0379   -373    +       8.00    CACGTG
                DTX1    -0073   -67     +       8.00    CACGTG
                PLBD2   +0003   9       +       8.00    CACGTG
                SDSL    -0127   -121    +       8.00    CACGTG
                RAI14   -0074   -68     +       8.00    CACGTG

    13  MA0156.1        FEV     12.1208832256   0.442307695746  -2003:-1856
7.05488822314e-05
                list of hits
                LRRIQ1  -1875   -1867   +       5.79    AGGGAAGT
                LRRIQ1  -1856   -1848   +       5.26    TGGGAAAT
                ALX1    -2003   -1995   +       7.28    TAGGAAGT
```

```
                FEM1B   -1996   -1988   +       8.11    AAGGAAAT
                RSU1    -1990   -1982   +       8.36    CCGGAAGT
                RSU1    -1950   -1942   -       5.94    ATTTCCCT
                RSU1    -1884   -1876   +       8.11    AAGGAAAT
                QDPR    -1991   -1983   +       6.99    CAGGATGT
                LAP3    -1891   -1883   +       5.26    GGGGAAAT
                TMEM215 -1878   -1870   +       8.11    AAGGAAAT
                NDUFB6  -1972   -1964   -       7.43    ATTTCCTA
                ACO1    -1957   -1949   -       7.43    ATTTCCTC
                PKHD1   -1883   -1875   +       7.96    AAGGAAGT
                OAS2    -1982   -1974   -       7.43    ATTTCCTC
                DTX1    -1883   -1875   -       7.28    ACTTCCTC
                TPCN1   -1955   -1947   +       5.26    GGGGAAAT
                PLBD2   -1898   -1890   -       5.26    ATTTCCCC
                SDS     -1962   -1954   -       6.19    ACTTCCGA
                TTC23L  -1869   -1861   -       5.79    ACTTCCCT
                AGXT2   -1861   -1853   -       5.11    ACTTCCCA

    14  MA0002.2        RUNX1   10.4014248352   0.501590918453  -877:-805
 7.14859891226e-05
                list of hits
                RSU1    -0842   -831    -       6.26    AAAGCACAGAT
                C1QL3   -0877   -866    -       5.93    AACACACAGAA
                BET1    -0875   -864    -       7.76    AAACCGCAAAC
                TFPI2   -0814   -803    -       6.51    AAAACACAAAT
                B3GAT1  -0805   -794    +       6.94    TCCTGTGGCTC
                DDX58   -0862   -851    -       6.75    ACACCACAACA
                DDX58   -0820   -809    -       5.68    AACCCAAAGAC
                DDX58   -0805   -794    -       6.09    AAATCACAGAA
                PKHD1   -0822   -811    -       6.00    AAAACACAAGT
                OAS2    -0835   -824    -       6.21    TGACCACACAT
                CFAP73  -0852   -841    +       6.39    TTCTGTGTCTT
                DTX1    -0867   -856    -       7.36    CACCCACAGGC
                SDSL    -0877   -866    +       6.83    GTCTGTGTTCT
                SDSL    -0835   -824    -       8.22    AAACCACAATT
                PRLR    -0875   -864    -       7.55    AAACCTCAGAG
                PRLR    -0807   -796    +       6.86    GCCAGTGGTTT

    15  MA0036.1        GATA2   5.68777361321   0.403773584962  -1387:-1332
 7.57797974028e-05
                list of hits
                CORO2B  -1356   -1351   -       4.95    TATCC
                TFPI2   -1387   -1382   +       4.95    GGATA
                TFPI2   -1346   -1341   -       4.95    TATCC
                GNGT1   -1338   -1333   +       4.95    GGATA
                LAP3    -1381   -1376   +       4.95    GGATA
                LAP3    -1361   -1356   -       4.95    TATCC
                TOPORS  -1336   -1331   -       4.95    TATCC
                DDX58   -1345   -1340   +       4.95    GGATA
                CFAP73  -1367   -1362   +       4.95    GGATA
                TPCN1   -1374   -1369   -       4.95    TATCC
                PRLR    -1332   -1327   +       4.95    GGATA
                RAI14   -1338   -1333   +       4.95    GGATA

    16  MA0058.1        MAX     12.6854143903   0.57058823479   -1942:-1902
 9.02629412533e-05
                list of hits
                RSU1    -1916   -1906   -       4.50    ACATGTGGGT
                PTER    -1904   -1894   +       6.90    ACACACGTGC
                TOPORS  -1910   -1900   -       7.36    CCATGTGGTT
                OAS2    -1927   -1917   +       6.42    GGACACATGA
                OAS2    -1905   -1895   -       5.52    ACATGTGACC
                RASAL1  -1942   -1932   +       5.60    CACCCCGTGG
                BRIX1   -1902   -1892   +       4.81    GACCCCATGA
                PRLR    -1941   -1931   +       4.34    AACCTCGTGA

    17  MA0091.1        TAL1::TCF3      14.0701377852   0.452651524916  -1461:-1392
 9.72366660183e-05
                list of hits
```

PTER	-1458	-1446	+	7.96	GATCCATCTGTT
GNGT1	-1425	-1413	-	5.28	AATAGATGTTTG
CLRN2	-1415	-1403	+	5.02	CAAGCATATGGT
NDUFB6	-1442	-1430	-	5.71	TAAAGATGTTCC
DDX58	-1414	-1402	+	4.62	TAGACATCTTTT
DTX1	-1402	-1390	-	4.46	AACATAAGGTTG
PLBD2	-1453	-1441	+	8.75	CTAACATCTGCT
SDS	-1451	-1439	+	5.29	GGGCCAGCTGTG
BRIX1	-1409	-1397	-	6.29	AACAAATGTTCT
DNAJC21	-1461	-1449	+	9.23	AGTCCATCTGTT
DNAJC21	-1448	-1436	-	7.83	AAAAGATGTTCC
PRLR	-1392	-1380	+	4.97	ACACCATCTTGG
