# Supplementary material for: Identification and Functional Annotation of Genes Related to Horses’ Performance: From GWAS to Post-GWAS
Source: Animals (Basel). 2020 Jul 10;10(7):1173. doi: 10.3390/ani10071173 (PMC7401650; doi:10.3390/ani10071173)
Supplement: Supplementary file 1 [file animals-10-01173-s001.zip › supplementarymaterials/TFM_explorer_out/tfmexplorer_group9.pdf]

```
;TFM Explorer
;Date              : Wed May 13 06:37:55 2020
;Scanned location  : -2129:0
;Scanned sequences : None (31 sequences)
;Scanned matrices  : (130 matrices)
;Parameters        : minsize= 30, maxsize= 1500, ratio=3.0, top=25
;
; headers
;    1    rank              window rank
;    2    matrix            matrix name
;    3    tf                transcription factor name
;    4    info              information of content of the matrix
;    5    gc                gc content of the matrix (G+C)%
;    6    location          location of the window (relatively to TSS)
;    7    pvalue            pvalue of the window

    1   MA0442.1        SOX10   7.96992529398   0.386363640428  -1548:-1456
1.14805732134e-05
                list of hits
                SUV39H2 -1542   -1536   -       6.89    ACAAAG
                HSPA14  -1531   -1525   +       6.67    CATTGT
                CDNF    -1456   -1450   -       6.89    ACAAAG
                FAM107B -1548   -1542   +       6.67    CATTGT
                FAM107B -1526   -1520   -       6.67    ACAATG
                NSUN6   -1546   -1540   +       6.67    CATTGT
                NSUN6   -1460   -1454   +       6.89    CTTTGT
                DMRT2   -1460   -1454   +       6.89    CTTTGT
                GCNT1   -1501   -1495   -       6.89    ACAAAG
                CEP78   -1548   -1542   +       6.89    CTTTGT
                LOC100146966    -1527   -1521   +       6.89    CTTTGT
                MTERF4  -1491   -1485   -       6.89    ACAAAG
                TRPV6   -1488   -1482   +       6.89    CTTTGT
                LOC100054240    -1505   -1499   -       6.89    ACAAAG
                LOC100054240    -1463   -1457   -       6.89    ACAAAG

    2   MA0003.1        TFAP2A  7.8120920509    0.800000024164  -1341:-1078
1.8763783038e-05
                list of hits
                CDNF    -1341   -1332   -       6.52    CCTCCCGGC
                CDNF    -1305   -1296   -       6.52    CCTCCCGGC
                CDNF    -1269   -1260   -       6.52    CCTCCCGGC
                CDNF    -1233   -1224   -       6.52    CCTCCAGGC
                CDNF    -1197   -1188   -       6.52    CCTCCCGGC
                CDNF    -1161   -1152   -       6.52    CCTCCCGGC
                CDNF    -1125   -1116   -       6.52    CCTCCCGGC
                CDNF    -1089   -1080   -       6.52    CCTCCCGGC
                GCNT1   -1156   -1147   +       6.70    GCCCTGAGC
                GCNT1   -1107   -1098   +       6.70    GCCCTGAGC
                PSAT1   -1130   -1121   +       6.14    GCCCTGCGC
                PSAT1   -1101   -1092   -       7.49    CCCCCGGGC
                CEP78   -1279   -1270   -       6.61    GCCTAAGGC
                LOC100057893    -1295   -1286   +       6.32    GCCAGGGGC
                HDLBP   -1336   -1327   +       6.19    GCCCGACGG
                HDLBP   -1271   -1262   +       7.28    GCCCGAGGC
                ANO7    -1141   -1132   +       6.09    GCCTGCAGC
                ANO7    -1084   -1075   +       7.25    GCCCTGGGG
                PASK    -1311   -1302   -       6.39    CCGCCGGGC
                PASK    -1290   -1281   +       6.32    GCCCCGCGG
                PASK    -1187   -1178   +       6.78    GCCTCAGGC
                SNED1   -1290   -1281   -       7.06    CCCCCAGGC
                SNED1   -1233   -1224   +       6.31    GCCGGAAGC
                SNED1   -1182   -1173   -       6.12    GCCTCTGGC
                LOC100055297    -1078   -1069   +       6.32    GCCCAGGGC

    3   MA0060.1        NFYA    12.9251246242   0.522629311625  -359:-302
2.27249942999e-05
                list of hits
                HSPA14  -0355   -339    -       10.70   ACGCTGATTGGTGGCG
```

```
                CDNF    -0359   -343    +       6.92    GAGAGCCAATAGAAAC
                PSAT1   -0354   -338    +       11.57   GCGAACCAATCAGCGC
                PSAT1   -0323   -307    +       8.87    TCGGGCCAATGGGGAC
                LOC100051018    -0345   -329    -       5.22    TCTCCCATAGGTCTGG
                ANO7    -0330   -314    +       5.25    CTAAGCAAATAAGAGG
                ANO7    -0302   -286    -       4.92    CGTGTCATTTGCTGGG
                PASK    -0356   -340    -       6.90    GGCACCATTGGTTGGC
                PASK    -0310   -294    -       9.57    TCTGCGATTGGTCAGT
                TRPV6   -0339   -323    -       9.33    GTGCTTATTGGCCATT

    4   MA0074.1        RXRA::VDR       20.4511670022   0.526666664581  -2001:-1940
2.72591395021e-05
                list of hits
                CDNF    -1961   -1946   -       1.48    GTAACTCGGTCACCT
                GCNT1   -1996   -1981   -       1.37    GGAGCTCATTGAGCT
                LOC100146966    -2001   -1986   -       0.59    TGGACTTTTTGCACC
                HDLBP   -1940   -1925   +       2.07    GGGTGACGAGATTCA
                ANO7    -1952   -1937   +       3.59    AGGGCAAGGAGTATG
                PPP1R7  -1956   -1941   -       0.59    TGAACTTCTGGAAGC
                MTERF4  -1989   -1974   -       1.28    TAATCTTATCAACCT
                TRPV6   -1980   -1965   -       0.32    TGTTACTGTTGACCT
                EPHB6   -1950   -1935   -       3.45    TGAATTCTGTGAGCC

    5   MA0076.1        ELK4    14.1232301212   0.583333336645  -293:-245
5.4861266417e-05
                list of hits
                SUV39H2 -0284   -275    -       4.71    GCCTCCGGT
                NSUN6   -0293   -284    +       9.19    GCCGGAAGT
                NSUN6   -0277   -268    +       8.42    GCCGGAAGC
                DMRT2   -0278   -269    -       5.25    ACTTCGGGT
                LOC100057471    -0248   -239    -       3.94    ATTTCCAGT
                MTERF4  -0274   -265    +       9.19    GCCGGAAGT
                MTERF4  -0245   -236    -       7.11    GTTTCCGGC
                LOC100055297    -0253   -244    -       5.25    ACTTCTGGT

    6   MA0051.1        IRF2    21.1344204686   0.412037036209  -1210:-1082
6.37114769633e-05
                list of hits
                HSPA14  -1187   -1169   -       6.24    TCTCAGGTTTTATTTTCC
                PRUNE2  -1195   -1177   +       0.03    TGTAAGAGAAAGCCTGAG
                RFK     -1205   -1187   +       -0.99   GGGAAATAAAAGTCTGGC
                RFK     -1098   -1080   +       3.43    GGAAAGTGACTGCAAAGT
                CEP78   -1140   -1122   -       2.66    CTTAGCTTTTCGATTTCC
                LOC100051018    -1210   -1192   -       0.63    ATTTAAATTTCATTTTAA
                LOC100051018    -1187   -1169   +       0.30    GAAAAATTAAAACTTACT
                LOC100051018    -1152   -1134   +       -0.91   GAATAAAGAAATTAAACT
                LOC100057392    -1161   -1143   +       1.02    AGAATATGAAACTGTAGA
                LOC100057392    -1128   -1110   -       1.67    GCAAGGTTTTCACTGTCA
                LOC100057392    -1103   -1085   +       2.67    CTAAAAGGAAACTAATTA
                LOC100057512    -1157   -1139   +       2.11    TTAAAGTTAAACTGAATA
                LOC100057512    -1093   -1075   +       -0.94   TAAAAACATAACGAAAAA
                LOC100057630    -1082   -1064   -       1.52    AATTTGGTTTCCTTAGCC
                LOC100146966    -1168   -1150   +       6.48    TGTAAGTGAAAGTAGAAG
                PPP1R7  -1142   -1124   +       1.91    GGAAAAGGAAAACCGATG
                TRPV6   -1207   -1189   +       2.65    CATAAGTGAAATCATACA

    7   MA0033.1        FOXL1   6.06853667575   0.244565224052  -2047:-1958
6.64326017366e-05
                list of hits
                SUV39H2 -2039   -2031   +       5.34    TAAGCATA
                SUV39H2 -2024   -2016   -       5.24    TATTTTAA
                HSPA14  -2043   -2035   +       5.43    GATATATA
                NSUN6   -2047   -2039   -       6.44    TATGTAAA
                NSUN6   -1965   -1957   -       6.15    TATGTTAA
                CACNB2  -2021   -2013   +       5.53    TTTAAATA
                CACNB2  -1971   -1963   +       6.15    TAGATATA
                TRPM6   -2047   -2039   -       6.51    TATGTCTA
                TRPM6   -1968   -1960   +       5.43    ACTACATA
                PRUNE2  -2030   -2022   +       5.60    TAAAAATA
```

GCNT1	-2007	-1999	+	6.44	TTTACATA
LOC100051018	-1958	-1950	+	5.43	AATAAATA
LOC100057893	-2045	-2037	+	5.35	ATGATATA
LOC100057893	-1991	-1983	+	5.88	TATAAATA
LOC100057893	-1979	-1971	+	5.60	TAAAAATA
LOC100057471	-2002	-1994	+	5.53	TTTAAATA
LOC100057471	-1989	-1981	-	5.53	TATTTAAA
LOC100057512	-1997	-1989	+	5.27	TATGTATA
LOC100057630	-1994	-1986	-	5.35	TATATTAT
PASK	-1958	-1950	+	6.06	AAAACATA
MTERF4	-2035	-2027	+	6.15	TTGACATA
MTERF4	-2020	-2012	-	5.35	TATATTAT
SNED1	-2016	-2008	+	5.47	TACATATA

8 MA0162.1 Egr1 14.4555832005 0.739393945445 -890:-674
7.68849943963e-05

list of hits

CDNF	-0820	-809	+	4.87	CCCGTAGGTGA
CDNF	-0794	-783	-	6.68	CCGCCCCCAGA
CDNF	-0729	-718	-	6.37	ACGGCCACGCA
FAM107B	-0713	-702	+	4.69	CGCGTGTGTGT
CACNB2	-0864	-853	-	3.85	CCTCCCCCATC
CACNB2	-0674	-663	-	6.58	GCGCCCACGCG
PRUNE2	-0762	-751	-	6.02	CCACCCCCACC
RFK	-0694	-683	+	5.09	TCTGGGGGAGG
PSAT1	-0881	-870	-	5.44	CCGCCTACATC
PSAT1	-0816	-805	-	3.94	CCACCTACCCA
CEP78	-0861	-850	+	7.48	TGCGTAGGAGA
CEP78	-0735	-724	+	7.76	CCCGTGGGAGG
LOC100057471	-0845	-834	-	7.10	ACACCTACACT
HDLBP	-0890	-879	+	6.64	AGCGGGGGAGA
HDLBP	-0807	-796	+	4.03	AGCGGGAGAGA
HDLBP	-0765	-754	+	4.15	AGCGGGGACGG
HDLBP	-0682	-671	-	5.62	CCTCCCCCACC
PASK	-0744	-733	-	6.99	CCGCCTCCGTT
SNED1	-0797	-786	-	7.48	CCTCCCCCGCC
SNED1	-0736	-725	-	10.43	CCGCCCCCGCG
SNED1	-0696	-685	+	7.98	CCCGGGGGCGG
EPHB6	-0839	-828	-	3.90	ACGCCCACCTT

9 MA0140.1 Tal1::Gata1 11.2966184617 0.45067769915 -264:-225
9.10647129407e-05

list of hits

LOC100051018	-0264	-246	+	5.95	CTGGGACTGTCAGATGAC
LOC100057392	-0264	-246	+	6.44	CTGGGACTGACAGATGAC
LOC100057392	-0225	-207	-	5.34	TTTATCTTTCTATTTCTT
LOC100057471	-0264	-246	+	6.36	CTGGGACTGACGGATAAT
LOC100057512	-0264	-246	+	6.44	CTGGGACTGACAGATGAC
LOC100057512	-0225	-207	-	5.77	TTTATCTTTCTATTTCTC
LOC100057630	-0264	-246	+	5.95	CTGGGACTGTCAGATGAC
