# Supplementary material for: Identification and Functional Annotation of Genes Related to Horses’ Performance: From GWAS to Post-GWAS
Source: Animals (Basel). 2020 Jul 10;10(7):1173. doi: 10.3390/ani10071173 (PMC7401650; doi:10.3390/ani10071173)
Supplement: Supplementary file 1 [file animals-10-01173-s001.zip › supplementarymaterials/TFM_explorer_out/tfmexplorer_group1.pdf]

;TFM	Explorer				
;Date	:	Wed	Jan	8	19:12:04
;Scanned	location	:	-3280:0		
;Scanned	sequences	:	None	(24	sequences)
;Scanned	matrices	:	(130	matrices)	
;Parameters	:	minsize=	30	maxsize=	1500
;					
;	headers				
;	1	rank	window	rank	
;	2	matrix	matrix	name	
;	3	tf	transcription	factor	name
;	4	info	information	of	content
;	5	gc	gc	content	of
;	6	location	location	of	the
;	7	pvalue	pvalue	of	the
1	MA0003.1	TFAP2A	78.120.920.509	0.800000024	-743:-203
list	of	hits			
EIF4G3	-695	-686	+	7.28	GCCCGAGGC
EIF4G3	-549	-540	+	6.31	GCCCCGCGC
EIF4G3	-317	-308	-	6.02	GCTGGAGGC
HP1BP3	-490	-481	+	6.47	GCCCCCAGG
HP1BP3	-409	-400	+	6.31	GCCCCGCGC
HP1BP3	-365	-356	-	7.07	CCCGCGGGC
HP1BP3	-311	-302	-	6.87	GCTCGGGGC
HP1BP3	-263	-254	+	6.52	GCCTGGAGG
HP1BP3	-215	-206	+	5.80	GCCCAGAGG
SH2D5	-649	-640	+	6.33	GCCAGGGGG
SH2D5	-481	-472	-	6.45	CCTCGCGGC
SH2D5	-349	-340	-	5.89	GCGGGGGGC
SH2D5	-312	-303	-	6.37	GCGCCGGGC
SH2D5	-301	-292	+	7.04	GCCGGGGGC
SH2D5	-286	-277	-	6.31	GCGCGGGGC
KIF17	-743	-734	-	6.47	CCTGGGGGC
KIF17	-709	-700	-	6.79	CCCTGAGGC
KIF17	-447	-438	-	7.07	CCCGCGGGC
KIF17	-423	-414	+	6.02	GCCTCCAGC
KIF17	-399	-390	-	6.99	CCCCGCGGC
KIF17	-346	-337	+	6.26	GCCACGGGG
KIF17	-284	-275	+	7.25	GCCCTGGGG
KIF17	-256	-247	+	6.09	GCCTGCAGC
KIF17	-218	-209	+	6.79	GCCTCAGGG

WWOX	-717	-708	+	6.25	GCCACGGGC
WWOX	-576	-567	+	6.56	GCCTCCGGC
WWOX	-423	-414	+	5.96	GCCGGGCGG
WWOX	-297	-288	+	5.89	GCCGAGGGC
WWOX	-264	-255	+	5.88	GCCGCGCGC
BCO1	-296	-287	+	6.51	GCCGGGAGC
A.KRD50	-528	-519	-	6.79	CCCTGCGGC
A.KRD50	-502	-493	-	6.70	GCTCAGGGC
A.KRD50	-391	-382	+	6.69	GCCCCAAGG
A.KRD50	-266	-257	+	6.25	GCCACGGGC
A.KRD50	-254	-245	+	6.64	GCCGGCGGG
A.KRD50	-228	-219	+	7.04	GCCGGGGGC
A.KRD50	-210	-201	+	7.04	GCCGGGGGC
AVPR1A	-669	-660	+	6.39	GCCCGGCGG
AVPR1A	-374	-365	+	6.82	GCCGTGGGG
AVPR1A	-211	-202	-	6.31	GCGCGGGGC
LOC1006300	-382	-373	-	6.32	CCTTCAGGC
LOC1006300	-357	-348	+	7.25	GCCCTGGGG
LOC1006300	-341	-332	+	5.99	GCCCTTGGC
STT3A	-388	-379	+	6.03	GCCTCCAGG
EI24	-309	-300	-	5.75	CCCAGCGGC
EI24	-212	-203	+	5.72	GCCGTGCGG
MCMDC2	-418	-409	+	6.94	GCCCGGAGC
MCMDC2	-366	-357	+	6.70	GCCCTGAGC
LOC1000520	-626	-617	-	6.86	CCCTCCGGC
LOC1000520	-567	-558	+	7.41	GCCCCGGGC
LOC1000520	-535	-526	+	7.41	GCCCCGGGC
LOC1000520	-362	-353	-	5.96	GCGGCGGGC
LOC1000520	-337	-328	+	5.96	GCCCGCCGC
LOC1000520	-255	-246	-	5.94	GCGCCCGGC
LOC1000520	-203	-194	+	5.96	GCCCGCCGC
ATP13A1	-724	-715	-	6.82	CCCCACGGC
ATP13A1	-445	-436	-	6.32	CCTTCCGGC
ATP13A1	-343	-334	+	6.32	GCCCAGGGC
ATP13A1	-210	-201	+	6.39	GCCCGGCGG
GMIP	-346	-337	+	6.25	GCCACGGGC
GMIP	-229	-220	-	6.57	CCCGGCGGC
LPAR2	-726	-717	+	6.32	GCCCAGGGC
LPAR2	-716	-707	+	6.05	GCCACAGGC
LPAR2	-683	-674	+	6.32	GCCAGGGGC
LPAR2	-612	-603	+	6.13	GCCAGAGGG
LPAR2	-601	-592	-	6.32	CCGCGGGGC
LPAR2	-570	-561	-	6.51	GCTCCAGGC

LPAR2		-478	-469	+	6.45	GCCGCGAGG
LPAR2		-413	-404	-	6.57	CCCGGCGGC
LPAR2		-381	-372	+	6.45	GCCGCGAGG
LPAR2		-276	-267	+	6.03	GCCGCCAGG
IF.AR2		-370	-361	-	6.82	CCCCAAGGC
IL10RB		-624	-615	+	6.57	GCCTCCGGG
IL10RB		-377	-368	-	5.89	GCGGGGGGC
IL10RB		-353	-344	+	7.49	GCCCGGGGG
IL10RB		-340	-331	+	6.79	GCCGCAGGG
IL10RB		-236	-227	+	6.39	GCCCGGCGG
IL10RB		-207	-198	-	6.51	GCTCCCGGC
IF.AR1		-672	-663	+	7.47	GCCCGGGGC
IF.AR1		-657	-648	+	6.99	GCCCCCGGC
IF.AR1		-554	-545	+	6.31	GCCCCGCGC
IF.AR1		-486	-477	+	7.00	GCCCCCGGG
IF.AR1		-444	-435	-	6.44	GCTCGAGGC
IF.AR1		-432	-423	+	7.04	GCCGGGGGC
IF.AR1		-390	-381	+	5.89	GCCGCGCGG
IF.AR1		-222	-213	+	6.78	GCCGCAGGC
PTPRM		-741	-732	+	7.07	GCCCGCGGG
PTPRM		-732	-723	+	6.78	GCCGCAGGC
PTPRM		-695	-686	-	6.45	CCTCGCGGC
PTPRM		-525	-516	+	6.98	GCCGCGGGC
PTPRM		-503	-494	-	6.99	CCCCGAGGC
PTPRM		-487	-478	+	6.64	GCCGGCGGG
PTPRM		-444	-435	-	6.37	GCGCCGGGC
PTPRM		-407	-398	+	6.64	GCCTGCGGG
PTPRM		-372	-363	+	6.31	GCCCCGCGC
PTPRM		-345	-336	+	5.90	GCCAGCGGC
PTPRM		-328	-319	+	7.49	GCCCGGGGG
PTPRM		-316	-307	-	6.87	GCTCGGGGC
PTPRM		-280	-271	-	5.88	GCGCGCGGC
PTPRM		-249	-240	+	6.63	GCCGGCGGC
	2	MA0146.1	Zfx	130.765.950.942	0.749369326	-456:-186
list		of	hits			
HP1BP3		-453	-439	-	5.30	CAGACCCCGCC
HP1BP3		-389	-375	-	5.11	GAGGCTCCGC
HP1BP3		-355	-341	+	5.57	GGGGGCGCGG
HP1BP3		-338	-324	+	5.33	GGGGGCGGGG
HP1BP3		-272	-258	+	10.81	GGCGGCTGGG
HP1BP3		-210	-196	-	6.21	GAGGCCGTGC
SH2D5		-200	-186	-	6.10	CGGGCCGGGG

KIF17	-432	-418	+	6.31	CGCGCGCCGG
KIF17	-292	-278	+	7.29	GCGGCCGGGC
KIF17	-265	-251	+	5.13	TGCGGCGGCG
KIF17	-227	-213	+	8.95	CCGGGCAGGG
KIF17	-186	-172	-	7.29	CAGGCGGCGG
A.KRD50	-434	-420	-	5.33	CAGCCCCGCC
A.KRD50	-322	-308	-	6.02	CAGGCACGGG
A.KRD50	-239	-225	-	7.92	AAGGCCGAGC
A.KRD50	-225	-211	+	5.33	GGGGGCGGGG
LOC1006300	-343	-329	+	6.95	CAGCCCTTGGC
EI24	-456	-442	-	5.80	CAGGACTCGGC
EI24	-427	-413	+	5.29	GGCGGCGCGG
EI24	-347	-333	+	5.41	CTCGCCCCGGA
EI24	-295	-281	+	5.66	GCGGCCCGGG
LOC1000520	-433	-419	+	5.62	GCGGGCCGGC
LOC1000520	-383	-369	-	7.57	GAGGCCGGAC
LOC1000520	-317	-303	+	5.15	CCGCCCGCGCC
ATP13A1	-441	-427	+	6.65	CCGGCCACAGC
ATP13A1	-346	-332	+	5.27	GGCGCCCAGG
ATP13A1	-219	-205	+	5.25	TGGGGCCCGG
ATP13A1	-195	-181	-	6.27	CAAGCCCGGGC
GMIP	-386	-372	+	6.39	GTTTCCTGGGC
GMIP	-360	-346	+	5.45	CAGGCCGAGG
GMIP	-343	-329	+	9.10	ACGGGCCGGG
GMIP	-326	-312	+	5.04	CGCGGCGGGG
GMIP	-305	-291	+	10.73	CGGGGCGGGG
GMIP	-240	-226	+	5.34	CGGGGCCGGG
LPAR2	-358	-344	-	7.41	GAGGCCCAAG
LPAR2	-210	-196	-	6.09	GAGGCTGGGG
IL10RB	-334	-320	+	5.73	GGGGGCGGGG
IL10RB	-279	-265	-	5.55	GAGGGCCGGG
IL10RB	-245	-231	+	5.62	GGGGCTTGGG
IF.AR1	-440	-426	-	5.32	GAGGCAGCGC
IF.AR1	-391	-377	+	8.24	AGCCGCGCGG
IF.AR1	-343	-329	+	6.62	AGGGTCAAGG
IF.AR1	-218	-204	-	7.28	CAGGCCCAACA
PTPRM	-456	-442	-	6.48	CGGGCCGCGG
PTPRM	-186	-172	+	6.23	GCGGGCGCGC

3 MA0162.1 Egr1 144.555.832.005 0.739393945 -400:-301

list of hits

HP1BP3	-370	-359	-	7.83	CCGCTCCCGCG
HP1BP3	-341	-330	+	10.43	CGCGGGGGCG

HP1BP3	-324	-313	+	5.20	CGCGGGGGCG
SH2D5	-390	-379	+	4.51	CGCGGGCGCG
SH2D5	-368	-357	+	4.44	CACGTGGGGG
SH2D5	-341	-330	+	5.20	CGCGGGGGCG
SH2D5	-301	-290	+	6.62	GCCGGGGGCG
KIF17	-393	-382	+	4.55	GGCGGGAGCG
KIF17	-302	-291	+	5.22	TGCGGCGGCG
A.KRD50	-333	-322	-	4.53	CCGCACACGCC
AVPR1A	-363	-352	+	5.40	TGCGGGGGGG
CHEK1	-385	-374	+	7.00	TGTGTGGGAG
STT3A	-390	-379	-	4.75	CCGCCTCCAGG
LOC1000520	-317	-306	-	4.68	CCGCCCGCGCC
GMIP	-333	-322	-	7.46	CCTCCTCCGCG
LPAR2	-346	-335	+	5.06	TGCGGGCGCG
IL10RB	-400	-389	+	5.20	CGCGGGGGCG
IL10RB	-352	-341	+	7.98	CCCGGGGGCG
IF.AR1	-305	-294	+	4.82	TACGAGGGCG
4	MA0035.2	Gata1	108.778.685.237	0.372818182	-2117:-1866
list	of	hits			
EIF4G3	-2100	-2089	+	6.94	AGAGATAATG
EIF4G3	-1975	-1964	-	7.36	CTGTTATCACC
EIF4G3	-1890	-1879	+	6.66	ACAGAGAAGA
HP1BP3	-1926	-1915	+	7.44	TGAGATAAACG
SH2D5	-2105	-2094	-	7.59	CGCTTATCAGG
WWOX	-2088	-2077	+	7.78	GAAGATAAGC
BCO1	-1937	-1926	+	6.50	GCTGATAATGG
A.KRD50	-2083	-2072	+	6.87	GGAGATAAAT
AVPR1A	-1883	-1872	-	8.81	TTTTTATCTCT
CHEK1	-2014	-2003	-	6.74	TTGTTATCAAT
EI24	-2067	-2056	+	6.64	AAAGATAGGC
LOC1006301	-1992	-1981	+	9.42	GGAGATAAGA
LOC1000520	-2117	-2106	+	8.79	TGAGATAAGC
LOC1000520	-2103	-2092	-	7.57	TTATTATCTCT
LOC1000520	-1950	-1939	-	6.25	TTCATATCTGT
LOC1000548	-2099	-2088	-	7.84	TTATTATCTGT
LOC1000548	-2077	-2066	-	7.03	ATTTTATCACT
IL10RB	-2021	-2010	-	6.87	ACCTTATCATA
IL10RB	-1920	-1909	-	7.45	CTTTTATCTAT
IF.AR1	-1866	-1855	+	6.38	AGAGATAAATT
5	MA0079.2	SP1	111.288.627.426	0.820000010	-232:-121
list	of	hits			

EIF4G3	-223	-213	+	5.74	CGCTTCCCCC
EIF4G3	-213	-203	-	8.47	GGGGAGGGGG
EIF4G3	-194	-184	+	9.04	CCCCGCCGCC
EIF4G3	-149	-139	+	8.00	CTCCGCCTCC
EIF4G3	-122	-112	+	6.62	GCCCCCCCCC
HP1BP3	-230	-220	-	6.30	GGCGGCGGGA
HP1BP3	-185	-175	-	6.60	GGAAGGGGGG
HP1BP3	-141	-131	-	6.58	GGGGAGTGGG
SH2D5	-200	-190	-	5.71	CGGGCCGGGG
SH2D5	-189	-179	-	7.07	TGGGGCTGGG
SH2D5	-171	-161	-	8.78	GGGAGCGGGG
SH2D5	-140	-130	-	7.02	GGAGGGAGGG
SH2D5	-123	-113	-	6.82	TGGGGAGGAG
KIF17	-154	-144	+	6.90	CCCCGCCGCT
KIF17	-143	-133	+	6.66	CCCCCTCCTC
WWOX	-190	-180	-	6.82	CGGGGAGGAG
WWOX	-132	-122	-	7.72	TGAGGAGGGG
A.KRD50	-225	-215	-	11.09	GGGGGCGGGG
A.KRD50	-213	-203	-	7.84	GGGGCCGGGG
A.KRD50	-189	-179	-	8.96	TGGGGCGGGG
A.KRD50	-179	-169	-	6.96	CGGGGCGGAG
AVPR1A	-231	-221	-	7.02	GGAGGGTGGG
AVPR1A	-123	-113	-	7.07	AGGGGCTGGG
ACRV1	-218	-208	+	8.04	CCCCTCCTTC
EI24	-231	-221	-	8.89	GGCGGAGGGG
EI24	-166	-156	-	7.04	GGCGGCGGAG
LOC1000520	-140	-130	-	7.71	GGGGGCGGGC
GMIP	-208	-198	+	7.07	CCCAGCCCCG
LPAR2	-232	-222	+	5.86	CCCTGTCCTC
LPAR2	-211	-201	-	8.11	GGAGGCTGGG
LPAR2	-188	-178	-	6.22	AGGGGCGGGA
LPAR2	-165	-155	-	11.09	GGGGGCGGGG
LPAR2	-140	-130	-	7.14	TAGGGCGGGG
IL10RB	-131	-121	-	8.81	CGGGGAGGGG
IF.AR1	-180	-170	-	6.76	CGCGGAGGGG
IF.AR1	-159	-149	-	5.98	AGGGGGAGGG
IF.AR1	-138	-128	-	8.96	AGGGGCGGGG
IF.AR1	-121	-111	-	6.58	GGAGACAGGG
PTPRM	-175	-165	+	6.79	CTCCGCTCCC
PTPRM	-133	-123	-	7.15	GGCGGCAGGG

	6	MA0112.2	ESR1	135.630.609.902	0.594271941 -195:-56
list		of	hits		

EIF4G3	-102	-82	-	5.61	TTGTCACGGAG
HP1BP3	-133	-113	+	4.98	GGCGCGGGTC(
SH2D5	-165	-145	-	7.12	GGGGCTCGGT(
WWOX	-110	-90	+	5.76	TGGGCGGGGC(
BCO1	-174	-154	-	8.01	GGGCCAGAGT(
A.KRD50	-69	-49	-	6.29	GGGGCAGCTG(
AVPR1A	-195	-175	+	4.82	TCCCGGAGCCA
ACRV1	-56	-36	+	5.39	TACCCGAATCA
LOC1006300	-189	-169	+	10.87	GCCACTGGTCA
LOC1006300	-126	-106	-	5.51	AGGGCAGTGG(
STT3A	-189	-169	-	5.22	ATGGCAGAGG(
EI24	-58	-38	-	6.02	AGGGCAGGCA(
LOC100630	-167	-147	+	7.83	GCCACTGATCA
GMIP	-129	-109	-	4.93	ATGCGAGAGTG
LPAR2	-164	-144	+	6.66	GGGGCGGGGC
LPAR2	-56	-36	+	4.89	AGCCGCGGTCG
IF.AR2	-121	-101	+	5.39	GAAGGGAGTC
IL10RB	-192	-172	-	5.97	ATGGCACGGAG
IL10RB	-64	-44	+	6.24	CGGCCCAGGG

7	MA0081.1	SPIB	906.007.130.057	0.466472323	-3192:-3018
list	of	hits			
EIF4G3	-3190	-3183	-	7.09	TTCCTGT
SH2D5	-3158	-3151	+	6.55	TCAGGAA
SH2D5	-3138	-3131	+	6.55	TCAGGAA
SH2D5	-3091	-3084	-	6.60	TTCCCCT
SH2D5	-3058	-3051	-	6.55	TTCCTGA
WWOX	-3038	-3031	+	7.09	ACAGGAA
BCO1	-3128	-3121	+	7.28	TGAGGAA
AVPR1A	-3060	-3053	+	7.28	TGAGGAA
LOC1006300	-3018	-3011	-	6.55	TTCCTGA
LOC100630	-3174	-3167	-	7.09	TTCCTGT
LOC1000520	-3190	-3183	-	7.09	TTCCTGT
LOC1000520	-3126	-3119	-	7.28	TTCCTCA
LOC1000548	-3094	-3087	+	7.28	TGAGGAA
GMIP	-3178	-3171	+	6.60	AGGGGAA
IF.AR2	-3027	-3020	-	7.28	TTCCTCA
IF.AR1	-3095	-3088	+	6.60	AGGGGAA
PTPRM	-3192	-3185	-	7.82	TTCCTCT
PTPRM	-3159	-3152	-	7.82	TTCCTCT

8	MA0259.1	HIF1A::ARNT	973.982.402.355	0.657451923	-1807:-1701
list	of	hits			

HP1BP3	-1736	-1728	+	7.08	GCACGTGG
HP1BP3	-1718	-1710	+	6.79	GCGCGTGC
SH2D5	-1760	-1752	+	7.08	GCACGTGG
LPAR2	-1725	-1717	+	6.95	GGGCGTGC
IF.AR2	-1807	-1799	+	7.23	GGACGTGG
IL10RB	-1701	-1693	-	6.96	GCACGTTC

9	MA0084.1	SRY	919.301.452.288	0.238095234	-2451:-2316
list	of	hits			

EIF4G3	-2376	-2367	+	7.84	TATAACAAT
HP1BP3	-2402	-2393	-	6.55	ATTGTGAAA
SH2D5	-2375	-2366	+	6.57	TGAAACAAA
KIF17	-2407	-2398	+	6.69	GAAGACAAT
WWOX	-2441	-2432	-	6.49	ATTGTGTAG
WWOX	-2349	-2340	+	6.98	GAATACAAT
A.KRD50	-2448	-2439	-	6.22	ATTGATAAA
A.KRD50	-2318	-2309	+	7.55	GTTAACAAA
AVPR1A	-2333	-2324	+	6.29	GATCACAAT
CHEK1	-2322	-2313	+	6.47	TAACACAAT
MCMDC2	-2316	-2307	+	6.49	CTACACAAT
LOC1000520[illegible]	-2451	-2442	-	6.49	ATTGTCTAG
LOC1000520[illegible]	-2409	-2400	-	7.25	TTTGTTTTA
LOC1000548[illegible]	-2424	-2415	-	6.57	ATTGTAATC
LOC1000548[illegible]	-2332	-2323	+	8.32	TTTAACAAT
ATP13A1	-2404	-2395	-	6.83	ATTGTAAAA
LPAR2	-2376	-2367	+	6.87	CTTAACAAA
IF.AR2	-2449	-2440	+	6.79	CAAAACAAA

10	MA0140.1	Tal1::Gata1	112.966.184.617	0.450677699	-3197:-2457
list	of	hits			

EIF4G3	-2714	-2696	+	5.68	TTGCTTGCAGT[illegible]
SH2D5	-2529	-2511	-	5.74	GTAATCTCCAG[illegible]
WWOX	-2457	-2439	-	6.34	TTTATCAAATA[illegible]
BCO1	-2862	-2844	-	7.48	CTTATCTCACC[illegible]
A.KRD50	-2457	-2439	+	6.75	ATTTGGGTGAT
AVPR1A	-3017	-2999	+	7.37	TTGTGAAACAT
AVPR1A	-2881	-2863	-	5.60	TCTATCAATAC[illegible]
CHEK1	-3074	-3056	-	7.99	CTTATCTCTTAC
ACRV1	-3026	-3008	-	7.28	GTTATCTTCAG[illegible]
ACRV1	-2721	-2703	-	6.69	CTCATCTTTTTG
LOC1006300[illegible]	-3197	-3179	+	7.34	CTCAGAGAAA[illegible]
STT3A	-3108	-3090	+	6.08	GAGTAAGGGA[illegible]
STT3A	-3000	-2982	+	9.18	TTGGATCATTT[illegible]

STT3A	-2516	-2498	+	7.60	TTGGAGATTTC/
EI24	-3114	-3096	-	6.61	TTTATCAGTTAT
EI24	-2867	-2849	-	7.16	GTTATCTCTTTA
EI24	-2787	-2769	+	6.69	CCACAACTTTC/
EI24	-2615	-2597	+	6.13	AGAGCTTCTAG
LOC1006301(	-3158	-3140	-	5.92	TTTATCTAAATT
MCMDC2	-2511	-2493	-	6.64	CTTATCGCTTG(
LOC1000520:	-2762	-2744	-	7.15	TTTATCTGCTAT
LOC1000520:	-2564	-2546	-	6.77	CCTATCATTATA
LOC1000520:	-2469	-2451	-	6.92	CTTATCTCATTG
LOC1000548:	-3196	-3178	-	6.21	TTTATCACTAA/
LOC1000548:	-2670	-2652	-	5.77	CATATCATATTT
ATP13A1	-3039	-3021	+	7.33	ACTGAGGGGT(
ATP13A1	-2580	-2562	+	9.27	ATGGTACATTC/
LPAR2	-2641	-2623	-	8.03	CATATCTCCTAT
IF.AR2	-3020	-3002	-	7.18	GTTATCACTAT(
IF.AR1	-3197	-3179	-	6.34	CTTATCAAAAG(
IF.AR1	-3028	-3010	+	6.39	TTTGGCAAAAC
IF.AR1	-2881	-2863	+	9.01	GGGGGACGGG
IF.AR1	-2539	-2521	+	7.28	GAGGAAATATT
PTPRM	-2569	-2551	+	5.68	GTTTGCTAGCT

11	MA0117.1	Mafb	676.893.922.026	0.608333337	-185:-53
list	of	hits			

EIF4G3	-90	-82	-	5.80	CCGGCAGC
EIF4G3	-53	-45	+	5.80	GCTGCCGG
HP1BP3	-79	-71	+	6.28	GCCGACGC
KIF17	-61	-53	+	5.80	GCTGCCGC
A.KRD50	-125	-117	-	5.80	GCGGCAGC
AVPR1A	-154	-146	-	6.28	GCGTCGGC
ACRV1	-93	-85	-	5.76	GTTTCAGC
LOC1006300	-56	-48	-	5.76	CTATCAGC
EI24	-185	-177	+	5.80	GCTGCCGC
EI24	-157	-149	+	5.80	GCTGCCGG
LOC1000548:	-184	-176	+	7.02	GCTGACTG
ATP13A1	-150	-142	+	5.76	GCTGATAG
IF.AR2	-116	-108	-	7.02	GAGTCAGC
IF.AR2	-98	-90	+	6.60	GCTGATGG
PTPRM	-146	-138	+	5.73	GCCGACTG

12	MA0158.1	HOXA5	875.888.972.496	0.319270834	-2988:-2946
list	of	hits			

EIF4G3	-2958	-2950	+	7.27	CTCTAATT

WWOX	-2974	-2966	-	6.94	AATTACAG
BCO1	-2974	-2966	-	7.09	AATTTCTG
ATP13A1	-2962	-2954	+	6.94	CTGTAATT
LPAR2	-2951	-2943	+	6.87	CATAAATT
IF.AR1	-2988	-2980	+	6.54	CGTAAATT
IF.AR1	-2946	-2938	+	7.27	CATTAATT

13	MA0147.1	Myc	11.157.239.362	0.685903085	-1790:-1691
list	of	hits			

HP1BP3	-1737	-1727	+	9.75	AGCACGTGGT
HP1BP3	-1719	-1709	-	5.30	TGCGCGTGCC
SH2D5	-1768	-1758	+	6.11	AGCACTTGGC
KIF17	-1790	-1780	+	6.26	CCCATGTGGA
KIF17	-1723	-1713	+	7.23	ATCACGTGGT
KIF17	-1691	-1681	-	5.69	CCCATGTGTG
BCO1	-1766	-1756	+	9.16	AACACGTGGC
BCO1	-1723	-1713	+	5.63	TGCACGCGTT
GMIP	-1703	-1693	+	5.25	AGCACTTGGA

14	MA0028.1	ELK1	881.228.537.154	0.567857146	-771:-296
list	of	hits			

EIF4G3	-410	-400	+	6.80	TCTCCGGAAA
WWOX	-750	-740	+	6.13	TGAACGGAAG
WWOX	-326	-316	-	7.19	CGTCCGGCTG
BCO1	-321	-311	+	6.41	GAACAGGAAG
A.KRD50	-662	-652	+	6.21	CACCCGGACA
AVPR1A	-753	-743	-	5.92	TGTCCGGAGG
AVPR1A	-647	-637	+	6.06	GCCCCGGACA
LOC1006300	-381	-371	-	6.69	CTTCAGGCAC
STT3A	-568	-558	-	5.91	TTTCCGCTTC
MCMDC2	-706	-696	-	5.88	CTTCCGTGCT
LOC1000520	-382	-372	+	6.61	AGGCCGGACA
ATP13A1	-611	-601	+	6.29	CAGCCGGATG
ATP13A1	-578	-568	-	8.86	CTTCCGGTTC
ATP13A1	-529	-519	-	7.93	TTTCCGGTCT
ATP13A1	-444	-434	-	7.56	CTTCCGGCCA
ATP13A1	-403	-393	-	7.42	CTTCCGGACT
ATP13A1	-334	-324	+	7.45	TCACCGGAAG
ATP13A1	-296	-286	+	6.65	TGCCCGGAAG
GMIP	-771	-761	+	6.58	GGACCTGAAG
LPAR2	-305	-295	+	8.46	GCGCCGGAAG
IF.AR2	-747	-737	-	5.83	TTTCCTGTAC
IF.AR2	-452	-442	+	5.94	GGGCAGGAAA

IL10RB		-756	-746	+		6.61	GTGCCTGAAA
IL10RB		-372	-362	+		6.96	GGGCCGGACA
IF.AR1		-417	-407	+		6.06	CAACAGGAAG
15	MA0136.1		ELF5		869.309.550.562	0.429292928	-2105:-2056
list	of		hits				
KIF17		-2105	-2096	-		7.29	CAGGAAGTG
KIF17		-2056	-2047	-		6.36	GAGGAAGCA
BCO1		-2101	-2092	-		6.59	AAGGATGCA
BCO1		-2060	-2051	-		6.98	ACGGATATA
A.KRD50		-2061	-2052	-		6.49	AAGGAAGGA
ACRV1		-2099	-2090	-		7.35	AAGGAACTA
STT3A		-2102	-2093	-		7.29	CAGGAAGTG
GMIP		-2086	-2077	+		6.34	TTCTTCCTG
IF.AR1		-2081	-2072	-		7.41	CAGGAAATT
16	MA0025.1		NFIL3		1.413.850.704	0.264822136	-1792:-1480
list	of		hits				
EIF4G3		-1782	-1771	-		4.60	GTATTACAAAT
WWOX		-1767	-1756	-		4.49	GAGTTACATAC
A.KRD50		-1646	-1635	+		5.18	TTCTGTAACTT
A.KRD50		-1563	-1552	-		4.42	TTCTTACTTAA
A.KRD50		-1539	-1528	+		7.77	ATATGTAATTT
AVPR1A		-1770	-1759	+		5.50	TGATATAACCG
LOC1006300		-1722	-1711	+		4.76	ATATATAAGTT
LOC1006300		-1582	-1571	-		7.77	AAACTACATAA
EI24		-1781	-1770	+		4.60	ATTTGTAAGAT
EI24		-1541	-1530	+		4.72	CTATGTAATAA
LOC1006301		-1505	-1494	-		5.07	GAGTTACATCT
LOC1000548		-1653	-1642	+		4.68	TTATGGAATAA
LOC1000548		-1523	-1512	+		7.64	ATATGTAAGGT
GMIP		-1487	-1476	-		4.60	TCATTACAAAT
IF.AR2		-1480	-1469	-		7.86	AAATTATGTAA
IL10RB		-1640	-1629	-		7.83	CGGTTACATCA
IF.AR1		-1792	-1781	+		5.63	TTATATAGCAA
IF.AR1		-1691	-1680	+		4.45	ATACATAACTC
PTPRM		-1708	-1697	+		9.35	TGATGTAACGT
17	MA0154.1		EBF1		115.644.748.683	0.648000004	-1610:-1501
list	of		hits				
SH2D5		-1596	-1586	-		6.01	TCCCTGTGAG
SH2D5		-1552	-1542	+		5.58	ATTAAAGGGA
WWOX		-1502	-1492	+		7.78	CCTCCTGGGA

AVPR1A		-1610	-1600 -		5.45	CCCCTTTGGA
STT3A		-1564	-1554 -		5.39	TCCCTTTAAG
LOC1006301(		-1572	-1562 +		7.44	TCCCTGGGGA
MCMDC2		-1578	-1568 -		6.30	TCCCAGGAGC
MCMDC2		-1519	-1509 -		7.09	TCTCCTGGGT
MCMDC2		-1501	-1491 -		6.60	TCCCTTAGAG
LOC1000548:		-1538	-1528 -		8.24	TCTCTTGGGT
LPAR2		-1595	-1585 -		8.48	TCCCCTGAGG
LPAR2		-1541	-1531 +		7.09	ACCCAGGAGA
IF.AR2		-1573	-1563 +		5.89	TCTTTAGGGA
PTPRM		-1561	-1551 +		7.62	GCCCCGGGGA
18	MA0080.2		SPI1	964.024.729.829	0.435374151	-2194:-2080
list	of		hits			
KIF17		-2168	-2161 +		6.31	AGGAAGA
KIF17		-2104	-2097 +		8.65	AGGAAGT
AVPR1A		-2155	-2148 +		6.31	AGGAAGA
AVPR1A		-2121	-2114 -		6.31	TCTTCCT
ACRV1		-2194	-2187 -		6.31	TCTTCCT
ACRV1		-2098	-2091 +		6.28	AGGAACT
STT3A		-2101	-2094 +		8.65	AGGAAGT
EI24		-2193	-2186 -		6.81	GCTTCCT
LOC1006301(		-2183	-2176 -		6.78	ATTTCCT
ATP13A1		-2120	-2113 +		6.78	AGGAAAT
GMIP		-2085	-2078 -		6.31	TCTTCCT
LPAR2		-2133	-2126 -		6.31	TCTTCCT
IF.AR2		-2166	-2159 +		6.78	AGGAAAT
IF.AR1		-2080	-2073 +		6.78	AGGAAAT
PTPRM		-2163	-2156 -		8.65	ACTTCCT
PTPRM		-2150	-2143 -		6.96	ACTTCCC
19	MA0080.2		SPI1	964.024.729.829	0.435374151	-1718:-1622
list	of		hits			
EIF4G3		-1706	-1699 -		6.81	GCTTCCT
HP1BP3		-1701	-1694 +		6.31	AGGAAGA
SH2D5		-1660	-1653 -		8.65	ACTTCCT
KIF17		-1649	-1642 -		6.81	GCTTCCT
AVPR1A		-1692	-1685 -		6.78	ATTTCCT
LOC1006300'		-1703	-1696 +		6.31	AGGAAGA
MCMDC2		-1718	-1711 -		6.81	GCTTCCT
LOC1000520:		-1657	-1650 +		6.78	AGGAAAT
GMIP		-1660	-1653 -		6.78	ATTTCCT
LPAR2		-1635	-1628 +		6.81	AGGAAGC

IF.AR1	-1656	-1649	+	6.31	AGGAAGA
IF.AR1	-1622	-1615	-	6.31	TCTTCCT
PTPRM	-1691	-1684	+	6.45	AAGAAGT
PTPRM	-1637	-1630	+	6.42	AGAAAGT
20	MA0161.1	NFIC	698.267.924.778	0.568504061	-617:-574
list	of	hits			
SH2D5	-608	-602	+	5.83	CTGGCA
WWOX	-606	-600	+	5.83	CTGGCA
CHEK1	-584	-578	-	6.73	TGCCAA
ACRV1	-617	-611	-	5.91	AGCCAA
ACRV1	-593	-587	-	5.91	AGCCAA
ACRV1	-574	-568	-	5.91	AGCCAA
LOC1006300	-592	-586	-	5.91	AGCCAA
IL10RB	-615	-609	+	6.73	TTGGCA
21	MA0149.1	EWSR1-FLI1	328.714.828.257	0.554497355	-982:-893
list	of	hits			
EIF4G3	-898	-880	-	-8.21	CTTTCCTTCCCC
HP1BP3	-912	-894	-	-8.41	AATTCCCTCTTT
SH2D5	-982	-964	+	-8.41	GGCCCGGAGG
SH2D5	-923	-905	+	1.99	AGGATGACGG
BCO1	-921	-903	+	-7.36	GGAAGATGGG
AVPR1A	-916	-898	-	-8.39	AAGTGCTTCTT
CHEK1	-915	-897	-	-6.94	CTCACATTACT
ACRV1	-968	-950	+	-2.58	GAACAAAAAG
ACRV1	-899	-881	+	-6.29	CTAAGGAAGC
LOC1006300	-893	-875	+	-8.09	GGAAAGGAAG
STT3A	-981	-963	+	-3.04	GGAGAGAGGG
EI24	-969	-951	+	-3.37	GCTAGGAAGG
EI24	-947	-929	+	-8.25	GGGGGGGGGG
EI24	-921	-903	+	-3.05	GAAAAAAAAG
MCMDC2	-917	-899	+	-6.23	GGGGGAAGGG
LOC1000548	-959	-941	+	-7.78	CAAAAGAAGG
LPAR2	-970	-952	+	-7.23	GGAAGGGTGA

2020

ratio=3.0, top=25

of	the	matrix	
the	matrix	(G+C)%	
window	(relatively	to	TSS)
window			

2,84E-03

7,48E+04

ACCCCGCCCGG
GCTCCGCGCGC
GGCGCGGCCGG
GGCGGGGCCGG
GGCTGGGCCTG
GCCGTGCGGGA
GCCGGGGCTGG

GCGCCGGCCTC	
GCCGGGCCCTG	
GGCGGCGCCTG	
GGCAGGGCCTC	
GCGGCGGCCCC	
CCCCCGCCCCC	
GCACGGGCGGC	
GCCGAGCGGCC	
GGCGGGGCCGG	
CCCTTGGCCTG	
GACTCGGCGTC	
GGCGCGGCCGG	
GCCCCGGACTG	
GCCCGGGCATG	
GGCCGGCCCTC	
GCCGGACAGCC	
CCCGCGCCCTC	
GCCACAGCCTG	
GCCCAGGGCTC	
GGCCCGGCCCG	
GCCCGGGCCGC	
TCCTGGGCCTT	
GCCGAGGCGTG	
GGCCGGGCCTC	
GGCGGGGCGTG	
GGCGGGGCCTC	
GGCCGGGCCCC	
GCCCAAGACTG	
GCTGGGGTCCC	
GGCGGGGCCCC	
GGCCGGGCTGG	
GCTTGGGCCCG	
GCAGCGCCGGG	
CGCGCGGCCTG	
GTCAAGGCCTC	
GCCCAACACTC	
GCCGCGGCTGC	
GGCGCGCCCTC	
	2,15E+05
CTCCCGCG	
GGGGGCGG	

GGGGGCGC
GGGCGCGG
GTGGGGGG
GGGGGCGC
GGGGGCGG
GGGAGCGA
GGCGGCGG
CACACGCC
GGGGGGGG
GTGGGAGA
CCTCCAGG
CCCGCGCC

GGGCGCGT
GGGGGCGC
GGGGGCGG
GAGGGCGG

9,14E+05

GATAATGC

GAGAAGAA
GATAAACG
TTATCAGG
GATAAGCT
GATAATGG
GATAAATG

GATAGGCA
GATAAGAG
GATAAGCA

GATAAATT

9,64E+05

GAGGGGG

AGGGGGG
GAGTGGG

AGCGGGG
GGGAGGG

GGAGGAG

GGCGGGG
GCCGGGG

GGGTGGG

GGAGGGG

GGCGGGC

GGCGGGA
GGCGGGG

GGAGGGG

GGGAGGG
GGCGGGG

1,10E+04

ACCGGCAGC
CACTGGACCC
CCCCAGGCGC
GCGGGGCCGCCTGCCCG
CCAGAGTGACAGGTGAG
GCAGCTGGAGCTGGCAG
CGGAGCCAAGCTGCCCC
CCGAATCAATCTGACTC
ACTGGTCATCCTGTCCT
GCAGTGGGACTGAGGAG
GCAGAGGGAGCCGAGCG
GCAGGCAGGGCCGGAGC
ACTGATCATCCTGTCCT
CGAGAGTGGCCTCCCCC
GCGGGGCGCTGTGGCCC
GCCGAGCCAT
AGCCTGGCTT
GCCTCTGGAG
ACCCCGACCC

2,96E+06

3,10E+05

3,15E+06

4,19E+05

CTTGCAGTAGATTAA
ATCTCCAGCCCCAGG
ATCAAATATTTACAT
ATCTCACCATGTAAT
TGGGTGATTGATAAA
TGAAACATTGATAAA
ATCAATACCTTCAAC
ATCTCTTACGTAAGA
ATCTTCAGGTCTCCG
ATCTTTTTGAACCAG
AGAGAAAGTGATAAG
TAAGGGAGAGATTAG
GATCATTTAGATAAG

GAGATTTCAGATAGA
ATCAGTTATAACACT
ATCTCTTTACAAGAC
CAACTTTCAGATAAG
GCTTCTAGAGATAAG
ATCTAAATTCTACTA
ATCGCTTGCTTCCAT
ATCTGCTATAAAACA
ATCATTATATGACAA
ATCTCATTGGAATAT
ATCACTAAATTGCTA
ATCATATTTGCCCAT
GAGGGGTGAGATAAA
GTACATTCAGATAAC
ATCTCCTATGCACAG
ATCACTATCCCCAGA
ATCAAAAGGAACAGA
GGCAAAACTGATAGA
GGACGGGGAGATAAA
GAAATATTAGATAAG
TGCTAGCTTGATAAA

4,63E+06

4,88E+06

5,40E+05

5,42E+06

5,55E+06

5,72E+06

TTACATAC

TATAACCG

CTACATAA

TGGAATAA
TGTAAGGT

TGTAACGT

6,07E+06

7,17E+06

7,37E+06

7,90E+06

9,58E+05

TCCTTCCCCCAGCCT
TCCCTCTTTTCTTTT
CCGGAGGGAGGAGGA
ATGACGGAAGGAAGG
AGATGGGCACGGATG
TGCTTCTTTCCTTTT
ACATTACTTACCTCC
CAAAAAGAAGGAAAG
AGGAAGCAGGGGAAG
AAGGAAGTAGTAAAA
GAGAGGGCAAAAAGG
AGGAAGGAAAATAGG
GGGGGGGGGCAAAG
AAAAAAGAAGAAATG
GGAAGGGAAGGTTAC
AAGAAGGTACTAAGG
AGGGTGACAGGGGAG
