# Supplementary material for: Identification and Functional Annotation of Genes Related to Horses’ Performance: From GWAS to Post-GWAS
Source: Animals (Basel). 2020 Jul 10;10(7):1173. doi: 10.3390/ani10071173 (PMC7401650; doi:10.3390/ani10071173)
Supplement: Supplementary file 1 [file animals-10-01173-s001.zip › supplementarymaterials/TFM_explorer_out/tfmexplorer_group2.pdf]

```
;TFM Explorer
;Date               : Wed May 13 06:26:21 2020
;Scanned location   : -3000:300
;Scanned sequences  : None (8 sequences)
;Scanned matrices   : (130 matrices)
;Parameters         : minsize= 30, maxsize= 1500, ratio=3.0, top=25
;
; headers
;    1    rank              window rank
;    2    matrix            matrix name
;    3    tf                transcription factor name
;    4    info              information of content of the matrix
;    5    gc                gc content of the matrix (G+C)%
;    6    location          location of the window (relatively to TSS)
;    7    pvalue            pvalue of the window

    1   MA0003.1        TFAP2A  7.8120920509    0.800000024164  -1678:-640
1.78361128641e-05
                list of hits
                GRM8    -1676   -1667   -       6.51    GCTCCAGGC
                GRM8    -1297   -1288   +       7.05    GCCCTAGGG
                GRM8    -0970   -961    -       6.86    CCCTCAGGC
                GRM8    -0901   -892    -       6.51    GCTCCCGGC
                GRM8    -0882   -873    +       7.04    GCCTGGGGC
                GRM8    -0796   -787    -       6.37    GCGCCGGGC
                GRM8    -0698   -689    +       6.39    GCCCGGCGG
                GRIK2   -1002   -993    +       6.02    GCCGCCAGC
                GRIK2   -0892   -883    +       6.03    GCCGCCAGG
                NEB     -1678   -1669   +       6.31    GCCTGAAGC
                CCDC148 -1453   -1444   +       7.06    GCCCGCGGC
                CCDC148 -1406   -1397   +       6.87    GCCCCGAGC
                CCDC148 -1265   -1256   -       6.57    CCCGGCGGC
                CCDC148 -1158   -1149   -       6.94    GCTCCGGGC
                CCDC148 -0955   -946    +       6.95    GCCCGGAGG
                CCDC148 -0930   -921    +       7.42    GCCCCGGGG
                CCDC148 -0913   -904    -       6.31    GCGCGGGGC
                CCDC148 -0826   -817    -       6.45    GCTGGGGGC
                CCDC148 -0708   -699    +       7.06    GCCGGGGGG
                CCDC148 -0640   -631    +       6.45    GCCGCGAGG
                ANK1    -1175   -1166   +       6.52    GCCGGGAGG
                ANK1    -1074   -1065   +       6.28    GCCCTCAGC
                ANK1    -0704   -695    -       6.99    CCCCGAGGC
                KAT6A   -1522   -1513   -       6.70    GCTCAGGGC
                KAT6A   -1470   -1461   -       6.70    GCTCAGGGC
                KAT6A   -1003   -994    +       6.26    GCCTCAAGG
                KAT6A   -0663   -654    +       6.31    GCCCCGCGC

    2   MA0139.1        CTCF    17.2051081365   0.644893727864  -852:-443
1.83288295322e-05
                list of hits
                GRM8    -0852   -833    +       9.09    CCGCCTCTAGATGGAACTT
                GRM8    -0690   -671    -       3.63    GCTCTCCATCGGCTGGAGG
                GRM8    -0576   -557    -       8.36    CCGCGCCCCCTTCTGCGCA
                MIR592  -0841   -822    +       5.15    GTTCCTCAAGGTGACAGTT
                GRIK2   -0584   -565    +       5.92    TGCACAGCGGGTGGTGGTG
                GRIK2   -0519   -500    +       5.02    ACGCCAGTAGGTGGACTAA
                NEB     -0784   -765    -       3.98    CATTATCTTCTCCTGGTAA
                NEB     -0725   -706    -       4.35    TTGAACCCTCTTGTGCCTG
                NEB     -0528   -509    -       3.79    TAATTACACCCTGTGGATA
                RIF1    -0450   -431    +       4.07    TCACCTAAAGTAGGCACTA
                CCDC148 -0642   -623    +       4.52    CGGCCGCGAGGCGGTGGGG
                CCDC148 -0591   -572    -       5.56    CGGCGCCCCTCGCAGGCAG
                ANK1    -0555   -536    +       3.60    CGAGCGGCGGGGGGAACAG
                ANK1    -0498   -479    -       7.38    CGGCGCCCCCGGCCGGACC
                ANK1    -0443   -424    +       8.93    CCGCCGCCCGGGGGCGCCC
                KAT6A   -0635   -616    +       4.29    CCTACAGCAGGGGTGAGCA
                KAT6A   -0447   -428    -       3.48    AAACGCCATCAAGAGTTCA
```

3 MA0024.1 E2F1 13.8381257118 0.625 -830:288 3.69804597541e-05

list of hits

GRM8	-0415	-407	-	8.82	GCGGCAAA
GRM8	+0134	142	+	4.20	GTTGGCGC
GRM8	+0288	296	+	4.20	CTTGGCGC
MIR592	-0360	-352	-	4.20	GAGCCAAA
GRIK2	-0830	-822	+	4.20	TTCGGCGC
GRIK2	-0733	-725	-	10.19	GCGCCAAA
GRIK2	-0542	-534	+	8.41	TTTCCCGC
GRIK2	-0210	-202	+	3.80	TCTCGCGC
GRIK2	-0202	-194	+	3.80	TTTCGCTC
NEB	+0177	185	+	4.20	TTTGGGGC
RIF1	-0072	-64	+	4.20	TTCGGCGC
RIF1	+0180	188	-	3.80	GCGCGAAC
RIF1	+0286	294	-	6.24	CCGGGAAA
CCDC148	-0745	-737	-	6.24	CCGGGAAA
CCDC148	-0012	-4	-	3.80	GAGCGAAA
CCDC148	+0040	48	+	4.20	TTGGGCGC
CCDC148	+0166	174	-	4.42	GCGTCAAA
ANK1	-0532	-524	+	6.24	TTTCCCGG
KAT6A	-0325	-317	+	6.24	TTTCCCGG
KAT6A	-0159	-151	+	6.24	TTTCCCGG
KAT6A	+0058	66	+	4.20	TTTGGGGC

4 MA0158.1 HOXA5 8.75888972496 0.319270834327 -2508:-2167 4.30556116033e-05

list of hits

GRM8	-2167	-2159	-	7.09	CATTACTG
MIR592	-2403	-2395	+	7.02	CACAAATG
MIR592	-2389	-2381	-	6.87	AATTTGAG
MIR592	-2191	-2183	-	7.09	AATTTCTG
NEB	-2342	-2334	-	6.54	CATTACAG
RIF1	-2303	-2295	-	6.94	AATTAACG
CCDC148	-2467	-2459	+	6.69	CAGAAATG
CCDC148	-2335	-2327	-	6.72	AATTAAAG
CCDC148	-2295	-2287	-	7.09	CATTACTG
KAT6A	-2508	-2500	+	7.09	CAGTAATG
KAT6A	-2340	-2332	+	7.42	CACAAATT

5 MA0003.1 TFAP2A 7.8120920509 0.800000024164 121:262 4.71030171077e-05

list of hits

GRM8	+0205	214	+	6.98	GCCGCGGGC
GRM8	+0229	238	+	5.88	GCCGCGCGC
GRM8	+0253	262	+	5.81	GCCGGTGGG
GRIK2	+0126	135	-	7.06	CCCCCCGGC
GRIK2	+0143	152	-	6.51	GCTCCCGGC
GRIK2	+0262	271	-	6.87	GCTCGGGGC
RIF1	+0213	222	-	6.45	CCTCGCGGC
RIF1	+0250	259	+	5.90	GCCCCCCGG
CCDC148	+0258	267	-	6.79	CCCTGAGGC
ANK1	+0121	130	-	5.96	GCGGCGGGC
ANK1	+0133	142	+	5.79	GCCCAGAGC
ANK1	+0144	153	-	5.52	GTCCCGGGC
ANK1	+0198	207	-	6.32	CCGCGGGGC
ANK1	+0223	232	+	5.72	GCCCTCCGC
KAT6A	+0124	133	+	5.91	GCCGAGGGG
KAT6A	+0135	144	+	6.99	GCCCCCGGC
KAT6A	+0145	154	+	7.41	GCCCCGGGC
KAT6A	+0160	169	+	7.28	GCCCGAGGC
KAT6A	+0175	184	+	7.42	GCCCCGGGG
KAT6A	+0195	204	+	5.89	GCCTCGCGG
KAT6A	+0257	266	-	5.94	GCGCCAGGC

6 MA0092.1 Hand1::Tcfe2a 10.1435823018 0.506896541268 -2108:-2072 7.15140387838e-05

list of hits

GRM8	-2072	-2062	-	5.88	CTTGCAGACC
CCDC148	-2108	-2098	-	7.04	GTACCAGACA
ANK1	-2096	-2086	+	5.84	GAGCTGGAAT

ANK1	-2086	-2076	-	7.26	TAGCCAGACT
KAT6A	-2074	-2064	+	5.85	TTTCTGGATC
