# Supplementary material for: Identification and Functional Annotation of Genes Related to Horses’ Performance: From GWAS to Post-GWAS
Source: Animals (Basel). 2020 Jul 10;10(7):1173. doi: 10.3390/ani10071173 (PMC7401650; doi:10.3390/ani10071173)
Supplement: Supplementary file 1 [file animals-10-01173-s001.zip › supplementarymaterials/TFM_explorer_out/tfmexplorer_group3.pdf]

```
;TFM Explorer
;Date              : Wed May 13 06:26:55 2020
;Scanned location  : -3000:300
;Scanned sequences : None (16 sequences)
;Scanned matrices  : (130 matrices)
;Parameters        : minsize= 30, maxsize= 1500, ratio=3.0, top=25
;
; headers
;    1    rank              window rank
;    2    matrix            matrix name
;    3    tf                transcription factor name
;    4    info              information of content of the matrix
;    5    gc                gc content of the matrix (G+C)%
;    6    location          location of the window (relatively to TSS)
;    7    pvalue            pvalue of the window

    1   MA0056.1        MZF1_1-4        8.58551308885   0.724999990314  -2998:-1715
4.13199505302e-17
                list of hits
                LOC102151104    -2173   -2167   +       7.05    TGGGGA
                INPP5J  -2722   -2716   +       7.05    TGGGGA
                SMTN    -2984   -2978   -       7.05    TCCCCA
                SMTN    -2281   -2275   -       7.05    TCCCCA
                SMTN    -1816   -1810   -       7.05    TCCCCA
                OSBP2   -2000   -1994   -       7.05    TCCCCA
                OSBP2   -1897   -1891   +       7.05    TGGGGA
                OSBP2   -1839   -1833   -       7.05    TCCCCA
                OSBP2   -1715   -1709   +       7.05    TGGGGA
                CNTN3   -2413   -2407   +       7.05    TGGGGA
                PDZRN3  -2462   -2456   -       7.05    TCCCCA
                PPP4R2  -2937   -2931   +       7.05    TGGGGA
                PPP4R2  -1968   -1962   +       7.05    TGGGGA
                PPP4R2  -1875   -1869   -       7.05    TCCCCA
                GXYLT2  -2265   -2259   -       7.05    TCCCCA
                VARS2   -2345   -2339   +       7.05    TGGGGA
                VARS2   -2107   -2101   +       7.05    TGGGGA
                VARS2   -1977   -1971   +       7.05    TGGGGA
                SFTA2   -2848   -2842   +       7.05    TGGGGA
                SFTA2   -2827   -2821   +       7.05    TGGGGA
                SFTA2   -2806   -2800   +       7.05    TGGGGA
                SFTA2   -2785   -2779   +       7.05    TGGGGA
                SFTA2   -2764   -2758   +       7.05    TGGGGA
                SFTA2   -2743   -2737   +       7.05    TGGGGA
                SFTA2   -2722   -2716   +       7.05    TGGGGA
                SFTA2   -2701   -2695   +       7.05    TGGGGA
                SFTA2   -2680   -2674   +       7.05    TGGGGA
                SFTA2   -2659   -2653   +       7.05    TGGGGA
                SFTA2   -2638   -2632   +       7.05    TGGGGA
                SFTA2   -2617   -2611   +       7.05    TGGGGA
                SFTA2   -2596   -2590   +       7.05    TGGGGA
                SFTA2   -2575   -2569   +       7.05    TGGGGA
                SFTA2   -2554   -2548   +       7.05    TGGGGA
                SFTA2   -2533   -2527   +       7.05    TGGGGA
                SFTA2   -2512   -2506   +       7.05    TGGGGA
                SFTA2   -2491   -2485   +       7.05    TGGGGA
                SFTA2   -2470   -2464   +       7.05    TGGGGA
                SFTA2   -2449   -2443   +       7.05    TGGGGA
                SFTA2   -2428   -2422   +       7.05    TGGGGA
                SFTA2   -2407   -2401   +       7.05    TGGGGA
                SFTA2   -2386   -2380   +       7.05    TGGGGA
                SFTA2   -2365   -2359   +       7.05    TGGGGA
                SFTA2   -2344   -2338   +       7.05    TGGGGA
                SFTA2   -2323   -2317   +       7.05    TGGGGA
                SFTA2   -2302   -2296   +       7.05    TGGGGA
                SFTA2   -2281   -2275   +       7.05    TGGGGA
                SFTA2   -2260   -2254   +       7.05    TGGGGA
                SFTA2   -2239   -2233   +       7.05    TGGGGA
                SFTA2   -2218   -2212   +       7.05    TGGGGA
```

```
                SFTA2   -2197   -2191   +       7.05    TGGGGA
                SFTA2   -2176   -2170   +       7.05    TGGGGA
                SFTA2   -2155   -2149   +       7.05    TGGGGA
                SFTA2   -2134   -2128   +       7.05    TGGGGA
                SFTA2   -2113   -2107   +       7.05    TGGGGA
                SFTA2   -2092   -2086   +       7.05    TGGGGA
                SFTA2   -1762   -1756   -       7.05    TCCCCA
                SFTA2   -1737   -1731   -       7.05    TCCCCA
                _GTF2H4 -2998   -2992   +       7.05    TGGGGA
                _GTF2H4 -1923   -1917   -       7.05    TCCCCA
                DAP     -2333   -2327   -       7.05    TCCCCA
                CCT5    -2855   -2849   +       7.05    TGGGGA
                CCT5    -2758   -2752   +       7.05    TGGGGA
                CCT5    -2424   -2418   +       7.05    TGGGGA
                FAM173B -2961   -2955   -       7.05    TCCCCA
                FAM173B -2942   -2936   -       7.05    TCCCCA
                FAM173B -2838   -2832   -       7.05    TCCCCA
                FAM173B -2335   -2329   +       7.05    TGGGGA

     2   MA0105.1       NFKB1   15.6269628514   0.757575756447  -2853:-2092
 8.82183505816e-17
                list of hits
                LOC102151104    -2319   -2308   +       6.30    GGGGCTGCCCT
                INPP5J  -2381   -2370   -       3.75    TGGGAACCCTC
                INPP5J  -2092   -2081   +       3.63    GGCGGTTCTCC
                SMTN    -2745   -2734   +       3.78    AGGGGTGTCCC
                SMTN    -2562   -2551   +       3.71    TGGGCTTTCCC
                CNTN3   -2832   -2821   -       2.99    GGGCCACTCCC
                CNTN3   -2576   -2565   +       5.52    GGGGGTTTGCC
                PPP4R2  -2654   -2643   -       2.97    TGGGATCCCTC
                PPP4R2  -2583   -2572   -       2.84    GGAGGACCCAC
                SFTA2   -2847   -2836   +       3.16    GGGGAGACCCG
                SFTA2   -2826   -2815   +       3.03    GGGGAGACTCC
                SFTA2   -2805   -2794   +       3.03    GGGGAGACTCC
                SFTA2   -2784   -2773   +       3.03    GGGGAGACTCC
                SFTA2   -2763   -2752   +       3.16    GGGGAGACCCG
                SFTA2   -2742   -2731   +       3.03    GGGGAGACTCC
                SFTA2   -2721   -2710   +       3.16    GGGGAGACCCG
                SFTA2   -2700   -2689   +       3.03    GGGGAGACTCC
                SFTA2   -2679   -2668   +       3.16    GGGGAGACCCG
                SFTA2   -2658   -2647   +       3.03    GGGGAGACTCC
                SFTA2   -2616   -2605   +       3.03    GGGGAGACTCC
                SFTA2   -2595   -2584   +       3.03    GGGGAGACTCC
                SFTA2   -2574   -2563   +       3.16    GGGGAGACCCG
                SFTA2   -2553   -2542   +       3.03    GGGGAGACTCC
                SFTA2   -2532   -2521   +       3.16    GGGGAGACCCG
                SFTA2   -2511   -2500   +       3.03    GGGGAGACTCC
                SFTA2   -2490   -2479   +       3.16    GGGGAGACCCG
                SFTA2   -2469   -2458   +       3.03    GGGGAGACTCC
                SFTA2   -2427   -2416   +       3.03    GGGGAGACTCC
                SFTA2   -2406   -2395   +       3.03    GGGGAGACTCC
                SFTA2   -2385   -2374   +       3.16    GGGGAGACCCG
                SFTA2   -2364   -2353   +       3.03    GGGGAGACTCC
                SFTA2   -2343   -2332   +       3.16    GGGGAGACCCG
                SFTA2   -2322   -2311   +       3.03    GGGGAGACTCC
                SFTA2   -2280   -2269   +       3.03    GGGGAGACTCC
                SFTA2   -2259   -2248   +       3.16    GGGGAGACCCG
                SFTA2   -2238   -2227   +       3.03    GGGGAGACTCC
                SFTA2   -2217   -2206   +       3.16    GGGGAGACCCG
                SFTA2   -2196   -2185   +       3.03    GGGGAGACTCC
                SFTA2   -2175   -2164   +       3.16    GGGGAGACCCG
                SFTA2   -2154   -2143   +       3.03    GGGGAGACTCC
                SFTA2   -2133   -2122   +       3.03    GGGGAGACTCC
                SFTA2   -2112   -2101   +       3.03    GGGGAGACTCC
                DAP     -2329   -2318   -       3.40    CAGAAATCCCC
                CCT5    -2853   -2842   -       3.71    GGGAAAGCACC
                CCT5    -2806   -2795   +       6.97    GGGGAGGCCCG
                FAM173B -2686   -2675   +       4.07    GGGGGTTGCCA
```

3 MA0061.1 NF-kappaB 13.3447617922 0.621052631736 -2847:-2110
5.69947050142e-11

list of hits

LOC102151104	-2319	-2309	+	7.45	GGGGCTGCCC
SMTN	-2744	-2734	+	7.09	GGGGTGTCCC
SMTN	-2561	-2551	-	7.14	GGGCTTTCCC
PDZRN3	-2230	-2220	-	4.91	AGAAGGTCCC
SFTA2	-2847	-2837	+	5.01	GGGGAGACCC
SFTA2	-2824	-2814	-	5.73	GGAGACTCCC
SFTA2	-2803	-2793	-	5.73	GGAGACTCCC
SFTA2	-2782	-2772	-	5.73	GGAGACTCCC
SFTA2	-2763	-2753	+	5.01	GGGGAGACCC
SFTA2	-2740	-2730	-	5.73	GGAGACTCCC
SFTA2	-2721	-2711	+	5.01	GGGGAGACCC
SFTA2	-2698	-2688	-	5.73	GGAGACTCCC
SFTA2	-2679	-2669	+	5.01	GGGGAGACCC
SFTA2	-2656	-2646	-	5.73	GGAGACTCCC
SFTA2	-2614	-2604	-	5.73	GGAGACTCCC
SFTA2	-2593	-2583	-	5.73	GGAGACTCCC
SFTA2	-2574	-2564	+	5.01	GGGGAGACCC
SFTA2	-2551	-2541	-	5.73	GGAGACTCCC
SFTA2	-2532	-2522	+	5.01	GGGGAGACCC
SFTA2	-2509	-2499	-	5.73	GGAGACTCCC
SFTA2	-2490	-2480	+	5.01	GGGGAGACCC
SFTA2	-2467	-2457	-	5.73	GGAGACTCCC
SFTA2	-2425	-2415	-	5.73	GGAGACTCCC
SFTA2	-2404	-2394	-	5.73	GGAGACTCCC
SFTA2	-2385	-2375	+	5.01	GGGGAGACCC
SFTA2	-2362	-2352	-	5.73	GGAGACTCCC
SFTA2	-2343	-2333	+	5.01	GGGGAGACCC
SFTA2	-2320	-2310	-	5.73	GGAGACTCCC
SFTA2	-2278	-2268	-	5.73	GGAGACTCCC
SFTA2	-2259	-2249	+	5.01	GGGGAGACCC
SFTA2	-2236	-2226	-	5.73	GGAGACTCCC
SFTA2	-2217	-2207	+	5.01	GGGGAGACCC
SFTA2	-2194	-2184	-	5.73	GGAGACTCCC
SFTA2	-2175	-2165	+	5.01	GGGGAGACCC
SFTA2	-2152	-2142	-	5.73	GGAGACTCCC
SFTA2	-2131	-2121	-	5.73	GGAGACTCCC
SFTA2	-2110	-2100	-	5.73	GGAGACTCCC
DAP	-2517	-2507	-	5.06	GCAAAGTCCC
DAP	-2328	-2318	-	7.85	AGAAATCCCC
CCT5	-2806	-2796	+	5.66	GGGGAGGCCC

4 MA0107.1 RELA 14.7568335332 0.566666663438 -2852:-2110
4.58380391565e-07

list of hits

SELM	-2801	-2791	-	6.42	GGAAAACCAC
SMTN	-2562	-2552	+	9.00	TGGGCTTTCC
SMTN	-2527	-2517	-	4.43	GGGAAGCCAG
CNTN3	-2576	-2566	+	4.58	GGGGGTTTGC
PDZRN3	-2134	-2124	+	3.79	CAGACTTTCC
PPP4R2	-2545	-2535	+	3.94	TGGCCTTTCC
PPP4R2	-2342	-2332	-	4.71	GGAATCCCAG
VARS2	-2266	-2256	-	4.48	GGAAATCTCG
SFTA2	-2824	-2814	-	5.03	GGAGACTCCC
SFTA2	-2803	-2793	-	5.03	GGAGACTCCC
SFTA2	-2782	-2772	-	5.03	GGAGACTCCC
SFTA2	-2740	-2730	-	5.03	GGAGACTCCC
SFTA2	-2698	-2688	-	5.03	GGAGACTCCC
SFTA2	-2656	-2646	-	5.03	GGAGACTCCC
SFTA2	-2614	-2604	-	5.03	GGAGACTCCC
SFTA2	-2593	-2583	-	5.03	GGAGACTCCC
SFTA2	-2551	-2541	-	5.03	GGAGACTCCC
SFTA2	-2509	-2499	-	5.03	GGAGACTCCC
SFTA2	-2467	-2457	-	5.03	GGAGACTCCC
SFTA2	-2425	-2415	-	5.03	GGAGACTCCC
SFTA2	-2404	-2394	-	5.03	GGAGACTCCC
SFTA2	-2362	-2352	-	5.03	GGAGACTCCC

SFTA2	-2320	-2310	-	5.03	GGAGACTCCC
SFTA2	-2278	-2268	-	5.03	GGAGACTCCC
SFTA2	-2236	-2226	-	5.03	GGAGACTCCC
SFTA2	-2194	-2184	-	5.03	GGAGACTCCC
SFTA2	-2152	-2142	-	5.03	GGAGACTCCC
SFTA2	-2131	-2121	-	5.03	GGAGACTCCC
SFTA2	-2110	-2100	-	5.03	GGAGACTCCC
DAP	-2517	-2507	-	4.74	GCAAAGTCCC
DAP	-2328	-2318	-	5.49	AGAAATCCCC
CCT5	-2852	-2842	-	4.29	GGAAAGCACC
FAM173B	-2686	-2676	+	4.69	GGGGGTTGCC

5 MA0136.1 ELF5 8.69309550562 0.429292928841 -1166:-919
1.3954916116e-05

list of hits

INPP5J	-0969	-960	+	6.08	CACTTCCAT
INPP5J	-0924	-915	+	6.97	CACTTCCTC
OSBP2	-1151	-1142	-	6.12	AAGGATCTT
OSBP2	-1023	-1014	-	6.08	ATGGAAGTG
PDZRN3	-1158	-1149	-	6.82	AAGGAAAAA
PPP4R2	-1150	-1141	+	6.49	TCCTTCCTT
PPP4R2	-1093	-1084	+	6.48	ATCTTCCTT
PPP4R2	-0919	-910	+	6.21	GACTTCCTG
GXYLT2	-1008	-999	-	6.25	GAGGATGTG
VARS2	-1166	-1157	+	6.13	CATTTCCTA
SFTA2	-1101	-1092	-	7.15	AAGGAAACA
_GTF2H4	-1115	-1106	+	6.03	CTTTTCCTT
ARL15	-1100	-1091	-	6.49	AAGGAAGGA
ARL15	-1060	-1051	-	6.51	CAGGAAACA
ARL15	-1019	-1010	-	6.37	ATGGAAGTT
CCT5	-0979	-970	+	6.31	ATTTTCCTT
CCT5	-0955	-946	+	7.05	CATATCCTT
CCT5	-0919	-910	-	6.48	AAGGAAGAT
FAM173B	-1093	-1084	+	6.98	TTCTTCCTT
FAM173B	-1084	-1075	-	6.59	AAGGATGCA

6 MA0081.1 SPIB 9.06007130057 0.466472323191 -2082:-1816
1.41313176681e-05

list of hits

LOC102151104	-2072	-2065	-	7.82	TTCCTCT
LOC102151104	-1832	-1825	-	7.28	TTCCTCA
INPP5J	-1921	-1914	-	6.60	TTCCCCT
SELM	-2082	-2075	+	7.28	TGAGGAA
SMTN	-2074	-2067	-	7.28	TTCCTCA
OSBP2	-1940	-1933	-	6.55	TTCCTGA
CNTN3	-1830	-1823	+	7.09	ACAGGAA
CNTN3	-1817	-1810	+	7.82	AGAGGAA
PPP4R2	-2010	-2003	-	7.82	TTCCTCT
PPP4R2	-1974	-1967	-	7.82	TTCCTCT
GXYLT2	-1985	-1978	+	6.60	AGGGGAA
GXYLT2	-1964	-1957	+	6.60	AGGGGAA
GXYLT2	-1888	-1881	+	6.55	TCAGGAA
_GTF2H4	-2014	-2007	-	6.55	TTCCTGA
_GTF2H4	-1816	-1809	+	7.28	TGAGGAA
ARL15	-1834	-1827	-	6.60	TTCCCCT
DAP	-1832	-1825	+	6.60	AGGGGAA
CCT5	-2077	-2070	-	7.09	TTCCTGT
CCT5	-2033	-2026	-	7.82	TTCCTCT

7 MA0114.1 HNF4A 9.61741431707 0.522388060843 -2633:-2545
1.925686689e-05

list of hits

INPP5J	-2581	-2568	+	5.87	GAGCCAAAGGGCC
SELM	-2545	-2532	-	6.24	TGGTTTTTGGTCT
SMTN	-2627	-2614	-	6.89	AAAACTTTGCCCG
SMTN	-2604	-2591	+	9.80	AGGGCAAAGACCA
SMTN	-2574	-2561	-	5.72	TGACCCCAGGCCT
PPP4R2	-2633	-2620	+	6.42	GGTCCAGAGCCCA
PPP4R2	-2552	-2539	-	6.57	TTCACTCTGGCCT

```
                _GTF2H4 -2596   -2583   +       6.22    AGGTCATAGTCCC
                _GTF2H4 -2573   -2560   -       6.05    TGGCCTTTGTAGC
                ARL15   -2560   -2547   -       5.68    TGGCCTCTTTCCT

    8   MA0154.1        EBF1    11.5644748683   0.648000004888  -2625:-1923
 2.12523767164e-05
                list of hits
                LOC102151104    -2178   -2168   +       6.13    CCTCCTGGGG
                INPP5J  -2386   -2376   +       6.63    CTCCCTGGGA
                INPP5J  -2155   -2145   -       5.68    CCCCCTGGAG
                INPP5J  -2118   -2108   +       5.87    CCTCCGGAGA
                INPP5J  -2102   -2092   +       6.70    TCTCCAGGGG
                INPP5J  -2084   -2074   -       5.57    TCCCTGGCAG
                SELM    -2087   -2077   -       6.07    TCTCCTGAGG
                OSBP2   -2625   -2615   +       6.40    CCCCAAGAGG
                OSBP2   -2545   -2535   +       8.04    ACCCCAGAGA
                OSBP2   -2019   -2009   +       5.88    CCACCTGGGA
                CNTN3   -2581   -2571   +       7.03    ACTCAGGGGG
                CNTN3   -2199   -2189   +       6.50    GCTCATGGGA
                PDZRN3  -2462   -2452   -       6.64    TCCCCAAGGG
                PPP4R2  -2338   -2328   -       7.78    TCCCAGGAGG
                PPP4R2  -2273   -2263   +       6.84    CTCCAAGGGG
                PPP4R2  -1972   -1962   +       7.70    CCTCTGGGGA
                GXYLT2  -2546   -2536   -       8.19    CCCCTTGAGT
                GXYLT2  -1990   -1980   +       6.01    ATTCCAGGGG
                GXYLT2  -1968   -1958   +       6.24    TTCCAGGGGA
                GXYLT2  -1926   -1916   +       6.41    ATCAAAGGGA
                VARS2   -2491   -2481   +       5.40    GTTCTAGGGA
                VARS2   -2389   -2379   -       7.74    TCCCTTGTGG
                SFTA2   -2608   -2598   -       6.60    TCCCTGAGAT
                SFTA2   -2587   -2577   -       6.90    TCCCTGAGGC
                SFTA2   -2419   -2409   -       6.60    TCCCTGAGAT
                SFTA2   -2398   -2388   -       6.90    TCCCTGAGGC
                SFTA2   -2146   -2136   -       6.60    TCCCTGAGAT
                SFTA2   -2125   -2115   -       6.90    TCCCTGAGGC
                _GTF2H4 -1923   -1913   -       6.55    TCCCCAGGAG
                DAP     -2608   -2598   +       8.18    ACCACAGGGA
                DAP     -2415   -2405   -       6.29    CCCCTTGTGT
                DAP     -2268   -2258   -       6.05    CCCCTAGGAG
                CCT5    -2512   -2502   -       5.89    TCCCTAAAGA
                FAM173B -2364   -2354   +       6.89    CCCCAGGAGA
                FAM173B -1956   -1946   -       5.39    TCCCTTTAAG

    9   MA0144.1        Stat3   13.6006727062   0.505057099889  -2251:-1562
 2.88021019304e-05
                list of hits
                LOC102151104    -1715   -1705   -       6.18    CTTCCTAGGA
                LOC102151104    -1674   -1664   -       4.87    CTTCCCGTGA
                LOC102151104    -1624   -1614   -       7.13    CTTCCTGACA
                LOC102151104    -1562   -1552   -       7.47    ATTCCTGGCA
                INPP5J  -2245   -2235   -       7.67    CTTCCTGGCC
                INPP5J  -2208   -2198   -       7.01    TTTCCAGGAG
                INPP5J  -2085   -2075   -       6.48    CTCCCTGGCA
                INPP5J  -1562   -1552   +       6.58    TGACGGGAAA
                SELM    -2234   -2224   -       5.60    CTTCTAGGCA
                SMTN    -2135   -2125   -       7.76    CTGCCAGGAA
                SMTN    -1840   -1830   -       5.25    CTTCCTGGGC
                SMTN    -1624   -1614   +       5.62    TTACAGTAAG
                OSBP2   -2109   -2099   -       4.62    CTTCCAAGCC
                OSBP2   -1951   -1941   -       5.03    CTTCCTAACA
                OSBP2   -1941   -1931   -       5.11    CTTCCTGAAG
                OSBP2   -1601   -1591   +       4.99    CCCCAGGAAG
                PDZRN3  -2208   -2198   +       5.34    TTCTAGGAGG
                PDZRN3  -1804   -1794   -       7.47    ATTCCTGGCA
                PPP4R2  -1565   -1555   -       7.31    CTTACAGGAA
                VARS2   -1742   -1732   +       4.97    TTACAGAAAG
                SFTA2   -1863   -1853   -       6.00    TTTCCAGGCC
                SFTA2   -1707   -1697   +       8.28    TCCCAGGAAG
                SFTA2   -1637   -1627   -       6.07    CTTCCCAGAC
```

_GTF2H4 -2214 -2204 + 6.75 AGCCAGGAAA
_GTF2H4 -2015 -2005 - 5.11 CTTCCTGAAG
_GTF2H4 -2001 -1991 - 7.85 GTTCCAGGAA
_GTF2H4 -1936 -1926 - 7.73 CTTCCAGGAG
ARL15 -1992 -1982 - 6.98 TTTCCTTGAA
ARL15 -1698 -1688 + 6.03 TTCATGGAAA
DAP -2251 -2241 + 4.86 GGCTAGGAAA
DAP -2020 -2010 - 5.39 CTTTCAGGCA
CCT5 -2078 -2068 - 4.92 TTTCCTGTGA
FAM173B -1595 -1585 - 7.35 TTTCCTGTCA

10 MA0133.1 BRCA1 5.26063524804 0.528239204415 -1431:-666
3.64914061249e-05
list of hits
LOC102151104 -1021 -1014 + 6.30 ACAACAC
INPP5J -0673 -666 - 5.61 CTGTTGG
INPP5J -0666 -659 + 5.61 CCAACAG
SMTN -0932 -925 - 5.62 CGGTTGT
SMTN -0706 -699 - 6.30 GTGTTGT
OSBP2 -1431 -1424 + 5.92 ACAACAG
OSBP2 -1192 -1185 + 5.92 ACAACAG
OSBP2 -1055 -1048 - 5.92 CTGTTGT
CNTN3 -0794 -787 + 5.61 CCAACAG
CNTN3 -0705 -698 + 5.61 CCAACAG
PDZRN3 -1344 -1337 + 5.99 CCAACAC
PDZRN3 -1220 -1213 - 5.90 GTGTTGC
PPP4R2 -0872 -865 + 5.62 ACAACCG
GXYLT2 -1413 -1406 + 6.00 ACAACCC
GXYLT2 -1338 -1331 - 6.00 GGGTTGT
GXYLT2 -0884 -877 - 6.00 GGGTTGT
VARS2 -1319 -1312 + 5.92 ACAACAG
VARS2 -1289 -1282 + 5.92 ACAACAG
VARS2 -1025 -1018 - 6.00 GGGTTGT
VARS2 -0966 -959 - 5.90 GTGTTGC
SFTA2 -1118 -1111 - 6.00 GGGTTGT
ARL15 -1066 -1059 + 5.69 CCAACCC
DAP -0808 -801 - 6.30 GTGTTGT
CCT5 -1248 -1241 + 6.00 ACAACCC
CCT5 -0731 -724 + 5.69 CCAACCC
FAM173B -0858 -851 + 6.30 ACAACAC

11 MA0095.1 YY1 8.10118848971 0.509803925951 -1115:-909
4.13334442794e-05
list of hits
INPP5J -1115 -1109 + 6.42 ACCATC
INPP5J -1109 -1103 + 6.42 ACCATC
INPP5J -1049 -1043 + 6.42 ACCATC
INPP5J -1030 -1024 + 6.42 ACCATC
INPP5J -1000 -994 + 6.42 ACCATC
INPP5J -0988 -982 + 6.42 ACCATC
INPP5J -0909 -903 + 6.42 ACCATC
OSBP2 -0925 -919 - 6.42 GATGGT
VARS2 -0992 -986 + 6.58 GCCATC
VARS2 -0954 -948 + 6.42 ACCATC
DAP -1024 -1018 - 6.42 GATGGT
CCT5 -0932 -926 + 6.42 ACCATC

12 MA0014.1 Pax5 12.4319606142 0.575000002235 -2812:-2490
5.48442454772e-05
list of hits
LOC102151104 -2616 -2596 - 5.51 TTTTCACTCTTCACATTTTA
INPP5J -2717 -2697 + 6.84 AGCGAAAGGAAGGAGGACAG
INPP5J -2683 -2663 - 5.02 TTGCCATCCACAGATGCCTC
INPP5J -2493 -2473 - 7.59 CCTCTCTGCTTCCCAGGGCA
OSBP2 -2806 -2786 - 5.32 CTTCTCCCCTCCAAGAACCA
OSBP2 -2619 -2599 + 5.57 GAGGAAGTGCAGAGAGGCCT
OSBP2 -2548 -2528 + 5.59 GGCACCCCAGAGAGGGGCAG
CNTN3 -2588 -2568 - 5.56 TTGTCACACTCAGGGGGTTT
PPP4R2 -2790 -2770 + 5.97 TGTCTATTGCTGTGTAACAA

```
                PPP4R2  -2680   -2660   -       5.05    TGGTCCCTCCAAGGGGCCCG
                VARS2   -2704   -2684   +       5.29    GAATGAACAAAGAAGGACCA
                _GTF2H4 -2812   -2792   -       5.08    CTGCTCCTCTCTGGTTTGTT
                _GTF2H4 -2631   -2611   -       8.48    CTTTTATGCTCCGCTGGCCT
                _GTF2H4 -2503   -2483   -       5.34    TTGTCCTTCAGCAGTCTTCC
                DAP     -2755   -2735   -       5.41    TTGTCACACTTGGCCACACT
                DAP     -2490   -2470   -       5.99    CTGCTCTGCCACCATTGTCA
                FAM173B -2617   -2597   +       6.91    GGGCCGCTGAAGTGGAGCAT

    13  MA0122.1        Nkx3-2  8.54224970754   0.462962961859  -452:-264
 5.88545819185e-05
                list of hits
                LOC102151104    -0266   -257    +       7.13    TTGAGTGGG
                SMTN    -0452   -443    -       6.74    GTCACTTAG
                SMTN    -0264   -255    -       7.28    GCCACTTAC
                PPP4R2  -0381   -372    -       6.66    CTCACTTAA
                GXYLT2  -0435   -426    +       6.33    GTGAGTGGG
                GXYLT2  -0356   -347    +       6.11    TGGAGTGGG
                GXYLT2  -0344   -335    +       6.44    GTAAGTGAA
                GXYLT2  -0303   -294    +       6.44    CGGAGTGGA
                VARS2   -0402   -393    +       6.29    GCAAGTGGA
                SFTA2   -0430   -421    +       6.53    CTGAGTGCC
                CCT5    -0310   -301    +       6.51    GGAAGTGGA
                FAM173B -0323   -314    -       7.09    TCCACTTGA

    14  MA0155.1        INSM1   14.8617070481   0.666666670392  -2165:-1951
 8.86960308607e-05
                list of hits
                INPP5J  -2165   -2153   -       4.88    CACCCCCTCACC
                INPP5J  -2087   -2075   -       4.79    TTCTCCCTGGCA
                INPP5J  -1972   -1960   -       4.30    CGCCCCCTGCTC
                SMTN    -2141   -2129   -       4.69    GTCCCCCTGCCA
                OSBP2   -2158   -2146   +       7.26    GGCCAGGGGCCT
                CNTN3   -1951   -1939   +       8.98    TTTCTGGGGGAA
                GXYLT2  -2109   -2097   +       5.00    ATTTTGGGGGTA
                GXYLT2  -1989   -1977   +       3.60    TTCCAGGGGAAA
                GXYLT2  -1968   -1956   +       3.78    TTCCAGGGGAAG
                VARS2   -2094   -2082   +       5.99    GGGTTGGGGGAA
                VARS2   -1996   -1984   +       7.76    GGGCTGGGGGAG
                _GTF2H4 -2140   -2128   +       4.32    TGCCTGAGGGAG
                _GTF2H4 -2066   -2054   +       5.34    TGTCAGGGAGCT
                _GTF2H4 -1956   -1944   -       3.92    TGCCCTCATCCA
                CCT5    -1974   -1962   +       3.89    TGCCAGGGATCT
                FAM173B -2127   -2115   +       7.04    GTTCTGGGGCCA

    15  MA0057.1        MZF1_5-13       9.40029161122   0.5875  70:259  9.71047928849e-05
                list of hits
                LOC102151104    +0079   89      -       6.01    CCCCCCCTCT
                INPP5J  +0189   199     -       6.11    ATGCCCCTCC
                SELM    +0259   269     -       6.68    CCACCCCTCA
                PPP4R2  +0116   126     +       8.83    GGAGGGGGAG
                PPP4R2  +0177   187     +       6.91    GGAGGCGGAG
                PPP4R2  +0256   266     -       7.24    GCCCCCCTCC
                VARS2   +0163   173     +       6.16    GGAGTGGGAG
                SFTA2   +0079   89      -       7.64    TCTCCCCTAC
                SFTA2   +0156   166     -       8.55    TTTCCCCTCC
                _GTF2H4 +0070   80      -       6.14    CTCCCCATCT
                _GTF2H4 +0103   113     -       6.64    TTTCCCCTAT
                _GTF2H4 +0174   184     +       8.06    CGAGGGGGGA
                _GTF2H4 +0191   201     +       6.16    GGAGTGGTAA
                _GTF2H4 +0213   223     +       6.92    AGAGGGGGAG
                DAP     +0137   147     +       7.31    CGAGGGGAAG
                DAP     +0246   256     +       6.16    GGCGGGGGAG
                CCT5    +0225   235     +       6.40    TGAGGGGAGG
                FAM173B +0132   142     +       6.16    GGAGTGGGAG
```
