# Supplementary material for: Identification and Functional Annotation of Genes Related to Horses’ Performance: From GWAS to Post-GWAS
Source: Animals (Basel). 2020 Jul 10;10(7):1173. doi: 10.3390/ani10071173 (PMC7401650; doi:10.3390/ani10071173)
Supplement: Supplementary file 1 [file animals-10-01173-s001.zip › supplementarymaterials/TFM_explorer_out/tfmexplorer_group7.pdf]

```
;TFM Explorer
;Date               : Wed May 13 06:35:24 2020
;Scanned location   : -3000:300
;Scanned sequences  : None (12 sequences)
;Scanned matrices   : (130 matrices)
;Parameters         : minsize= 30, maxsize= 1500, ratio=3.0, top=25
;
; headers
;    1    rank              window rank
;    2    matrix            matrix name
;    3    tf                transcription factor name
;    4    info              information of content of the matrix
;    5    gc                gc content of the matrix (G+C)%
;    6    location          location of the window (relatively to TSS)
;    7    pvalue            pvalue of the window

   1   MA0052.1        MEF2A   15.709056118    0.179310347063  -1947:-1831
1.94083402159e-07
               list of hits
               OPCML   -1900   -1890   +       6.18    ATATTTTTAA
               IGSF9B  -1894   -1884   +       4.70    TTGTTTATAG
               JAM3    -1935   -1925   +       4.89    CTCTTTTAAG
               JAM3    -1895   -1885   -       5.13    CTAGAAATAA
               NTM     -1859   -1849   -       4.68    CTATAAATCA
               CCDC90B -1940   -1930   -       3.99    TTAAAAAAAG
               CCDC90B -1833   -1823   +       6.44    TTATTAATAA
               ANKRD42 -1834   -1824   +       8.02    TTATTTTTAA
               PCF11   -1873   -1863   +       6.33    TTATATTTAA
               RAB30   -1946   -1936   +       5.73    TTATATAAAG
               RAB30   -1862   -1852   -       10.11   CTAAAAATAA
               RAB30   -1831   -1821   -       4.56    CTAAATGTAA
               PRCP    -1848   -1838   +       5.58    CTATATATGA
               DDIAS   -1947   -1937   +       3.92    CTACATATAT
               DDIAS   -1905   -1895   +       4.48    CTCTTAATAA

   2   MA0060.1        NFYA    12.9251246242   0.522629311625  -70:18  1.19747437135e-06
               list of hits
               IGSF9B  -0070   -54     +       9.19    CAGAGCCAATCGGCCC
               IGSF9B  -0040   -24     +       7.71    AGTGCCCAATGGGCGG
               NTM     -0065   -49     -       5.40    AGCCTGATTTGCCCAG
               NTM     +0018   34      +       6.56    TAGAACCAATGATTGC
               PCF11   -0062   -46     +       8.09    GCGAGCCAATCACGCC
               PRCP    -0055   -39     +       5.68    GCCCTCCAATCGAATC
               PRCP    +0007   23      +       10.72   CAGAGCCAATCAGAAG
               DDIAS   -0048   -32     -       10.72   CTTCTGATTGGCTCTG
               DDIAS   +0014   30      -       5.68    GATTCGATTGGAGGGC

   3   MA0025.1        NFIL3   14.13850704     0.264822136611  -2024:-1926
2.47494841433e-06
               list of hits
               OPCML   -2004   -1993   -       4.68    ACCTTGCATAA
               OPCML   -1992   -1981   +       4.82    TTACCTAACCT
               OPCML   -1945   -1934   -       7.08    TAATTACATAT
               NCAPD3  -1926   -1915   -       4.54    CTGGTACATAA
               CCDC90B -2024   -2013   +       4.81    TTTTATAATAG
               RAB30   -1949   -1938   -       7.33    TGCTTATATAA
               PRCP    -2003   -1992   -       4.20    CGGTTACAGAA
               PRCP    -1937   -1926   +       5.07    ATATATAACTA
               DDIAS   -1950   -1939   -       4.58    TGGCTACATAT

   4   MA0148.1        FOXA1   12.5328023695   0.332119902252  -2657:-2417
8.08292959748e-06
               list of hits
               OPCML   -2420   -2409   -       5.97    CACAGCACACA
               SPATA9  -2547   -2536   -       5.61    TAAAATAAATA
               IGSF9B  -2630   -2619   +       6.40    TGTTTTTTCAG
               IGSF9B  -2551   -2540   -       5.25    CGATGTAAATA
               JAM3    -2522   -2511   +       5.26    TGATTGCTCAG
```

NCAPD3	-2626	-2615	-	5.73	CTCAGCACACA
CCDC90B	-2657	-2646	-	6.28	CTGAGCTAACA
CCDC90B	-2605	-2594	-	6.28	CTGAGCTAACA
CCDC90B	-2430	-2419	+	5.23	TCTTTACATTC
ANKRD42	-2621	-2610	+	5.98	TGTTGACCCAC
PCF11	-2645	-2634	-	5.50	TAGAATAAATA
PCF11	-2611	-2600	-	9.55	ATGTGCAAACA
PCF11	-2535	-2524	-	7.86	ATGTGTAAATA
PCF11	-2500	-2489	-	7.27	TAGTGTAAATA
PCF11	-2477	-2466	+	7.86	TATTTACACAT
PCF11	-2456	-2445	+	9.41	TGTTTACATAC
RAB30	-2595	-2584	-	5.67	AAATACAAATA
RAB30	-2417	-2406	-	6.78	TAATACAAACA
PRCP	-2580	-2569	+	6.95	TGTTTGTTCAA
PRCP	-2554	-2543	+	7.97	TATTTGCATTT

5 MA0063.1 Nkx2-5 8.26972553332 0.218487398433 -1503:-942 1.45146299507e-05

list of hits

OPCML	-1270	-1263	+	6.06	TTAATTT
OPCML	-1230	-1223	+	5.99	TTAAGAG
OPCML	-1078	-1071	+	7.10	ATAATTG
OPCML	-1045	-1038	+	6.06	TTAATTT
SPATA9	-1503	-1496	-	6.10	CTATTAT
SPATA9	-1152	-1145	+	6.06	TTAATTT
SPATA9	-0947	-940	+	6.35	TTAATAG
IGSF9B	-1412	-1405	+	7.00	TTAAGTG
IGSF9B	-1305	-1298	+	5.99	TTAAGAG
JAM3	-1249	-1242	+	6.06	TTAATTT
NCAPD3	-1374	-1367	+	6.06	TTAATTT
NTM	-0942	-935	+	5.93	ACAATTG
CCDC90B	-1152	-1145	+	6.06	TTAATTT
CCDC90B	-1020	-1013	-	6.06	AAATTAA
ANKRD42	-1393	-1386	+	6.75	ATAAGTG
ANKRD42	-1371	-1364	+	6.06	TTAATTT
PCF11	-1465	-1458	+	6.06	TTAATTT
PCF11	-1449	-1442	-	6.10	CTATTAT
PCF11	-1240	-1233	-	7.10	CAATTAT
PCF11	-1146	-1139	+	7.00	TTAAGTG
PCF11	-0994	-987	+	6.06	TTAATTT
RAB30	-1372	-1365	+	6.06	TTAATTT
RAB30	-1081	-1074	-	6.06	AAATTAA
PRCP	-1326	-1319	+	7.00	TTAAGTG

6 MA0029.1 Evi1 17.9085404291 0.280423281291 -1597:-1170 2.01428854603e-05

list of hits

SPATA9	-1429	-1415	+	2.64	GAAATAACATAAGA
SPATA9	-1399	-1385	+	3.50	ATGAAAATATAATT
SPATA9	-1258	-1244	+	7.35	AAGAAAAGACCAAA
SPATA9	-1170	-1156	-	5.34	TGTTTTCTTATATA
JAM3	-1551	-1537	-	3.62	TGTGATCTTGTTCT
JAM3	-1407	-1393	-	4.65	CATTATCTCATGTT
JAM3	-1307	-1293	+	2.81	GACAAAAGAGAAGA
JAM3	-1242	-1228	-	2.29	CTCTATTTTGTGTT
NCAPD3	-1202	-1188	+	5.56	GATATAAGATACTA
CCDC90B	-1182	-1168	-	4.31	TCTAATCTTTTTTT
ANKRD42	-1521	-1507	-	7.44	TTTTCTCTTATCTG
ANKRD42	-1197	-1183	+	3.67	CTGGTAAGACAAAA
PCF11	-1361	-1347	+	2.87	GAGAAAATACAAGC
PCF11	-1279	-1265	-	2.69	AACAATCTTGTACA
RAB30	-1597	-1583	-	7.59	ATTTATCTTGTGAC
RAB30	-1565	-1551	+	5.61	CACATAAGATCATT
RAB30	-1470	-1456	-	2.43	GTTTAGCCTTTCTT
PRCP	-1541	-1527	-	3.07	ATTAGTCTTGTAAC
PRCP	-1465	-1451	-	3.64	ACTGGTCTTTTCCT
PRCP	-1352	-1338	-	3.58	ATCACTCTTATCTT
PRCP	-1225	-1211	-	2.91	GTCTATCTTGTTCA

```
    7   MA0100.1        Myb     9.88327768463   0.590686273136  -1662:-587
 2.30863082472e-05
                list of hits
                OPCML   -0998   -990    +       8.53    GGCCGTTG
                IGSF9B  -1201   -1193   -       6.45    CAACAGCC
                IGSF9B  -1046   -1038   -       6.45    CAACTGTA
                IGSF9B  -0658   -650    +       6.36    CGCAGTTG
                NCAPD3  -1489   -1481   -       6.58    CAACTGCA
                NCAPD3  -1336   -1328   +       6.39    GTCAGTTG
                NTM     -1129   -1121   +       6.58    TGCAGTTG
                ANKRD42 -1121   -1113   -       6.23    CAACTGTG
                ANKRD42 -0929   -921    -       6.91    CAACTGTT
                ANKRD42 -0648   -640    +       6.50    GGCAGTTA
                PCF11   -0773   -765    +       6.45    GGCTGTTG
                RAB30   -0596   -588    -       8.47    CAACCGCC
                RAB30   -0587   -579    -       8.35    CAACTGTC
                PRCP    -1496   -1488   -       8.35    CAACTGTC
                PRCP    -0984   -976    +       6.58    TGCAGTTG
                DDIAS   -1662   -1654   +       6.32    GACTGTTG
                DDIAS   -1650   -1642   -       6.58    CAACTGCA
                DDIAS   -1473   -1465   +       6.39    GTCGGTTG

    8   MA0152.1        NFATC2  9.85861508532   0.346153842592  -1808:-1458
 2.82129056678e-05
                list of hits
                OPCML   -1790   -1783   +       7.20    TTTTCCT
                OPCML   -1682   -1675   +       6.81    ATTTCCA
                OPCML   -1621   -1614   +       8.69    TTTTCCA
                OPCML   -1494   -1487   +       6.92    TTTTCCC
                IGSF9B  -1653   -1646   +       6.42    GTTTCCA
                JAM3    -1638   -1631   -       7.20    AGGAAAA
                NCAPD3  -1790   -1783   +       8.69    TTTTCCA
                NCAPD3  -1651   -1644   -       6.37    TGGAACA
                NTM     -1466   -1459   -       7.20    AGGAAAA
                CCDC90B -1711   -1704   -       6.92    GGGAAAA
                CCDC90B -1650   -1643   -       6.42    TGGAAAC
                PCF11   -1762   -1755   +       8.69    TTTTCCA
                PCF11   -1534   -1527   -       7.20    AGGAAAA
                PCF11   -1491   -1484   -       8.69    TGGAAAA
                RAB30   -1731   -1724   +       7.20    TTTTCCT
                RAB30   -1677   -1670   +       6.81    ATTTCCA
                PRCP    -1682   -1675   +       7.20    TTTTCCT
                PRCP    -1636   -1629   +       6.81    ATTTCCA
                PRCP    -1477   -1470   +       8.69    TTTTCCA
                PRCP    -1458   -1451   +       7.20    TTTTCCT
                DDIAS   -1808   -1801   -       6.81    TGGAAAT
                DDIAS   -1555   -1548   -       6.42    TGGAAAC

    9   MA0081.1        SPIB    9.06007130057   0.466472323191  191:258 4.57438269372e-05
                list of hits
                NCAPD3  +0211   218     +       7.09    ACAGGAA
                NCAPD3  +0251   258     +       6.55    TCAGGAA
                NTM     +0210   217     +       6.55    TCAGGAA
                NTM     +0258   265     +       7.82    AGAGGAA
                ANKRD42 +0191   198     +       6.60    AGGGGAA
                ANKRD42 +0231   238     -       7.82    TTCCTCT
                PCF11   +0234   241     +       7.82    AGAGGAA
                DDIAS   +0256   263     +       7.82    AGAGGAA

    10  MA0442.1        SOX10   7.96992529398   0.386363640428  -2233:-2196
 4.95980366363e-05
                list of hits
                IGSF9B  -2233   -2227   +       6.89    CTTTGT
                JAM3    -2210   -2204   +       6.67    CATTGT
                CCDC90B -2196   -2190   -       6.89    ACAAAG
                ANKRD42 -2217   -2211   -       6.67    ACAATG
                ANKRD42 -2207   -2201   +       6.89    CTTTGT
                DDIAS   -2207   -2201   +       6.89    CTTTGT
```

```
    11  MA0080.2        SPI1    9.64024729829   0.435374151649  -2362:-2189
5.07215345463e-05
                list of hits
                SPATA9  -2339   -2332   +       6.78    AGGAAAT
                SPATA9  -2204   -2197   -       6.78    ATTTCCT
                IGSF9B  -2221   -2214   -       6.42    ACTTTCT
                NCAPD3  -2229   -2222   -       6.42    ACTTTCT
                NTM     -2362   -2355   -       8.65    ACTTCCT
                NTM     -2343   -2336   -       6.42    ACTTTCT
                NTM     -2287   -2280   -       6.45    ACTTCTT
                NTM     -2223   -2216   +       6.45    AAGAAGT
                NTM     -2201   -2194   -       6.78    ATTTCCT
                CCDC90B -2359   -2352   +       6.31    AGGAAGA
                ANKRD42 -2346   -2339   -       6.81    GCTTCCT
                ANKRD42 -2189   -2182   -       6.31    TCTTCCT
                PCF11   -2353   -2346   -       6.28    AGTTCCT
                PCF11   -2342   -2335   -       6.42    ACTTTCT

    12  MA0135.1        Lhx3    16.3541414376   0.130769234437  -1166:-798
5.19814301523e-05
                list of hits
                OPCML   -1051   -1038   -       3.42    ATAGACTTAATTT
                OPCML   -0818   -805    +       6.84    AGATTAACTAAAA
                SPATA9  -1158   -1145   -       4.54    TATTTGTTAATTT
                JAM3    -1102   -1089   +       3.53    GATTGAATTATTT
                JAM3    -1065   -1052   -       3.98    GGTAATGTAATTA
                JAM3    -0933   -920    +       3.53    TAATTTAGTATAC
                JAM3    -0888   -875    +       4.27    AAACTAAATATAC
                JAM3    -0798   -785    +       4.97    TATTTTACTAATG
                NTM     -1009   -996    +       5.88    GAATTTATTAGAA
                NTM     -0808   -795    -       3.77    AGAAGATAAATGT
                CCDC90B -1166   -1153   +       3.42    TAAATAATCATTA
                CCDC90B -1023   -1010   -       4.99    TATAAATTAATAA
                CCDC90B -0984   -971    -       9.27    AAATATTTAATGA
                CCDC90B -0838   -825    +       3.46    TGATTATTTTTCT
                ANKRD42 -1026   -1013   +       6.83    GAACTAATTATTC
                ANKRD42 -0921   -908    -       5.34    AAATAGGTAATTA
                RAB30   -1135   -1122   -       13.29   AGTTAATTAATTA
                RAB30   -1115   -1102   -       5.91    GATTTTTTAATTA
                RAB30   -1084   -1071   -       5.12    CACAAATTAAACA
                PRCP    -1141   -1128   +       4.26    GAATAAACTAACT

    13  MA0038.1        Gfi     9.47016685671   0.390566043556  -2261:-2123
8.11958586816e-05
                list of hits
                JAM3    -2137   -2127   +       6.22    TGAATCATTG
                NCAPD3  -2157   -2147   -       7.20    GGGTGATTTC
                CCDC90B -2123   -2113   +       6.12    CAAATCCCAT
                ANKRD42 -2184   -2174   -       7.51    CTCAGATTTG
                ANKRD42 -2125   -2115   -       6.92    GACTGATTTT
                PCF11   -2261   -2251   -       7.74    CAGAGATTTT
                RAB30   -2159   -2149   -       7.19    CTTAGATTTG
                DDIAS   -2252   -2242   +       6.91    CAAATCTTAG
                DDIAS   -2212   -2202   +       7.01    CAAATCTTTG
                DDIAS   -2196   -2186   -       7.10    CGGAGATTTT
```
