# Supplementary material for: Identification and Functional Annotation of Genes Related to Horses’ Performance: From GWAS to Post-GWAS
Source: Animals (Basel). 2020 Jul 10;10(7):1173. doi: 10.3390/ani10071173 (PMC7401650; doi:10.3390/ani10071173)
Supplement: Supplementary file 1 [file animals-10-01173-s001.zip › supplementarymaterials/TFM_explorer_out/tfmexplorer_group6.pdf]

```
;TFM Explorer
;Date               : Wed May 13 06:35:17 2020
;Scanned location   : -3280:0
;Scanned sequences  : None (193 sequences)
;Scanned matrices   : (130 matrices)
;Parameters         : minsize= 30, maxsize= 1500, ratio=3.0, top=25
;
; headers
;    1    rank               window rank
;    2    matrix             matrix name
;    3    tf                 transcription factor name
;    4    info               information of content of the matrix
;    5    gc                 gc content of the matrix (G+C)%
;    6    location           location of the window (relatively to TSS)
;    7    pvalue             pvalue of the window

 1   MA0003.1        TFAP2A  7.8120920509    0.800000024164  -437:-278
1.36515551547e-29
               list of hits
               DUSP5   -0416   -407    -       5.96    CCGCCCGGC
               DUSP5   -0392   -383    -       7.00    CCCGGGGGC
               DUSP5   -0374   -365    +       6.75    GCCCGAAGG
               XP.PEP1 -0378   -369    +       6.31    GCCCCGCGC
               SMC3    -0402   -393    +       6.45    GCCGCGAGG
               SMC3    -0322   -313    +       6.01    GCCCTTGGG
               SMC3    -0285   -276    +       6.15    GCCCTGCGG
               RBM20   -0402   -393    +       6.63    GCCGGCGGC
               RBM20   -0345   -336    -       5.94    GCGCCCGGC
               RBM20   -0329   -320    -       6.29    CCTGAGGGC
               ADK     -0341   -332    +       6.32    GCCAGGGGC
               KAT6B   -0329   -320    +       6.39    GCCGTCGGC
               SAMD8   -0316   -307    -       6.28    CCTCAAGGC
               COMTD1  -0373   -364    +       5.97    GCCCGCCGG
               VDAC2   -0394   -385    -       7.00    CCCGGGGGC
               VDAC2   -0301   -292    +       6.01    GCCCCGGGT
               DUPD1   -0423   -414    +       5.91    GCCAGCGGG
               LOC100068320    -0430   -421    +       6.16    GCCCCTGGC
               KLHL2   -0335   -326    +       6.99    GCCGCGGGG
               CPE     -0417   -408    +       5.94    GCCGGGCGC
               CPE     -0375   -366    -       6.34    CCCCTGGGC
               CPE     -0348   -339    +       5.97    GCCCGCCGG
               LOC106780946    -0433   -424    -       6.64    CCCGCCGGC
               LOC106780946    -0396   -387    -       6.37    GCGCCGGGC
               LOC106780946    -0369   -360    +       7.06    GCCCGCGGC
               LOC106780946    -0345   -336    -       6.87    GCTCGGGGC
               EPB41L3 -0409   -400    +       6.63    GCCGGCGGC
               EPB41L3 -0358   -349    -       6.31    GCGCGGGGC
               L3MBTL4 -0434   -425    +       6.70    GCCCTGAGC
               L3MBTL4 -0382   -373    +       6.70    GCCCTGAGC
               TMEM200C        -0410   -401    +       6.87    GCCCCGAGC
               TMEM200C        -0386   -377    +       6.98    GCCGCGGGC
               TMEM200C        -0359   -350    +       6.37    GCCCGGCGC
               TMEM200C        -0322   -313    +       6.64    GCCGGCGGG
               TMEM200C        -0309   -300    +       6.99    GCCGCGGGG
               TMEM200C        -0296   -287    +       6.98    GCCGCGGGC
               .R2E1   -0330   -321    +       6.13    GCCAGAGGG
               FOXO3   -0293   -284    +       6.98    GCCGCGGGC
               ARMC2   -0366   -357    -       6.10    CCTGCCGGC
               SES.1   -0410   -401    -       5.94    GCGCCCGGC
               CEP57L1 -0393   -384    -       6.86    CCCTCCGGC
               CEP57L1 -0360   -351    -       5.88    GCGCGCGGC
               CEP57L1 -0280   -271    +       7.06    GCCTGGGGG
               OSTM1   -0413   -404    -       5.88    GCGCGAGGC
               OSTM1   -0391   -382    +       5.97    GCCCGCCGG
               OSTM1   -0374   -365    -       6.51    GCTCCCGGC
               OSTM1   -0348   -339    +       5.88    GCCGCGCGC
               OSTM1   -0302   -293    -       6.44    GCTCGCGGC
               OSTM1   -0279   -270    +       6.31    GCCGGAAGC
```

PPIL6	-0432	-423	-	6.05	GCCTGTGGC
PPIL6	-0376	-367	+	6.39	GCCGTCGGC
PPIL6	-0363	-354	-	6.33	CCCCCTGGC
PPIL6	-0339	-330	-	6.34	CCCCTGGGC
PPIL6	-0316	-307	-	6.99	CCCCGCGGC
PPIL6	-0278	-269	+	5.89	GCCGCGCGG
SMPD2	-0427	-418	+	6.99	GCCGCGGGG
SMPD2	-0404	-395	+	6.34	GCCCAGGGG
SMPD2	-0380	-371	+	6.33	GCCAGGGGG
SMPD2	-0367	-358	-	6.39	GCCGACGGC
SMPD2	-0311	-302	+	6.05	GCCACAGGC
MICAL1	-0437	-428	+	6.99	GCCCCCGGC
MICAL1	-0319	-310	+	5.94	GCCGGGCGC
ZBTB24	-0353	-344	+	7.04	GCCGGGGGC
ZBTB24	-0304	-295	-	6.51	GCTCCCGGC
FIG4	-0348	-339	-	6.69	CCTTGGGGC
WASF1	-0412	-403	-	6.52	CCTCCCGGC
CDC40	-0434	-425	+	6.52	GCCGGGAGG
METTL24	-0405	-396	+	6.18	GCCCGACGC
METTL24	-0388	-379	+	5.92	GCCCACGGG
METTL24	-0356	-347	+	6.39	GCCCGGCGG
METTL24	-0335	-326	-	6.09	GCTGCCGGC
METTL24	-0315	-306	+	7.41	GCCCCGGGC
AK9	-0315	-306	+	6.69	GCCCCAAGG
SLC22A16	-0413	-404	+	6.14	GCCCTGCGC
SLC22A16	-0298	-289	+	6.52	GCCCGCAGC
SLC22A16	-0284	-275	-	5.88	GCGCGCGGC
RBFOX3	-0362	-353	+	6.86	GCCGGAGGG
E.GASE	-0333	-324	+	6.45	GCCCCCAGC
E.GASE	-0292	-283	+	6.98	GCCGCGGGC
C1QT.F1	-0343	-334	+	5.97	GCCCGCCGG
CA.T1	-0436	-427	+	6.86	GCCTGAGGG
CA.T1	-0285	-276	+	6.51	GCCGGGAGC
LGALS3BP	-0425	-416	+	6.81	GCCTTGGGC
LGALS3BP	-0372	-363	+	7.21	GCCCCAGGC
TIMP2	-0383	-374	+	6.51	GCCGGGAGC
TIMP2	-0357	-348	+	6.02	GCCGCCAGC
TIMP2	-0291	-282	+	6.52	GCCCGCAGC
CYTH1	-0401	-392	+	7.06	GCCTGGGGG
CYTH1	-0319	-310	-	5.96	GCGGCGGGC
CYTH1	-0305	-296	+	6.32	GCCCCGCGG
CYTH1	-0286	-277	+	6.63	GCCGGCGGC
SOCS3	-0335	-326	+	6.56	GCCGCCGGC
SOCS3	-0290	-281	-	5.96	CCGCCCGGC
SOCS3	-0279	-270	+	6.34	GCCCAGGGG
TMEM235	-0384	-375	-	5.89	CCGCGCGGC
AFMID	-0281	-272	+	5.91	GCCCACGGC
CA4	-0338	-329	+	6.33	GCCAGGGGG
Z.HIT3	-0334	-325	+	5.88	GCCGCGCGC
GG.BP2	-0351	-342	-	5.96	CCGCCCGGC
GG.BP2	-0285	-276	-	6.26	CCCCGTGGC
DHRS11	-0405	-396	-	6.37	GCGCCGGGC
DHRS11	-0373	-364	+	6.32	GCCAGGGGC
DHRS11	-0331	-322	+	6.51	GCCGGGAGC
DHRS11	-0322	-313	-	6.11	GCGTGGGGC
LHX1	-0348	-339	-	5.91	CCCCTCGGC
LHX1	-0324	-315	-	7.06	CCCCCAGGC
AATF	-0282	-273	+	6.74	GCCCGAAGC
ACACA	-0405	-396	+	6.02	GCCGCCAGC
ACACA	-0383	-374	+	6.14	GCCCAAGGG
ACACA	-0322	-313	+	5.96	GCCCGCCGC
ACACA	-0310	-301	-	5.91	CCCCTCGGC
USP32	-0408	-399	+	6.52	GCCGGGAGG
SPTB.1	-0428	-419	+	6.31	GCCCCGCGC
SPTB.1	-0320	-311	+	6.31	GCCCCGCGC
ACYP2	-0326	-317	-	6.02	GCTGGCGGC
PSME4	-0391	-382	+	6.56	GCCGCCGGC
PSME4	-0381	-372	+	6.99	GCCGCGGGG
GPR75	-0292	-283	+	6.81	GCCTTGGGC

C15H2orf73	-0403	-394	+	5.96	GCCCGCCGC
C15H2orf73	-0341	-332	-	7.06	CCCCCAGGC
C15H2orf73	-0316	-307	-	5.94	GCGCCCGGC
KC.S3	-0313	-304	-	6.95	CCTCCGGGC
GE.1	-0331	-322	-	6.57	CCCGGAGGC
SMC6	-0395	-386	-	6.31	GCTTCCGGC
SMC6	-0327	-318	-	5.97	CCGGCGGGC
VS.L1	-0279	-270	-	6.02	GCTGGAGGC
RDH14	-0296	-287	+	5.80	GCCGGTGGC
.OL10	-0377	-368	-	6.52	CCTCCCGGC
ODC1	-0319	-310	-	6.18	GCGTCGGGC
ODC1	-0284	-275	-	5.96	GCGGCGGGC
HPCAL1	-0432	-423	+	7.04	GCCGGGGGC
HPCAL1	-0415	-406	+	7.06	GCCTGGGGG
HPCAL1	-0387	-378	+	6.53	GCCCGCAGG
HPCAL1	-0358	-349	+	5.88	GCCGCGCGC
HPCAL1	-0346	-337	+	6.99	GCCCCCGGC
HPCAL1	-0333	-324	+	6.57	GCCTCCGGG
RRM2	-0368	-359	-	6.94	GCTCCGGGC
RRM2	-0356	-347	+	5.97	GCCCGCCGG
RRM2	-0309	-300	-	6.32	CCGCGGGGC
RRM2	-0288	-279	+	5.75	GCCGCTGGG
RRM2	-0278	-269	+	7.04	GCCGGGGGC
KLF11	-0434	-425	+	6.09	GCCGGCAGC
KLF11	-0396	-387	-	5.94	GCGCCCGGC
KLF11	-0339	-330	-	6.99	CCCCGCGGC
KLF11	-0298	-289	+	6.45	GCCGCGAGG
GRHL1	-0390	-381	-	6.52	CCTCCCGGC
GRHL1	-0355	-346	+	6.02	GCCGCCAGC
TAF1B	-0294	-285	-	6.99	CCCCGCGGC
ADAM17	-0384	-375	+	5.89	GCCGCGCGG
ADAM17	-0369	-360	-	6.31	GCTTCCGGC
ADAM17	-0327	-318	+	6.31	GCCGGAAGC
CYS1	-0378	-369	+	6.99	GCCCCCGGC
CYS1	-0297	-288	+	5.94	GCCGGGCGC
RPS7	-0345	-336	+	6.32	GCCCAGGGC
ADI1	-0435	-426	+	6.31	GCCCCGCGC
ADI1	-0402	-393	-	6.87	GCTCGGGGC
ADI1	-0317	-308	+	5.97	GCCCGCCGG
TRAPPC12	-0379	-370	-	6.88	CCTCGGGGC
TRAPPC12	-0329	-320	-	6.09	GCTGCCGGC
PDZR.3	-0421	-412	-	6.52	CCTCCCGGC
PPP4R2	-0417	-408	-	6.45	CCTCGCGGC
PPP4R2	-0369	-360	-	5.88	GCGCGCGGC
PPP4R2	-0319	-310	+	5.94	GCCGGGCGC
GXYLT2	-0404	-395	+	5.97	GCCCGCCGG
GXYLT2	-0393	-384	+	6.87	GCCCCGAGC
GXYLT2	-0348	-339	+	5.88	GCCGCGCGC
GXYLT2	-0317	-308	-	5.96	CCGCCCGGC
GXYLT2	-0280	-271	+	7.49	GCCCGGGGG
SHQ1	-0336	-327	-	6.11	GCGTGGGGC
SHQ1	-0289	-280	+	6.31	GCCGGAAGC
RYBP	-0435	-426	-	5.94	GCGCCCGGC
RYBP	-0317	-308	-	6.28	GCTGAGGGC
GXYLT2	-0404	-395	+	5.97	GCCCGCCGG
GXYLT2	-0393	-384	+	6.87	GCCCCGAGC
GXYLT2	-0348	-339	+	5.88	GCCGCGCGC
GXYLT2	-0317	-308	-	5.96	CCGCCCGGC
GXYLT2	-0280	-271	+	7.49	GCCCGGGGG
PROK2	-0408	-399	-	6.31	GCGCGGGGC
PROK2	-0394	-385	-	5.89	GCGGGGGGC
PROK2	-0371	-362	+	7.28	GCCCGAGGC
PROK2	-0350	-341	+	5.88	GCCGCGCGC
PROK2	-0313	-304	-	5.90	GCCGCTGGC
DIPK2A	-0422	-413	+	6.45	GCCCCCAGC
DIPK2A	-0388	-379	-	5.88	GCGCGCGGC
DIPK2A	-0356	-347	+	6.87	GCCCCGAGC
TASP1	-0333	-324	-	6.31	GCTTCCGGC
ISM1	-0371	-362	+	6.37	GCCCGGCGC

ISM1	-0341	-332	+	6.15	GCCCTGCGG
LOC100034197	-0364	-355	+	7.22	GCCCCAGGG
LOC100056127	-0338	-329	+	7.42	GCCCCGGGG
SLC31A2	-0418	-409	+	6.32	GCCCCGCGG
SLC31A2	-0357	-348	+	7.21	GCCCCAGGC
PRPF4	-0293	-284	+	6.18	GCCCCTGGG
R.F183	-0412	-403	+	6.70	GCCCTGAGC
WDR31	-0345	-336	+	6.18	GCCCCTGGG
WDR31	-0311	-302	+	6.09	GCCGGCAGC
WDR31	-0292	-283	+	6.95	GCCCGGAGG
BSPRY	-0334	-325	+	6.64	GCCGGCGGG
BSPRY	-0301	-292	+	5.90	GCCCCCCGG
HDHD3	-0356	-347	-	5.89	GCGGGGGGC
ALAD	-0396	-387	+	5.97	GCCCGCCGG
ALAD	-0362	-353	+	6.44	GCCTCGAGC
ALAD	-0301	-292	+	6.29	GCCCTCAGG
POLE3	-0404	-395	-	6.64	CCCGCCGGC
Z.F618	-0437	-428	+	7.04	GCCGGGGGC
Z.F618	-0340	-331	+	5.89	GCCCCCCGC
Z.F618	-0329	-320	+	5.89	GCCCCCCGC
Z.F618	-0314	-305	+	5.90	GCCCCCCGG
AMBP	-0326	-317	+	6.82	GCCTTGGGG
COL27A1	-0291	-282	+	5.85	GCCTTCAGC
AK.A	-0356	-347	+	6.98	GCCTCGGGC
TEX48	-0373	-364	-	6.31	GCTTCCGGC
TEX48	-0289	-280	+	6.86	GCCGGAGGG
LOC100050034	-0394	-385	+	6.24	GCCTCAAGC
DCK	-0431	-422	+	7.24	GCCCTGGGC
DCK	-0373	-364	-	5.88	GCGCGCGGC
DCK	-0353	-344	-	5.89	CCGCGCGGC
DCK	-0314	-305	+	5.94	GCCGGGCGC
DCK	-0290	-281	+	6.74	GCCCGAAGC
MOB1B	-0432	-423	-	6.32	CCGCGGGGC
MOB1B	-0373	-364	+	6.86	GCCGGAGGG
MOB1B	-0317	-308	-	5.89	GCGGGGGGC
GRSF1	-0428	-419	-	6.08	ACCCCGGGC
GRSF1	-0414	-405	+	6.34	GCCCAGGGG
GRSF1	-0400	-391	+	7.41	GCCCCGGGC
GRSF1	-0388	-379	+	6.69	GCCCCAAGG
GRSF1	-0337	-328	+	5.89	GCCTCGCGG
GRSF1	-0292	-283	-	6.52	CCTCCCGGC
RUFY3	-0426	-417	+	6.34	GCCCAGGGG
RUFY3	-0398	-389	+	6.86	GCCGGAGGG
RUFY3	-0322	-313	+	6.78	GCCGCAGGC
RUFY3	-0298	-289	+	6.09	GCCGGCAGC
UTP3	-0414	-405	+	6.45	GCCCCCAGC
Z.F572	-0285	-276	+	6.57	GCCTCCGGG
SQLE	-0380	-371	-	6.02	GCTGGCGGC
SQLE	-0352	-343	-	6.31	GCGCGGGGC
WASHC5	-0307	-298	+	6.32	GCCAGGGGC
.SMCE2	-0366	-357	+	6.16	GCCCCTGGC
PLEKHH2	-0284	-275	-	5.79	GCTCTGGGC
ABCG8	-0343	-334	-	6.09	CCCCATGGC
ABCG8	-0315	-306	-	6.09	CCCCATGGC
ABCG8	-0287	-278	-	6.09	CCCCATGGC
LRPPRC	-0342	-333	-	5.94	GCGCCCGGC
LRPPRC	-0319	-310	-	6.37	GCGCCGGGC
PPM1B	-0379	-370	+	5.84	GCCCTGGGT
KLHL29	-0427	-418	+	7.47	GCCCGGGGC
KLHL29	-0359	-350	+	6.16	GCCCCTGGC
PLCB1	-0400	-391	+	6.57	GCCGCCGGG
PLCB1	-0349	-340	+	5.79	GCCAGGAGG
TMX4	-0355	-346	-	6.08	ACCCCGGGC
COX20	-0431	-422	+	7.47	GCCCGGGGC
COX20	-0412	-403	+	6.32	GCCGGAAGG
COX20	-0386	-377	+	7.21	GCCCCAGGC
COX20	-0335	-326	+	7.06	GCCCGCGGC
CATSPERE	-0311	-302	+	6.99	GCCGCGGGG
CATSPERE	-0295	-286	-	5.88	GCGCGCGGC

ZBTB18	-0424	-415	+	6.99	GCCCCCGGC
ZBTB18	-0343	-334	+	6.09	GCCATGGGG
ZBTB18	-0289	-280	+	5.91	GCCAGCGGG
ADSS2	-0395	-386	+	6.99	GCCGCGGGG
ADSS2	-0371	-362	-	6.64	CCCGCCGGC
ADSS2	-0348	-339	-	6.53	CCTGCGGGC
ADSS2	-0319	-310	-	5.88	GCGCGAGGC
ADSS2	-0280	-271	-	6.31	GCGCGGGGC
DESI2	-0419	-410	-	6.79	CCCTGCGGC
H.R.PU	-0415	-406	+	5.89	GCCGCGCGG
H.R.PU	-0398	-389	+	6.37	GCCCGGCGC
H.R.PU	-0379	-370	+	5.94	GCCGGGCGC
H.R.PU	-0364	-355	-	6.51	GCTCCCGGC

2 MA0162.1 Egr1 14.4555832005 0.739393945445 -503:-268
9.58601738119e-24

list of hits

DUSP5	-0392	-381	+	7.98	CCCGGGGGCGT
ADD3	-0366	-355	-	4.55	CCTCCCACAGC
XP.PEP1	-0340	-329	+	5.44	GATGGGGGCGG
XP.PEP1	-0298	-287	+	4.46	GGCGTGGCCGG
MXI1	-0301	-290	-	6.71	ACACTCACACA
SMC3	-0421	-410	-	4.56	CCCCCTACGCT
SMC3	-0370	-359	-	7.20	CCGCCTCCACG
RBM20	-0431	-420	-	5.38	CCGCTCCCGGG
RBM20	-0278	-267	-	4.51	CCGCGCCCGCG
ADK	-0412	-401	-	4.25	CCTCCTACAGT
ADK	-0401	-390	+	5.96	CGGGTGGGCGG
ADK	-0330	-319	+	7.00	TGTGTGGGAGA
COMTD1	-0324	-313	-	4.38	CCGCCGCCGCT
VDAC2	-0492	-481	-	4.58	CCGCCCCCTCG
VDAC2	-0425	-414	-	4.15	CGGCCCCCGCT
VDAC2	-0394	-383	+	7.98	CCCGGGGGCGG
LOC100061588	-0471	-460	-	5.32	CCACCCACTCA
LOC100068208	-0500	-489	+	6.02	GGTGGGGGTGG
MSMO1	-0472	-461	-	6.60	CCGCCTCCGGG
KLHL2	-0333	-322	+	4.44	CGCGGGGGCCG
TMEM192	-0270	-259	+	8.29	AGTGGGGGCGG
APELA	-0362	-351	+	5.32	AGTGGAGGAGG
APELA	-0343	-332	+	5.03	GCCGGGGGAGG
LOC100066472	-0377	-366	+	5.62	GGTGTAGGAGT
LOC100068041	-0442	-431	+	4.92	TGAGTGGGAGG
LOC106780946	-0478	-467	-	4.38	CCGCCGCCGCT
LOC106780946	-0366	-355	+	4.66	CGCGGCGGCGG
EPB41L3	-0473	-462	+	4.58	TGTGTGCGCGT
EPB41L3	-0395	-384	+	6.47	GGCGGGAGCGG
EPB41L3	-0366	-355	-	4.44	CCGCCCCGGCG
TMEM200C	-0391	-380	-	4.66	CCGCCGCCGCG
TMEM200C	-0279	-268	+	4.38	AGCGGCGGCGG
S.X3	-0400	-389	-	7.16	CCGCCTCCGGA
AFG1L	-0328	-317	-	6.41	CCTCCCACGGC
FOXO3	-0481	-470	+	4.38	AGCGGCGGCGG
FOXO3	-0344	-333	-	7.48	CCTCCCCCGCC
FOXO3	-0328	-317	-	4.44	CCGCCCCGGCG
ARMC2	-0332	-321	+	8.86	TGCGTGGGAGA
CEP57L1	-0444	-433	-	6.37	CCGCCCAGGCA
CEP57L1	-0279	-268	+	6.13	CCTGGGGGCGT
OSTM1	-0464	-453	-	4.05	CCACCTCCGGC
PPIL6	-0440	-429	-	4.87	CCTCTCCCGCC
PPIL6	-0330	-319	-	5.44	CCGCCCCCATC
PPIL6	-0290	-279	+	10.15	AGCGGGGGCGG
SMPD2	-0455	-444	-	10.15	CCGCCCCCGCT
SMPD2	-0415	-404	+	5.44	GATGGGGGCGG
SMPD2	-0305	-294	+	4.87	GGCGGGAGAGG
MICAL1	-0448	-437	+	10.45	GGCGTGGGCGG
ZBTB24	-0314	-303	+	4.66	CGCGCAGGCGG
WASF1	-0352	-341	+	4.58	CGAGGGGGCGG
CDC40	-0496	-485	-	4.58	CCGCCCCCTCG
METTL24	-0292	-281	+	6.23	CGCGGGAGAGG

SLC22A16	-0322	-311	+	9.07	GGCGGGGGCGG
E.GASE	-0335	-324	-	4.77	CCGCCCCCAGC
CA.T1	-0502	-491	-	4.46	CCGCCTCCAGT
CA.T1	-0480	-469	-	4.11	TCACCCCCACC
CA.T1	-0317	-306	-	4.51	CCGCGCCCGCG
CA.T1	-0285	-274	+	4.02	GCCGGGAGCGT
LGALS3BP	-0402	-391	-	7.22	ACGCCCACAGT
TIMP2	-0295	-284	-	5.06	CCGCGCCCGCA
USP36	-0314	-303	+	6.14	GCTGTGGGCGT
CYTH1	-0429	-418	+	6.04	CGCGCGGGCGG
CYTH1	-0400	-389	+	6.13	CCTGGGGGCGG
CYTH1	-0329	-318	+	6.04	CGCGCGGGCGG
CYTH1	-0274	-263	+	4.44	CGCGGGGCCGG
SOCS3	-0361	-350	-	4.66	CCGCCGCCGCG
SOCS3	-0296	-285	-	7.16	TCGCCCCCGCC
TMEM235	-0286	-275	-	4.09	ACTCTCCCACT
Z.HIT3	-0293	-282	+	4.53	GGCGTGCGCGT
MYO19	-0491	-480	+	8.58	CGTGGGGGCGG
GG.BP2	-0304	-293	-	5.75	CCGCCCTCGCT
LHX1	-0327	-316	-	4.53	CCTCCCCCAGG
AATF	-0407	-396	-	4.03	ACGCGCACACG
AATF	-0339	-328	-	5.84	ACGCCCCCAGT
AATF	-0310	-299	-	4.44	CCTCCCGCGCG
ACACA	-0423	-412	-	7.46	CCTCCTCCGCG
ACACA	-0401	-390	-	4.62	CCAGCCACGCG
ACACA	-0314	-303	-	4.58	CCGCCCCCTCG
USP32	-0305	-294	+	4.61	GGTGGGAGCGG
USP32	-0281	-270	+	5.42	CATGGAGGCGG
ACYP2	-0316	-305	-	5.00	CCTCCCTCGCA
PSME4	-0363	-352	+	7.46	CGCGGAGGAGG
PSME4	-0317	-306	+	4.08	AGGGTGGGAGG
PSME4	-0269	-258	+	5.96	CGGGTGGGCGG
ERLEC1	-0365	-354	+	6.62	TCCGTAGGCGA
ERLEC1	-0288	-277	+	5.84	GGTGGAGGCGG
KC.S3	-0345	-334	+	9.24	CGCGGGGGTGG
GE.1	-0495	-484	-	6.04	CCGCCCGCGCG
SMC6	-0349	-338	-	5.88	CCGCGCACGCG
SMC6	-0316	-305	+	7.22	GGTGGGGGCGG
ODC1	-0502	-491	-	4.66	CCGCCGCCGCG
HPCAL1	-0462	-451	+	9.07	GGCGGGGGCGG
HPCAL1	-0432	-421	+	6.62	GCCGGGGGCGG
HPCAL1	-0414	-403	+	6.13	CCTGGGGGCGT
HPCAL1	-0398	-387	+	8.95	AGCGGGGGTGG
HPCAL1	-0348	-337	-	6.62	CCGCCCCCGGC
HPCAL1	-0335	-324	-	6.60	CCGCCTCCGGG
HPCAL1	-0322	-311	-	6.04	CCGCCCTCGCG
KLF11	-0386	-375	-	4.44	CCGCCCCGGCG
KLF11	-0343	-332	-	10.43	CCGCCCCCGCG
KLF11	-0271	-260	+	6.04	CGCGCGGGCGG
GRHL1	-0462	-451	-	4.68	CCGCCCGCGCC
GRHL1	-0434	-423	-	4.99	CCGCCCCGGCA
YWHAQ	-0295	-284	+	4.06	CGTGGGAGCGA
ADAM17	-0355	-344	+	6.14	GCTGTGGGCGG
CYS1	-0496	-485	-	7.70	CCGCCCCCGGT
CYS1	-0478	-467	-	4.25	CCTCCCCCAGT
CYS1	-0434	-423	-	4.25	CCTCCCCCAGT
CYS1	-0406	-395	-	7.70	CCGCCCCCGGT
CYS1	-0363	-352	-	4.16	CCTCCCGCGCT
CYS1	-0346	-335	-	4.16	CCTCCCGCGCT
ALLC	-0377	-366	+	6.94	TCCGGGGGAGG
RPS7	-0374	-363	-	5.00	ACTCCCACGAA
TRAPPC12	-0432	-421	+	5.44	GATGGGGGCGG
TRAPPC12	-0273	-262	+	10.99	TGCGGGGGCGG
PDZR.3	-0396	-385	+	4.60	GGGGTGGGCGG
PPP4R2	-0491	-480	+	9.07	GGCGGGGGCGG
PPP4R2	-0473	-462	+	9.07	GGCGGGGGCGG
PPP4R2	-0455	-444	+	7.94	TGTGGGGGTGG
PPP4R2	-0408	-397	-	7.70	ACGCCTCCGCC
GXYLT2	-0419	-408	-	4.33	CCTCCTCCGTC

GXYLT2	-0364	-353	+	4.33	GACGGAGGAGG
GXYLT2	-0268	-257	-	4.66	CCGCCGCCGCG
SHQ1	-0318	-307	-	6.25	TCTCTCACGCA
RYBP	-0498	-487	-	9.07	CCGCCCCCGCC
RYBP	-0405	-394	+	7.70	GGCGGAGGCGG
RYBP	-0360	-349	+	4.38	AGCGGCGGCGG
RYBP	-0301	-290	+	8.84	CGCGGGGGAGG
GXYLT2	-0419	-408	-	4.33	CCTCCTCCGTC
GXYLT2	-0364	-353	+	4.33	GACGGAGGAGG
GXYLT2	-0268	-257	-	4.66	CCGCCGCCGCG
PROK2	-0365	-354	+	9.07	GGCGGGGGCGT
PROK2	-0276	-265	+	5.06	TGCGGGCGCGG
DIPK2A	-0444	-433	-	4.38	CCGCCGCCGCT
DIPK2A	-0405	-394	+	5.88	CGCGTGCGCGT
TASP1	-0346	-335	+	4.18	CAGGTGGGCGT
TASP1	-0329	-318	-	4.04	CCGGCCACGTG
TASP1	-0286	-275	-	7.04	TCACCCCCGCT
ISM1	-0501	-490	-	4.68	CCGCCGACGCC
ISM1	-0455	-444	-	6.62	ACGCCCCCGGC
ISM1	-0423	-412	+	9.61	TGCGGAGGCGG
SPTLC3	-0380	-369	+	4.38	CATGTGAGTGT
ZFP37	-0311	-300	+	5.43	GCCGGGGGTGG
FKBP15	-0461	-450	-	4.30	CCGCCCCCCCT
FKBP15	-0427	-416	+	4.44	CGCAGGGGCGT
SLC31A1	-0353	-342	-	4.44	ACGCCCCTGCG
SLC31A1	-0319	-308	+	4.30	AGGGGGGGCGG
PRPF4	-0269	-258	+	5.26	AGCGGAGGAGA
ALAD	-0497	-486	-	9.39	CCTCCCCCGCA
ALAD	-0347	-336	+	12.37	TGCGTGGGCGG
RGS3	-0428	-417	-	4.34	ACACCCATGCT
POLE3	-0461	-450	-	4.02	CCGCTCCCGGC
POLE3	-0379	-368	-	8.86	TCTCCCACGCA
Z.F618	-0437	-426	+	6.62	GCCGGGGGCGG
Z.F618	-0420	-409	+	10.43	CGCGGGGGCGG
Z.F618	-0382	-371	+	4.66	CGCGGCGGCGG
Z.F618	-0293	-282	+	6.10	GGCGGAGGAGG
Z.F618	-0281	-270	+	4.38	AGCGGCGGCGG
AMBP	-0446	-435	-	5.03	CCTCCCCCGGC
COL27A1	-0273	-262	-	4.77	CCGCCCCCAGC
AK.A	-0469	-458	-	9.61	CCGCCTCCGCA
AK.A	-0457	-446	+	5.44	GATGGGGGCGT
AK.A	-0345	-334	+	4.52	TGTTTGGGCGG
ATP6V1G1	-0357	-346	-	6.10	CCTCCTCCGCC
T.FSF8	-0494	-483	-	7.40	CCACCCACACC
T.FSF8	-0451	-440	-	6.30	CCTCCCACATT
DCK	-0398	-387	-	4.66	CCGCCCCCGAG
MOB1B	-0419	-408	+	5.96	TCCGGAGGTGT
MOB1B	-0327	-316	+	5.44	GATGGGGGCGG
GRSF1	-0354	-343	-	5.78	TCGCCTCCGCC
RUFY3	-0465	-454	+	4.44	CGCCGGGGCGG
UTP3	-0500	-489	-	6.10	ACTCCCCCGGT
UTP3	-0416	-405	-	4.77	ACGCCCCCAGC
R.PC3	-0346	-335	-	5.68	ACTCCTCCGTG
COL11A1	-0385	-374	+	5.96	CGAGTGGGCGG
SQLE	-0503	-492	-	8.29	ACGCCCCCACT
SQLE	-0339	-328	+	4.60	GGGGTGGGCGG
WASHC5	-0464	-453	-	7.75	TCGCCCACACT
TRIB1	-0480	-469	-	4.58	CCGCCCCCCCG
TRIB1	-0336	-325	+	6.10	GGCGGAGGAGG
LRPPRC	-0327	-316	-	4.32	TCTCTCCCGCG
PREPL	-0468	-457	-	4.34	ACAACCACGCT
SLC3A1	-0355	-344	+	7.40	GGTGTGGGTGT
KLHL29	-0412	-401	-	5.62	CCTCCCCCACC
KLHL29	-0399	-388	-	7.88	CCACCCCCGCC
KLHL29	-0334	-323	-	5.00	CCTCCCTCGCA
PLCB1	-0373	-362	+	4.56	AGCGAGGGTGG
C25H9orf43	-0503	-492	-	7.70	CCGCCTCCGCC
COX20	-0272	-261	+	10.15	AGCGGGGGCGG
CATSPERE	-0277	-266	+	9.07	GGCGTAGGCGG

ZBTB18	-0472	-461	+	5.24	TGCGTGCGTGG
ZBTB18	-0453	-442	-	9.07	CCGCCCCCGCC
ZBTB18	-0441	-430	-	9.07	CCGCCCCCGCC
ZBTB18	-0426	-415	-	6.62	CCGCCCCCGGC
ZBTB18	-0286	-275	+	10.15	AGCGGGGGCGG
ADSS2	-0481	-470	+	5.96	CGGGTGGGCGG
ADSS2	-0441	-430	-	7.16	TCGCCCCCGCC
ADSS2	-0391	-380	+	4.58	CGGGGGGGCGT
DESI2	-0494	-483	+	4.99	TGCGGGGGCTT
DESI2	-0345	-334	-	9.06	CCGCCTCCGCG
DESI2	-0322	-311	+	4.56	AGCGGGGGGGG
H.R.PU	-0392	-381	+	10.43	CGCGGGGGCGG
H.R.PU	-0358	-347	+	9.07	GGCGGGGGCGG
H.R.PU	-0336	-325	+	6.44	TGCGTGCGCGG
H.R.PU	-0323	-312	-	4.44	CCGCCCCTGCG

3 MA0079.2 SP1 11.1288627426 0.820000010729 -398:-322
2.72407069304e-23

list of hits

DUSP5	-0368	-358	-	8.96	AGGGGCGGGG
ADD3	-0326	-316	+	5.80	CCCTGCTTCC
XP.PEP1	-0389	-379	+	8.11	CCCTGCCTCC
XP.PEP1	-0353	-343	-	9.67	GTGGGCGGGG
XP.PEP1	-0337	-327	-	11.09	GGGGGCGGGG
SMC3	-0372	-362	+	8.00	CTCCGCCTCC
ADK	-0398	-388	-	6.94	GTGGGCGGGA
KAT6B	-0392	-382	-	6.51	AGGAGAGGGG
KAT6B	-0350	-340	+	6.75	CCCCCGCCCC
SAMD8	-0351	-341	+	7.69	CCCCGCTTCC
SAMD8	-0322	-312	+	7.39	CCCTGCCCTC
VDAC2	-0391	-381	-	11.09	GGGGGCGGGG
LOC100068191	-0349	-339	+	8.11	CCCAGCCTCC
TMEM192	-0337	-327	+	9.13	CCCCTCCCTC
CPE	-0328	-318	+	6.51	CCCCTCTCCT
APELA	-0388	-378	-	6.34	CGGGAGGGGG
APELA	-0378	-368	-	8.01	GGGGGGGGAG
APELA	-0359	-349	-	9.86	GGAGGAGGGG
APELA	-0346	-336	-	6.03	GAGGCCGGGG
LOC106780946	-0363	-353	-	5.72	GGCGGCGGCG
EPB41L3	-0329	-319	-	9.28	GAGGGCGGGG
L3MBTL4	-0362	-352	+	5.66	CCCATCTTCC
TMEM200C	-0393	-383	+	5.72	CGCCGCCGCC
RIT2	-0347	-337	-	6.47	GGGGGCAGGA
SYT4	-0364	-354	-	6.64	GGGAGAGGAG
SYT4	-0344	-334	-	7.55	GGAAGAGGGG
S.X3	-0355	-345	-	5.72	GGCGGCGGCG
AFG1L	-0363	-353	+	7.97	CCCTTCCTCC
AFG1L	-0335	-325	+	8.96	CCCCGCCCCT
FOXO3	-0393	-383	-	5.72	GGCGGCGGCG
FOXO3	-0351	-341	+	7.43	CCCCGTCCCT
FOXO3	-0340	-330	+	6.90	CCCCGCCGCA
FOXO3	-0330	-320	+	8.96	CCCCGCCCCG
ARMC2	-0383	-373	+	7.54	CTCCTCCCAC
SES.1	-0372	-362	-	9.28	GAGGGCGGGG
SES.1	-0353	-343	-	9.86	GGAGGAGGGG
CEP57L1	-0398	-388	+	9.28	CCCCGCCCTC
OSTM1	-0359	-349	-	8.96	TGGGGCGGGG
OSTM1	-0338	-328	-	9.56	GGGGACGGGG
PPIL6	-0368	-358	+	11.09	CCCCGCCCCC
PPIL6	-0355	-345	+	8.96	CCCCGCCCCA
PPIL6	-0345	-335	+	6.38	TCCCACCCCC
PPIL6	-0332	-322	+	7.71	GCCCGCCCCC
SMPD2	-0389	-379	-	8.96	TGGGGCGGGG
SMPD2	-0376	-366	-	11.09	GGGGGCGGGG
MICAL1	-0342	-332	+	6.58	CCCACTCCCC
ZBTB24	-0372	-362	+	8.96	CCCCGCCCCG
ZBTB24	-0362	-352	+	8.96	CCCCGCCCCG
FIG4	-0363	-353	+	9.09	CTCCGCCCCC
FIG4	-0345	-335	-	8.96	TGGGGCGGGG

FIG4	-0327	-317	-	8.02	GGAGGTGGGG
WASF1	-0349	-339	-	11.09	GGGGGCGGGG
METTL24	-0393	-383	+	5.71	CCCCGGCCCA
AK9	-0337	-327	+	8.02	CCCCACCTCC
SLC22A16	-0373	-363	-	5.97	AGAGGCTGGG
SLC22A16	-0363	-353	-	10.00	GGGGGGGGGG
SLC22A16	-0348	-338	-	6.90	CGCGGCGGGG
SLC22A16	-0325	-315	-	6.90	AGCGGCGGGG
E.GASE	-0392	-382	+	5.95	CCCAGGCCCC
E.GASE	-0368	-358	+	9.28	CCCCGCCCTC
E.GASE	-0352	-342	+	8.96	CCCCGCCCCA
E.GASE	-0337	-327	+	11.09	CCCCGCCCCC
E.GASE	-0325	-315	+	7.97	CCCTTCCTCC
C1QT.F1	-0369	-359	-	6.47	GGAGGAGGGC
CA.T1	-0376	-366	+	7.86	CTCCTCCTCC
LGALS3BP	-0383	-373	-	5.83	AGAGGAAGGG
TIMP2	-0395	-385	-	8.81	CGGGGAGGGG
TIMP2	-0343	-333	-	8.00	GGAGGCGGAG
USP36	-0350	-340	-	7.43	CGGGACGGGG
CYTH1	-0397	-387	-	9.09	GGGGGCGGAG
CYTH1	-0323	-313	-	5.72	GGCGGCGGCG
SOCS3	-0375	-365	+	5.72	CGCCGCCGCC
SOCS3	-0363	-353	+	5.72	CGCCGCCGCC
SOCS3	-0349	-339	+	5.84	CTCCGGCCCC
SOCS3	-0322	-312	+	6.89	CCCAGCTCCC
TMEM235	-0374	-364	+	8.78	CCCCGCTCCC
TMEM235	-0361	-351	+	6.89	CCCTGCTCCC
TMEM235	-0347	-337	+	7.87	CCCCGCCTCT
TMEM235	-0331	-321	+	7.25	CCCCGTTCCC
CA4	-0388	-378	-	6.79	GGGAGCGGAG
CA4	-0355	-345	-	7.72	AGAGGAGGGG
CA4	-0334	-324	-	8.95	GGGGGAGGAG
MYO19	-0391	-381	-	5.73	AGAGGAGGAG
GG.BP2	-0363	-353	-	10.00	GGAGGCGGGG
GG.BP2	-0334	-324	+	7.00	CCCCTCCCTG
GG.BP2	-0324	-314	+	7.30	CCCCACCCTC
DHRS11	-0370	-360	-	8.96	AGGGGCGGGG
DHRS11	-0350	-340	-	8.96	AGGGGCGGGG
LHX1	-0372	-362	+	6.54	TCCCGCCCTC
LHX1	-0362	-352	+	6.06	CCCTCCCGCC
LHX1	-0352	-342	+	6.19	CTCCCCCCTC
LHX1	-0341	-331	+	5.82	GCCTGCCCCC
LHX1	-0330	-320	+	8.47	CCCCCTCCCC
ACACA	-0322	-312	+	5.65	GCCCGCCGCC
USP32	-0396	-386	-	6.19	GAGGGGGGAG
USP32	-0360	-350	-	7.00	AAGGGAGGGG
USP32	-0325	-315	-	9.13	GAGGGAGGGG
SPTB.1	-0360	-350	+	6.40	TCCCTCCCTC
PSME4	-0360	-350	-	6.54	GGAGGAGGCG
PSME4	-0345	-335	-	6.51	CGGAGAGGGG
PSME4	-0333	-323	-	7.84	GGGGCCGGGG
C15H2orf73	-0347	-337	+	8.36	TCCCGCCCCC
KC.S3	-0362	-352	-	8.36	GGGGGCGGGA
KC.S3	-0343	-333	-	5.98	CGGGGGTGGG
GE.1	-0376	-366	-	6.22	TGGGGCGGGA
SMC6	-0387	-377	+	7.72	CCCCTCCTCT
RDH14	-0360	-350	+	5.64	CGCCGCCCCT
.OL10	-0384	-374	+	6.92	CCCTTCCCCT
ODC1	-0363	-353	+	6.11	CTCAGCCTCC
ODC1	-0351	-341	+	5.72	CGCCGCCGCC
HPCAL1	-0396	-386	-	5.98	CGGGGGTGGG
HPCAL1	-0350	-340	+	11.09	CCCCGCCCCC
HPCAL1	-0337	-327	+	10.00	CCCCGCCTCC
HPCAL1	-0324	-314	+	6.54	TCCCGCCCTC
KLF11	-0388	-378	+	8.96	CCCCGCCCCG
KLF11	-0345	-335	+	11.09	CCCCGCCCCC
GRHL1	-0395	-385	+	6.62	GCCCGCCTCC
GRHL1	-0374	-364	+	6.08	TCCCTCCCCG
TAF1B	-0349	-339	-	7.40	CTGGGAGGGG

YWHAQ	-0398	-388	+	6.22	TCCCGCCCCG
YWHAQ	-0387	-377	+	7.37	CCCCTTCGCC
YWHAQ	-0336	-326	-	7.27	GGAGGCGGGA
CYS1	-0391	-381	+	7.40	CCCCTCCCAG
CYS1	-0376	-366	+	7.84	CCCCGGCCCC
CYS1	-0353	-343	+	6.22	TCCCGCCCCT
ALLC	-0375	-365	-	5.98	CGGGGGAGGG
ALLC	-0349	-339	+	6.49	CCCCATCTCC
COLEC11	-0327	-317	+	10.00	CCCCCCCCCC
ADI1	-0328	-318	+	9.67	CCCCGCCCAC
TRAPPC12	-0397	-387	+	9.56	CCCCGTCCCC
TRAPPC12	-0365	-355	+	9.67	CCCCGCCCAC
DCDC2C	-0380	-370	+	8.64	CCCCTCTCCC
PDZR.3	-0393	-383	-	6.94	GTGGGCGGGA
PPP4R2	-0348	-338	-	9.04	GGCGGCGGGG
GXYLT2	-0328	-318	+	9.04	CCCCGCCGCC
SHQ1	-0384	-374	+	6.44	CCCTTTCTCC
SHQ1	-0356	-346	+	5.74	TCCCGTCTCC
SHQ1	-0333	-323	-	6.22	TGGGGCGGGA
RYBP	-0357	-347	-	5.72	GGCGGCGGCG
GXYLT2	-0328	-318	+	9.04	CCCCGCCGCC
MIR9170	-0393	-383	-	6.25	GTGGACAGGG
PROK2	-0368	-358	-	7.87	CGAGGCGGGG
DIPK2A	-0371	-361	+	5.92	CCCCGCACTC
DIPK2A	-0361	-351	+	6.75	CCCCCGCCCC
DIPK2A	-0332	-322	-	7.95	GGCGGGGGGG
TASP1	-0365	-355	-	9.09	GGGGGCGGAG
ISM1	-0376	-366	+	5.71	CCCCGGCCCG
ISM1	-0361	-351	+	6.90	CCCCGCCGCG
ISM1	-0327	-317	-	8.78	GGGAGCGGGG
SPTLC3	-0338	-328	+	7.12	CTCCACCCCC
LOC100034197	-0384	-374	-	7.54	GTGGGAGGAG
LOC100056127	-0358	-348	-	7.54	GTGGGAGGAG
SLC31A1	-0322	-312	-	5.64	GGCAGGGGGG
PRPF4	-0396	-386	+	6.90	CTCCTCCGCC
WDR31	-0368	-358	-	6.05	CAAGGCGGGG
BSPRY	-0322	-312	-	5.71	CGGGCCGGGG
HDHD3	-0380	-370	-	6.06	GGCGGGAGGG
HDHD3	-0368	-358	-	6.96	CGGGGCGGAG
HDHD3	-0353	-343	-	11.09	GGGGGCGGGG
HDHD3	-0328	-318	-	11.09	GGGGGCGGGG
ALAD	-0344	-334	-	9.67	GTGGGCGGGG
POLE3	-0324	-314	-	6.92	TGGGGAAGGG
Z.F618	-0397	-387	-	5.64	AGGGGCGGCG
Z.F618	-0379	-369	-	9.04	GGCGGCGGGG
AMBP	-0386	-376	+	5.95	CCCTGGCCCC
COL27A1	-0391	-381	+	6.65	CCCCCCACCC
ATP6V1G1	-0359	-349	+	7.12	TCCCTCCTCC
ATP6V1G1	-0348	-338	+	8.96	CCCCGCCCCT
T.FSF15	-0331	-321	+	7.70	CCCCACCCAC
T.C	-0398	-388	-	6.89	GGGAGCAGGG
TEX48	-0392	-382	+	6.25	CCCAGTCCAC
DCK	-0365	-355	+	8.96	CCCCGCCCCT
DCK	-0345	-335	+	9.09	CTCCGCCCCC
MOB1B	-0368	-358	-	8.96	AGGGGCGGGG
MOB1B	-0324	-314	-	7.77	GGGGGCGGCG
GRSF1	-0392	-382	+	8.96	CCCCGCCCCA
GRSF1	-0341	-331	+	7.87	CCCCGCCTCG
RUFY3	-0344	-334	+	7.77	CGCCGCCCCC
COL11A1	-0382	-372	-	7.68	GTGGGCGGAG
SQLE	-0367	-357	-	9.67	GTGGGCGGGG
SQLE	-0336	-326	-	9.67	GTGGGCGGGG
WASHC5	-0340	-330	+	6.96	CTCCGCCCCT
.SMCE2	-0334	-324	-	6.96	AGGGGCGGAG
TRIB1	-0339	-329	-	8.00	GGAGGCGGAG
PPM1B	-0325	-315	+	6.05	CCCCGCCTTT
PREPL	-0349	-339	-	5.89	TGAGGTGGGG
KLHL29	-0395	-385	+	9.67	CCCCGCCCAC
KLHL29	-0339	-329	+	6.62	GCCCGCCTCC

PLCB1	-0394	-384	-	6.33	GGGGGAAGGA
PLCB1	-0362	-352	-	6.92	CGGGGAAGGG
PLCB1	-0345	-335	-	7.86	GGAGGAGGAG
PLCB1	-0332	-322	-	7.14	GAGGGAGGAG
HAO1	-0379	-369	-	5.73	TGAGGAGGAG
HAO1	-0366	-356	-	5.71	GAGGAAAGGG
COX20	-0344	-334	+	6.96	CTCCGCCCCG
ADSS2	-0393	-383	-	5.82	CGCGGGGGGG
DESI2	-0347	-337	+	8.00	CTCCGCCTCC
DESI2	-0322	-312	-	5.82	AGCGGGGGGG
H.R.PU	-0389	-379	-	11.09	GGGGGCGGGG
H.R.PU	-0355	-345	-	11.09	GGGGGCGGGG

4 MA0039.2 Klf4 12.6183576774 0.771222760739 -371:-312
1.66990586527e-12

list of hits

DUSP5	-0367	-357	+	7.75	GGGGCGGGGA
XP.PEP1	-0352	-342	+	10.68	TGGGCGGGGC
XP.PEP1	-0336	-326	+	10.13	GGGGCGGGGC
SMC3	-0314	-304	+	7.12	GGGGAGGGGT
KAT6B	-0350	-340	-	7.85	CCCCCGCCCC
KAT6B	-0319	-309	-	7.25	GCAACGCCCA
LOC100061588	-0326	-316	+	5.66	AAGGTGTGGT
MSMO1	-0317	-307	+	6.37	GAGGCGGGGC
TMEM192	-0343	-333	-	7.53	GACACGCCCC
EPB41L3	-0328	-318	+	8.11	AGGGCGGGGA
SYT4	-0338	-328	+	5.94	GGGGAGGGGG
.R2E1	-0324	-314	+	5.35	GGGGTGTGTG
AFG1L	-0336	-326	-	10.13	GCCCCGCCCC
FOXO3	-0336	-326	-	7.06	GCCGCACCCC
SES.1	-0371	-361	+	8.11	AGGGCGGGGA
SES.1	-0347	-337	+	9.02	GGGGCGGGGT
OSTM1	-0358	-348	+	10.13	GGGGCGGGGC
PPIL6	-0369	-359	-	10.13	GCCCCGCCCC
PPIL6	-0356	-346	-	10.13	GCCCCGCCCC
PPIL6	-0346	-336	-	5.78	ATCCCACCCC
PPIL6	-0333	-323	-	6.84	GGCCCGCCCC
MICAL1	-0342	-332	-	5.88	CCCACTCCCC
ZBTB24	-0363	-353	-	10.13	GCCCCGCCCC
FIG4	-0364	-354	-	7.48	GCTCCGCCCC
FIG4	-0344	-334	+	10.13	GGGGCGGGGC
FIG4	-0326	-316	+	5.37	GAGGTGGGGT
WASF1	-0348	-338	+	10.13	GGGGCGGGGC
WASF1	-0316	-306	-	9.02	ACCCCGCCCC
DDO	-0327	-317	+	6.19	AGGGTGGGCT
AK9	-0338	-328	-	5.37	ACCCCACCTC
AK9	-0320	-310	-	10.13	GCCCCGCCCC
SLC22A16	-0357	-347	+	5.65	GGGGGGGGGC
SLC22A16	-0318	-308	+	10.13	GGGGCGGGGC
LOC102147786	-0355	-345	+	7.58	GGGGTGGAGC
E.GASE	-0369	-359	-	10.49	GCCCCGCCCT
E.GASE	-0353	-343	-	10.13	GCCCCGCCCC
E.GASE	-0338	-328	-	10.13	GCCCCGCCCC
CYTH1	-0315	-305	+	7.71	CGGGCGGGGC
SOCS3	-0328	-318	-	5.57	GCCCGGCCCA
CA4	-0333	-323	+	5.57	GGGGAGGAGC
GG.BP2	-0362	-352	+	6.37	GAGGCGGGGC
GG.BP2	-0335	-325	-	8.59	GCCCCTCCCT
GG.BP2	-0325	-315	-	10.60	GCCCCACCCT
DHRS11	-0369	-359	+	10.13	GGGGCGGGGC
DHRS11	-0349	-339	+	10.13	GGGGCGGGGC
DHRS11	-0318	-308	+	10.13	GGGGCGGGGC
LHX1	-0336	-326	-	7.85	CCCCCGCCCC
AATF	-0342	-332	-	6.71	GCAACGCCCC
AATF	-0318	-308	-	10.13	GCCCCGCCCC
USP32	-0359	-349	+	6.21	AGGGAGGGGA
USP32	-0337	-327	+	6.72	AGGGCGGAGT
USP32	-0324	-314	+	6.21	AGGGAGGGGA
USP32	-0312	-302	+	9.02	GGGGCGGGGT

SPTB.1	-0361	-351	-	5.24	GTCCCTCCCT
SPTB.1	-0320	-310	-	5.48	GCCCCGCGCA
PSME4	-0339	-329	+	7.95	GGGGTGGGGG
C15H2orf73	-0348	-338	-	6.79	GTCCCGCCCC
KC.S3	-0341	-331	+	7.70	GGGGTGGGTC
SMC6	-0323	-313	+	6.60	CGGGCGGGGT
SMC6	-0312	-302	+	10.13	GGGGCGGGGC
HPCAL1	-0351	-341	-	10.13	GCCCCGCCCC
HPCAL1	-0325	-315	-	7.15	GTCCCGCCCT
RRM2	-0361	-351	-	7.71	GCCCCGCCCG
RRM2	-0333	-323	-	10.61	GCCACGCCCA
KLF11	-0351	-341	-	6.95	GCCGCGCCCC
KLF11	-0332	-322	-	7.42	GCCGCACCCT
KLF11	-0314	-304	-	10.13	GCCCCGCCCC
TAF1B	-0348	-338	+	6.48	TGGGAGGGGG
ADAM17	-0351	-341	+	7.32	TGGGCGGTGC
CYS1	-0371	-361	-	10.13	GCCCCGCCCC
CYS1	-0349	-339	-	5.81	GCCCCTCCCG
ADI1	-0359	-349	-	5.81	GCCCCTCCCG
ADI1	-0334	-324	-	6.84	GGCCCGCCCC
TRAPPC12	-0366	-356	-	10.68	GCCCCGCCCA
DCDC2C	-0371	-361	+	5.17	CGGGCGGGTC
SHQ1	-0332	-322	+	6.79	GGGGCGGGAC
PROK2	-0361	-351	+	7.78	GGGGCGTGGG
DIPK2A	-0361	-351	-	7.85	CCCCCGCCCC
TASP1	-0312	-302	-	6.20	AGCACGCCCA
ISM1	-0357	-347	-	7.49	GCCGCGCCCA
SPTLC3	-0339	-329	-	5.20	TCTCCACCCC
LOC100034197	-0313	-303	+	5.78	GGGGAGTGGA
SLC31A1	-0356	-346	-	5.12	CCTACGCCCC
SLC31A1	-0315	-305	+	10.13	GGGGCGGGGC
WDR31	-0367	-357	+	6.73	AAGGCGGGGC
HDHD3	-0352	-342	+	10.13	GGGGCGGGGC
HDHD3	-0327	-317	+	10.13	GGGGCGGGGC
ALAD	-0343	-333	+	10.68	TGGGCGGGGC
Z.F618	-0319	-309	-	7.85	CCCCCGCCCC
AK.A	-0364	-354	+	5.15	TTGGCGGGGC
AK.A	-0341	-331	+	5.73	TGGGCGGAGG
ATP6V1G1	-0354	-344	-	5.19	CCTCCGCCCC
T.FSF15	-0332	-322	-	10.78	GCCCCACCCA
DCK	-0366	-356	-	10.13	GCCCCGCCCC
DCK	-0346	-336	-	7.48	GCTCCGCCCC
MOB1B	-0367	-357	+	9.02	GGGGCGGGGT
GRSF1	-0342	-332	-	6.37	GCCCCGCCTC
SQLE	-0366	-356	+	10.68	TGGGCGGGGC
SQLE	-0335	-325	+	10.68	TGGGCGGGGC
WASHC5	-0353	-343	-	10.43	GCCACGCCCT
.SMCE2	-0321	-311	+	10.43	AGGGCGTGGC
ABCG5	-0353	-343	-	7.24	GGCACACCCT
SLC3A1	-0357	-347	+	7.89	GGGGTGTGGG
SLC3A1	-0345	-335	+	5.80	TGGGTGTGTA
PLCB1	-0312	-302	+	8.20	AGGGCGGGGG
TMX4	-0325	-315	+	10.07	GGGGCGTGGC
COX20	-0345	-335	-	5.19	CCTCCGCCCC
H.R.PU	-0354	-344	+	10.13	GGGGCGGGGC
H.R.PU	-0326	-316	-	5.90	GCGCCGCCCC

5 MA0006.1 Arnt::Ahr 9.53241699429 0.715277771155 -530:-303 3.42488344196e-11

list of hits

RBM20	-0369	-363	-	7.05	CACGCG
MSMO1	-0491	-485	-	7.05	CACGCG
LOC100068026	-0346	-340	-	7.37	CACGCA
LOC100068026	-0346	-340	-	7.37	CACGCA
EPB41L3	-0467	-461	+	7.05	CGCGTG
.R2E1	-0426	-420	+	7.05	CGCGTG
ARMC2	-0332	-326	+	7.37	TGCGTG
SES.1	-0481	-475	-	7.37	CACGCA
CEP57L1	-0331	-325	-	7.37	CACGCA

CEP57L1	-0325	-319	+	7.37	TGCGTG
PPIL6	-0508	-502	-	7.05	CACGCG
LOC102147786	-0513	-507	-	7.37	CACGCA
USP36	-0386	-380	+	7.37	TGCGTG
CYTH1	-0528	-522	+	7.37	TGCGTG
CYTH1	-0477	-471	+	7.05	CGCGTG
CYTH1	-0435	-429	+	7.37	TGCGTG
CYTH1	-0375	-369	+	7.05	CGCGTG
CYTH1	-0363	-357	+	7.05	CGCGTG
CYTH1	-0345	-339	+	7.05	CGCGTG
AFMID	-0387	-381	+	7.05	CGCGTG
GG.BP2	-0456	-450	-	7.05	CACGCG
DHRS11	-0323	-317	+	7.05	CGCGTG
AATF	-0422	-416	-	7.05	CACGCG
AATF	-0408	-402	-	7.05	CACGCG
ACACA	-0396	-390	-	7.05	CACGCG
ACACA	-0390	-384	-	7.05	CACGCG
ACACA	-0372	-366	+	7.37	TGCGTG
ACACA	-0334	-328	+	7.37	TGCGTG
GE.1	-0488	-482	+	7.05	CGCGTG
GE.1	-0308	-302	-	7.37	CACGCA
SMC6	-0524	-518	+	7.37	TGCGTG
SMC6	-0344	-338	-	7.05	CACGCG
.OL10	-0416	-410	+	7.05	CGCGTG
ODC1	-0416	-410	-	7.05	CACGCG
HPCAL1	-0508	-502	+	7.05	CGCGTG
KLF11	-0460	-454	-	7.05	CACGCG
GRHL1	-0496	-490	-	7.05	CACGCG
GRHL1	-0481	-475	-	7.05	CACGCG
PDZR.3	-0365	-359	+	7.37	TGCGTG
PPP4R2	-0394	-388	-	7.05	CACGCG
SHQ1	-0313	-307	-	7.37	CACGCA
RYBP	-0448	-442	+	7.05	CGCGTG
PROK2	-0453	-447	+	7.05	CGCGTG
PROK2	-0379	-373	+	7.37	TGCGTG
DIPK2A	-0405	-399	+	7.05	CGCGTG
CDC26	-0519	-513	+	7.05	CGCGTG
PRPF4	-0498	-492	+	7.37	TGCGTG
ALAD	-0457	-451	-	7.05	CACGCG
ALAD	-0347	-341	+	7.37	TGCGTG
POLE3	-0374	-368	-	7.37	CACGCA
COL27A1	-0528	-522	-	7.05	CACGCG
COL27A1	-0317	-311	+	7.05	CGCGTG
T.FSF8	-0515	-509	-	7.37	CACGCA
T.FSF8	-0478	-472	-	7.37	CACGCA
DCK	-0304	-298	-	7.37	CACGCA
COL11A1	-0451	-445	+	7.05	CGCGTG
SQLE	-0486	-480	-	7.37	CACGCA
SQLE	-0395	-389	+	7.37	TGCGTG
.SMCE2	-0485	-479	-	7.05	CACGCG
DY.C2LI1	-0304	-298	-	7.37	CACGCA
PREPL	-0490	-484	-	7.37	CACGCA
TMX4	-0487	-481	-	7.05	CACGCG
TMX4	-0453	-447	-	7.05	CACGCG
HAO1	-0489	-483	+	7.37	TGCGTG
COX20	-0322	-316	-	7.05	CACGCG
ZBTB18	-0476	-470	+	7.05	CGCGTG
ZBTB18	-0468	-462	+	7.37	TGCGTG
ADSS2	-0525	-519	-	7.37	CACGCA
DESI2	-0530	-524	+	7.37	TGCGTG
H.R.PU	-0336	-330	+	7.37	TGCGTG
H.R.PU	-0303	-297	+	7.37	TGCGTG

6 MA0161.1 NFIC 6.98267924778 0.5685040611 -1507:-1417 1.32023656629e-09

list of hits

VDAC2	-1432	-1426	+	6.73	TTGGCA
LOC100068208	-1480	-1474	+	6.73	TTGGCA
LOC100068026	-1446	-1440	+	6.73	TTGGCA

LOC100068026	-1423	-1417	-	6.73	TGCCAA
LOC100068026	-1446	-1440	+	6.73	TTGGCA
LOC100068026	-1423	-1417	-	6.73	TGCCAA
RIT2	-1465	-1459	-	5.83	TGCCAG
SYT4	-1482	-1476	-	6.73	TGCCAA
SES.1	-1417	-1411	-	5.91	AGCCAA
OSTM1	-1506	-1500	+	6.73	TTGGCA
PPIL6	-1439	-1433	+	5.83	CTGGCA
PPIL6	-1432	-1426	-	5.91	AGCCAA
CD164	-1502	-1496	+	5.91	TTGGCT
C1QT.F1	-1498	-1492	+	5.91	TTGGCT
CA4	-1455	-1449	+	6.73	TTGGCA
PIGW	-1492	-1486	-	5.83	TGCCAG
LHX1	-1488	-1482	-	5.91	AGCCAA
USP32	-1457	-1451	+	5.91	TTGGCT
ERLEC1	-1424	-1418	+	5.83	CTGGCA
SMC6	-1426	-1420	+	5.91	TTGGCT
LOC100056590	-1481	-1475	+	5.91	TTGGCT
.OL10	-1441	-1435	-	5.83	TGCCAG
ODC1	-1418	-1412	+	5.83	CTGGCA
GRHL1	-1483	-1477	+	5.83	CTGGCA
TAF1B	-1457	-1451	+	5.91	TTGGCT
COLEC11	-1454	-1448	-	5.83	TGCCAG
TRAPPC12	-1423	-1417	+	5.83	CTGGCA
RYBP	-1455	-1449	-	5.91	AGCCAA
PROK2	-1507	-1501	+	6.73	TTGGCA
SPTLC3	-1502	-1496	+	5.91	TTGGCT
LOC100056127	-1492	-1486	+	6.73	TTGGCA
FKBP15	-1432	-1426	+	5.91	TTGGCT
SLC31A1	-1473	-1467	+	6.73	TTGGCA
PRPF4	-1468	-1462	-	5.91	AGCCAA
RGS3	-1451	-1445	+	5.91	TTGGCT
RGS3	-1427	-1421	+	5.91	TTGGCT
ATP6V1G1	-1445	-1439	+	5.83	CTGGCA
T.FSF15	-1461	-1455	+	6.73	TTGGCA
LOC100050100	-1489	-1483	+	5.91	TTGGCT
GC	-1420	-1414	+	6.73	TTGGCA
JCHAI.	-1425	-1419	+	5.83	CTGGCA
SLC4A4	-1466	-1460	-	6.73	TGCCAA
SLC4A4	-1449	-1443	-	6.73	TGCCAA
WASHC5	-1423	-1417	+	5.83	CTGGCA
TRIB1	-1469	-1463	+	5.83	CTGGCA
LRPPRC	-1492	-1486	+	5.83	CTGGCA
SLC3A1	-1460	-1454	+	6.73	TTGGCA
TMX4	-1500	-1494	-	5.83	TGCCAG
C25H9orf43	-1443	-1437	+	5.91	TTGGCT
DESI2	-1490	-1484	+	5.83	CTGGCA
H.R.PU	-1425	-1419	-	5.83	TGCCAG

7 MA0104.2 Mycn 11.1040870395 0.698630136997 -617:-448 2.92984293472e-09

list of hits

XP.PEP1	-0600	-590	-	5.70	GCCACGTTCC
KAT6B	-0538	-528	-	6.05	GGCATGTGGG
VDAC2	-0606	-596	+	7.42	CCCACGCGGG
DUPD1	-0544	-534	+	5.80	TCCACGTGAT
LOC100068320	-0558	-548	-	6.05	GGCATGTGGG
CPE	-0482	-472	+	6.63	TTCACGTGGT
FOXO3	-0616	-606	-	5.97	GCCCCGTGCG
PPIL6	-0510	-500	+	6.64	GGCACGCGGG
SMPD2	-0466	-456	+	6.17	ACCGCGCGGC
CD164	-0489	-479	+	6.06	GTCACGTGGG
USP36	-0487	-477	+	6.72	CCCACGTGCG
CYTH1	-0544	-534	-	5.82	GCAGCGTGCC
CYTH1	-0479	-469	-	5.69	CGCGCGTGCG
AFMID	-0617	-607	+	5.79	ACCACGGGGC
AFMID	-0568	-558	-	5.56	GCCACGTTGA
GG.BP2	-0552	-542	+	6.20	CCCGCGCGGC
AATF	-0471	-461	+	7.67	TCCACGTGCC

```
                  ACACA   -0592   -582    +       7.37    CCCGCGTGGC
                  ACACA   -0450   -440    -       7.15    GCCTCGTGCC
                  C15H2orf73      -0559   -549    -       6.20    GCCGCGCGGG
                  KC.S3   -0586   -576    -       5.95    GCCACATGCC
                  GE.1    -0490   -480    -       5.69    CGCGCGTGCG
                  ODC1    -0604   -594    -       6.10    TGCGCGTGCG
                  HPCAL1  -0510   -500    +       5.92    CCCGCGTGGA
                  KLF11   -0511   -501    +       7.09    AACACGTGCC
                  KLF11   -0462   -452    +       7.63    ATCACGCGGC
                  GRHL1   -0498   -488    +       6.91    GCCACGCGGA
                  GRHL1   -0483   -473    +       6.91    GCCACGCGGA
                  ADAM17  -0593   -583    +       7.55    CGCACGCGCC
                  DCDC2C  -0590   -580    -       6.48    GCCACGTTGG
                  RYBP    -0587   -577    -       5.65    GGCACGCGCG
                  PROK2   -0520   -510    +       9.10    AGCACGTGGA
                  PROK2   -0455   -445    -       7.55    GGCGCGTGCG
                  ZFP37   -0572   -562    +       8.96    AACACGTGGC
                  CDC26   -0521   -511    -       5.80    GGCGCGTGAG
                  ALAD    -0459   -449    +       7.42    CCCACGCGGG
                  COL27A1 -0587   -577    +       6.91    TCCACGCGGA
                  COL27A1 -0530   -520    +       6.50    TCCACGCGCC
                  AK.A    -0612   -602    +       8.59    CCCACGTGGG
                  AK.A    -0599   -589    +       5.55    CCCACGCGCG
                  TEX48   -0568   -558    +       5.68    GCCACGCTGC
                  GRSF1   -0548   -538    +       6.13    GCCACGTGTT
                  COL11A1 -0617   -607    +       7.27    AGCACGCGTC
                  SQLE    -0448   -438    -       5.59    GCCGCCTGCG
                  .SMCE2  -0487   -477    +       6.10    CGCACGCGCA
                  TRIB1   -0573   -563    -       5.84    GCCACCTGCA
                  PREPL   -0465   -455    +       6.56    ACCACGCTGC
                  KLHL29  -0601   -591    -       7.52    GCCACGCGCG
                  PLCB1   -0569   -559    +       5.70    GGAACGTGGC
                  TMX4    -0489   -479    +       5.96    CACACGCGCC
                  TMX4    -0455   -445    +       6.54    CACACGCGGT
                  C25H9orf43      -0567   -557    +       6.15    CTCACGTTGC
                  COX20   -0600   -590    +       6.35    CGCGCGCGGC
                  COX20   -0485   -475    -       7.88    GCCATGTGGT
                  ZBTB18  -0478   -468    -       9.42    GCCGCGTGCG

    8   MA0147.1        Myc     11.157239362    0.685903085415  -617:-455
 3.06174041938e-09
                  list of hits
                  DUSP5   -0467   -457    +       5.91    AGCACACGGA
                  XP.PEP1 -0600   -590    -       6.63    GCCACGTTCC
                  KAT6B   -0538   -528    +       5.57    GGCATGTGGG
                  VDAC2   -0606   -596    +       7.11    CCCACGCGGG
                  DUPD1   -0544   -534    -       7.11    TCCACGTGAT
                  LOC100068320    -0558   -548    +       5.57    GGCATGTGGG
                  CPE     -0482   -472    +       6.37    TTCACGTGGT
                  APELA   -0605   -595    +       5.32    TGCATGTGGG
                  LOC106780946    -0460   -450    +       5.61    CGCCCGCGGC
                  PPIL6   -0510   -500    +       7.03    GGCACGCGGG
                  CD164   -0489   -479    +       5.88    GTCACGTGGG
                  TIMP2   -0488   -478    -       6.46    GCCAGGTGCG
                  USP36   -0487   -477    +       6.13    CCCACGTGCG
                  CYTH1   -0544   -534    -       5.39    GCAGCGTGCC
                  CYTH1   -0479   -469    -       5.65    CGCGCGTGCG
                  SOCS3   -0547   -537    +       5.61    CGCCCGCGGC
                  AFMID   -0568   -558    -       5.49    GCCACGTTGA
                  MYO19   -0574   -564    -       6.11    GCCAGGTGCT
                  LHX1    -0531   -521    -       6.01    GCCACGGGCC
                  LHX1    -0485   -475    +       6.46    CGCACCTGGC
                  AATF    -0471   -461    +       6.39    TCCACGTGCC
                  ACACA   -0592   -582    +       6.28    CCCGCGTGGC
                  KC.S3   -0586   -576    -       7.04    GCCACATGCC
                  GE.1    -0490   -480    -       5.65    CGCGCGTGCG
                  ODC1    -0604   -594    -       6.27    TGCGCGTGCG
                  HPCAL1  -0510   -500    +       5.43    CCCGCGTGGA
                  KLF11   -0511   -501    +       6.81    AACACGTGCC
```

```
                    KLF11   -0462   -452    +       6.60    ATCACGCGGC
                    GRHL1   -0498   -488    +       6.76    GCCACGCGGA
                    GRHL1   -0483   -473    +       6.76    GCCACGCGGA
                    YWHAQ   -0476   -466    +       5.73    ACCCCGTGGC
                    ADAM17  -0593   -583    +       7.13    CGCACGCGCC
                    DCDC2C  -0590   -580    -       6.71    GCCACGTTGG
                    MIR9170 -0544   -534    +       5.49    GGCACTTGGC
                    PROK2   -0520   -510    +       9.63    AGCACGTGGA
                    PROK2   -0455   -445    -       7.13    GGCGCGTGCG
                    ISM1    -0616   -606    -       5.80    CCCATGTGCA
                    ZFP37   -0572   -562    +       9.16    AACACGTGGC
                    ALAD    -0459   -449    +       7.11    CCCACGCGGG
                    COL27A1 -0587   -577    +       6.51    TCCACGCGGA
                    AK.A    -0612   -602    +       8.47    CCCACGTGGG
                    AK.A    -0599   -589    -       5.70    CCCACGCGCG
                    LOC100050034    -0496   -486    -       5.47    TCCACATGTT
                    LOC100051562    -0512   -502    -       5.47    TCCACATGTT
                    LOC100050100    -0501   -491    -       5.47    TCCACATGTT
                    GRSF1   -0548   -538    +       6.35    GCCACGTGTT
                    JCHAI.  -0539   -529    +       5.91    AAAACGTGGC
                    COL11A1 -0617   -607    +       7.22    AGCACGCGTC
                    .SMCE2  -0487   -477    +       6.27    CGCACGCGCA
                    TRIB1   -0573   -563    -       5.54    GCCACCTGCA
                    KLHL29  -0601   -591    -       7.18    GCCACGCGCG
                    PLCB1   -0569   -559    +       6.63    GGAACGTGGC
                    TMX4    -0489   -479    +       5.80    CACACGCGCC
                    TMX4    -0455   -445    +       7.41    CACACGCGGT
                    COX20   -0600   -590    +       5.81    CGCGCGCGGC
                    COX20   -0485   -475    +       5.42    GCCATGTGGT
                    ZBTB18  -0478   -468    -       9.48    GCCGCGTGCG

    9   MA0003.1        TFAP2A  7.8120920509    0.800000024164  -668:-633
8.14016173123e-08
                    list of hits
                    DUSP5   -0653   -644    +       6.45    GCCCCCAGC
                    RBM20   -0658   -649    +       7.05    GCCCTAGGG
                    ADK     -0652   -643    +       6.28    GCCGTGAGG
                    COMTD1  -0648   -639    +       7.22    GCCCCAGGG
                    LOC100067994    -0661   -652    -       6.11    GCGTGGGGC
                    LOC106780946    -0664   -655    -       6.39    GCCGACGGC
                    LOC106780946    -0638   -629    -       6.45    CCTCGCGGC
                    FOXO3   -0661   -652    +       7.25    GCCCTGGGG
                    SES.1   -0653   -644    +       6.31    GCCCCGCGC
                    CEP57L1 -0642   -633    -       6.31    GCGCGGGGC
                    AK9     -0664   -655    -       6.62    CCCTACGGC
                    CYTH1   -0667   -658    +       6.87    GCCCCGAGC
                    SOCS3   -0658   -649    +       6.57    GCCGCCGGG
                    SOCS3   -0637   -628    -       6.64    CCCGCCGGC
                    CA4     -0644   -635    +       6.86    GCCGGAGGG
                    ACACA   -0647   -638    +       6.99    GCCGCGGGG
                    ACACA   -0634   -625    +       6.34    GCCCAGGGG
                    SPTB.1  -0663   -654    +       7.29    GCCCGAGGG
                    ASB3    -0656   -647    +       6.32    GCCTGAAGG
                    PSME4   -0668   -659    +       7.00    GCCCCCGGG
                    GE.1    -0645   -636    -       6.47    CCTGGGGGC
                    ODC1    -0643   -634    +       6.51    GCCTGGAGC
                    HPCAL1  -0658   -649    +       6.57    GCCTCCGGG
                    HPCAL1  -0638   -629    -       6.64    CCCGCAGGC
                    RRM2    -0667   -658    +       6.64    GCCGGCGGG
                    GRHL1   -0652   -643    -       6.82    GCCGAGGGC
                    CYS1    -0663   -654    +       6.16    GCCCCTGGC
                    CYS1    -0651   -642    +       6.99    GCCCCCGGC
                    DCDC2C  -0642   -633    +       6.79    GCCGCAGGG
                    DIPK2A  -0665   -656    -       6.64    CCCGCCGGC
                    DIPK2A  -0653   -644    +       6.99    GCCCCCGGC
                    SLC31A2 -0633   -624    +       7.04    GCCTGGGGC
                    AK.A    -0657   -648    +       6.64    GCCGGCGGG
                    DCK     -0640   -631    -       6.52    CCTCCAGGC
                    TRIB1   -0660   -651    +       7.41    GCCCCGGGC
```

```
                TRIB1   -0649   -640    +       6.86    GCCGGAGGG
                C25H9orf43      -0650   -641    +       6.78    GCCTCAGGC
                COX20   -0634   -625    -       7.07    CCCGCGGGC

    10  MA0076.1        ELK4    14.1232301212   0.583333336645  -345:-246
 1.34472905636e-07
                list of hits
                SAMD8   -0342   -333    -       5.68    CCTTCCGGT
                SAMD8   -0271   -262    -       8.59    AGTTCCGGT
                S.X3    -0311   -302    +       5.76    GCCGGAACC
                OSTM1   -0279   -270    +       8.42    GCCGGAAGC
                ZBTB24  -0289   -280    -       7.75    GCTTCCGGG
                FIG4    -0309   -300    +       6.44    TCCGGAAAC
                AK9     -0255   -246    +       5.09    CCCGGAACC
                USP36   -0247   -238    -       5.73    GTATCCGGC
                TMEM235 -0307   -298    +       5.09    CCCGGAACC
                AATF    -0296   -287    +       8.52    CCCGGAAGT
                CHAC2   -0345   -336    +       5.36    GCCGGAAAA
                CHAC2   -0263   -254    -       3.88    ACAGCCGGT
                ERLEC1  -0341   -332    +       7.14    CCCGGATGT
                ERLEC1  -0317   -308    +       6.44    CCCGGAAAC
                C15H2orf73      -0254   -245    -       4.69    TTTTCCGGG
                RDH14   -0277   -268    +       8.52    CCCGGAAGT
                LOC100056590    -0249   -240    +       3.94    ACAGGAAAT
                .OL10   -0332   -323    -       6.00    TCTTCCGGG
                .OL10   -0314   -305    -       7.11    GTTTCCGGC
                .OL10   -0304   -295    +       8.52    CCCGGAAGT
                ADAM17  -0327   -318    +       8.42    GCCGGAAGC
                ADAM17  -0286   -277    -       7.75    GCTTCCGGG
                ADAM17  -0255   -246    -       8.73    TCTTCCGGT
                RPS7    -0259   -250    -       8.73    TCTTCCGGT
                TRAPPC12        -0345   -336    +       9.17    ACCGGAAAC
                SHQ1    -0289   -280    +       8.42    GCCGGAAGC
                TASP1   -0333   -324    -       8.42    GCTTCCGGC
                TASP1   -0247   -238    -       11.25   ACTTCCGGT
                LOC100034197    -0282   -273    -       5.25    ACTCCCGGT
                LOC100056127    -0257   -248    -       5.25    ACTCCCGGT
                FKBP15  -0286   -277    -       3.88    ACATCCCGT
                PRPF4   -0320   -311    +       3.94    ACCGCAAAT
                ALAD    -0246   -237    -       7.75    GCTTCCGGG
                LOC100050034    -0337   -328    +       4.31    ACCGGATGG
                LOC100050034    -0276   -267    -       5.25    ACTTCCGTT
                LOC100050100    -0342   -333    +       7.35    ACCGGATGA
                LOC100050100    -0281   -272    -       5.25    ACTTCCGTT
                UTP3    -0304   -295    +       4.77    ACCGGAATC
                CAMKMT  -0301   -292    +       9.94    ACCGGAAAT
                ABCG5   -0296   -287    -       4.01    TGTTCCGGC

    11  MA0162.1        Egr1    14.4555832005   0.739393945445  -663:-597
 8.5752756276e-07
                list of hits
                KLHL2   -0626   -615    -       4.02    CCGCTCCCGGC
                LOC100067994    -0641   -630    +       4.65    ACTGGGGGTGG
                LOC106780946    -0601   -590    +       8.52    CGCGGGGGCGA
                TMEM200C        -0654   -643    +       6.59    TGCGAGGGCGG
                TMEM200C        -0617   -606    -       6.04    CCGCCCTCGCG
                FOXO3   -0632   -621    +       7.48    GGCGGGGGAGG
                LOC102147786    -0612   -601    +       4.78    TGCCTGGGAGG
                CYTH1   -0626   -615    +       9.06    CGCGGAGGCGG
                AFMID   -0633   -622    -       6.50    CCACCCCCGGT
                ACACA   -0604   -593    -       7.54    CCTCCCCCACA
                USP32   -0651   -640    +       9.95    CGTGTGGGCGG
                USP32   -0602   -591    +       6.04    CGCGCGGGCGG
                SPTB.1  -0632   -621    +       6.51    TGGGTGGGCGG
                PSME4   -0610   -599    -       4.73    CCTCCTCCGGT
                ERLEC1  -0617   -606    -       7.06    CCGCTCACACT
                C15H2orf73      -0605   -594    +       4.44    CGCCGGGGCGG
                GE.1    -0645   -634    +       6.13    CCTGGGGGCGT
                SMC6    -0626   -615    -       6.44    TCTCCCACACG
```

```
                ODC1    -0654   -643    +       5.88    CGCGTGCGCGG
                ODC1    -0602   -591    +       5.88    CGCGTGCGCGT
                HPCAL1  -0660   -649    -       6.60    CCGCCTCCGGG
                KLF11   -0600   -589    -       10.43   CCGCCCCCGCG
                GRHL1   -0662   -651    +       4.18    CAAGTGGGCGG
                YWHAQ   -0614   -603    +       6.63    CGCGGGAGTGG
                CYS1    -0653   -642    -       6.62    CCGCCCCCGGC
                ADI1    -0661   -650    -       8.37    CCGCCCCCGTT
                ADI1    -0634   -623    -       6.10    ACACCCCCGTC
                TRAPPC12        -0612   -601    +       4.78    TGCGTGGCAGG
                PROK2   -0630   -619    -       6.79    ACACCCCCGGG
                DIPK2A  -0655   -644    -       6.62    CCGCCCCCGGC
                ISM1    -0659   -648    -       7.54    CCGCTCCCGCT
                LOC100034197    -0662   -651    +       4.21    GATGTGAGCGT
                LOC100056127    -0638   -627    +       4.21    GATGTGAGCGG
                AK.A    -0615   -604    -       6.52    TCTCCCACGTG
                LOC100051420    -0597   -586    -       7.48    CCTCCCCCGCC
                LOC100050034    -0661   -650    -       4.11    TCACCCCCACC
                LOC100051562    -0663   -652    -       7.48    CCTCCCCCGCC
                LOC100050100    -0652   -641    -       5.62    CCTCCCCCACC
                TRIB1   -0613   -602    +       7.98    CCCGGGGGCGT
                ABCG5   -0618   -607    -       4.74    CCACTTACGGA
                C25H9orf43      -0658   -647    -       4.59    TCACCTCCGCC
                C30H1orf100     -0597   -586    -       4.78    CCACTCCCACG

    12  MA0109.1        Hltf    7.46926527425   0.397046551108  -2165:-2133
 1.72088122308e-06
                list of hits
                DUSP5   -2164   -2154   +       5.70    GCCCTTATCA
                LOC100068208    -2149   -2139   +       6.18    AACCATATGC
                LOC100068320    -2138   -2128   -       5.98    TCATAAGGAC
                MSMO1   -2156   -2146   +       5.61    ATACATATAG
                LOC100066472    -2155   -2145   +       5.73    ATCCATAGAT
                LOC100066472    -2141   -2131   -       5.78    GGATAAGGGA
                LOC100068026    -2154   -2144   +       5.84    TTCCTTTTAC
                LOC100068026    -2154   -2144   +       5.84    TTCCTTTTAC
                MICAL1  -2147   -2137   +       5.66    GAACATATCC
                WASF1   -2145   -2135   -       5.75    ACCTAAGTTA
                CA.T1   -2134   -2124   +       5.76    AACCTTATTG
                CYTH1   -2162   -2152   -       5.92    TGAAAAGGTC
                C15H2orf73      -2164   -2154   +       5.68    TAACATTTGT
                GE.1    -2144   -2134   +       5.75    TAACTTAGGT
                TRAPPC12        -2154   -2144   +       5.66    ACACATATGT
                DCDC2C  -2140   -2130   +       5.74    ATACTTTTGC
                GXYLT2  -2156   -2146   +       5.68    GTACTTTTCT
                SHQ1    -2163   -2153   +       5.76    GACCTTAGAA
                GXYLT2  -2156   -2146   +       5.68    GTACTTTTCT
                LOC100034197    -2133   -2123   -       6.38    CTATAAGGTT
                LOC100056127    -2156   -2146   +       5.81    TAACATATAC
                CDC26   -2165   -2155   +       5.75    ACACTTTTAT
                PRPF4   -2157   -2147   -       5.87    CTAAAAGGAT
                POLE3   -2156   -2146   +       5.63    ATCCATATTT
                Z.F618  -2165   -2155   -       5.69    GCCTAAGGTC
                ATP6V1G1        -2151   -2141   +       5.77    ACCCTTTTAA
                LOC100051420    -2134   -2124   -       6.38    CTATAAGGTT
                RUFY3   -2136   -2126   -       5.81    GTAAAAGGTG
                DY.C2LI1        -2150   -2140   -       5.71    AGAAAAGGGA
                ABCG5   -2138   -2128   +       5.80    AACCTTGTCC
                PREPL   -2153   -2143   -       5.85    CTAAAAGTTT
                PREPL   -2143   -2133   -       5.66    GCAAATGTTT
                PLCB1   -2146   -2136   +       5.66    TTCCTTTTAG
                C25H9orf43      -2155   -2145   +       6.17    AAACATATGT

    13  MA0056.1        MZF1_1-4        8.58551308885   0.724999990314  -2180:-2114
 2.89031386155e-06
                list of hits
                ADD3    -2167   -2161   -       7.05    TCCCCA
                KAT6B   -2118   -2112   +       7.05    TGGGGA
                DUSP13  -2169   -2163   -       7.05    TCCCCA
```

```
                COMTD1  -2155   -2149   +       7.05    TGGGGA
                LOC100067994    -2157   -2151   +       7.05    TGGGGA
                LOC100146977    -2156   -2150   -       7.05    TCCCCA
                FOXO3   -2114   -2108   +       7.05    TGGGGA
                FIG4    -2129   -2123   -       7.05    TCCCCA
                RBFOX3  -2174   -2168   -       7.05    TCCCCA
                D.AH17  -2136   -2130   -       7.05    TCCCCA
                CA4     -2145   -2139   -       7.05    TCCCCA
                SPTB.1  -2151   -2145   +       7.05    TGGGGA
                SPTB.1  -2140   -2134   +       7.05    TGGGGA
                CHAC2   -2172   -2166   +       7.05    TGGGGA
                CHAC2   -2162   -2156   +       7.05    TGGGGA
                KC.S3   -2133   -2127   +       7.05    TGGGGA
                RDH14   -2180   -2174   -       7.05    TCCCCA
                .OL10   -2119   -2113   +       7.05    TGGGGA
                GRHL1   -2158   -2152   -       7.05    TCCCCA
                YWHAQ   -2139   -2133   +       7.05    TGGGGA
                CYS1    -2173   -2167   +       7.05    TGGGGA
                CYS1    -2126   -2120   -       7.05    TCCCCA
                PPP4R2  -2175   -2169   -       7.05    TCCCCA
                BSPRY   -2143   -2137   -       7.05    TCCCCA
                KIF12   -2134   -2128   +       7.05    TGGGGA
                COL27A1 -2119   -2113   -       7.05    TCCCCA
                T.FSF8  -2180   -2174   +       7.05    TGGGGA
                TEX48   -2158   -2152   -       7.05    TCCCCA
                GRSF1   -2135   -2129   -       7.05    TCCCCA
                TRIB1   -2165   -2159   -       7.05    TCCCCA
                TRIB1   -2116   -2110   +       7.05    TGGGGA
                C25H9orf43      -2174   -2168   +       7.05    TGGGGA

        14  MA0004.1        Arnt    10.9916749403   0.641666668778  -2118:-1953
    9.70150131415e-06
                list of hits
                LOC100067994    -2112   -2106   -       6.62    CACGTT
                S.X3    -1953   -1947   -       6.62    CACGTT
                METTL24 -2118   -2112   +       8.00    CACGTG
                DDO     -2085   -2079   +       8.00    CACGTG
                RBFOX3  -2046   -2040   +       6.62    AACGTG
                LGALS3BP        -2084   -2078   -       6.62    CACGTT
                LGALS3BP        -2077   -2071   -       6.62    CACGTT
                TIMP2   -2051   -2045   +       6.62    AACGTG
                CYTH1   -2072   -2066   +       6.62    AACGTG
                MRM1    -2029   -2023   +       8.00    CACGTG
                KC.S3   -2011   -2005   +       8.00    CACGTG
                ODC1    -2110   -2104   +       8.00    CACGTG
                RRM2    -2013   -2007   +       8.00    CACGTG
                TASP1   -1961   -1955   +       6.62    AACGTG
                LOC100056127    -2028   -2022   +       6.62    AACGTG
                HDHD3   -1962   -1956   -       6.62    CACGTT
                RGS3    -1976   -1970   +       6.62    AACGTG
                KIF12   -2044   -2038   -       6.62    CACGTT
                WASHC5  -2079   -2073   +       6.62    AACGTG
                ABCG8   -2028   -2022   -       6.62    CACGTT
                ABCG8   -2017   -2011   +       6.62    AACGTG
                PREPL   -1987   -1981   +       6.62    AACGTG
                COX20   -2092   -2086   +       6.62    AACGTG

        15  MA0035.2        Gata1   10.8778685237   0.372818182556  -2790:-2747
    1.03175348622e-05
                list of hits
                XP.PEP1 -2786   -2775   +       7.06    GGTGATAAGTG
                VDAC2   -2780   -2769   +       6.12    GGAGATAGAGG
                DUPD1   -2749   -2738   -       8.50    CTCTTATCTAT
                LOC100061588    -2773   -2762   -       6.50    CTTTTATCATA
                LOC100066359    -2756   -2745   +       8.65    TAAGATAAGGA
                EPB41L3 -2752   -2741   -       7.30    CTGTTATCTAT
                RIT2    -2761   -2750   +       8.11    CCAGATAAAGA
                SYT4    -2790   -2779   -       8.32    CCCTTATCAGA
                FIG4    -2748   -2737   +       8.30    CCTGATAAGGA
```

SLC22A16 -2758 -2747 - 7.19 TTGTTATCACA
C1QT.F1 -2771 -2760 - 6.35 TTCTAATCTCT
C15H2orf73 -2752 -2741 - 8.36 TTCTTATCAGG
ADAM17 -2785 -2774 - 8.62 TCTTTATCTCC
ADAM17 -2761 -2750 - 7.19 TGTTTATCTTA
SHQ1 -2788 -2777 - 8.62 TGTTTATCTGT
PROK2 -2748 -2737 + 6.18 CAAGATAGGAA
SPTLC3 -2748 -2737 + 6.41 ACTGATAATAC
RGS3 -2754 -2743 - 5.95 ACATTATCTGG
GRSF1 -2785 -2774 - 7.76 TCCTTATCAAC
LOC100049851 -2779 -2768 + 6.82 TGAGATAAACT
SQLE -2747 -2736 - 6.41 GTATTATCAGT
WASHC5 -2787 -2776 - 6.70 TATTTATCTCA
.SMCE2 -2759 -2748 + 6.12 TAAGATAATAG
PPM1B -2763 -2752 - 8.11 TCTTTATCTGG
TMX4 -2785 -2774 - 6.70 TATTTATCTCA

16 MA0048.1 NHLH1 14.131557299 0.674382714037 -244:-211
1.38923056368e-05
list of hits
XP.PEP1 -0231 -219 + 7.69 GATCAGCTGGTC
SMC3 -0212 -200 + 5.65 AAGCAGGTGAGG
ADK -0219 -207 + 8.43 CAGCAGCTGAGG
KAT6B -0243 -231 + 4.95 GGGCAGCTTCTC
SAMD8 -0242 -230 - 6.94 GCGCGGCTGAGA
VDAC2 -0213 -201 + 9.13 CCGCAGCTGCAG
TMEM192 -0214 -202 + 5.11 GCGCAGGTGGAG
LOC100066444 -0229 -217 - 5.93 CTACAGCTGAGT
S.X3 -0231 -219 + 5.42 TCTCAGCCGGGC
ZBTB24 -0244 -232 + 5.53 CAGAAGCTGGGG
METTL24 -0233 -221 + 5.71 GCGCTGCTGCTC
CYTH1 -0230 -218 + 4.60 GGCCAGGTGCGG
ACACA -0236 -224 - 4.95 GAGCAGCTTCCC
ACYP2 -0219 -207 + 4.74 CCCCAGCTGCCC
VS.L1 -0215 -203 - 6.00 GCGCGCCTGCGC
LOC100034197 -0239 -227 - 4.70 ATGAAGCTGCTG
LOC100056127 -0214 -202 - 4.70 ATGAAGCTGCTG
SLC31A2 -0211 -199 + 7.32 GAGAAGCTGCGG
AMBP -0241 -229 + 5.31 TCTCAGCTGCCT
KIF12 -0224 -212 + 5.12 CCGGAGCTGCTG
ABCG5 -0220 -208 + 6.87 GAGCAGGTGGGG
LRPPRC -0237 -225 - 5.42 GCCCTGCTGAGA
HAO1 -0212 -200 + 5.48 ACGCAGCTTTGA
CATSPERE -0238 -226 - 6.04 GCGCTGCTGTGG
CATSPERE -0224 -212 + 4.88 GCTGAGCTGCGG
DESI2 -0232 -220 - 4.58 CCGAGGCTGCGC

17 MA0004.1 Arnt 10.9916749403 0.641666668778 -1132:-1054
1.65716648912e-05
list of hits
LOC100068208 -1068 -1062 + 6.62 AACGTG
LOC100066501 -1067 -1061 + 6.62 AACGTG
LOC100067994 -1054 -1048 - 6.62 CACGTT
ARMC2 -1114 -1108 + 6.62 AACGTG
CEP57L1 -1066 -1060 - 6.62 CACGTT
AATF -1102 -1096 + 8.00 CACGTG
ODC1 -1116 -1110 + 6.62 AACGTG
SHQ1 -1060 -1054 - 6.62 CACGTT
RYBP -1102 -1096 + 8.00 CACGTG
ALAD -1101 -1095 + 6.62 AACGTG
ALAD -1075 -1069 - 6.62 CACGTT
AMBP -1132 -1126 - 6.62 CACGTT
T.FSF15 -1087 -1081 + 8.00 CACGTG
LOC100051562 -1126 -1120 + 6.62 AACGTG
RUFY3 -1095 -1089 - 6.62 CACGTT
LRPPRC -1122 -1116 + 8.00 CACGTG

18 MA0032.1 FOXC1 6.50807164809 0.4453125 -2707:-2650
2.067367994e-05

list of hits

ADD3	-2652	-2644	-	5.72	TACTTTCC
SMC3	-2668	-2660	-	5.54	TACTCACG
KAT6B	-2651	-2643	+	5.43	GGGAAGTA
LOC100066472	-2684	-2676	+	5.54	GCAAAGTA
L3MBTL4	-2686	-2678	-	5.94	TACTTACC
MICAL1	-2677	-2669	+	5.72	GGAAAGTA
CA.T1	-2650	-2642	+	5.54	AGTGAGTA
PIGW	-2653	-2645	-	5.54	TACTCACG
ACACA	-2707	-2699	-	5.72	TACTTACT
SPTLC3	-2662	-2654	+	5.72	GGCAAGTA
CDC26	-2676	-2668	+	5.50	AGAAAGTA
R.F183	-2700	-2692	-	5.54	TACTCACG
HDHD3	-2670	-2662	-	5.54	TACTTAGT
WASHC5	-2704	-2696	-	5.94	TACTTACC
PPM1B	-2694	-2686	-	5.72	TACTTACT
SLC3A1	-2702	-2694	-	5.50	TACTTGCT
COX20	-2684	-2676	+	5.54	ACTAAGTA
ZBTB18	-2677	-2669	-	5.50	TACTTTCT

19 MA0145.1 Tcfcp2l1 11.6502911816 0.609628129495 -252:-218 2.15932001258e-05

list of hits

RBM20	-0249	-235	-	5.83	CAGGACCTCACCGG
COMTD1	-0221	-207	+	6.81	CCGGCCTCTTCCTG
MSMO1	-0241	-227	-	5.76	CTGGGAGAGGCAGG
CPE	-0232	-218	+	9.16	GCAGCTCCAGCCAG
D.AH17	-0223	-209	+	5.87	CTAGAAGAGGCCAG
MYO19	-0248	-234	-	5.64	CTGGAGAAAACCGC
PIGW	-0227	-213	+	6.05	CCTGCTCAAGCCTC
GG.BP2	-0227	-213	+	6.09	CCCGCCCAGGCCAG
DHRS11	-0229	-215	-	5.94	CAGGATGGAGGCGG
GE.1	-0227	-213	-	6.72	CCGGAAGAGGCAGG
RRM2	-0245	-231	+	5.97	CCTGTCTGAACCGC
CYS1	-0224	-210	-	5.96	CCGGCGGGGCCTGG
GXYLT2	-0218	-204	+	5.78	CCTGCTCGCGCTGG
GXYLT2	-0218	-204	+	5.78	CCTGCTCGCGCTGG
LOC100034197	-0243	-229	-	7.30	CAGGATGAAGCTGC
LOC100056127	-0218	-204	-	7.30	CAGGATGAAGCTGC
ALAD	-0252	-238	+	5.65	GCAGCTGCTTCCGG
Z.F618	-0224	-210	-	6.20	CCGGGAGGAGCAGG
AMBP	-0235	-221	-	6.63	CTGCCTCCAGCAGG
T.C	-0236	-222	+	6.42	CTGGCCACAGCCTG
RUFY3	-0229	-215	-	6.98	CTGGCTCGGACTCG
E.AM	-0223	-209	-	7.13	CTGGTACATACAGG
CAMKMT	-0237	-223	-	7.03	CAGGTCCTGACAGG
LRPPRC	-0235	-221	+	7.56	CCTGCTGAGACCTG
C25H9orf43	-0246	-232	-	6.67	GTGGACTGAGCCGG
ADSS2	-0251	-237	-	6.90	CCGGCGGGGGCAGG

20 MA0125.1 Nobox 9.57325016988 0.25986841647 -3240:-3200 2.50110282093e-05

list of hits

SMC3	-3218	-3210	-	6.08	TGCAATTA
SAMD8	-3232	-3224	-	8.20	ACTAATTA
LOC100068320	-3240	-3232	-	8.20	ACTAATTA
LOC100068041	-3214	-3206	-	6.71	AATAATTA
AK9	-3213	-3205	+	8.17	TAATTGCT
SLC22A16	-3213	-3205	+	7.09	TAATTGAT
DHRS11	-3239	-3231	-	6.10	TCTAATTA
ACYP2	-3202	-3194	-	7.09	AACAATTA
CHAC2	-3209	-3201	+	8.20	TAATTAGT
GPR75	-3223	-3215	+	7.79	TAATTACT
C15H2orf73	-3223	-3215	-	7.09	ATCAATTA
YWHAQ	-3234	-3226	-	8.20	ACTAATTA
DCDC2C	-3230	-3222	+	6.44	TAATTAAC
WDR31	-3229	-3221	+	8.31	TAATTGGC
JCHAI.	-3200	-3192	-	6.71	ATTAATTA
R.PC3	-3224	-3216	-	6.06	CTCAATTA

```
                R.PC3   -3205   -3197   -       6.82    GACAATTA
                CAMKMT  -3200   -3192   -       6.71    AATAATTA
                SLC3A1  -3227   -3219   -       6.06    CTCAATTA

    21  MA0139.1        CTCF    17.2051081365   0.644893727864  -926:-892
 2.55083011703e-05
                list of hits
                ADK     -0906   -887    -       7.39    TACCGCTCCCTTCCGGCCC
                LOC100068208    -0892   -873    +       5.20    CAACCCCCGGGGGTACAA
                LOC100067994    -0919   -900    -       3.75    TTGTTCCCACTTGAGGGAG
                AFG1L   -0924   -905    -       4.05    TGCCAGCCTCTTCTGAACA
                FOXO3   -0918   -899    -       3.47    GGGGGCCCTCCGCCGGGAG
                SES.1   -0907   -888    +       5.50    AGCCCGCCGGAGGGCGGGG
                CEP57L1 -0924   -905    -       3.09    CCCCGCCCTCCGCCGCTCC
                PPIL6   -0896   -877    +       4.11    TCACCAGCAGTTGAGGCTG
                CDC40   -0912   -893    -       5.25    CGCCGCCCCCCACCGCCCC
                SOCS3   -0917   -898    +       4.41    GGGCCTCCTGGGGCCGCGA
                TMEM235 -0926   -907    +       4.18    GGACCAACAGGGGCCTGCC
                AFMID   -0925   -906    +       7.05    GGGCCACCGGAGGACAGGG
                MRM1    -0926   -907    +       5.39    CCCCCACTAGGGGCTAGAA
                GPR75   -0904   -885    -       4.12    CAATTCCTTCTGCTGCAAA
                MSG.1   -0905   -886    -       7.30    CACAGCCACCTTGGGGCTA
                ODC1    -0919   -900    +       4.27    AGGGCAGAAGGGGCCAGAG
                ISM1    -0892   -873    -       3.90    GGCCCCCATCTGGAGCCGG
                RGS3    -0920   -901    -       4.47    GTCCTCCCTCTCCTGAGAA
                AMBP    -0895   -876    -       3.96    CAACTCCACTTCCTGGCTG
                AK.A    -0922   -903    +       4.42    GATCCTCCAGAGGCCACGC
                LOC100050034    -0904   -885    +       3.77    TGGACTGTTGAGGACACAG
                UTP3    -0897   -878    -       5.71    CGGTGCCTTCTGCAGCCAC
                SQLE    -0912   -893    -       5.70    TCCTTTCTTCTTCTGGCAA
                TRIB1   -0923   -904    -       3.81    CAGCTCCAACCGGAGGTCC
                C25H9orf43      -0894   -875    +       3.78    GGCGCGGGAGATGGCGCTC
                COX20   -0903   -884    -       4.31    CGGCGCCCGCCCCTGGCAC

    22  MA0062.2        GABPA   13.3349243401   0.647453861192  -570:-532
 2.77112231052e-05
                list of hits
                LOC100061588    -0542   -531    -       7.49    GTCACTTCCTC
                LOC100068208    -0568   -557    -       6.53    GGGGCTTCCTG
                KLHL2   -0545   -534    +       4.73    GAGGAAGGGGA
                SES.1   -0550   -539    +       5.13    CCGGAATCGGC
                SLC22A16        -0553   -542    +       4.54    CCCGAAGCGCC
                SLC22A16        -0542   -531    -       6.53    GGGGCTTCCTG
                CD164   -0537   -526    -       7.96    ACAACTTCCGC
                CYTH1   -0553   -542    +       6.04    CGGGAAGCGGC
                DHRS11  -0568   -557    -       6.08    TTCTCTTCCTG
                PSME4   -0570   -559    +       11.43   CCGGAAGTGAC
                PSME4   -0548   -537    +       6.61    CCGGAACCGCT
                KC.S3   -0548   -537    +       5.02    GCGGAACTACA
                MSG.1   -0562   -551    +       4.59    GCGGAAAGGAG
                RRM2    -0537   -526    -       5.34    GCCGCTCCCGG
                COLEC11 -0559   -548    +       8.64    CAGGAAGTGGT
                TRAPPC12        -0535   -524    +       4.72    CAGGAAGAAAA
                DCDC2C  -0567   -556    -       5.07    CTCCCTTCCGT
                RYBP    -0532   -521    -       4.62    CCCACGTCCGC
                POLE3   -0563   -552    +       7.29    CCGGAAAAGGC
                AMBP    -0564   -553    -       7.35    TCAGCTTCCGC
                MOB1B   -0539   -528    -       5.77    CCCACTTCCTT
                GRSF1   -0544   -533    -       6.52    CGTGTTTCCGG
                E.AM    -0534   -523    -       5.30    GTCATTTCCTC
                CATSPERE        -0538   -527    -       4.73    ATCACTTACGG
                ZBTB18  -0570   -559    -       5.00    GCCGCTTTCGG

    23  MA0161.1        NFIC    6.98267924778   0.5685040611    -2359:-2329
 2.89684028946e-05
                list of hits
                DUSP13  -2359   -2353   -       6.73    TGCCAA
                SMPD2   -2337   -2331   +       6.73    TTGGCA
                LOC102147786    -2348   -2342   +       6.73    TTGGCA
```

```
                TMEM235 -2354   -2348   -       6.73    TGCCAA
                GG.BP2  -2353   -2347   +       6.73    TTGGCA
                PSME4   -2329   -2323   +       6.73    TTGGCA
                ISM1    -2354   -2348   -       6.73    TGCCAA
                ALAD    -2356   -2350   -       6.73    TGCCAA
                T.C     -2357   -2351   -       6.73    TGCCAA
                GC      -2348   -2342   +       6.73    TTGGCA
                LOC100049851    -2339   -2333   -       6.73    TGCCAA
                R.PC3   -2354   -2348   -       6.73    TGCCAA
                TRIB1   -2333   -2327   +       6.73    TTGGCA

    24  MA0109.1        Hltf    7.46926527425   0.397046551108  -1917:-1878
3.04110693034e-05
                list of hits
                SMC3    -1882   -1872   -       6.03    TCATAAGTAT
                RBM20   -1878   -1868   -       5.76    GCATATGGAA
                LOC100066444    -1879   -1869   +       5.97    AACCTTAGGA
                LOC100066472    -1912   -1902   -       5.66    ACATATGTGT
                LOC106780946    -1909   -1899   +       6.42    AACCTTTTGT
                CEP57L1 -1881   -1871   +       6.11    TTACTTATAT
                ZBTB24  -1883   -1873   +       5.88    AAACATTTGT
                E.GASE  -1906   -1896   -       5.69    CTACAAGGTT
                LGALS3BP        -1914   -1904   +       5.66    AAACATTTCC
                USP36   -1884   -1874   +       6.02    CACCTTATGA
                ACACA   -1917   -1907   +       5.66    ATACATTTGT
                .OL10   -1903   -1893   -       5.77    ACCTAAGGAA
                KLF11   -1883   -1873   +       5.73    AAACATTTAC
                ADAM17  -1889   -1879   -       5.76    ATATATGGGA
                RPS7    -1881   -1871   +       5.76    TTACTTTTCT
                ADI1    -1901   -1891   -       5.74    ACACAAGGTC
                PDZR.3  -1913   -1903   +       6.27    AACCTTTTAA
                PDZR.3  -1883   -1873   +       6.09    ATCCTTATGG
                DIPK2A  -1907   -1897   +       5.83    TCACTTATAT
                ZFP37   -1896   -1886   -       5.66    CTAAAAGGAA
                PRPF4   -1901   -1891   -       5.84    TTAAAAGGAA
                POLE3   -1888   -1878   +       5.69    AACCTTTGGA
                POLE3   -1878   -1868   -       5.72    AGACATGGTT
                T.FSF8  -1899   -1889   +       5.70    GCCCTTTTAT
                T.C     -1892   -1882   -       5.68    TCAAAAGTTC
                TEX48   -1912   -1902   -       5.79    ACACAAGTTT
                TEX48   -1888   -1878   +       6.00    TTCCTTTTCT
                LOC100050100    -1879   -1869   +       6.00    TTCCTTTTGT
                GC      -1907   -1897   -       5.97    GGAAAAGTTT
                UTP3    -1899   -1889   -       5.79    AGACAAGTTT
                SLC4A4  -1916   -1906   +       5.80    AAACTTAGAC
                SLC4A4  -1891   -1881   -       5.75    ATAAAAGTAC
                LOC100049851    -1891   -1881   +       5.83    TACCTTAGAA
                SLC3A1  -1903   -1893   +       5.79    CTCCATATAT
                ZBTB18  -1917   -1907   -       5.69    TGCAAAGGTT

    25  MA0060.1        NFYA    12.9251246242   0.522629311625  -3255:-3198
3.13629191277e-05
                list of hits
                DUSP13  -3201   -3185   -       4.96    GCTTCCATTGGAGCCT
                SAMD8   -3225   -3209   -       6.81    AGGCTTATTGGTCTGT
                LOC100068191    -3233   -3217   -       7.39    GTGTTTATTGGCCATC
                LOC100066501    -3245   -3229   +       5.26    TTTGGCAAATCAAAAG
                MICAL1  -3255   -3239   -       5.42    CTAATGATTGGATATT
                ZBTB24  -3239   -3223   -       5.64    GAGTCCATTGGAGGAG
                ZBTB24  -3200   -3184   -       9.11    TTTTTCATTGGCTGAA
                CD164   -3207   -3191   +       11.49   ATTGGCCAATCAGAAG
                D.AH17  -3253   -3237   +       5.34    AGCAGCCGATGAGCCT
                MRM1    -3253   -3237   -       6.49    CCCATCATTGGTCTCT
                LHX1    -3200   -3184   -       4.85    CGCTCGATTGGACCAG
                ACACA   -3255   -3239   -       4.84    TTTCCCATTGGGGTAC
                KLF11   -3235   -3219   +       7.83    TTCGGCCAATAGCATC
                WDR31   -3233   -3217   -       8.94    GTCCTAATTGGCTGAC
                AMBP    -3217   -3201   +       5.58    CTGCCCCAATGGGGTG
                COL27A1 -3236   -3220   -       6.29    TCTGTTATTGGATGTT
```

LOC100051562	-3252	-3236	-	6.12	TAAATCATTGGTCATT
PPM1B	-3224	-3208	+	5.70	GATTACCAATGACCAA
C25H9orf43	-3217	-3201	-	5.55	TCACTCATTTGTCAAC
ZBTB18	-3198	-3182	-	6.11	AACATTATTGGCTCTT
