# Supplementary material for: Identification and Functional Annotation of Genes Related to Horses’ Performance: From GWAS to Post-GWAS
Source: Animals (Basel). 2020 Jul 10;10(7):1173. doi: 10.3390/ani10071173 (PMC7401650; doi:10.3390/ani10071173)
Supplement: Supplementary file 1 [file animals-10-01173-s001.zip › supplementarymaterials/TFM_explorer_out/tfmexplorer_group4.pdf]

```
;TFM Explorer
;Date               : Wed May 13 06:29:37 2020
;Scanned location   : -3000:300
;Scanned sequences  : None (2 sequences)
;Scanned matrices   : (130 matrices)
;Parameters         : minsize= 30, maxsize= 1500, ratio=3.0, top=25
;
; headers
;    1    rank                window rank
;    2    matrix              matrix name
;    3    tf                  transcription factor name
;    4    info                information of content of the matrix
;    5    gc                  gc content of the matrix (G+C)%
;    6    location            location of the window (relatively to TSS)
;    7    pvalue              pvalue of the window

    1   MA0006.1        Arnt::Ahr       9.53241699429   0.715277771155  -288:-55
 1.68156944989e-07
                list of hits
                SORCS3  -0288   -282    +       7.37    TGCGTG
                SORCS3  -0277   -271    -       7.05    CACGCG
                SORCS3  -0219   -213    -       7.37    CACGCA
                SORCS3  -0193   -187    -       7.37    CACGCA
                SORCS3  -0069   -63     -       7.37    CACGCA
                SORCS3  -0061   -55     -       7.05    CACGCG
                SORCS3  -0055   -49     -       7.37    CACGCA
```
