# Supplementary material for: Identification and Functional Annotation of Genes Related to Horses’ Performance: From GWAS to Post-GWAS
Source: Animals (Basel). 2020 Jul 10;10(7):1173. doi: 10.3390/ani10071173 (PMC7401650; doi:10.3390/ani10071173)
Supplement: Supplementary file 1 [file animals-10-01173-s001.zip › supplementarymaterials/TFM_explorer_out/tfmexplorer_group5.pdf]

```
;TFM Explorer
;Date               : Wed May 13 06:33:31 2020
;Scanned location   : -3275:0
;Scanned sequences  : None (178 sequences)
;Scanned matrices   : (130 matrices)
;Parameters         : minsize= 30, maxsize= 1500, ratio=3.0, top=25
;
; headers
;    1    rank               window rank
;    2    matrix             matrix name
;    3    tf                 transcription factor name
;    4    info               information of content of the matrix
;    5    gc                 gc content of the matrix (G+C)%
;    6    location           location of the window (relatively to TSS)
;    7    pvalue             pvalue of the window

1   MA0089.1        NFE2L1::MafG    7.93814372721   0.421568617371  -486:-313
2.80696885964e-10
                list of hits
                MIR331  -0471   -465    +       6.66    CATGAC
                MIR135A-2       -0334   -328    -       6.66    GTCATG
                ART4    -0485   -479    +       6.66    CATGAC
                GRI.2B  -0380   -374    +       6.66    CATGAC
                GSG1    -0444   -438    +       6.57    GATGAC
                MIR9170 -0321   -315    +       6.66    CATGAC
                PIP5K1B -0313   -307    -       6.66    GTCATG
                TJP2    -0391   -385    +       6.66    CATGAC
                TJP2    -0313   -307    -       6.66    GTCATG
                MAMDC2  -0364   -358    +       6.66    CATGAC
                LOC100052901    -0476   -470    -       6.57    GTCATC
                LOC100052951    -0458   -452    -       6.57    GTCATC
                LOC100052951    -0397   -391    -       6.57    GTCATC
                LOC100052638    -0397   -391    -       6.57    GTCATC
                LOC100052638    -0336   -330    -       6.57    GTCATC
                LOC100052689    -0412   -406    -       6.57    GTCATC
                LOC100052689    -0351   -345    -       6.57    GTCATC
                _LOC100049805   -0469   -463    -       6.57    GTCATC
                LOC100052229    -0414   -408    -       6.57    GTCATC
                LOC100052292    -0486   -480    -       6.57    GTCATC
                SPRYD7  -0374   -368    +       6.57    GATGAC
                R.ASEH2B        -0410   -404    -       6.57    GTCATC
                PSMB2   -0336   -330    -       6.57    GTCATC
                ZMYM4   -0473   -467    +       6.66    CATGAC
                ZMYM4   -0464   -458    -       6.66    GTCATG
                ZBTB6   -0349   -343    -       6.66    GTCATG
                OR1K1   -0428   -422    +       6.66    CATGAC
                LOC100071189    -0367   -361    +       6.66    CATGAC
                LOC100071189    -0360   -354    -       6.66    GTCATG
                LOC100071244    -0444   -438    +       6.66    CATGAC
                LOC100067520    -0442   -436    +       6.66    CATGAC
                LOC100071251    -0387   -381    +       6.66    CATGAC
                LOC100071227    -0439   -433    +       6.66    CATGAC
                LOC100071264    -0348   -342    +       6.66    CATGAC
                LOC100071264    -0339   -333    +       6.57    GATGAC
                _LOC100071297   -0354   -348    -       6.57    GTCATC
                OR1.1   -0459   -453    +       6.57    GATGAC
                LOC100071407    -0428   -422    +       6.66    CATGAC
                LOC100071445    -0336   -330    -       6.57    GTCATC
                LOC100071469    -0465   -459    -       6.57    GTCATC

2   MA0130.1        ZNF354C 8.95800882514   0.614583333333  -707:-634
1.05430731724e-09
                list of hits
                PLX.C1  -0686   -680    +       6.86    CTCCAC
                TMCC3   -0638   -632    +       6.86    CTCCAC
                SLCO1C1 -0650   -644    -       7.01    GTGGAT
                LOC100065342    -0677   -671    -       6.86    GTGGAG
                DGKD    -0668   -662    +       7.01    ATCCAC
```

EIF4E2	-0663	-657	+	7.01	ATCCAC
CHR.G	-0707	-701	-	6.86	GTGGAG
SOX5	-0672	-666	-	6.86	GTGGAG
SMCO3	-0695	-689	+	6.86	CTCCAC
ART4	-0638	-632	-	7.01	GTGGAT
MGP	-0680	-674	+	7.01	ATCCAC
H4-16	-0651	-645	+	7.01	ATCCAC
FOXP1	-0648	-642	-	7.01	GTGGAT
DMRT3	-0641	-635	-	6.86	GTGGAG
FOXD4	-0634	-628	+	7.01	ATCCAC
APBA1	-0668	-662	-	6.86	GTGGAG
MAMDC2	-0636	-630	-	6.86	GTGGAG
SMC2	-0698	-692	+	6.86	CTCCAC
LOC100052951	-0679	-673	+	6.86	CTCCAC
LOC100052638	-0662	-656	+	6.86	CTCCAC
LOC100052689	-0674	-668	+	6.86	CTCCAC
LOC100052689	-0634	-628	+	6.86	CTCCAC
_LOC100049805	-0691	-685	+	6.86	CTCCAC
LOC100052229	-0679	-673	+	6.86	CTCCAC
LOC100052229	-0639	-633	+	6.86	CTCCAC
VPS36	-0695	-689	+	6.86	CTCCAC
.EK5	-0683	-677	-	6.86	GTGGAG
WDFY2	-0645	-639	+	6.86	CTCCAC
R.ASEH2B	-0672	-666	+	7.01	ATCCAC
TE.M4	-0690	-684	-	6.86	GTGGAG
AGO4	-0695	-689	-	7.01	GTGGAT
AGO1	-0664	-658	-	6.86	GTGGAG
_CSF3R	-0650	-644	-	6.86	GTGGAG
STK40	-0700	-694	-	6.86	GTGGAG
LSM10	-0644	-638	+	7.01	ATCCAC
CLSP.	-0660	-654	-	7.01	GTGGAT
TFAP2E	-0699	-693	+	6.86	CTCCAC
ZBTB6	-0652	-646	+	7.01	ATCCAC
RC3H2	-0704	-698	-	6.86	GTGGAG
_LOC100071170	-0668	-662	-	6.86	GTGGAG
LOC100071338	-0666	-660	+	6.86	CTCCAC
LOC100071486	-0706	-700	+	7.01	ATCCAC
FBXO5	-0651	-645	+	6.86	CTCCAC

3 MA0031.1 FOXD1 11.9264176032 0.27500000177 -1242:-1137
1.991759584e-08

list of hits

.DUFA12	-1228	-1220	+	6.85	GTAAATAT
MIR135A-2	-1171	-1163	+	5.88	GTAAATAG
SLCO1A2	-1241	-1233	-	6.80	ATGTTGAC
SLCO1A2	-1167	-1159	-	6.72	TTGCTTAC
IAPP	-1157	-1149	+	6.91	ATAAACAT
.EU2	-1161	-1153	+	6.91	TTAAACAT
.EU2	-1153	-1145	-	5.88	CTGGTTAC
.EU2	-1145	-1137	-	6.72	TTGCTTAC
SOX5	-1198	-1190	+	5.94	CTAAACAG
ERP27	-1237	-1229	-	6.91	ATGTTTAG
H2AJ	-1208	-1200	-	6.72	TTATTTAC
PLBD1	-1173	-1165	-	6.72	TTTTTTAC
PROK2	-1171	-1163	-	9.69	ATGTTTAC
DMRT2	-1142	-1134	+	6.85	GTAACCAT
_KA.K1	-1223	-1215	-	5.94	CTGTTTAT
DOCK8	-1230	-1222	-	6.72	TTATTTAC
DOCK8	-1193	-1185	+	6.72	GTAACCAA
LOC100052901	-1152	-1144	-	6.72	TTTTTTAC
LOC100052638	-1165	-1157	-	5.94	CTGTTTAG
LOC100052689	-1212	-1204	-	6.91	ATGTTTAG
LOC100052689	-1176	-1168	-	5.94	CTGTTTAG
LOC100147522	-1233	-1225	-	5.94	CTGTTTAG
CKAP2	-1215	-1207	-	6.72	TTTTTTAC
.EK3	-1225	-1217	-	6.72	TTTTTTAC
.EK3	-1181	-1173	-	6.80	ATGTTGAC
ATP7B	-1213	-1205	+	6.85	GTAAAAAT
ATP7B	-1192	-1184	-	6.85	ATTTTTAC

```
                CCDC70  -1150   -1142   -       6.77    TTGTTTAA
                DHRS12  -1190   -1182   +       8.72    GTAAACAG
                _I.TS6  -1164   -1156   -       5.94    CTGTTTAT
                KC.RG   -1162   -1154   +       9.56    GTAAACAA
                MIR15A  -1187   -1179   -       5.94    CTGTTTAG
                DLEU7   -1137   -1129   +       5.88    GTAAATAG
                _CSF3R  -1137   -1129   +       6.77    ATAAACAA
                SH3D21  -1232   -1224   +       6.91    ATAAACAT
                SH3D21  -1181   -1173   +       6.85    GTAAAAAT
                AGO3    -1220   -1212   +       6.77    CTAAACAA
                PDCL    -1233   -1225   +       6.72    GTAAAAAA
                LOC100071212    -1193   -1185   +       6.77    TTAAACAA
                LOC100071244    -1187   -1179   -       6.91    ATGTTTAA
                LOC100067520    -1235   -1227   -       6.91    ATGTTTAG
                LOC100071251    -1167   -1159   -       6.91    ATGTTTAG
                OR1B1   -1137   -1129   +       5.94    TTAAACAG
                LOC100071332    -1166   -1158   +       6.85    GTAAATAT
                LOC100071407    -1225   -1217   -       6.91    ATGTTTAG
                LOC100071420    -1152   -1144   -       6.72    TTATTTAC
                LOC100071420    -1138   -1130   +       6.91    TTAAACAT
                LOC100071469    -1158   -1150   -       6.91    ATGTTTAG
                MTRF1L  -1223   -1215   +       6.72    GTAACCAA
                VIP     -1166   -1158   -       6.77    TTGTTTAA
                RGS17   -1242   -1234   -       6.85    ATATTTAC

        4       MA0095.1        YY1     8.10118848971   0.509803925951  -199:-141
5.01648758677e-08
                list of hits
                MIR135A-2       -0186   -180    +       6.58    GCCATC
                C6H12orf60      -0199   -193    +       6.58    GCCATC
                C6H12orf60      -0173   -167    +       6.58    GCCATC
                TMEM252 -0157   -151    +       6.58    GCCATC
                APBA1   -0169   -163    -       6.42    GATGGT
                LOC100052951    -0150   -144    +       6.42    ACCATC
                _LOC100049805   -0166   -160    +       6.42    ACCATC
                ADPRHL2 -0159   -153    +       6.58    GCCATC
                OR1K1   -0156   -150    +       6.58    GCCATC
                LOC100071212    -0159   -153    +       6.58    GCCATC
                LOC100071244    -0198   -192    +       6.58    GCCATC
                LOC100071244    -0141   -135    +       6.42    ACCATC
                LOC100071218    -0159   -153    +       6.58    GCCATC
                LOC100067520    -0198   -192    +       6.58    GCCATC
                LOC100067520    -0141   -135    +       6.58    GCCATC
                LOC100071251    -0142   -136    +       6.58    GCCATC
                LOC100071227    -0198   -192    +       6.58    GCCATC
                LOC100071227    -0141   -135    +       6.58    GCCATC
                OR1Q1   -0171   -165    +       6.58    GCCATC
                LOC100071311    -0153   -147    +       6.58    GCCATC
                LOC100071407    -0187   -181    +       6.58    GCCATC
                LOC100071420    -0144   -138    +       6.42    ACCATC
                LOC100071448    -0144   -138    +       6.42    ACCATC

        5       MA0070.1        PBX1    14.6408950838   0.310185187496  -53:-17 6.40650017578e-08
                list of hits
                MIR135A-2       -0043   -31     -       4.39    GTTGATTCATCT
                PDE3A   -0017   -5      +       4.89    ATATGAATCATA
                MGP     -0021   -9      +       4.30    CCATCAATTTAA
                LOC100052689    -0025   -13     +       5.95    TGATCCATCCAT
                LOC100052526    -0050   -38     -       4.18    TTTGGTTGTTGT
                LOC100052229    -0023   -11     +       6.13    CCATCCATCCAC
                MIR3613 -0050   -38     +       6.29    AGCACAATCAAA
                MIR3613 -0018   -6      +       3.68    CCCACAATCTTT
                MIR16-2 -0041   -29     -       5.95    ATTGAATGAGAT
                MIR15A  -0031   -19     -       5.76    TTTTATTGATAA
                THRAP3  -0025   -13     +       3.27    CAACCACTCAAA
                STRBP   -0051   -39     -       3.89    ATTGTTTGTTGT
                GPR21   -0029   -17     +       5.76    AGATGAATCAAA
                _LOC100071297   -0036   -24     -       5.49    TATGCTGGATGC
                LOC100071311    -0018   -6      -       3.66    TATGCTGGGTGT
```

```
                LOC100071329    -0053   -41     +       3.44    TAAACATGCAAA
                LOC100071329    -0037   -25     +       7.84    GGATCAATCCAT
                LOC100071329    -0021   -9      -       3.66    TATGCTGGGTGT
                LOC100071346    -0025   -13     +       4.86    AAGTCCATCAAG
                LOC100071365    -0037   -25     +       5.11    GAGTCAATCCAT
                LOC100071402    -0033   -21     +       4.23    CAGTCAATCCTC
                LOC100071469    -0021   -9      +       4.65    TCACCAATCTGT
                MYCT1   -0047   -35     -       3.44    ACTGCATGATTT

    6   MA0063.1        Nkx2-5  8.26972553332   0.218487398433  -2735:-2636
 2.37774186268e-07
                list of hits
                CEP83   -2715   -2708   +       6.35    TTAATAG
                CEP83   -2637   -2630   -       7.00    CACTTAA
                TMCC3   -2717   -2710   -       6.18    CAATTGA
                MIR135A-2       -2685   -2678   -       6.06    AAATTAA
                SLCO1C1 -2733   -2726   -       7.35    CAATTAA
                .EU2    -2657   -2650   +       6.18    TCAATTG
                SOX5    -2658   -2651   -       7.00    CACTTAA
                SOX5    -2636   -2629   -       6.75    CACTTAT
                WBP11   -2723   -2716   +       6.10    ATAATAG
                ART4    -2670   -2663   -       6.75    CACTTAT
                H2AJ    -2638   -2631   -       7.00    CACTTAA
                ATF7IP  -2721   -2714   -       7.00    CACTTAA
                GRI.2B  -2641   -2634   -       5.99    CTCTTAA
                PGM5    -2735   -2728   -       6.75    CACTTAT
                TMEM252 -2717   -2710   +       6.35    TTAATAG
                FAM122A -2733   -2726   +       7.00    TTAAGTG
                APBA1   -2723   -2716   -       7.00    CACTTAA
                APBA1   -2642   -2635   -       6.10    CTATTAT
                C23H9orf135     -2673   -2666   -       7.00    CACTTAA
                C23H9orf135     -2643   -2636   -       6.35    CTATTAA
                MAMDC2  -2706   -2699   -       6.06    AAATTAA
                SMC2    -2641   -2634   -       6.06    AAATTAA
                _KA.K1  -2697   -2690   -       6.06    AAATTAA
                DOCK8   -2667   -2660   -       7.00    CACTTAA
                LOC100052901    -2683   -2676   +       6.06    TTAATTT
                CKAP2   -2706   -2699   +       6.06    TTAATTT
                WDFY2   -2694   -2687   -       7.00    CACTTAA
                SERPI.E3        -2656   -2649   -       6.06    AAATTAA
                DLEU7   -2704   -2697   +       5.99    TTAAGAG
                DLEU7   -2643   -2636   +       5.93    ACAATTG
                C1H15orf41      -2673   -2666   +       5.93    ACAATTG
                TE.M4   -2657   -2650   -       6.06    AAATTAA
                C2H1orf216      -2695   -2688   +       7.10    ATAATTG
                PSMB2   -2704   -2697   -       6.06    AAATTAA
                STRBP   -2646   -2639   -       6.06    AAATTAA
                ZBTB6   -2700   -2693   -       6.18    CAATTGA
                ZBTB26  -2648   -2641   -       6.06    AAATTAA
                LOC100071212    -2686   -2679   +       5.93    ACAATTG
                LOC100067520    -2715   -2708   -       7.00    CACTTAA
                LOC100071251    -2695   -2688   +       7.35    TTAATTG
                OR1B1   -2658   -2651   -       7.35    CAATTAA
                LOC100071270    -2683   -2676   -       6.06    AAATTAA
                LOC100071270    -2653   -2646   +       6.35    TTAATAG
                _LOC100071297   -2642   -2635   -       5.99    CTCTTAA
                LOC100071346    -2665   -2658   -       6.06    AAATTAA
                LOC100071358    -2727   -2720   +       6.06    TTAATTT
                LOC100071358    -2682   -2675   -       5.99    CTCTTAA
                LOC100071407    -2731   -2724   -       6.35    CTATTAA
                LOC100071486    -2679   -2672   -       5.93    CAATTGT

    7   MA0047.2        Foxa2   13.2676175109   0.315129053384  -1814:-1777
 4.73018047045e-07
                list of hits
                CEP83   -1810   -1798   +       5.11    TCTTTACATTTA
                CEP83   -1780   -1768   +       4.98    TATTTATTTGAG
                MIR135A-2       -1793   -1781   -       5.47    GGTGAGCCAACA
                SOX5    -1800   -1788   -       7.75    GAAGAATAAACA
```

GRI.2B	-1791	-1779	-	5.43	GAAAAGAAAACA
GSG1	-1810	-1798	+	4.99	TAATTACACAGG
_PTAR1	-1791	-1779	-	5.74	GTTAAGTAAAAA
C23H9orf135	-1795	-1783	-	8.18	GTAAAATAAACA
C23H9orf135	-1783	-1771	-	5.92	CATGAACAAACA
_KA.K1	-1811	-1799	-	6.44	TTTGTGTACACA
DOCK8	-1779	-1767	-	7.04	CCTTAGTACACA
.EK5	-1814	-1802	-	4.99	AGGGTGTAAACA
CCDC70	-1792	-1780	-	6.12	TTTGTGCAAATA
EBPL	-1809	-1797	-	6.91	TAACTGTAAATA
C1H15orf41	-1791	-1779	+	4.92	TGTTGAAACTGG
DPH6	-1810	-1798	-	5.77	ATTTGGTAAATA
AGO4	-1787	-1775	-	5.67	CATTTGTACACA
.CD.	-1796	-1784	+	10.47	TGTTTACTTAAT
GPR21	-1812	-1800	-	4.98	GATCTATCAACA
ZBTB6	-1795	-1783	+	7.40	TATTTATTTATT
RC3H2	-1798	-1786	+	10.75	TGTTTACATAAG
OR1K1	-1812	-1800	+	5.92	TATTTGCAGAGA
LOC100071212	-1795	-1783	-	5.63	AATATGTAAAGA
LOC100071258	-1801	-1789	-	5.59	TCAATACAAATA
LOC100071258	-1786	-1774	-	7.40	AATAAATAAATA
_LOC100071297	-1793	-1781	+	5.54	TATTGACTTGTC
_LOC100071297	-1777	-1765	+	5.01	TATTGGCTGTGT
LOC100071346	-1813	-1801	+	5.93	TGTTTGTTTAAT
LOC100071420	-1806	-1794	+	9.36	TATTTACATAAT
LOC100071420	-1790	-1778	-	5.42	CTAACGTAAATA
LOC100071420	-1777	-1765	+	8.90	TGTTTACTGAAG
LOC100071445	-1791	-1779	+	8.32	TATTGACTTATC
MTRF1L	-1785	-1773	+	5.68	TGTTTTCATTTG

8 MA0027.1 En1 6.42106531013 0.445454545184 -1667:-1565
1.03926279007e-06

list of hits

PLX.C1	-1570	-1559	+	6.35	TGGTGGTGCTC
.R2C1	-1606	-1595	-	5.61	AACCATAACTT
PDE3A	-1566	-1555	+	6.42	AAGTTATTGCC
SLCO1A2	-1653	-1642	-	5.66	GAAAACAACCC
IAPP	-1577	-1566	+	6.64	ACGTGGTGGTC
SLCO1B3	-1613	-1602	-	6.30	GGACTCTACTC
SLCO1C1	-1643	-1632	-	5.89	GCCAACCACTT
USP40	-1667	-1656	+	6.64	AGGTGGTGTCC
I.PP5D	-1605	-1594	-	5.86	GAACTTCACTT
.EU2	-1637	-1626	+	5.61	AAGTTATGTTT
.GEF	-1571	-1560	+	5.61	AAAGAGTGCCC
KC.J13	-1653	-1642	-	6.45	GAACACTCCTT
MGP	-1643	-1632	+	5.73	GAGTTATTTCC
PGM5	-1646	-1635	+	5.89	CAGTGGTTTCC
FAM189A2	-1616	-1605	+	5.90	AAGTAATGACC
C23H9orf135	-1627	-1616	+	7.83	AAGTAGTGGTC
LOC100052526	-1589	-1578	+	5.79	ACATGGTGCTC
VPS36	-1580	-1569	+	6.17	TAGTAGTTCCT
CKAP2	-1628	-1617	-	5.61	GACCATTCCTT
MIR3613	-1664	-1653	+	5.86	AAATAATTGCC
MIR3613	-1632	-1621	-	5.86	GGAATCTATTT
AGO4	-1660	-1649	-	5.61	AAAAATTACTT
AGO4	-1588	-1577	+	6.01	GAATTGTGTCC
_CSF3R	-1617	-1606	-	5.88	AGGAACAACTA
STK40	-1635	-1624	-	5.61	AGGCATCACTT
C2H1orf216	-1667	-1656	+	6.17	AAGTGGTTTTT
PDCL	-1602	-1591	+	6.30	AAGTAGAATCC
LOC100071244	-1656	-1645	+	5.77	AAGTTGTGGCA
LOC100071244	-1614	-1603	-	5.77	GAGTACTCCTT
LOC100071258	-1622	-1611	-	6.35	GGCAACTACCA
OR1B1	-1659	-1648	-	6.86	GAGCACCACTC
_LOC100071297	-1565	-1554	-	5.73	GAGAATAACTC
LOC100071329	-1624	-1613	+	6.18	TAGTAGTGGGC
LOC100071358	-1628	-1617	-	5.79	GAAAACTATCT
LOC100071438	-1619	-1608	-	6.17	AGCAACAACTT
LOC100071445	-1566	-1555	-	5.73	GAGAATAACTC

```
                LOC100071460    -1568   -1557   -       6.42    GAAAATCACTT
                MTRF1L  -1588   -1577   +       5.86    AAATAATTTCC
                VIP     -1657   -1646   -       6.42    GAGAACCATTT

    9   MA0089.1        NFE2L1::MafG    7.93814372721   0.421568617371  -2886:-2737
 1.55300729324e-06
                list of hits
                TMCC3   -2886   -2880   -       6.66    GTCATG
                TMCC3   -2756   -2750   +       6.66    CATGAC
                TMCC3   -2747   -2741   -       6.66    GTCATG
                FGD6    -2826   -2820   +       6.66    CATGAC
                LOC100065342    -2806   -2800   +       6.57    GATGAC
                CHR.D   -2753   -2747   +       6.57    GATGAC
                PRSS56  -2781   -2775   +       6.66    CATGAC
                EIF4E2  -2885   -2879   +       6.57    GATGAC
                ARHGDIB -2860   -2854   +       6.66    CATGAC
                MGP     -2853   -2847   +       6.66    CATGAC
                GRI.2B  -2839   -2833   +       6.66    CATGAC
                DMRT3   -2738   -2732   -       6.57    GTCATC
                SMC2    -2802   -2796   +       6.66    CATGAC
                DOCK8   -2884   -2878   +       6.57    GATGAC
                LOC100052689    -2747   -2741   -       6.66    GTCATG
                LOC100052292    -2787   -2781   +       6.57    GATGAC
                LOC100147522    -2783   -2777   -       6.57    GTCATC
                .EK3    -2838   -2832   +       6.66    CATGAC
                .EK5    -2822   -2816   +       6.66    CATGAC
                _I.TS6  -2846   -2840   +       6.66    CATGAC
                EBPL    -2775   -2769   +       6.57    GATGAC
                STK40   -2876   -2870   -       6.57    GTCATC
                STK40   -2787   -2781   +       6.57    GATGAC
                AGO3    -2875   -2869   -       6.57    GTCATC
                AGO3    -2791   -2785   -       6.66    GTCATG
                AGO3    -2769   -2763   -       6.66    GTCATG
                MAP7D1  -2880   -2874   -       6.66    GTCATG
                CLSP.   -2737   -2731   +       6.57    GATGAC
                C2H1orf216      -2778   -2772   +       6.57    GATGAC
                C2H1orf216      -2767   -2761   +       6.57    GATGAC
                .CD.    -2849   -2843   +       6.57    GATGAC
                RABGAP1 -2842   -2836   +       6.66    CATGAC
                RC3H2   -2757   -2751   +       6.66    CATGAC
                LOC100071189    -2851   -2845   -       6.66    GTCATG
                LOC100071189    -2801   -2795   -       6.57    GTCATC
                LOC100071212    -2860   -2854   +       6.57    GATGAC
                _LOC100071297   -2878   -2872   +       6.57    GATGAC
                LOC100071332    -2821   -2815   -       6.66    GTCATG
                LOC100071402    -2800   -2794   -       6.57    GTCATC
                LOC100071420    -2774   -2768   -       6.66    GTCATG
                LOC100071445    -2882   -2876   +       6.57    GATGAC
                MTRF1L  -2743   -2737   +       6.66    CATGAC

    10  MA0152.1        NFATC2  9.85861508532   0.346153842592  -2799:-2758
 2.85459229986e-06
                list of hits
                PLX.C1  -2791   -2784   -       7.20    AGGAAAA
                USP40   -2797   -2790   -       8.69    TGGAAAA
                ATG16L1 -2766   -2759   +       8.69    TTTTCCA
                PLBD1   -2772   -2765   -       8.69    TGGAAAA
                PLBD1   -2758   -2751   +       6.81    ATTTCCA
                CBWD1   -2792   -2785   -       7.20    AGGAAAA
                FOXD4   -2786   -2779   +       6.92    TTTTCCC
                PGM5    -2784   -2777   +       6.42    GTTTCCA
                C23H9orf135     -2798   -2791   -       7.20    AGGAAAA
                C23H9orf135     -2764   -2757   +       7.20    TTTTCCT
                LOC100052526    -2799   -2792   +       6.81    ATTTCCA
                LOC100052526    -2790   -2783   +       8.69    TTTTCCA
                LOC100052526    -2777   -2770   +       6.81    ATTTCCA
                .EK5    -2762   -2755   +       6.37    TCTTCCA
                ATP7B   -2758   -2751   -       6.92    GGGAAAA
                _I.TS6  -2790   -2783   -       6.42    TGGAAAC
```

```
                TRIM13  -2799   -2792   +       6.37    TGTTCCA
                DLEU7   -2776   -2769   +       6.92    TTTTCCC
                C1H15orf41      -2783   -2776   +       6.92    TTTTCCC
                OSCP1   -2799   -2792   -       6.92    GGGAAAA
                AGO1    -2758   -2751   +       6.37    TGTTCCA
                PSMB2   -2798   -2791   -       6.92    GGGAAAA
                RABGAP1 -2789   -2782   -       6.37    TGGAAGA
                LOC100071251    -2791   -2784   +       6.37    TCTTCCA
                OR1Q1   -2774   -2767   +       7.20    TTTTCCT
                LOC100071270    -2770   -2763   -       6.42    TGGAAAC
                _LOC100071297   -2791   -2784   -       6.92    GGGAAAA
                _LOC100071297   -2759   -2752   -       8.69    TGGAAAA
                LOC100071346    -2778   -2771   -       6.92    GGGAAAA
                LOC100071365    -2760   -2753   +       6.92    TTTTCCC
                LOC100071420    -2781   -2774   -       7.20    AGGAAAA
                LOC100071445    -2794   -2787   -       7.20    AGGAAAA
                FBXO5   -2796   -2789   +       7.20    TTTTCCT
                FBXO5   -2761   -2754   -       8.69    TGGAAAA
                MYCT1   -2777   -2770   -       6.37    TGGAAGA

    11  MA0148.1        FOXA1   12.5328023695   0.332119902252  -2939:-2901
3.26060686909e-06
                list of hits
                PLX.C1  -2937   -2926   -       9.06    AAGGGTAAACA
                CEP83   -2936   -2925   +       5.44    TGTTTGTAATT
                TMCC3   -2923   -2912   -       5.73    ATGAATCAACA
                PDE3A   -2923   -2912   +       6.55    TGTTTCCTCTC
                LOC100065342    -2930   -2919   -       6.22    AATAATAAACA
                GUCY2C  -2907   -2896   +       5.23    TGTTTGCAGGT
                PDE6H   -2917   -2906   +       6.21    TATTTTCTCTT
                H2AJ    -2926   -2915   +       6.44    TGGTTACTTAG
                GRI.2B  -2902   -2891   +       7.53    TATTTACTGAG
                EMP1    -2933   -2922   -       10.18   GAAAGTAAACA
                PROK2   -2939   -2928   +       5.31    TGTCTACCCTG
                _KA.K1  -2903   -2892   +       5.80    TATTTACAAAT
                _LOC100049805   -2929   -2918   -       6.62    AAAGACAAACA
                .EK3    -2938   -2927   +       6.28    TGTTAGCTCAG
                ATP7B   -2901   -2890   +       5.41    TGTTTACCTCT
                MIR16-2 -2911   -2900   -       6.52    ATGGATAAACA
                _RCBTB1 -2918   -2907   +       7.90    TGTTTGTTTAT
                R.ASEH2B        -2913   -2902   +       5.35    TTTTTGCATAT
                LSM10   -2907   -2896   -       11.37   CAGAGTAAACA
                THRAP3  -2906   -2895   +       6.52    TGTTTGATCTG
                CLSP.   -2937   -2926   +       5.85    TGTTTTCTAAT
                CLSP.   -2920   -2909   -       5.91    TAAGGAAAACA
                C2H1orf216      -2928   -2917   -       5.45    CTAAATACACA
                .CD.    -2911   -2900   +       8.77    TATTTACATAG
                ZMYM4   -2914   -2903   -       5.21    ACAAGCCAACA
                STRBP   -2915   -2904   -       5.60    GAAAGCAAAAA
                ZBTB26  -2930   -2919   -       6.39    AAAAGCAAACT
                PDCL    -2932   -2921   -       6.28    ATGAGTAAACT
                PDCL    -2921   -2910   +       9.67    TGTTTGCATAT
                OR1B1   -2905   -2894   +       7.40    TGTTGACCTAG
                _LOC100071297   -2904   -2893   +       5.44    TGTTTGTAATT
                LOC100071338    -2939   -2928   +       9.91    TGTTTGCATTT
                OR1.1   -2913   -2902   -       6.12    GTAGATAAACA
                LOC100071445    -2908   -2897   +       5.44    TGTTTGTAATT
                MYCT1   -2925   -2914   +       5.46    TGTTTATTAAC
                RGS17   -2921   -2910   -       7.07    AAGAGGAAACA

    12  MA0006.1        Arnt::Ahr       9.53241699429   0.715277771155  -455:-418
4.636342681e-06
                list of hits
                GIGYF2  -0455   -449    -       7.05    CACGCG
                ARHGDIB -0429   -423    -       7.05    CACGCG
                RYBP    -0448   -442    +       7.05    CGCGTG
                PROK2   -0453   -447    +       7.05    CGCGTG
                FOXP1   -0450   -444    +       7.37    TGCGTG
                FAM122A -0453   -447    -       7.05    CACGCG
```

```
                APBA1   -0418   -412    -       7.05    CACGCG
                DOCK8   -0436   -430    -       7.05    CACGCG
                ALG11   -0423   -417    -       7.05    CACGCG
                _I.TS6  -0452   -446    +       7.05    CGCGTG
                FAM124A -0419   -413    -       7.37    CACGCA
                TE.M4   -0418   -412    +       7.37    TGCGTG
                AGO4    -0426   -420    -       7.37    CACGCA
                MRPS15  -0435   -429    -       7.37    CACGCA
                TFAP2E  -0445   -439    +       7.37    TGCGTG
                STRBP   -0431   -425    +       7.05    CGCGTG
                RGS17   -0418   -412    -       7.05    CACGCG

    13  MA0031.1        FOXD1   11.9264176032   0.27500000177   -2939:-2902
 4.91922843776e-06
                list of hits
                PLX.C1  -2933   -2925   +       9.69    GTAAACAT
                FGD6    -2924   -2916   -       5.88    CTGGTTAC
                SLCO1A2 -2924   -2916   +       5.94    CTAAACAG
                LOC100065342    -2926   -2918   +       6.77    ATAAACAA
                I.PP5D  -2921   -2913   +       6.77    ATAAACAA
                CHR.G   -2922   -2914   +       6.77    TTAAACAA
                GRI.2B  -2922   -2914   +       6.72    GTAAAAAA
                GRI.2B  -2903   -2895   -       6.85    ATATTTAC
                EMP1    -2929   -2921   +       9.56    GTAAACAA
                _KA.K1  -2904   -2896   -       6.85    ATATTTAC
                CKAP2   -2909   -2901   +       6.91    TTAAACAT
                ATP7B   -2902   -2894   -       9.69    ATGTTTAC
                WDFY2   -2934   -2926   +       6.77    TTAAACAA
                SERPI.E3        -2939   -2931   +       6.91    ATAAACAT
                MIR16-2 -2907   -2899   +       6.77    ATAAACAA
                _RCBTB1 -2915   -2907   -       6.77    TTGTTTAT
                DPH6    -2935   -2927   +       5.88    GTAACCAG
                LSM10   -2903   -2895   +       9.56    GTAAACAA
                .CD.    -2912   -2904   -       6.85    ATATTTAC
                GPR21   -2937   -2929   -       6.72    TTTTTTAC
                OR1B1   -2906   -2898   -       6.80    ATGTTGAC
                LOC100071346    -2924   -2916   -       5.94    CTGTTTAT
                OR1.1   -2909   -2901   +       6.91    ATAAACAT
                MYCT1   -2926   -2918   -       6.91    ATGTTTAT

    14  MA0083.1        SRF     17.9647717767   0.465579714133  -2988:-2923
 7.1966303339e-06
                list of hits
                EFHD1   -2976   -2964   +       4.10    TCCCAAAGATGG
                GUCY2C  -2948   -2936   -       2.99    TTATATGTGGGA
                DMRT3   -2946   -2934   +       3.25    GTCCCTATAGGG
                PGM5    -2955   -2943   -       2.67    GCAACTATGGGC
                FAM189A2        -2927   -2915   +       3.67    TGCCACATTTGG
                APBA1   -2944   -2932   -       3.12    GCAAATTTGGGT
                LOC100052526    -2964   -2952   +       5.25    TCCAATATAAAG
                LOC100052292    -2988   -2976   -       4.40    CTTTATCAGGGC
                LOC100052292    -2969   -2957   +       4.44    GACCACATAGGG
                OSCP1   -2956   -2944   -       10.33   TCTTATAAGGGC
                TRAPPC3 -2927   -2915   +       3.41    TCCCAAAGAAGG
                MAP7D1  -2926   -2914   -       3.41    CCTTCTTTGGGA
                ADPRHL2 -2951   -2939   -       3.41    CCTTATTTGTGA
                STRBP   -2958   -2946   +       5.92    GCCCCTATTTGA
                GPR21   -2962   -2950   +       3.05    TCCAATATCTGG
                ZBTB6   -2927   -2915   +       3.64    ATCCAAATATGA
                RC3H2   -2973   -2961   +       4.26    TGCCATATTTAG
                LOC100071258    -2939   -2927   +       3.19    GACCATAAAAAA
                OR1B1   -2923   -2911   +       2.49    AACATCATATGG
                LOC100071438    -2982   -2970   -       3.28    CCTTCTATGCTC
                LOC100071448    -2983   -2971   -       4.59    GCTAATAGGGGC

    15  MA0140.1        Tal1::Gata1     11.2966184617   0.45067769915   -3108:-3030
 7.57917797919e-06
                list of hits
                FGD6    -3033   -3015   -       6.10    TTTATCATAAGTCTGAAT
```

PRSS56	-3108	-3090	+	6.75	GGTTCGAACTCAGATAAA
GUCY2C	-3054	-3036	+	6.04	TTTTATGAGGAAGATTAG
PDE6H	-3093	-3075	+	6.35	CGTGACACCATAGATAGG
C6H12orf60	-3095	-3077	-	6.80	CTTATCTGCAAAGTTCTT
MGP	-3046	-3028	-	5.98	ATTATCTAAAACCCAAAT
H2AJ	-3094	-3076	+	9.00	GTGGCCCCCACAGATAAT
EMP1	-3069	-3051	-	5.62	TTTATCTTTCAAAAATAT
PIP5K1B	-3030	-3012	-	6.22	CGTATCACCTCCCTTCAG
FX.	-3104	-3086	+	7.06	GTGAAGTGGGGTGATAGG
FX.	-3044	-3026	-	5.78	ATTATCTGGAGGTAGACA
LOC100052901	-3047	-3029	+	6.96	CCGTGTTATTTAGATAAC
LOC100052951	-3108	-3090	+	7.14	CTAGCAACAAAAGATAAA
LOC100052951	-3055	-3037	+	6.66	CTGTGTACTAAAGATAGT
LOC100052689	-3071	-3053	+	6.27	CAACAGCCAAAAGATAAG
LOC100052582	-3035	-3017	-	8.52	TCTATCTCCTGCCACCAA
LOC100052526	-3077	-3059	-	6.14	CCTATCAGTAGTTGCATG
_LOC100049805	-3060	-3042	+	5.87	TTGTGTACTAAAGATAGT
LOC100052292	-3068	-3050	-	6.71	TCTATCTCCATGTCCCCA
_I.TS6	-3100	-3082	-	6.31	TTTATCTAGGGACCACTG
KP.A3	-3035	-3017	+	6.40	TTGCTCCCCAGAGATTAA
MIR15A	-3059	-3041	-	6.76	ATTATCAAAAAAAAACAG
_CSF3R	-3060	-3042	+	6.58	ACCCCTGTGGCAGATAAG
THRAP3	-3102	-3084	-	5.90	CTAATCTTCCTCAAAAAT
THRAP3	-3031	-3013	+	5.68	GGTAGAATTTCAGATAAA
CLSP.	-3090	-3072	+	5.94	GTGTGATATAATGATAGA
_LOC100071297	-3053	-3035	-	8.00	CTTATCTGAAAAATAGAG
LOC100071338	-3056	-3038	-	5.84	TTTATCTTTATTCTAATA
LOC100071407	-3093	-3075	+	8.20	AGTGAAACTGCAGATAAG
LOC100071445	-3060	-3042	-	8.00	CTTATCTGAAAAATAGAG
LOC100071469	-3032	-3014	-	6.35	CTAATCTTTCTCAAAAAA

16 MA0081.1 SPIB 9.06007130057 0.466472323191 -2920:-2872
8.68057919725e-06

list of hits

.DUFA12	-2913	-2906	-	7.28	TTCCTCA
PDE3A	-2920	-2913	-	7.82	TTCCTCT
SLCO1B3	-2912	-2905	-	7.82	TTCCTCT
.EU2	-2880	-2873	+	7.82	AGAGGAA
EFHD1	-2881	-2874	-	7.82	TTCCTCT
CHR.D	-2879	-2872	+	7.09	ACAGGAA
CHR.G	-2907	-2900	-	7.82	TTCCTCT
C6H12orf60	-2872	-2865	-	6.55	TTCCTGA
ART4	-2901	-2894	-	7.82	TTCCTCT
PLBD1	-2905	-2898	+	6.60	AGGGGAA
PROK2	-2880	-2873	-	7.09	TTCCTGT
DMRT2	-2910	-2903	-	6.55	TTCCTGA
FOXD4	-2912	-2905	-	6.60	TTCCCCT
PIP5K1B	-2904	-2897	+	6.55	TCAGGAA
C23H9orf135	-2903	-2896	+	7.28	TGAGGAA
C23H9orf135	-2895	-2888	+	7.09	ACAGGAA
WDFY2	-2894	-2887	+	7.28	TGAGGAA
TRIM13	-2902	-2895	-	7.82	TTCCTCT
SPRYD7	-2898	-2891	-	7.28	TTCCTCA
EBPL	-2919	-2912	+	7.09	ACAGGAA
AGO1	-2890	-2883	-	7.09	TTCCTGT
STK40	-2899	-2892	+	7.82	AGAGGAA
THRAP3	-2872	-2865	-	7.82	TTCCTCT
TRAPPC3	-2875	-2868	-	6.60	TTCCCCT
LOC100071189	-2901	-2894	+	6.60	AGGGGAA
_LOC100071297	-2872	-2865	+	7.82	AGAGGAA
LOC100071332	-2916	-2909	-	6.55	TTCCTGA
LOC100071420	-2898	-2891	+	6.60	AGGGGAA
LOC100071445	-2876	-2869	+	7.82	AGAGGAA
RGS17	-2920	-2913	+	7.82	AGAGGAA

17 MA0070.1 PBX1 14.6408950838 0.310185187496 -511:-446
1.12664644452e-05

list of hits

MIR135A-2	-0461	-449	-	4.80	GTTGAAGGATAA

PDE3A	-0484	-472	-	7.32	TTTGCTTGAGGA
CHR.G	-0493	-481	-	6.35	AAAGATGGATGG
WBP11	-0490	-478	+	6.75	GTGTCAATCAAC
ARHGDIB	-0472	-460	+	4.45	ATATCACTCAAG
PDE6H	-0466	-454	-	6.19	ATTGATTGTGTG
ATF7IP	-0484	-472	+	7.32	ACCTCATTCAAA
DMRT2	-0460	-448	-	6.97	AATGATTGAGTC
FOXD4	-0478	-466	-	6.19	CTGGATTGTTGG
MAMDC2	-0491	-479	-	7.32	TTTGCTTGAGGA
THSD1	-0460	-448	+	5.95	TCATCAAGCCAC
VPS36	-0488	-476	-	4.30	TTTAATTGGTGG
TRIM13	-0511	-499	+	7.38	ACAACAATCCAA
EBPL	-0500	-488	+	3.99	AAATCAAGCCTC
LSM10	-0450	-438	-	6.17	AAAGCTTGATGA
SH3D21	-0507	-495	-	6.69	CTTGAATGATTC
CLSP.	-0454	-442	+	4.83	CCGCCAATCCAA
PSMB2	-0508	-496	+	5.95	TCCTCAAGCAAG
_LOC100071170	-0459	-447	-	3.89	ATGGTTTGATGA
LOC100071227	-0499	-487	+	4.12	ACAACAATGAAA
LOC100071270	-0491	-479	+	7.67	TCCTCAATCAGA
_LOC100071297	-0502	-490	+	6.85	TCATCAATAAAA
LOC100071365	-0491	-479	-	5.05	CTAGATGGATGC
LOC100071358	-0458	-446	+	5.82	ATATCTATCAAA
LOC100071358	-0446	-434	+	4.06	ACATGCATCAAA
LOC100071486	-0449	-437	-	3.83	ATTAATTGAGGA
VIP	-0500	-488	-	4.46	TATGCTTGGTAC

18 MA0077.1 SOX9 9.07881468797 0.358187139655 -2665:-2593 1.19689911211e-05

list of hits

.DUFA12	-2593	-2584	+	7.54	ATACAATGG
FGD6	-2630	-2621	+	9.56	GAACAATGG
PDE3A	-2594	-2585	-	6.46	CTATTGGTC
I.PP5D	-2644	-2635	-	6.07	CCATTATTC
.EU2	-2659	-2650	+	5.74	TATCAATTG
GIGYF2	-2665	-2656	-	5.95	GGATTGTTC
CHR.D	-2595	-2586	-	6.57	CCATGGTTT
CHR.G	-2626	-2617	-	6.92	CCATTGGTC
WBP11	-2600	-2591	+	6.98	TAACAATGA
C6H12orf60	-2599	-2590	+	6.00	AAACAATAT
EMP1	-2665	-2656	-	5.94	CCTTTGTTA
FOXP1	-2646	-2637	+	5.98	AAACAATTC
CBWD1	-2634	-2625	+	6.71	GAACAATGT
FX.	-2615	-2606	+	6.28	GAACAAAAG
_PTAR1	-2598	-2589	+	7.77	AAACAATTG
ALG11	-2593	-2584	+	7.81	AATCAATGG
MIR3613	-2608	-2599	-	6.25	CAATTGTAC
DLEU7	-2645	-2636	+	7.77	AAACAATTG
AGO1	-2657	-2648	-	5.83	CCAATGTTT
THRAP3	-2617	-2608	+	5.83	AAACACTGG
KIAA0319L	-2605	-2596	-	6.23	TAATTGTTC
STRBP	-2640	-2631	+	7.52	AAACAATGC
RABGAP1	-2665	-2656	+	6.02	AATCAATGA
LOC100071212	-2615	-2606	+	6.65	AAACTATAG
LOC100071264	-2620	-2611	+	7.06	AAACAATAC
_LOC100071297	-2638	-2629	+	5.94	TAACAAAGG
LOC100071329	-2653	-2644	+	6.30	CAACAATGC
LOC100071329	-2608	-2599	-	5.75	GCATTGTCC
LOC100071346	-2657	-2648	+	7.00	TTACAATGG
LOC100071438	-2648	-2639	-	6.46	ACATTGTTT
LOC100071420	-2647	-2638	-	6.02	CTTTTGTTT
LOC100071445	-2643	-2634	+	5.94	TAACAAAGG
LOC100071469	-2634	-2625	-	6.68	CTATTGTGT
VIP	-2640	-2631	-	5.83	CCAATGTTT
VIP	-2624	-2615	-	6.02	CTTTTGTTT

19 MA0108.2 TBP 10.0863285279 0.376692369239 -2342:-2300 1.33914105896e-05

list of hits

```
                .R2C1   -2342   -2327   -       6.61    TCTGCCTATTTATCC
                SLCO1C1 -2310   -2295   +       6.29    GTTTAAAAATGCTTG
                SOX5    -2340   -2325   +       6.62    TTATAAATTCCAGGC
                GUCY2C  -2338   -2323   -       7.69    GGGGGCCTTTTATCG
                H4-16   -2314   -2299   -       6.87    AGCAGTATTTTATAC
                GSG1    -2308   -2293   -       6.58    ATTGGCCTTTTATGT
                EIF4E3  -2330   -2315   +       7.08    ATATAAAAATGCTTA
                EIF4E3  -2303   -2288   +       6.60    TTATAAAAGAAAATG
                FAM189A2        -2342   -2327   +       6.08    GTATAAAACTATCTA
                FAM189A2        -2302   -2287   -       6.21    TCTGTTTCTATATAC
                APBA1   -2335   -2320   +       6.65    CCATATAAAAGAGCG
                _KA.K1  -2319   -2304   +       9.51    CTATAAAAAGGAATG
                LOC100052951    -2317   -2302   -       7.70    TGATTCCTTTTATAT
                LOC100052689    -2308   -2293   -       5.86    TTGGAGTATATATAT
                _LOC100049805   -2324   -2309   -       7.70    TGATTCCTTTTATAT
                LOC100052229    -2329   -2314   +       8.81    CTATAAAAAGAATGA
                FAM124A -2335   -2320   -       6.04    AGGGGCCATTTGTAC
                ZMYM4   -2323   -2308   +       7.61    CTATAAATCCCTCTG
                LOC100071264    -2341   -2326   +       7.58    CTATAAAATGAAGAC
                LOC100071346    -2329   -2314   +       8.08    CTATATAAAACAGCA
                LOC100071346    -2304   -2289   +       7.65    CCATAAAAAGAAGGA
                OR1.1   -2318   -2303   +       8.56    CTATAAAAGAAAGTG
                LOC100071420    -2338   -2323   +       7.11    GTTTAAAAACACAGT
                RGS17   -2300   -2285   -       6.21    TGGTTGTATTTAAAG

    20  MA0153.1        HNF1B   16.8212281893   0.22222222512   -1028:-962
1.5201192336e-05
                list of hits
                PLX.C1  -0971   -959    -       5.10    AATAAATATTAG
                FGD6    -1016   -1004   +       3.13    TTAGTGAGTATC
                MIR331  -1024   -1012   -       5.57    GTTTAACATGAA
                PDE3A   -1009   -997    -       3.73    GTCAAATTTGAA
                MGP     -1028   -1016   -       7.46    GTTAATTATCAA
                FOXP1   -0962   -950    +       4.23    CTAGTTGTTAAC
                DMRT2   -1012   -1000   +       6.04    TTAAAGGTTAAC
                CBWD1   -0997   -985    -       3.41    GTTATCCATGGA
                LOC100052951    -1007   -995    -       10.72   GTTAATTAGTAG
                LOC100052689    -0963   -951    -       7.97    GTCAATTAGTGG
                _LOC100049805   -1019   -1007   -       7.97    GTCAATTAGTGG
                LOC100052229    -0968   -956    -       7.97    GTCAATTAGTGG
                VPS36   -1026   -1014   -       5.69    GTTATACATGAA
                .EK5    -1025   -1013   +       3.18    CTAGTATTTATT
                WDFY2   -0991   -979    -       8.82    GTTAACTATGGG
                EBPL    -0967   -955    -       4.20    GTTGCCTATTAA
                GPR21   -1004   -992    +       7.28    TCAATATTTAAG
                ZBTB6   -0971   -959    +       2.78    TCAGTAAGTCAC
                LOC100071264    -1020   -1008   -       3.82    GATAATCACTGG
                LOC100071264    -0977   -965    -       4.88    GCTACACATTGA
                LOC100071332    -1024   -1012   -       2.65    TTTACTTATGAG
                LOC100071338    -1013   -1001   +       4.10    TTAATGGTTGAG
                LOC100071460    -1027   -1015   -       3.47    TTTAATTAGGAA
                LOC100071469    -0992   -980    -       3.73    AACAAATATTAA
                MTRF1L  -1020   -1008   +       4.53    CCCATATTTTAC
                SY.E1   -0967   -955    -       4.37    GATACTCATTGA
                MYCT1   -0990   -978    +       3.98    TTACTATGTTAC
                RGS17   -0964   -952    +       7.46    TTAATAATTCAC

    21  MA0063.1        Nkx2-5  8.26972553332   0.218487398433  -2488:-2449
1.58384961451e-05
                list of hits
                MIR331  -2487   -2480   +       7.00    TTAAGTG
                SLCO1B3 -2486   -2479   +       7.10    ATAATTG
                DGKD    -2456   -2449   +       7.35    TTAATTG
                GSG1    -2481   -2474   +       6.06    TTAATTT
                GSG1    -2460   -2453   +       6.75    ATAAGTG
                RYBP    -2449   -2442   -       7.00    CACTTAA
                EIF4E3  -2465   -2458   -       7.10    CAATTAT
                FOXP1   -2486   -2479   -       7.35    CAATTAA
                APBA1   -2453   -2446   -       6.06    AAATTAA
```

```
                _KA.K1  -2473   -2466   -       6.06    AAATTAA
                ARL11   -2477   -2470   -       6.10    CTATTAT
                TE.M4   -2449   -2442   +       6.18    TCAATTG
                RC3H2   -2458   -2451   +       6.06    TTAATTT
                _LOC100071297   -2483   -2476   +       6.18    TCAATTG
                LOC100071329    -2477   -2470   +       7.00    TTAAGTG
                LOC100071329    -2458   -2451   +       6.10    ATAATAG
                LOC100071358    -2488   -2481   +       7.00    TTAAGTG
                LOC100071407    -2487   -2480   -       5.99    CTCTTAA
                LOC100071438    -2458   -2451   +       6.18    TCAATTG
                LOC100071420    -2478   -2471   +       6.35    TTAATAG
                LOC100071445    -2488   -2481   +       6.18    TCAATTG
                LOC100071448    -2480   -2473   +       5.99    TTAAGAG
                MTRF1L  -2473   -2466   -       7.00    CACTTAA

    22  MA0136.1        ELF5    8.69309550562   0.429292928841  -1290:-1231
 1.76063872487e-05
                list of hits
                .R2C1   -1241   -1232   -       6.19    AAGGAAGAG
                SLCO1A2 -1261   -1252   +       6.70    GATTTCCTT
                KC.J13  -1278   -1269   -       6.47    AAGGAATTA
                PDE6H   -1233   -1224   -       6.26    AAGGATGAA
                MGP     -1233   -1224   +       6.27    CATTTCCGG
                ATF7IP  -1289   -1280   +       7.41    AATTTCCTG
                EIF4E3  -1265   -1256   -       6.56    CCGGAAATT
                FOXP1   -1256   -1247   +       7.15    TGTTTCCTT
                PGM5    -1249   -1240   -       8.56    AAGGAAATA
                FAM122A -1269   -1260   +       6.13    CATTTCCTA
                PIP5K1B -1290   -1281   -       6.20    GAGGAAACA
                PIP5K1B -1265   -1256   -       6.98    AAGGAAGAA
                STXBP6  -1287   -1278   +       6.82    TTTTTCCTT
                LOC100052638    -1250   -1241   -       6.81    GAGGAAATG
                LOC100052689    -1261   -1252   -       6.81    GAGGAAATG
                CKAP2   -1284   -1275   +       6.18    TTTTTCCTG
                CKAP2   -1231   -1222   +       7.93    CACTTCCTT
                ATP7B   -1258   -1249   +       6.93    TATTTCCTA
                SERPI.E3        -1236   -1227   -       6.30    TAGGAAGTG
                KC.RG   -1246   -1237   -       6.33    AAGGAAAGA
                MIR708  -1246   -1237   +       7.35    TAGTTCCTT
                EVA1B   -1245   -1236   +       7.10    AATTTCCTC
                AGO3    -1288   -1279   -       6.69    CAGGATATT
                C2H1orf216      -1285   -1276   +       6.34    TTCTTCCTG
                C2H1orf216      -1239   -1230   +       6.09    AACTTCCCT
                PSMB2   -1257   -1248   -       6.12    AAGGATCTT
                RABGAP1 -1270   -1261   +       6.97    CACTTCCTC
                RABGAP1 -1236   -1227   +       6.33    TCTTTCCTT
                ZBTB6   -1237   -1228   +       6.20    AAGTTCCTG
                LOC100071189    -1285   -1276   -       6.15    ATGGATGTA
                LOC100071189    -1238   -1229   +       6.64    AGTTTCCTT
                LOC100071270    -1273   -1264   -       7.57    CAGGAAGTT
                LOC100071438    -1238   -1229   +       7.87    TACTTCCGT
                LOC100071420    -1271   -1262   +       6.88    TATATCCTC
                FBXO5   -1276   -1267   -       6.33    AAGGAAAGA
                SY.E1   -1257   -1248   -       6.67    CAGGAAGCA

    23  MA0087.1        Sox5    10.8307137621   0.198757763952  -2646:-2530
 1.92617159936e-05
                list of hits
                FGD6    -2630   -2623   +       7.41    GAACAAT
                I.PP5D  -2566   -2559   -       8.38    ATTGTTT
                .EU2    -2627   -2620   -       8.38    ATTGTTT
                WBP11   -2600   -2593   +       8.38    TAACAAT
                ARHGDIB -2555   -2548   +       7.69    CAACAAT
                C6H12orf60      -2599   -2592   +       8.38    AAACAAT
                SMCO3   -2543   -2536   +       7.41    GAACAAT
                FOXP1   -2646   -2639   +       8.38    AAACAAT
                CBWD1   -2634   -2627   +       7.41    GAACAAT
                PGM5    -2554   -2547   +       7.69    CAACAAT
                _PTAR1  -2598   -2591   +       8.38    AAACAAT
```

```
                DOCK8   -2532   -2525   -       8.38    ATTGTTT
                FAM124A -2557   -2550   -       7.41    ATTGTTC
                MIR16-2 -2593   -2586   +       7.69    CAACAAT
                DLEU7   -2645   -2638   +       8.38    AAACAAT
                AGO1    -2530   -2523   +       8.38    TAACAAT
                TEKT2   -2624   -2617   +       8.38    TAACAAT
                KIAA0319L       -2603   -2596   -       7.41    ATTGTTC
                ZMYM4   -2551   -2544   +       7.69    CAACAAT
                STRBP   -2640   -2633   +       8.38    AAACAAT
                _LOC100071170   -2548   -2541   +       7.69    CAACAAT
                LOC100071189    -2536   -2529   -       8.38    ATTGTTT
                LOC100071258    -2604   -2597   -       7.41    ATTGTTC
                LOC100071227    -2625   -2618   -       8.38    ATTGTTT
                LOC100071264    -2620   -2613   +       8.38    AAACAAT
                LOC100071358    -2600   -2593   -       8.38    ATTGTTA
                LOC100071358    -2586   -2579   -       8.38    ATTGTTA
                LOC100071438    -2646   -2639   -       8.38    ATTGTTT
                LOC100071448    -2557   -2550   +       7.41    GAACAAT
                LOC100071460    -2576   -2569   -       7.69    ATTGTTG
                FBXO5   -2586   -2579   +       8.38    AAACAAT

    24  MA0113.1        NR3C1   14.7489180922   0.432098770583  -317:-282
 1.97336073377e-05
                list of hits
                .DUFA12 -0308   -290    +       6.79    GGGAACCTTCTGTCGTGG
                SLCO1B3 -0311   -293    +       5.93    GAAAGCAGCTTTTACTGC
                ERP27   -0282   -264    -       5.77    TGGGAACAGTTTGATCCC
                ARHGDIB -0283   -265    -       11.18   TTAGAACATTTTTTTTTT
                SMCO3   -0294   -276    +       9.41    GAGAAAATAAATTTCTGT
                TMEM252 -0301   -283    +       5.94    GAGAGCATTCTCTCCCAG
                LOC100052292    -0289   -271    -       4.87    CCTGGTCTTATTGGCCTT
                CKAP2   -0317   -299    -       5.31    TCTGAACTATTTCGTTTT
                CCDC70  -0314   -296    +       4.69    GGTAACAATCATTTCTGA
                MIR15A  -0298   -280    -       5.99    GCAGCACATAATGGTTTG
                DLEU7   -0304   -286    -       4.52    CTCCAAAGTAATTTCCTT
                RC3H2   -0313   -295    -       5.99    CTAGGTCGGGATCCCTCC
                LOC100071251    -0295   -277    -       6.36    CTTGATCAAATTGTCCTT
                _LOC100071297   -0309   -291    +       5.88    GGAAACCACATGTCCAGT
                LOC100071329    -0285   -267    +       4.64    CAGAGCAGTGTGTCCAAG
                LOC100071346    -0286   -268    +       5.28    GGGAGAAACATGAAGTAG
                LOC100071338    -0284   -266    -       6.33    AAAGAACATTCTTCCTCT
                LOC100071402    -0285   -267    +       4.35    CAGAGCAGAGTGTCCAAG
                LOC100071438    -0294   -276    +       7.60    AGAATCAACCAGTCCAGT
                LOC100071448    -0307   -289    -       4.69    CTTGAAAGGATTTTATCT

    25  MA0078.1        Sox17   10.5018372033   0.383512551426  -2872:-2781
 1.98290157695e-05
                list of hits
                FGD6    -2835   -2826   -       7.13    AACAATGGA
                SLCO1C1 -2788   -2779   -       7.87    GACAATAGA
                CHR.G   -2870   -2861   +       6.24    CTCATTGGC
                PDE6H   -2842   -2833   +       8.03    TCCATTGTG
                SMCO3   -2859   -2850   +       7.44    TTCATTGTT
                GSG1    -2850   -2841   +       8.03    TCCATTGTG
                PROK2   -2835   -2826   +       8.10    ATCATTGTG
                PGM5    -2781   -2772   +       8.56    TCCATTGTC
                SMC2    -2865   -2856   +       7.63    AGCATTGTC
                _KA.K1  -2831   -2822   -       6.75    AACAATAAA
                LOC100052951    -2796   -2787   -       7.95    CACAATGAC
                LOC100147522    -2816   -2807   -       6.94    CACAATATC
                ATP7B   -2863   -2854   -       6.19    AACAATATT
                ATP7B   -2800   -2791   -       6.75    AACAATAAA
                TRIM13  -2866   -2857   +       7.09    ACTATTGTG
                KC.RG   -2836   -2827   +       6.26    GGTATTGTG
                MIR16-2 -2831   -2822   -       6.44    AACAATATG
                R.ASEH2B        -2805   -2796   -       7.95    CACAATGAC
                OSCP1   -2785   -2776   +       8.35    TTCATTGTG
                AGO1    -2864   -2855   +       8.03    TCCATTGTG
                C2H1orf216      -2786   -2777   -       7.13    AACAATGGG
```

```
RABGAP1 -2826   -2817   +       6.58    CTAATTGTG
ZBTB6   -2832   -2823   -       6.75    AACAATAAA
LOC100071189    -2791   -2782   -       7.13    AACAATGGA
LOC100071244    -2782   -2773   +       6.58    CTAATTGTG
LOC100071218    -2805   -2796   +       6.54    ACAATTGTC
OR1Q1   -2855   -2846   -       6.39    GACAATTGC
OR1Q1   -2814   -2805   -       7.10    GACAATTAG
OR1B1   -2781   -2772   -       7.34    CACAATATA
LOC100071338    -2872   -2863   -       7.87    GACAATATG
LOC100071338    -2810   -2801   -       6.50    AACAATAAT
LOC100071407    -2848   -2839   -       6.44    AACAATATA
LOC100071438    -2834   -2825   -       7.34    CACAATAGG
LOC100071420    -2820   -2811   -       7.44    AACAATGAA
```
