# Supplementary material for: Identification and Functional Annotation of Genes Related to Horses’ Performance: From GWAS to Post-GWAS
Source: Animals (Basel). 2020 Jul 10;10(7):1173. doi: 10.3390/ani10071173 (PMC7401650; doi:10.3390/ani10071173)
Supplement: Supplementary file 1 [file animals-10-01173-s001.zip › supplementarymaterials/Gene-TF networks/geneTF_network_group7.pdf]

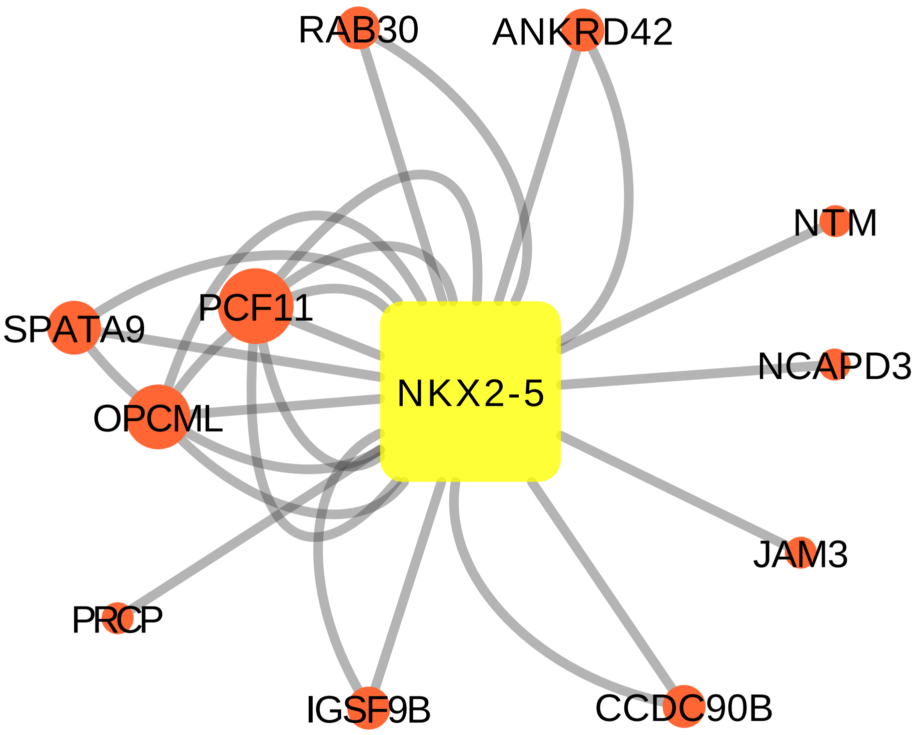

Gene-Transcription Factor (TF) network of genes identified on Group 7. Yellow rounded squares nodes represent the TFs associated with candidate genes (circle nodes) related with horse performance. The node size corresponds to the network analyses (Cytoscape; Shannon et al., 2003) in which the larger nodes correspond to a higher edge density associated with the number of TF binding sites.
