# Supplementary figures and images for: Identification and Functional Annotation of Genes Related to Horses’ Performance: From GWAS to Post-GWAS
Source: Animals (Basel). 2020 Jul 10;10(7):1173. doi: 10.3390/ani10071173 (PMC7401650; doi:10.3390/ani10071173)

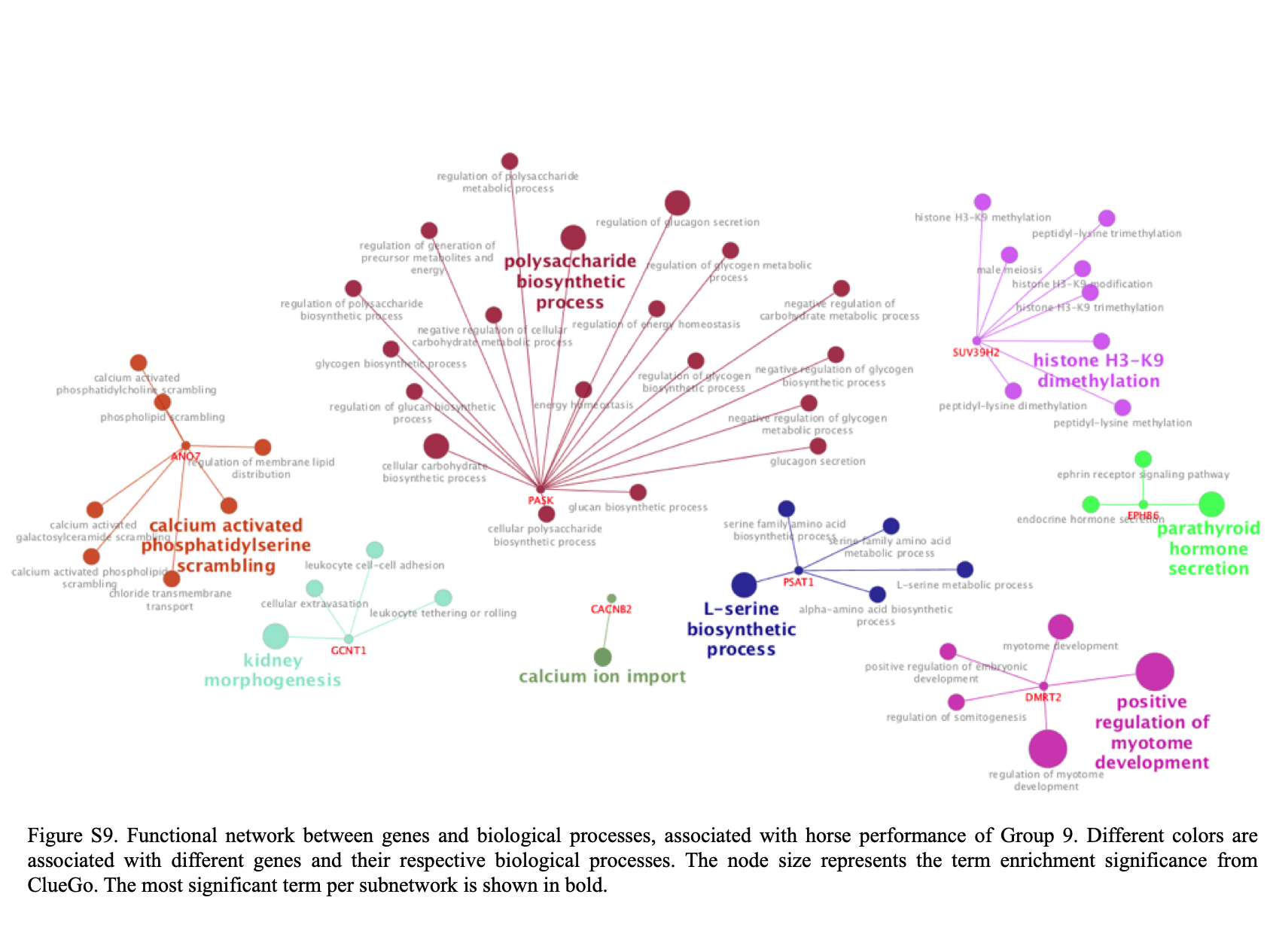

Supplement: Supplementary file 1 [file animals-10-01173-s001.zip › supplementarymaterials/Figure S9.png]

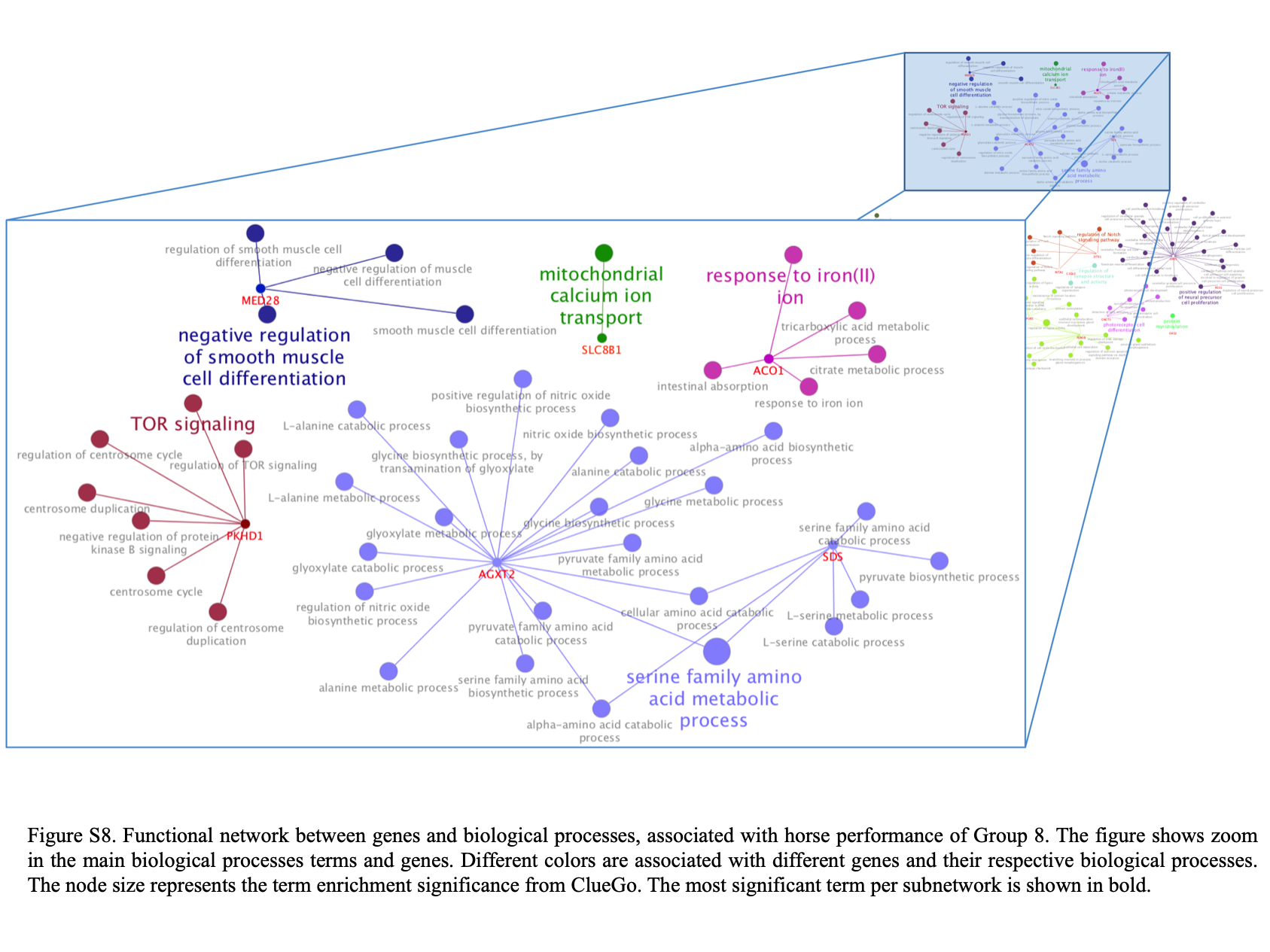

Supplement: Supplementary file 1 [file animals-10-01173-s001.zip › supplementarymaterials/Figure S8.png]

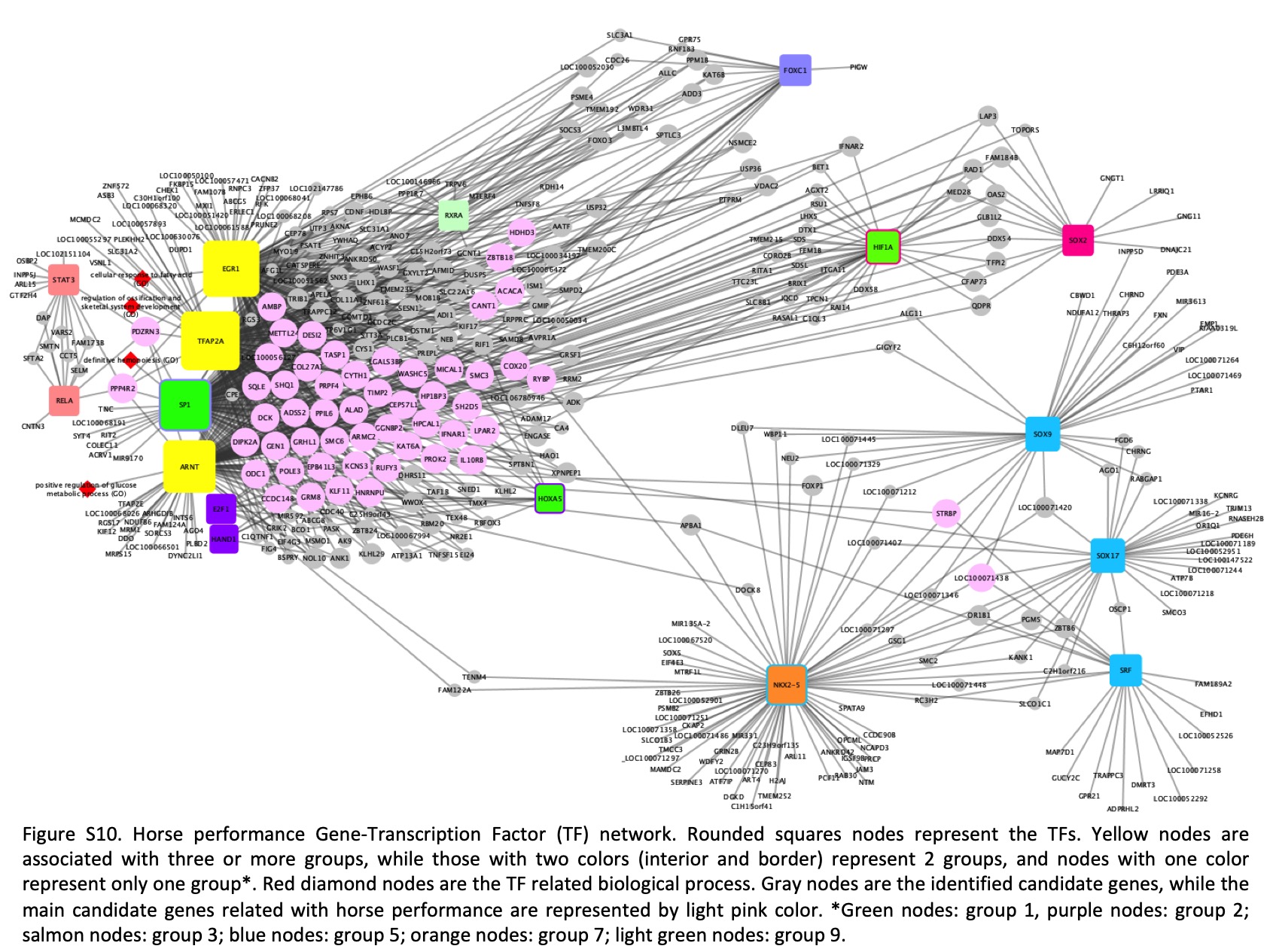

Supplement: Supplementary file 1 [file animals-10-01173-s001.zip › supplementarymaterials/Figure S10.jpg]

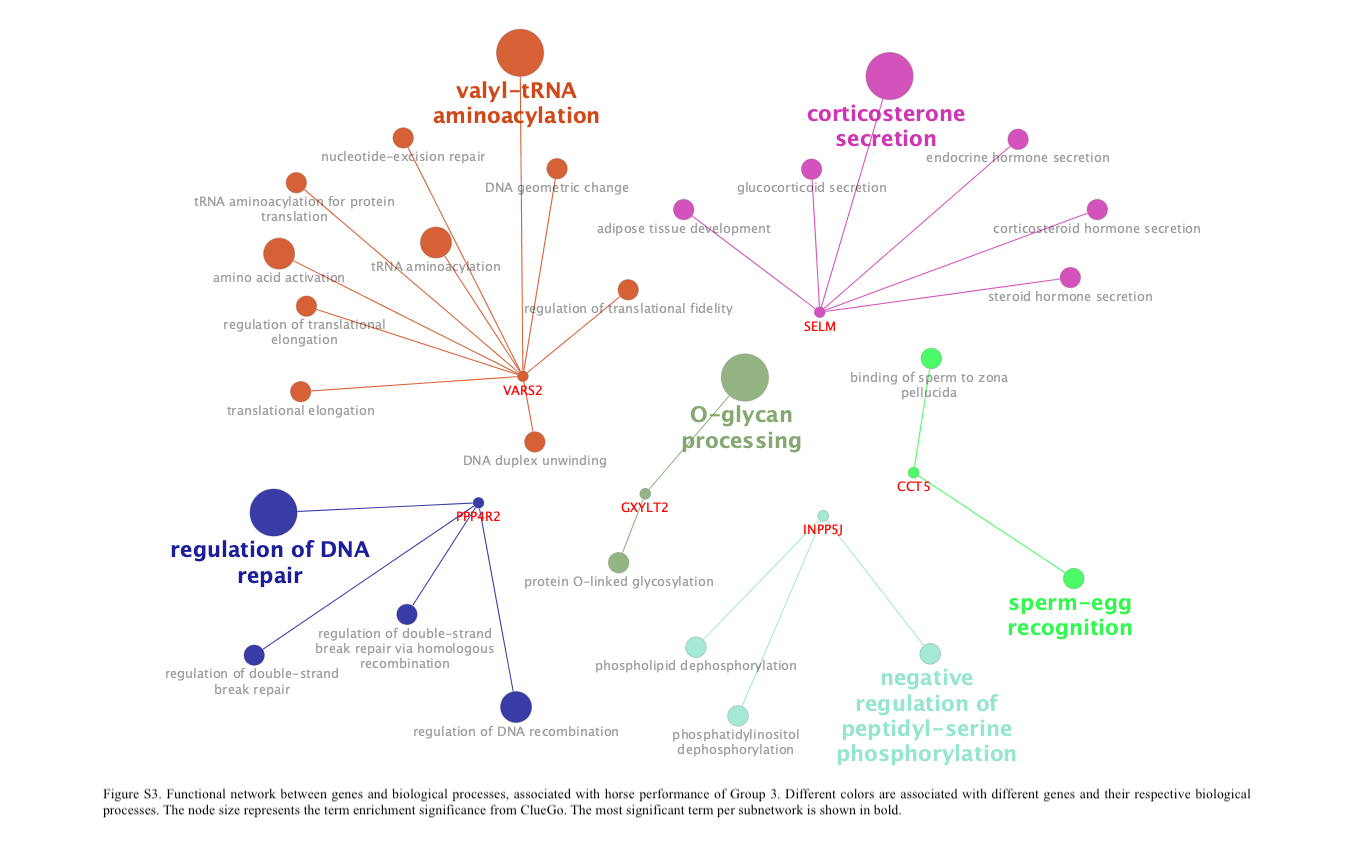

Supplement: Supplementary file 1 [file animals-10-01173-s001.zip › supplementarymaterials/Figure S3.png]

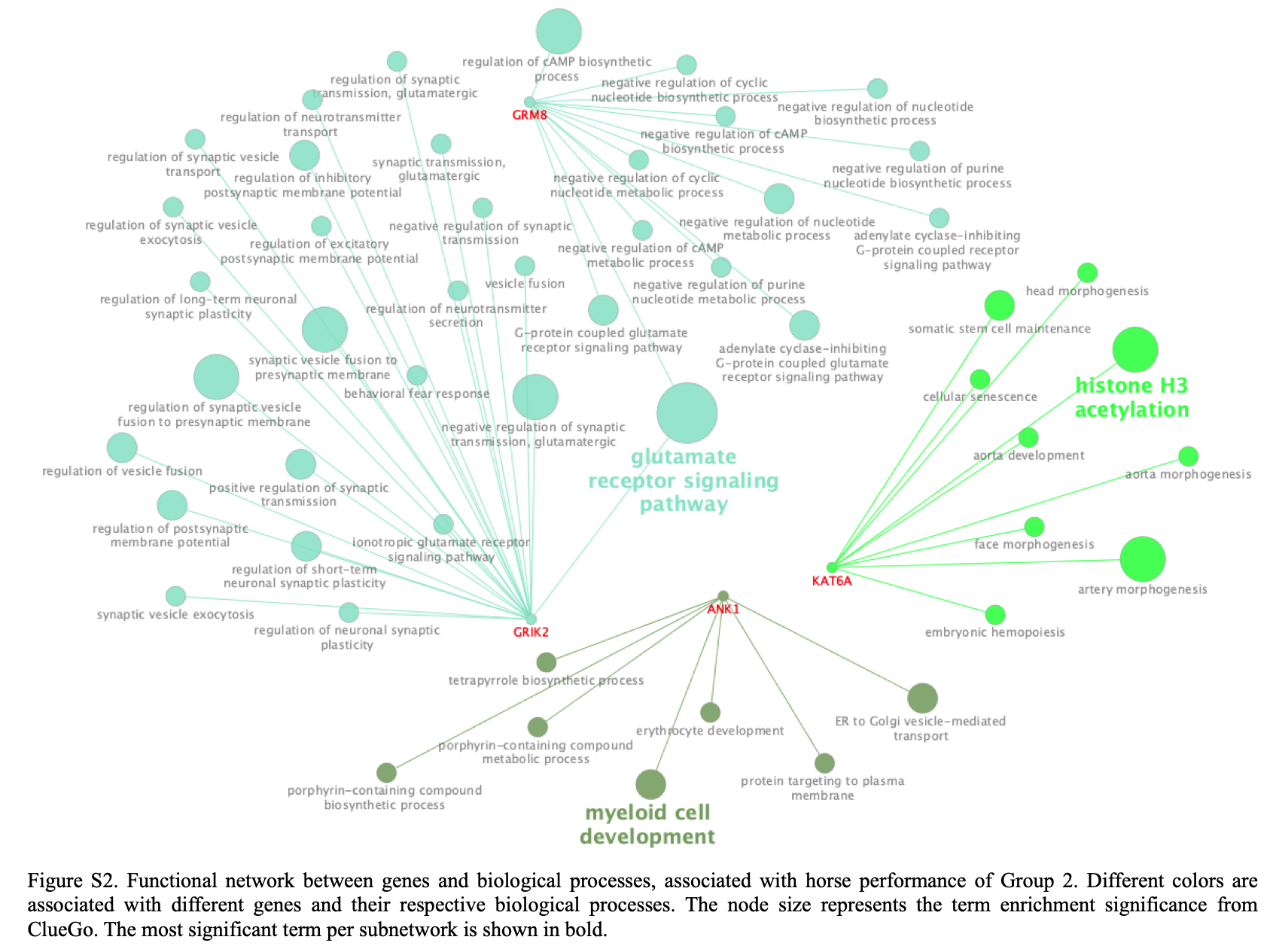

Supplement: Supplementary file 1 [file animals-10-01173-s001.zip › supplementarymaterials/Figure S2.png]

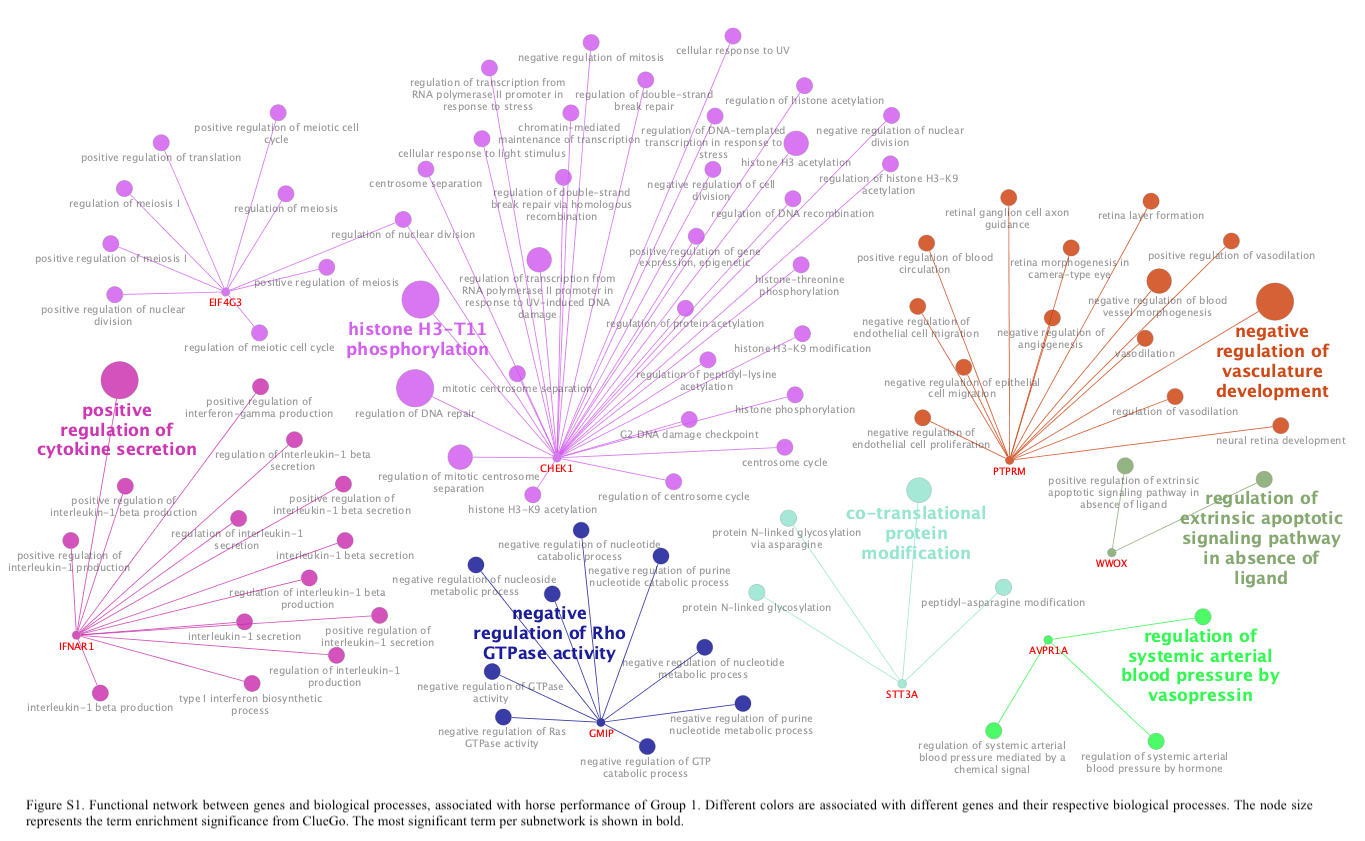

Supplement: Supplementary file 1 [file animals-10-01173-s001.zip › supplementarymaterials/Figure S1.png]

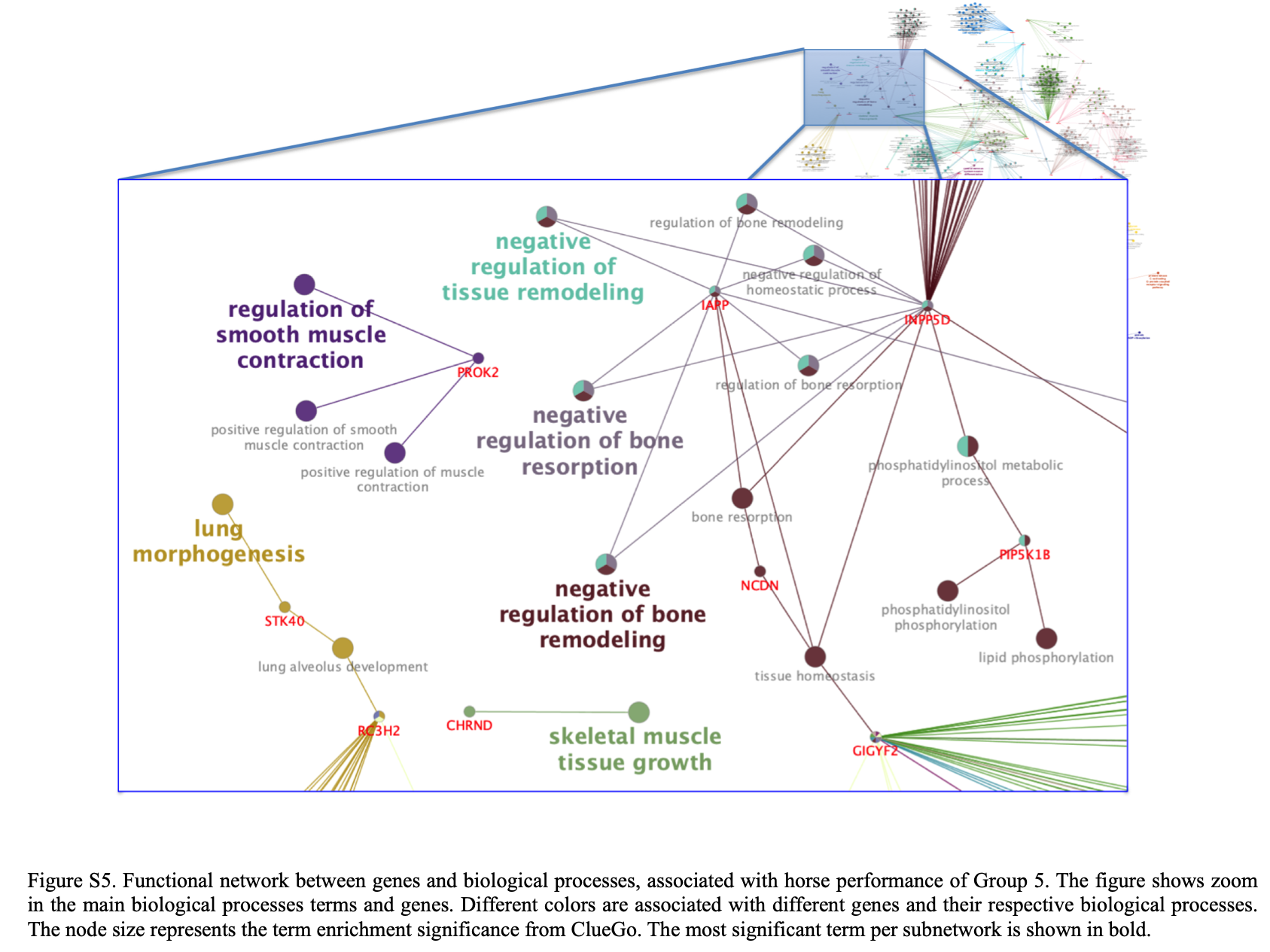

Supplement: Supplementary file 1 [file animals-10-01173-s001.zip › supplementarymaterials/Figure S5.png]

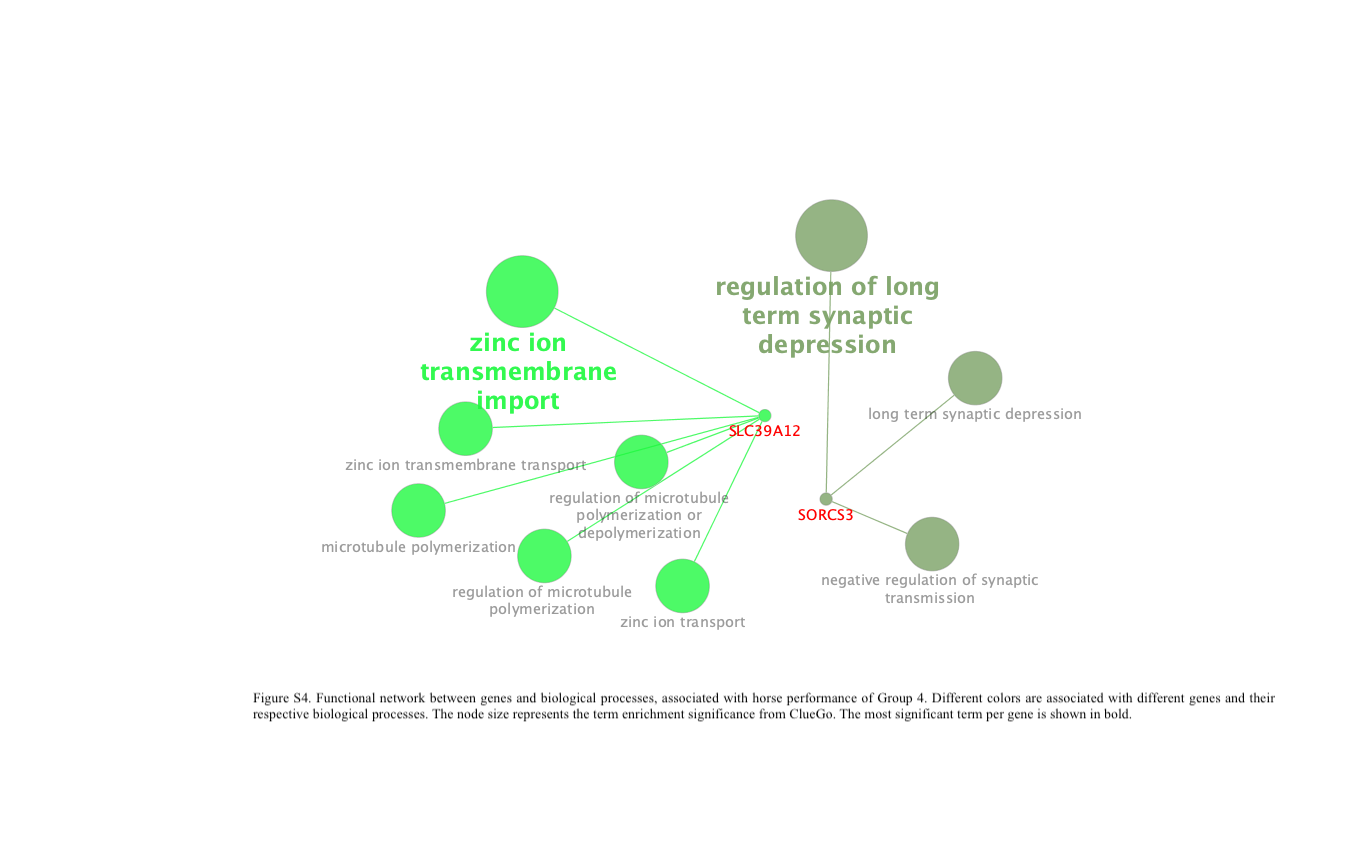

Supplement: Supplementary file 1 [file animals-10-01173-s001.zip › supplementarymaterials/Figure S4.png]

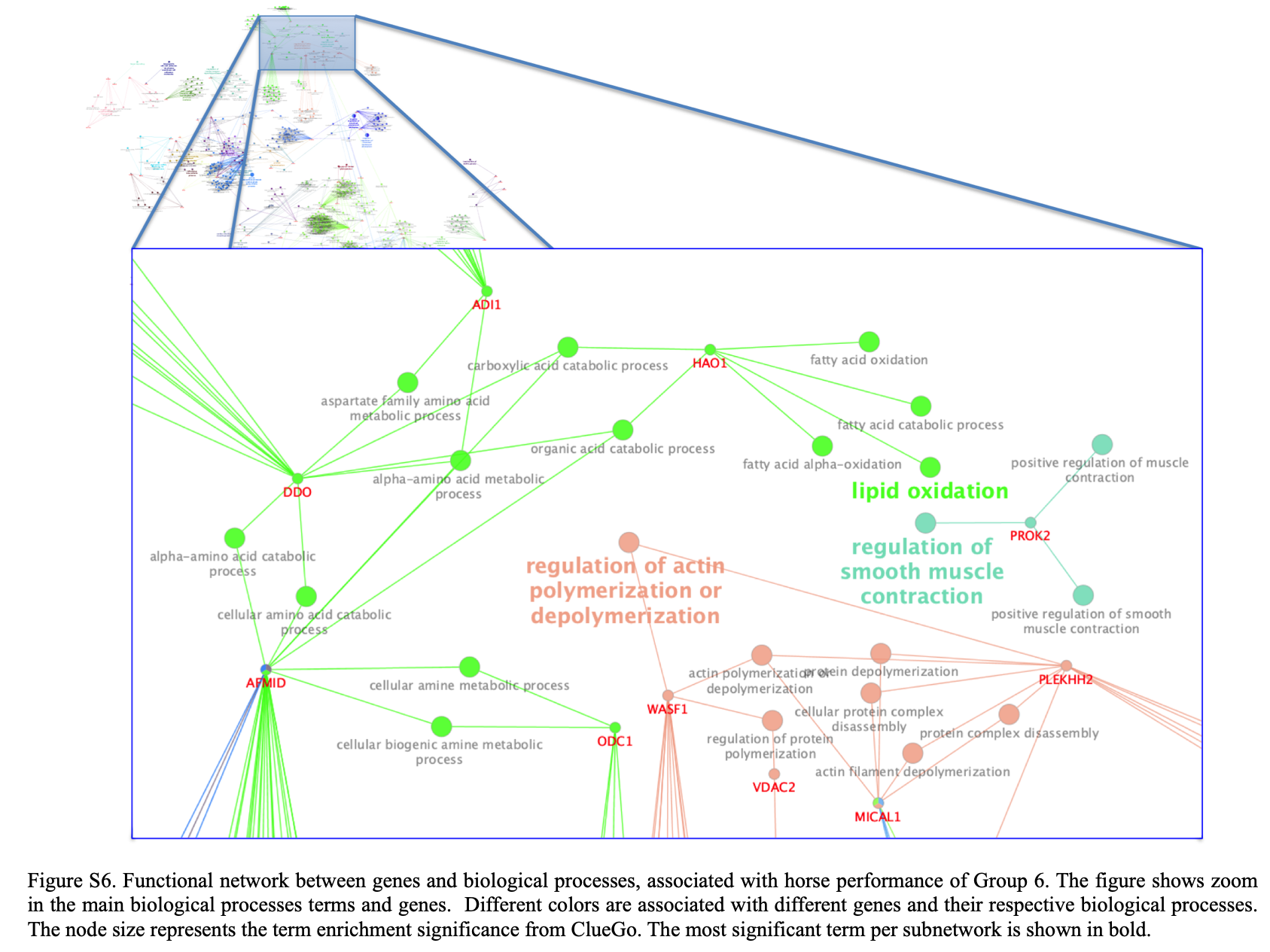

Supplement: Supplementary file 1 [file animals-10-01173-s001.zip › supplementarymaterials/Figure S6.png]

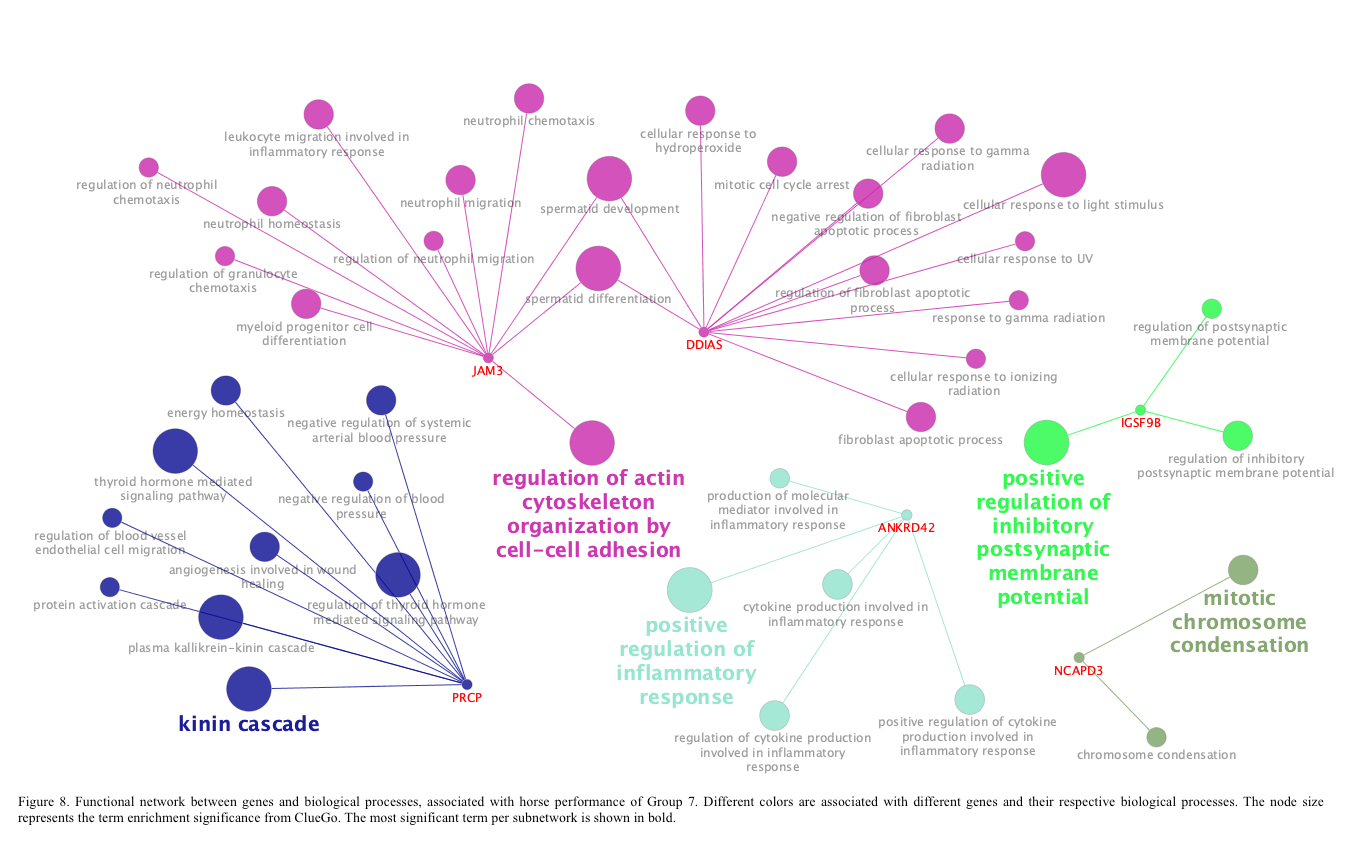

Supplement: Supplementary file 1 [file animals-10-01173-s001.zip › supplementarymaterials/Figure S7.png]
